# Harnessing Methyltransferase‐Guided Targeting for Sequence‐Specific Proximity Labeling of DNA

**DOI:** 10.1002/anie.202520412

**Published:** 2026-03-01

**Authors:** Xiong Chen, Gang Wen, Niels Ooghe, Sergey Abakumov, Taoufik Rohand, Volker Leen, Peter Dedecker, Tanja Weil, Johan Hofkens

**Affiliations:** ^1^ Department of Chemistry KU Leuven Leuven Belgium; ^2^ Department of Biotechnology and Biophysics Biocenter University of Würzburg Würzburg Germany; ^3^ Applied Chemistry Team FSTH Abdelmalek Essaâdi University Tetouan Morocco; ^4^ Perseus Biomics B.V Leuven Belgium; ^5^ Max Planck Institute for Polymer Research Mainz Germany

**Keywords:** DNA methyltransferase, methylation‐independent, proximity labeling, SAM analogues, sequence‐specific

## Abstract

Methyltransferase (MTase)‐based DNA labeling has become a powerful strategy for genomic and epigenetic analysis because of its unique ability to recognize and functionalize DNA sequences in a site‐specific manner. Expanding this toolbox is essential to fully exploit MTases as programmable molecular guides. Here, we introduce an MTase‐directed **proximity labeling** approach that enables sequence‐specific DNA modification beyond the natural catalytic transfer site: **GLOW**, Guided Labeling Outside the natural site With MTases. Using newly designed *S*‐adenosyl‐L‐methionine (SAM) analogues, we demonstrate sequence‐specific DNA labeling revealed by single‐molecule fluorescence imaging and gel‐based restriction enzyme assays, confirming that labeling occurs adjacent to, rather than within, the canonical recognition site. Unlike conventional MTase‐mediated methods, this strategy provides enhanced ligand stability and avoids interference from endogenous DNA methylation, thereby broadening its potential to complex genomic contexts. These findings establish MTase‐guided proximity labeling as a conceptually new mode of enzymatic targeting that enriches the chemical biology toolkit for sequence‐specific DNA modification.

## Graphical abstract

A novel methyltransferase‐mediated approach leveraging rationally designed *S*‐adenosyl‐L‐methionine (SAM) analogues was developed to achieve sequence‐specific DNA labeling but in proximity to the natural transfer site, addressing key limitations of previously established methods, including ligand instability and methylation‐sensitive labeling.

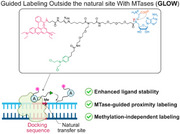

## 1 Introduction

DNA methyltransferases (MTases) catalyze the transfer of a methyl group to DNA, a fundamental epigenetic modification that regulates gene expression in living organisms [1, 2]. Such modification is accomplished through the catalytic transmethylation activity of MTases, which make use of the natural cofactor *S*‐adenosyl‐L‐methionine (SAM) as the methyl donor [3]. The exquisite accuracy of DNA methylation is conferred by MTases, which have evolved as molecular guides that recognize defined DNA sequences and selectively target them for modification. A range of studies have determined the structural features underlying this high specificity. Crystal studies have revealed that MTases, as represented by the C5‐cytosine MTase M.*Hha*I [4] and the N6‐adenine MTase M.*Taq*I, [5] consist of two distinct domains: a catalytic domain responsible for cofactor binding and catalytic transfer, and a DNA‐recognition domain that defines sequence specificity [2]. A significant feature of the ternary complexes is that the DNA is bound in the cleft between the two domains, where the target nucleobase is flipped out of the DNA helix through the minor groove. This flipping process positions the nucleobase in the enzyme's active site and places it in close proximity to the cofactor, thereby enabling precise and efficient transmethylation. Building on this natural mechanism, MTases have also been shown to be compatible with a broad range of artificial substrates based on rationally designed SAM analogues, and can be hijacked to transfer various functional tags of interest (e.g., amine, [6, 7, 8] azide, [9, 10, 11] alkyne, [12, 13, 14] biotin, [15, 16] fluorophores, [17, 18, 19] etc.) to specific recognition sites. This powerful tool has sparked significant interest in DNA‐targeted research, and the past decade has witnessed diverse applications of MTase‐directed DNA labeling, such as DNA mapping, [20, 21] epigenetic analysis [22, 23] and biomolecule enrichment [8, 12].

**FIGURE 1:**
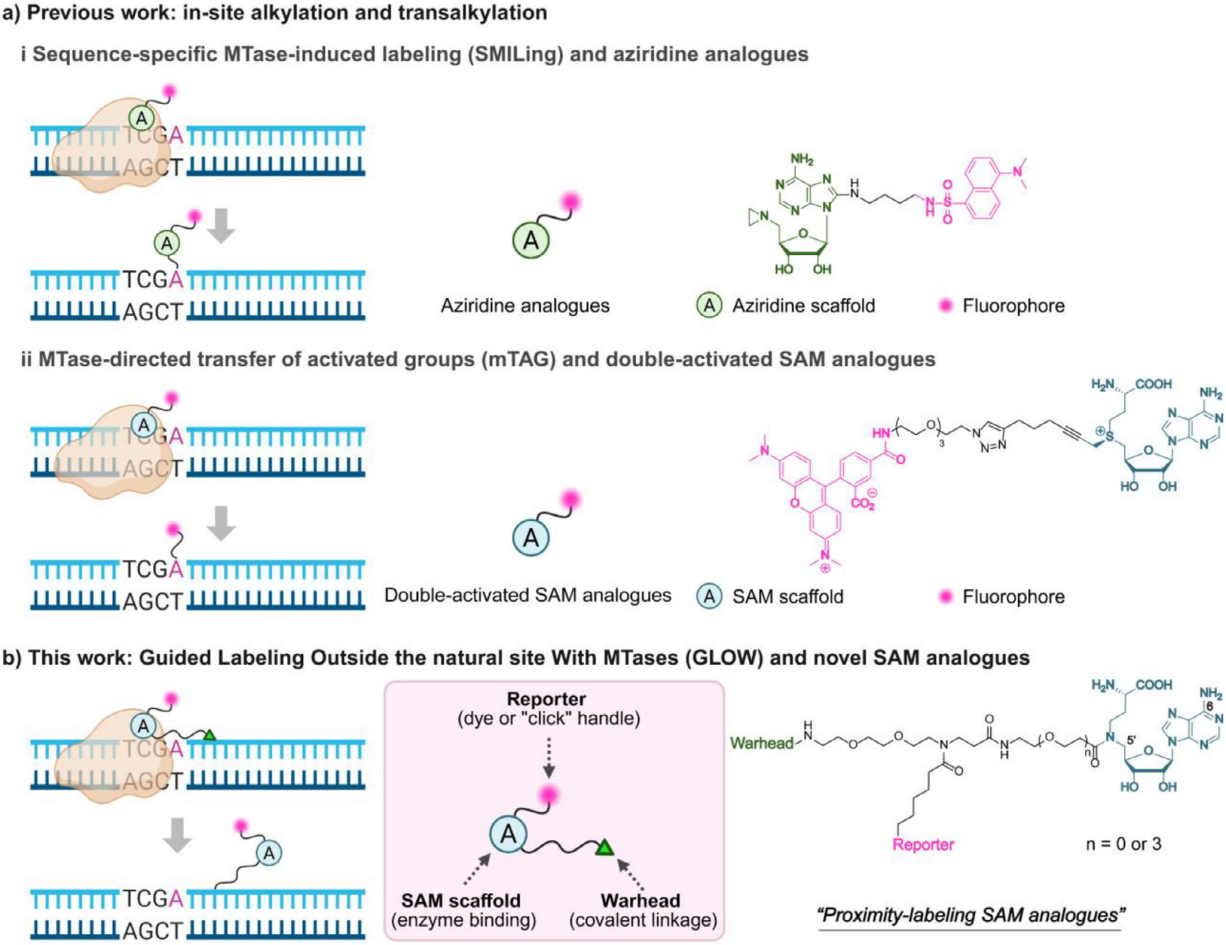
A schematic summary of various MTase‐directed DNA labeling approaches exemplified by the M.*Taq*I enzyme targeting 5′‐TCGA‐3′ sequence. (a) In‐site labeling of target DNA sequences via SMILing using aziridine analogues (i) and via mTAG using double‐activated SAM analogues (ii). (b) The GLOW concept and design of proximity‐labeling SAM analogues in this work.

Central to these advances is the rational modification of the natural cofactor SAM. Two main types of functional SAM analogues have been developed, including aziridinoadenosines [24] (Figure 1a, panel (i)) and double‐activated SAM analogues [25] (Figure 1a, panel (ii)) for sequence‐specific methyltransferase‐induced labeling (SMILing) and methyltransferase‐directed transfer of activated groups (mTAG), respectively. While powerful, both strategies suffer from limitations. These SAM analogues are unstable either due to the labile aziridine ring [26] or the sulfonium center [27]. This stability issue was mitigated by rationally modifying the chemical scaffold of SAM, such as replacement of the sulfonium center with a more stable selenium center, [12, 28] substitution of the carboxylate group with less nucleophilic amide or tetrazole moieties, [29, 30] or a combined strategy [30]. More fundamentally, the targeted nucleobase of an MTase may have been modified through natural DNA methylation processes within the host organism, rendering it unavailable for transalkylation and precluding such labeling by methodologies such as SMILing and mTAG approaches. This methylation‐sensitive feature leads to a significant limitation in that the labeling depends on the particulars of the host organisms and the myriad of processes occurring therein, thereby introducing variability and impacting downstream applications.

To address the aforementioned limitations of existing methods, we report a novel approach, GLOW (Guided Labeling Outside the natural site With MTases), that leverages the sequence detection capacity of MTases but modifies DNA at a site adjacent to, rather than within, the canonical target base. In this study, we describe the design and synthesis of proximity‐labeling SAM analogues, evaluate their labeling performance, and demonstrate their ability to achieve methylation‐independent, sequence‐specific DNA labeling. This strategy expands the functional repertoire of MTases beyond conventional cofactor analogues and establishes a new framework for exploiting MTases as programmable guides in DNA chemistry.

## 2 Results and Discussion

Existing functional SAM analogues include aziridine analogues and double‐activated SAM analogues. From a mechanistic perspective, an MTase (e.g., M.*Taq*I) can guide the cofactor ligand to the target sequence 5′‐TCGA‐3′ in the genome. Within the close proximity to the flipped adenine base enabled by the MTase, the whole aziridine ligand can be covalently attached to the target base via nucleophilic attack, causing the three‐membered aziridine ring to open [17] (Figure 1a, panel (i)). Double‐activated SAM analogues permit the direct transfer of extended groups, such as fluorophores, to the target adenine site [18] (Figure 1a, panel (ii)). This is facilitated by the unsaturated bond in the *β* position to the sulfonium center, which stabilizes the transition state (*sp3* to *sp2* hybridization) and activates the reactive carbon for the transfer reaction [25]. Both of these approaches, however, share the basic drawback that the reaction cannot proceed if the nucleobase is blocked by the previous methylation.

**FIGURE 2:**
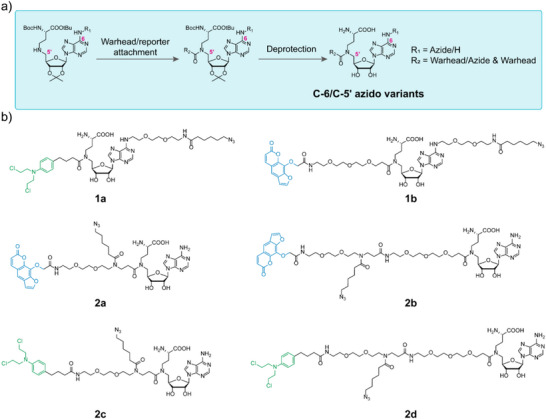
The design and synthesis of proximity‐labeling SAM analogues. (a) The synthetic strategy for the construction of multifunctional SAM analogues. (b) The chemical structures of designed and synthesized C‐6 and C‐5′ azido proximity‐labeling SAM analogues.

In this work, we instead adopt an innovative approach that is not limited to direct labeling. Deviating from conventional SAM analogues, we rationally designed and synthesized a series of proximity‐labeling analogues building on the scaffold of the natural cofactor SAM and the GLOW concept, but replaced the unstable aziridine ring or sulfonium center with a charge‐neutral and stable amide moiety to avoid main degradation pathways, including intramolecular cyclization and depurination [27, 29]. The diversified analogues incorporate a SAM scaffold for enzyme binding, a reporter group for signal readout and a linker‐extended “warhead” group for covalent linkage near target sites (Figure 1b). We hypothesized that, once the enzyme‐substrate complex is localized at the correct DNA sequence motif, the warhead can be activated and can react with the nucleobases located close to the recognition site. Since the labeling is not at the enzyme‐targeted nucleobase itself, labeling can occur independent of the DNA methylation status, while the actual labeling site is sufficiently close to the targeted nucleobase as to be essentially indistinguishable from direct labeling in many assays. The chemical modifications were introduced at the C‐6 and C‐5′ positions with flexible linkers attached as to evaluate the impact of structural variations on labeling performance. For the C‐6 azido variants, the fully protected adenosine scaffold with C‐6 azide modification was first prepared, followed by the introduction of the warheads and removal of all protecting groups (Figure 2a). For the C‐5′ azido variants, bifunctional linkers of varying lengths, harboring both an azide moiety and a warhead, were first prepared. Then, the linkers were coupled to the protected adenosine scaffold, resulting in trifunctional MTase ligands after full deprotection (Figure 2a). To covalently label the target DNA, the warheads, including light‐activated psoralen (see **1b**, **2a,** and **2b**) and heat‐activated chlorambucil (see **1a**, **2c,** and **2d**), were utilized for this purpose (Figure 2b). Our choice to incorporate the azide moiety allows us to evaluate DNA labeling performance through a two‐step staining procedure, including covalent labeling of DNA and subsequent fluorophore tagging via a strain‐promoted azide‐alkyne cycloaddition (SPAAC) reaction.

**FIGURE 3:**
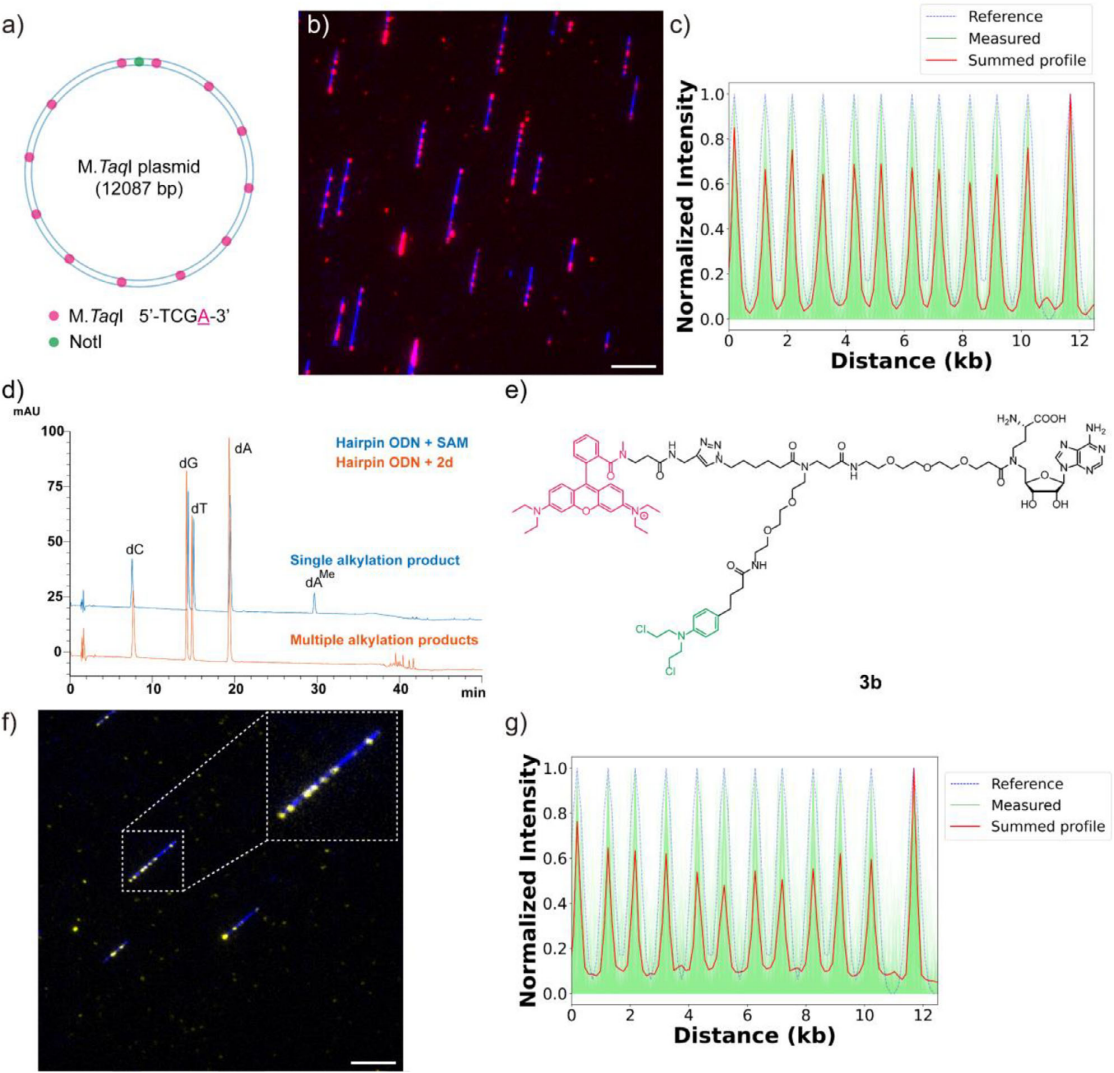
Microscopy imaging and HPLC analysis of DNA labeling using proximity‐labeling SAM analogues and M.*Taq*I enzyme. (a) The design of a 12‐site plasmid DNA (12 kb, 1 site/kb) for M.*Taq*I labeling. (b) Imaging result of M.*Taq*I labeled plasmid DNA using **2d**. (c) Consensus map generated by imaging data of **2d** labeling. 802 traces with full plasmid DNA length were analyzed. (d) Enzymatic fragmentation analysis of **SAM** and **2d** labeled hairpin ODN using M.*Taq*I. The absorption at 260 nm was detected for free (dC, dG, dT, dA) and modified nucleosides (dA^Me^ and **2d**‐modified multiple alkylation products, Me: ‐CH_3_). (e) The chemical structure of fluorescent proximity‐labeling SAM analogue **3b**. (f) Imaging result of M.*Taq*I labeled plasmid DNA using **3b**. (g) Consensus map generated by imaging data of **3b** labeling. 882 traces with full plasmid DNA length were analyzed. For **2d** labeling, DBCO‐Atto647N was used to introduce the fluorophore via SPAAC reaction. For all samples, the labeled DNA was further stained with YOYO‐1 to visualize the backbone. Red color: Atto647N. Yellow color: rhodamine B. Blue color: YOYO‐1. Scale bars: 5 µm.

With these novel analogues in hand, we next evaluated their capability for sequence‐specific DNA labeling using a custom plasmid, containing 12 evenly spaced M.*Taq*I recognition sites (5′‐TCGA‐3′) (Figure 3a). The DNA was first subjected to M.*Taq*I enzyme and a ligand, followed by fluorophore tagging via a SPAAC reaction. Using the proposed labeling strategy, we found that analogues **1a** and **1b** with C‐6 azido modification failed to label the DNA (Figure S1), indicating either undesirable enzyme compatibility or inefficient attack of nearby nucleobases by the warhead moieties. However, the psoralen variant **2b** with C‐5′ azido modification and a long linker did label the DNA (Figure S2a–c), whereas the negative control without M.*Taq*I enzyme showed no DNA labeling (Figure S2d–f), providing the first glimpse on the possible guiding role of the M.*Taq*I enzyme. A possible explanation for such a difference, as suggested by previous x‐ray [5] and modeling [17] studies, is that the C‐6 position is less exposed to the solvent region compared to the C‐5′ position (Figure S3), which may limit the flexibility of chemical modifications. Strikingly, the psoralen variant **2a** with C‐5′ azido modification and a short linker failed to yield covalent labeling (Figure S2g–i). However, the chlorambucil variant **2c** demonstrated MTase‐dependent DNA labeling despite having the same linker length as **2a** (Figure S4a). Also, the chlorambucil variant **2d** with a long linker displayed robust labeling (Figure 3b). It should be noted that the imaging results indicated incomplete labeling of all 12 sites, possibly reflecting a DNA structure dependence that is still being investigated. The sequence specificity of labeling was confirmed by generating a consensus map, which shows completely matched patterns to the reference profile (Figure 3c). These observations suggest that the alkylating agent chlorambucil is more suitable than the intercalating agent psoralen for MTase‐directed proximity labeling, even though psoralen agents achieved excellent labeling performance when combined with minor groove binders [31]. It is well known that psoralen undergoes a photocycloaddition reaction with thymine upon irradiation at 365 nm [32]. After scrutinizing the sequence of M.*Taq*I plasmid, 10 of the 12 sites containing a thymine within 6 base pairs of the recognition sites were identified. This inefficiency of labeling may be explained by the requirement for the psoralen moiety to adopt a specific orientation to intercalate into the double‐stranded helix, which can be more difficult when the ligand is bound to the MTase and thus not positioned in the groove as compared to minor‐groove‐binding conjugates.

As apparent from visual analysis of the DNA images (Figure 3), the labeling of the sequence sites does not reach completion under the reported conditions. To gain further insights, we reverted to a fluorescence counting assay based on discrete labeled spots on DNA plasmid strands. Because the distance between adjacent labeling sites exceeds the optical diffraction limit (∼250 nm), individual fluorescent signals can be reliably resolved, though full quantification is complicated by fluorophore blinking [33]. Hence, for this purpose, we synthesized a fluorescent double‐activated SAM analogue **AdoYnAtto647N** (structure shown in Figure S6a) as a control, where full labeling efficiency was first confirmed by a restriction enzyme assay (Figure S6b), indicating MTase‐directed in‐site labeling and full protection of M.*Taq*I sites from endonuclease cleavage. Labeling specificity was further validated by plasmid DNA labeling and consensus mapping (Figure S6c), which showed well‐matched fluorescence distributions with the reference sequence. The sample thus prepared under conditions of complete labeling was then used as a reference for the counting assay, yielding an apparent labeling efficiency of 70.3% for **2d** (Figures S5 and S7).

Inherently, this difference will be unaffected by the underlying DNA sequence, as reactive nucleobases need to be available in the proximity of the labeling site. To uncover first insights into the mechanisms of labeling, we next sought to figure out at which nucleotide(s) the labeling actually happened. To this end, a hairpin oligodeoxynucleotide (ODN) containing a single M.*Taq*I recognition site was designed as previously reported (Figure S8a), [34] and the enzymatic fragmentation assay was first validated to confirm complete fragmentation of the hairpin ODN and appropriate HPLC conditions (Figure S8b). The suitability of the hairpin design was further validated by comparison with a double‐stranded ODN used in earlier studies (Figure S9a) [35]. Both hairpin and double‐stranded ODNs were labeled using SAM or the fluorescent cofactor **AdoYnRho110** (Figure S9b), followed by DNA purification and enzymatic fragmentation. HPLC analysis demonstrated that both ODN forms produced comparable labeling outcomes, each yielding a single product peak corresponding to the labeled adenine nucleoside (dA^Me^ or dA^Rho110^) (Figure S9C). Based on these results, the hairpin ODN was subsequently employed for MTase‐directed proximity labeling. Fragmentation analysis revealed that, unlike double‐activated cofactors tested, **2d** generated multiple product peaks (Figure 3d), indicating multiple alkylation events within 6 base pairs of the recognition sites. Previous studies have shown that chlorambucil preferentially reacts with the N7 position of guanine and the N3 position of adenine [36]. The resulting positively charged dG and dA adducts are prone to degradation, leading to depurination and loss of the sugar moiety (Figure S10) [37]. In contrast to the single, stable alkylation products formed by double‐activated cofactors, the multiple and unstable adducts generated by **2d** complicate the quantitative analysis in the fragmentation assay. However, in a genomic context and for single‐molecule imaging use, these features do not compromise the analytical accuracy due to intact genomic structures.

Encouraged by these results, we further synthesized fluorescent analogues **3a** and **3b** for direct staining of DNA (See Supporting Information and Figure 3e), thereby simplifying the labeling procedures by eliminating the need for a separate dye conjugation step. The results demonstrated that both fluorescent variants enabled site‐specific fluorophore tagging, with **3b** showing superior labeling output (Figures S4b and 3f), and the labeling specificity was validated by a consensus map of **3b** labeling (Figure 3g). Thus, **3b** was selected for subsequent studies.

**FIGURE 4:**
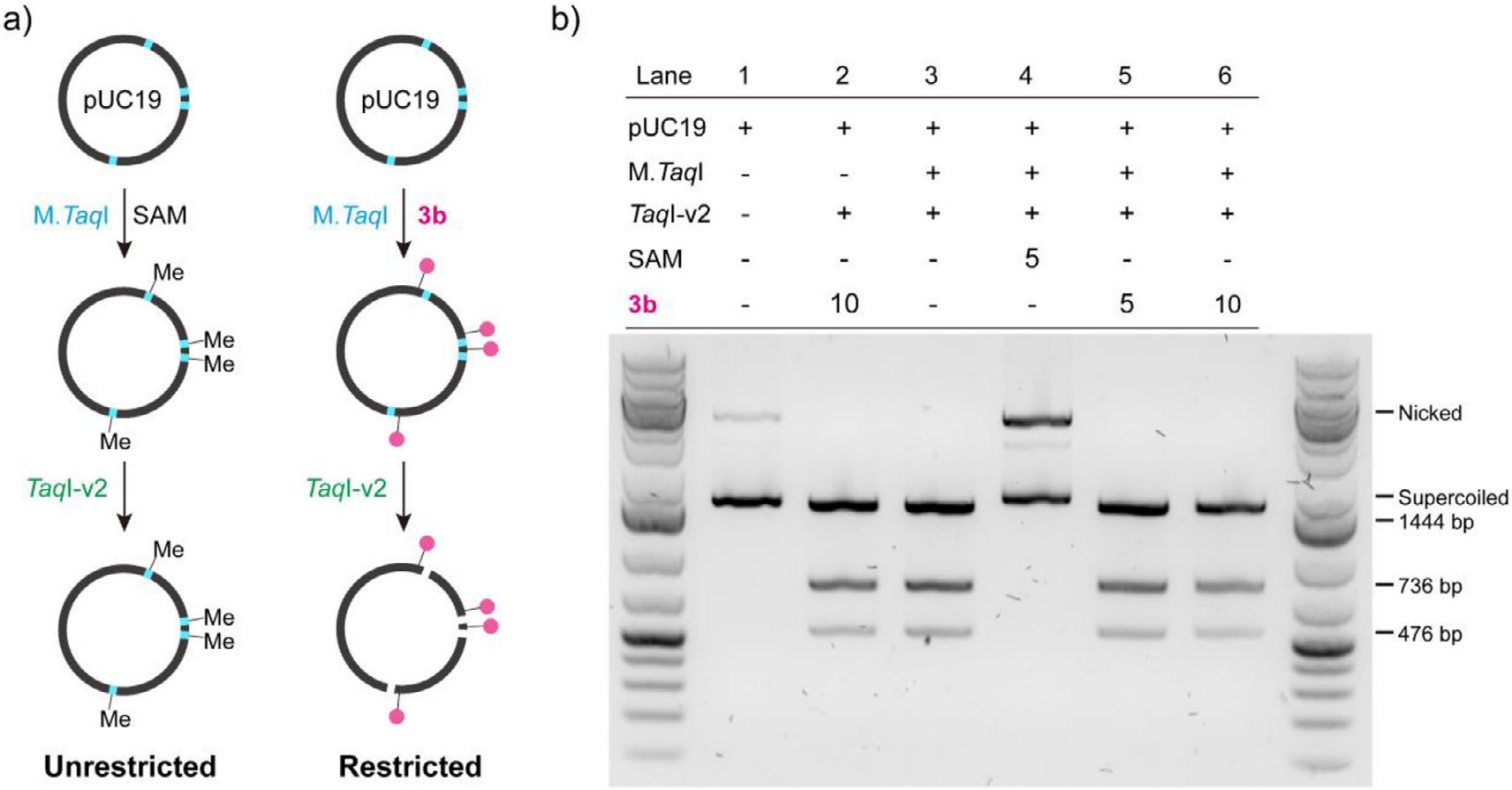
Gel‐based restriction enzyme assay to validate the proximity character of the labeling. (a) Schematic diagram of the restriction enzyme analysis. SAM was used as a control in this experiment. (b) Agarose gel restriction assay. For each sample, the components are specified in the table above. Lane 2 and lane 3 were designed as controls, either without M.*Taq*I (lane 2) or without cofactor (lane 3). For SAM, 5 µM was used for MTase‐directed labeling (lane 4). For **3b**, 5 or 10 µM were used for MTase‐directed proximity labeling (lane 2, lane 5, and lane 6). The different forms of pUC19 (nicked and supercoiled) and the length of restricted fragments (1444, 736, and 476 bp) are specified.

To validate that the DNA was indeed labeled outside the natural transfer site targeted by the enzyme, we performed a gel‐based restriction enzyme assay (Figure 4a). For comparison, natural cofactor SAM was used as a control, where the methyl (Me) group is directly transferred to M.*Taq*I recognition sites (5′‐TCGA‐3′) on the plasmid DNA, thus protecting the sites from cleavage by the restriction endonuclease *Taq*I‐v2 at the same recognition site. However, if the DNA is indeed proximity‐labeled using our methodology, then it should remain susceptible to cleavage as the target site itself is not labeled and modified. The gel‐based restriction analysis demonstrated that SAM‐modified DNA showed full protection against restriction, as revealed from lane 4 (Figure 4b), while the treatment of proximity‐labeling SAM analogue **3b** did not affect the cutting at specific recognition sites in the plasmids, as revealed from lane 5 and lane 6 (Figure 4b), directly supporting our proximity‐labeling strategy. This result was further supported by in‐gel fluorescence scanning, in which we used the azide‐containing analogue **2d** to couple to cyclooctyne‐containing Atto647N to generate distinguishable fluorescence from GelRed staining. The gel‐based restriction analysis suggested MTase‐directed DNA labeling as revealed from lane 2 (control) and lane 6 (Figure S11a,b). The pUC19 DNA labeled by **2d** was cleaved and all 3 fragments yielded fluorescence signals from Atto647N dye (Figure S11b), further supporting the sequence‐specific DNA labeling outside the transfer site.

**FIGURE 5:**
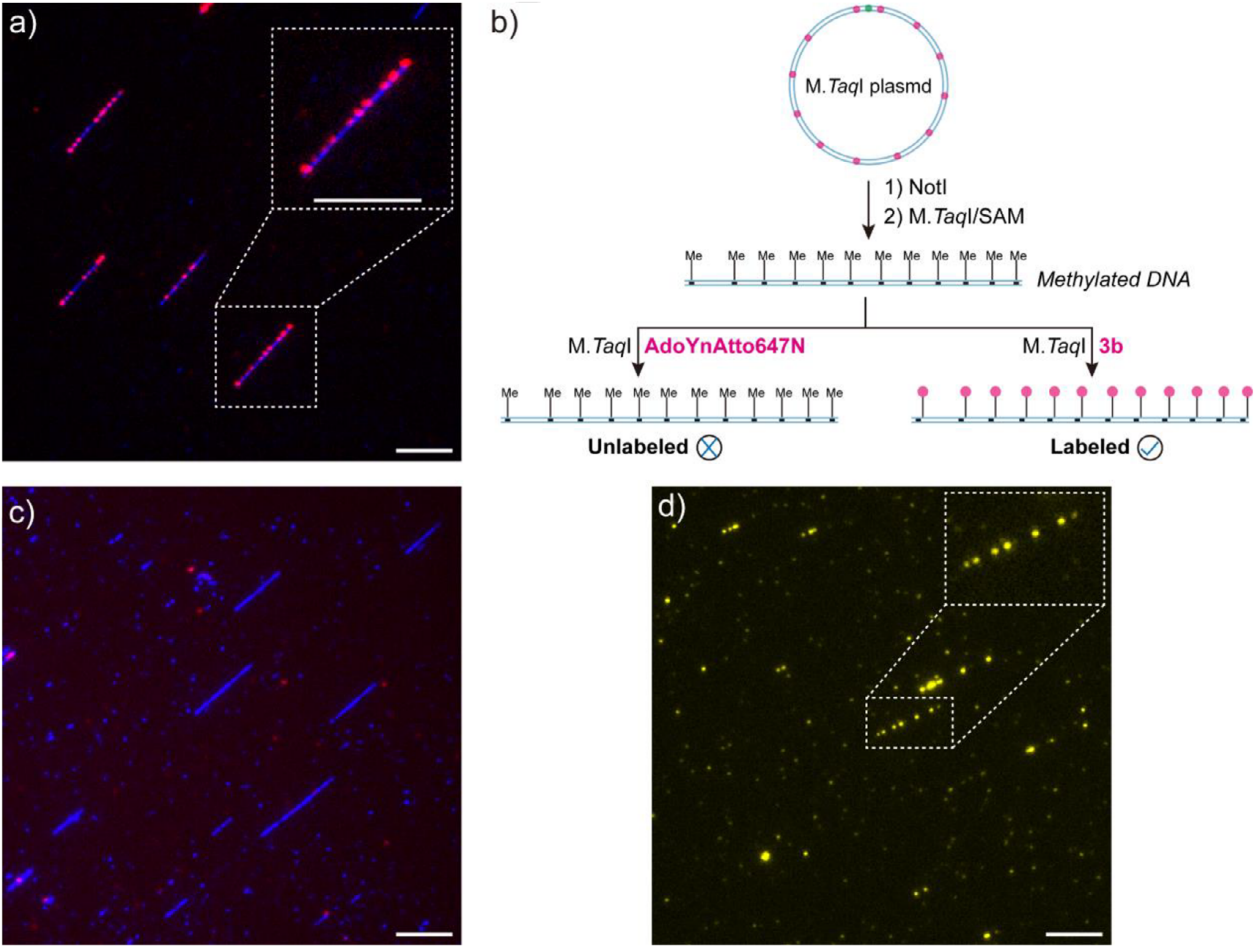
Validation of methylation‐independent DNA labeling. (a) Imaging result of M.*Taq*I labeled plasmid DNA using **AdoYnAtto647N**. (b) Schematic diagram of the methylation‐independent labeling concept. Cofactors **AdoYnAtto647N** and **3b** were used for a comparison study. (c), (d) Imaging results of M.*Taq*I labeled methylated plasmid DNA using **AdoYnAtto647N** (c) and **3b** (d). The labeled DNA was further stained with YOYO‐1 to visualize the backbone. Red color: Atto647N. Yellow color: rhodamine B. Blue color: YOYO‐1. Scale bars: 5 µm.

An additional significant advantage of the GLOW approach is the ability to highlight the presence of genomic features free of the interference of natural DNA methylation. If the target nucleobase is naturally methylated, the enzymatic transfer of functional groups to the DNA using double‐activated SAM analogues will be blocked. However, the proximity‐labeling approach may provide an opportunity to bypass this issue. To demonstrate this, we utilized the double‐activated SAM analogue **AdoYnAtto647N** as a control (Figure 5a). For the methylation‐independent labeling, we first prepared methylated plasmid DNA using SAM and M.*Taq*I enzyme. After methylation, the obtained DNA was further treated with **AdoYnAtto647N** or a fluorescent proximity‐labeling SAM analogue **3b**, respectively (Figure 5b). In line with our expectation, **AdoYnAtto647N** did not yield any observable DNA labeling due to the methylation of the target sites, and the existence of DNA was confirmed by YOYO‐1 staining (Figure 5c). However, the sequence‐specific DNA labeling pattern was still observed from the analogue **3b**, demonstrating methylation‐independent labeling of DNA (Figure 5d). This observation was also validated using methylated pUC19 and a restriction enzyme assay. The full methylation of pUC19 was indicated in the gel (Figure S12a, lane 3), showing no DNA cleavage. The fluorescent labeling of methylated pUC19 (^Me^pUC19) was achieved using **2d** after conjugation with cyclooctyne‐containing Atto647N (Figure S12b; lane 6 and lane 7), whereas the labeling failed in the case of **AdoYnAtto647N** (**Ctrl**, lane 5). Overall, these unprecedented results highlight the unique advantage of the proximity‐labeling strategy, which can provide accurate site information even though potential methylation status is present in the treated DNA species. This methylation‐independent activity distinguishes GLOW from conventional MTase‐based strategies and broadens its potential for applications in native genomic contexts.

## 3 Conclusion

In summary, in stark difference to previously established in‐site MTase‐directed labeling methods, we developed **GLOW** (Guided Labeling Outside the natural site with MTases), an MTase‐directed proximity labeling strategy, utilizing a novel type of MTase ligands. By exploiting the sequence detection capacity of M.*Taq*I enzyme while positioning reactive groups outside the canonical transfer site (within nanometer distances), GLOW achieves sequence‐specific DNA labeling in proximity to natural recognition sites. Importantly, this approach circumvents interference from endogenous DNA methylation, a key limitation of conventional MTase‐mediated strategies. Together, these features establish GLOW as a robust and versatile addition to the DNA modification toolbox. Future studies on optimizing the “warhead” chemistry, expanding the approach to a broader repertoire of MTases, and probing sequence‐dependence effects will further enhance labeling efficiency and broaden the applicability of this approach. Beyond methodological refinement, GLOW opens new avenues for genome mapping, epigenetic profiling, and single‐molecule imaging, further extending the privileged potential of MTases.

## Conflicts of Interest

The authors declare the following competing financial interest(s): Volker Leen holds the patent rights to some of the inventions described in this study.

## Supporting information



The authors have cited additional references within the Supporting Information [7, 21, 31, 35, 37–39]**Supporting File 1**: Anie71700‐sup‐0001‐SuppMat.pdf.

## Data Availability

The data that support the findings of this study are available in the supplementary material of this article.
