# Supplementary material for: Harnessing Methyltransferase‐Guided Targeting for Sequence‐Specific Proximity Labeling of DNA
Source: Angew Chem Int Ed Engl. 2026 Mar 1;65(15):e20412. doi: 10.1002/anie.202520412 (PMC13053919; doi:10.1002/anie.202520412)
Supplement: Supplementary file 1 — The authors have cited additional references within the Supporting Information [7, 21, 31, 35, 37–39]Supporting File 1: Anie71700‐sup‐0001‐SuppMat.pdf. [file ANIE-65-e20412-s001.pdf]

Supporting information

Harnessing Methyltransferase-Guided Targeting for Sequence-Specific Proximity Labeling of DNA

Xiong Chen,[a] Gang Wen,[b] Niels Ooghe,[a] Sergey Abakumov,[a] Taoufik Rohand,[c] Volker Leen,[d] Peter Dedecker,[a] Tanja Weil,[e] and Johan Hofkens*[a],[e]

[a] Department of Chemistry, KU Leuven, Leuven 3001, Belgium

[b] Department of Biotechnology and Biophysics, Biocenter, University of Würzburg, Am Hubland, Würzburg 97074, Germany

[c] Applied Chemistry team, FSTH, Abdelmalek Essaâdi University, Tetouan 93000, Morocco

[d] Perseus Biomics B.V., Leuven 3001, Belgium

[e] Max Planck Institute for Polymer Research, Mainz 55128, Germany

*E-mail correspondence to: johan.hofkens@kuleuven.be

Table of Contents

Page

1. ***Biological Experiments*……………………………..……………………………….....2**
2. ***Supporting Tables and Figures* ………………………….………………………..6**
3. ***Synthesis and Characterization* ……………………………………………………...15**
4. ***Copies of ^{1}H and ^{13}C NMR Spectra* ………………………………………………..44**
5. ***References* ………………………………………………………………………………..82**

1 Biological Experiments

Proximity Labeling of DNA Using Synthesized SAM Analogues

For M.*Taq*I plasmid labeling, the DNA was first linearized via treatment with the restriction endonuclease NotI-HF. 1 µg of circular plasmid DNA (100 ng/µL in Milli-Q, 10 µL), 16 µL of Milli-Q, 3 µL of rCutsmart buffer (10X, NEB) and 1 µL of NotI-HF enzyme (20,000 units/mL, R3189L, NEB) were added into a 0.5 mL of DNA low-bind tube to achieve a total reaction volume of 30 µL. The mixture was always homogenized by gentle pipetting with a wide-bore pipet tip to minimize shearing of DNA. The sample was incubated at 37 °C, 350 rpm for 45 min to complete the restriction, followed by heating at 65 °C, 350 rpm for 20 min to inactivate the enzyme. Subsequently, to the linearized plasmid DNA above were added 6.25 µL of Milli-Q, 1 µL of rCutsmart buffer (10X final), 0.15 µg/µL of M.*Taq*I enzyme (2.67 µg/µL, 2.25 µL) and 5 µM of the proximity-labeling SAM analogue (0.4 mM, 0.5 µL) to achieve a total reaction volume of 40 µL. For the negative control samples, M.*Taq*I enzyme was not added. To initiate MTase-directed labeling, the warheads attached were activated under specified conditions. For SAM analogues **1b**, **2a**, and **2b**, the samples were irradiated under 365 nm for 45 min. For SAM analogues **1a**, **2c**, **2d**, **3a** and **3b**, the samples were incubated at 37 °C, 350 rpm for 12 h. After completion of the labeling, 2 µL of proteinase K (800 units/mL, P8107S, NEB) was added, and the samples were allowed to incubate at 50 °C, 350 rpm for 1 h to digest the enzyme. For SAM analogues **1a**, **1b**, and **2a-2d**, 1 µL of DBCO-Atto647N (1 mM in DMSO, ATTO-TEC) and 9 µL of DMSO were added to the reaction mixture, followed by incubation at 25 °C, 350 rpm for 8 h to complete SPAAC reaction. For SAM analogues **3a** and **3b**, the fluorescent dye was pre-coupled and the samples were directly subject to DNA purification.

Purification and Deposition of Labeled DNA

The labeled DNA sample was purified by agarose plug washing and membrane dialysis as specified previously.[1] Before fluorescence imaging, the purified DNA was further stained with the DNA intercalating dye, YOYO-1, to visualize the DNA backbone. 20 ng of purified DNA sample was incubated with 1 µM of YOYO-1 in 50 mM MES buffer (pH 5.6), followed by heating at 50 °C for 15 min. The sample was ready for imaging after deposition onto Zeonex-coated glass coverslips via molecular combing as previously described.[2]

Microscopy Imaging

A Nikon Eclipse Ti2 microscope, equipped with a perfect focus system, was used for imaging of prepared coverslips. The excitation was performed with an Oxxius L6Cc laser combiner, using the wavelengths of 488, 561, and 640 nm for YOYO-1, rhodamine B and Atto647N, respectively. The emitted light was collected by a Nikon CFI Apochromat TIRF 100XC Oil objective with a 100× magnification and an NA of 1.49, and projected onto a PCO Edge 4.2 camera with a final virtual pixel size of 78.6 nm. For the labeled DNA with YOYO-1 staining, images were first collected using a 640 nm laser with the Chroma ET670/50m emission bandpass filter, followed by a 488 nm laser with the ET525/50m emission bandpass filter. For exposure time, 0.2 s was used for imaging.

Consensus Map

Multi-channel DNA tile scan images were processed using a custom Python pipeline. DNA strands were segmented from the YOYO-1 channel using Otsu thresholding and size filtering (11–13 kb). Fluorescence intensity profiles were then extracted from the M.*Taq*I channel and normalized for each segmented region. The reference trace was generated by first assigning a label to each M.*Taq*I recognition site (5′-TCGA-3′), followed by convolution with a Gaussian function. The full width at half maximum (FWHM) of the Gaussian matched the theoretical FWHM of the point spread function of the objective lens (CFI Apochromat TIRF 100XC Oil, NA 1.49) at the emission wavelength of the bound fluorophore. Subsequently, the extracted intensity profiles were aligned to the theoretical reference trace using a cross-correlation algorithm based on expected M.*Taq*I labeling sites.[3] Finally, the aligned profiles were aggregated to generate a consensus intensity map. The analysis was performed using NumPy, SciPy, scikit-image, and Matplotlib.

Fluorescence Counting Assay

Labeled plasmid DNA was stained with a sequence-specific Atto647N fluorophore from **2d** or **AdoYnAtto647N** labeling, and counterstained with YOYO-1 to visualize the DNA backbone. Image analysis was performed by first segmenting individual plasmid molecules using the YOYO-1 channel. Segmentation was achieved by Otsu thresholding and size filtering to select DNA molecules within the expected length range (11–13 kb), thereby excluding fragmented or aggregated species. For each segmented DNA molecule, fluorescence intensity profiles along the DNA contour were extracted from the Atto647N channel and subsequently normalized. The experimental intensity profiles were then aligned to a theoretical reference profile corresponding to the 12 M.*Taq*I recognition sites (5′-TCGA-3′) on the plasmid. Alignment was performed using a cross-correlation algorithm, enabling precise mapping of fluorescence signals to their expected genomic positions. Following alignment, the presence of fluorescence signals at each recognition site was recorded for all analyzed DNA traces. Labeling efficiency at each site was quantified as the percentage of detected fluorescence signals, calculated as the ratio of detected signals to the total number of analyzed DNA traces.

Enzymatic Fragmentation and HPLC analysis

5 µM of hairpin ODN (0.1 mM, 2.5 µL, IDT) and 47.5 µL of Milli-Q were added into a 0.5 mL of DNA low-bind tube to achieve a total volume of 50 µL. Next, 0.5 µL of Nuclease P1 (NEB, 100,000 units/mL) and 5.5 µL of Nuclease P1 reaction buffer (NEB, 10X) were added, followed by incubation at 37 °C, 350 rpm for 4 h. After that, 1 µL of Quick CIP (NEB, 5,000 units/mL) and 6.2 µL of rCutsmart buffer (10X, NEB) were added, followed by incubation at 37 °C, 350 rpm for 30 min. Upon completion of the fragmentation, the reaction mixture is heated at 80 °C, 350 rpm for 10 min, followed by centrifugation at 19,000 g for 30 min. Centrifugate samples were then analyzed by HPLC using a reverse C18 column (2 µm, 2.1 × 100 mm). The eluting solvents consist of acetonitrile and H_2O (50 mM ammonium formate) with a flow of 0.2 mL/min. The eluting procedure involves a gradient as follows: 0−5% acetonitrile (0-10 min); 5% acetonitrile (10-15 min); 5-10% acetonitrile (15-30 min); 10-70% acetonitrile (30-40 min); 70% acetonitrile (40-50 min). The injection volume is 5 µL. As controls, commercially ordered deoxyadenosine (dA), deoxythymidine (dT), deoxycytidine (dC), and deoxyguanosine (dG) were also analyzed by the same HPLC method. For the analysis of the complete hairpin ODN,

a different eluting procedure was performed as follows: 0−70% acetonitrile (0-40 min); 70% acetonitrile (40-50 min).

Validation of the Hairpin ODN Design and Proximity Labeling

To validate the design of the hairpin ODN, MTase-directed DNA labeling and fragmentation analysis were performed using both the hairpin ODN and the reported double-stranded ODN (ds ODN).[4] Both ODNs contain one M.*Taq*I recognition site (5′-TCG<u>A</u>-3′). For the cofactors, SAM and the double-activated **AdoYnRho110** were used. For SAM labeling, 5 µM of hairpin ODN (0.1 mM, 2.5 µL, IDT) or ds ODN (0.1 mM, 2.5 µL, IDT), 36.5 µL of Milli-Q, 5 µL of rCutsmart buffer (10X, NEB), 5.57 µM of M.*Taq*I (55.7 µM, 5 µL), and 20 µM of SAM (1.0 mM, 1 µL) were added into a 0.5 mL of DNA low-bind tube to achieve a total volume of 50 µL. For **AdoYnRho110** labeling, 5 µM of hairpin ODN (0.1 mM, 2.5 µL, IDT) or ds ODN (0.1 mM, 2.5 µL, IDT), 35.3 µL of Milli-Q, 5 µL of rCutsmart buffer (10X, NEB), 5.57 µM of M.*Taq*I (55.7 µM, 5 µL), and 20 µM of **AdoYnRho110** (0.46 mM, 2.2 µL) were added into a 0.5 mL of DNA low-bind tube to achieve a total volume of 50 µL. The reaction mixtures were incubated at 37 °C, 350 rpm for 12 h.

To test the proximity-labeling SAM analogue **2d**, 5 µM of hairpin ODN (0.1 mM, 2.5 µL, IDT), 36.5 µL of Milli-Q, 5 µL of rCutsmart buffer (10X, NEB), 5.57 µM of M.*Taq*I (55.7 µM, 5 µL), and 20 µM of **2d** (1.0 mM, 1 µL) were added into a 0.5 mL of DNA low-bind tube to achieve a total volume of 50 µL.

Upon completion of the reaction, the labeled ONDs were purified by Amicon Ultra centrifugal filters (0.5 mL, 10K, Merck Millipore). More specifically, 450 µL of Milli-Q was added to the reaction mixtures and the samples were then loaded on the Amicon filter, followed by centrifugation at 14,000 g for 20 min. Then the filtrate was removed and 500 µL of Milli-Q was loaded on the filter, followed by centrifugation at 14,000 g for 20 min. After that, the filters were put upside down in new tubes and centrifuged at 1,000 g for 2 min to recover the labeled ODNs. The obtained volume was around 30 µL and additional Milli-Q was added to achieve a volume of 50 µL, followed by the enzymatic fragmentation assay and HPLC analysis described earlier. Free and methylated nucleosides (dA^{Me}) were detected at 260 nm. Fluorescently modified nucleoside (dA^{Rho110}) was detected at both 260 nm and 510 nm. The labeling ratios were calculated based on the integration of peaks and respective extinction coefficients at 260 nm (dC: 7,009 mol L^{-1} cm^{-1}; dG: 11,715 mol L^{-1} cm^{-1}; dT: 8,902 mol L^{-1} cm^{-1}; dA: 15,663 mol L^{-1} cm^{-1}; dA^{Me}: 15,663 mol L^{-1} cm^{-1}),[4,5] and at 510 nm (dA^{Rho110}: 80,000 mol L^{-1} cm^{-1}).

Gel-Based Restriction Enzyme Assay

Multiple DNA reaction mixtures, as specified in the tables above the gel images, were prepared by mixing pUC19 DNA (1 µg), Milli-Q, rCutsmart buffer (10X, NEB), cofactor and M.*Taq*I (2.67 µg/µL) in a typical reaction volume of 20 µL. The final DNA concentration was 50 ng/µL and M.*Taq*I concentration was 0.15 µg/µL. Natural SAM (5 µM) or SAM analogues **3b** (5 and 10 µM), **2d** (5 and 10 µM), and **AdoYnAtto647N** (2.5-30 µM) were used for DNA labeling. All reaction mixtures were incubated at 37 °C, 350 rpm for 12 h, followed by proteinase K (800 units/mL, P8107S, NEB) treatment at 50 °C for 1 h. For **2d**, 1 µL of DBCO-Atto647N (1 mM in DMSO, ATTO-TEC) and 4 µL of DMSO were added to the reaction mixture, followed by incubation at 25 °C, 350 rpm for 8 h to introduce the fluorescent dye. Next, the

reaction mixtures were purified by the Genejet PCR Purification Kit (K0702, Thermo Scientific) according to the protocol provided by the manufacturer. The concentrations of purified DNA samples were measured by Biodrop, and the purified DNA samples were then subjected to restriction enzyme treatment. The reaction mixtures were prepared by mixing purified DNA above (200 ng), Milli-Q, rCutsmart buffer (10X, NEB) and *Taq*I-v2 (20,000U/mL, R0149S, NEB) in a typical reaction volume of 20 µL. 10 units of the restriction enzyme *Taq*I-v2 were used. Then, all reactions were incubated at 65 °C, 350 rpm for 30 min to complete the restriction step. For the gel to show methylation-independent labeling (**Figure S12**), the methylated pUC19 DNA (^{Me}pUC19) was first prepared using SAM (5 µM) and M.*Taq*I (0.15 µg/µL). The purified ^{Me}pUC19 DNA was then used for the restriction gel assay using the same procedures specified above.

The restricted DNA samples were subsequently analyzed by agarose gel electrophoresis. To prepare 1% agarose gel, 0.7 g agarose was dissolved in 70 mL of 1X TAE buffer by microwave heating until a clear solution was obtained. Then, the hot gel solution was slightly cooled down, followed by the addition of 3.5 µL of DNA staining dye GelRed. The resulting gel solution was mixed well and poured into the rack. The gel solution solidified after 30 min. Before loading the samples for electrophoresis, 4 µL of loading dye (6X) was added to the restricted DNA samples to mix well. The electrophoresis was performed at 100 V for 35 min. Finally, the agarose gel was visualized by in-gel fluorescence scanning to detect DNA staining from GelRed and Atto647N dye.

Proximity Labeling of Methylated Plasmid DNA

The circular M.*Taq*I plasmid was first linearized via treatment with the restriction endonuclease NotI-HF. Then, to the linearized plasmid DNA were added 4.25 µL of Milli-Q, 1 µL of rCutsmart buffer (10X final, NEB), 0.15 µg/µL of M.*Taq*I enzyme (2.67 µg/µL, 2.25 µL) and 20 µM of SAM (0.32 mM, 2.5 µL) to achieve a total reaction volume of 40 µL. The mixture was always homogenized by gentle pipetting with a wide-bore pipet tip to minimize shearing of DNA. The sample was then incubated at 65 °C, 350 rpm for 1 h to complete DNA methylation at 5′-TCG<u>A</u>-3′ sites. Next, 2 µL of proteinase K (800 units/mL, P8107S, NEB) was added, and the sample was allowed to incubate at 50 °C, 350 rpm for 1 h to digest the enzyme. The methylated DNA was subject to a dialysis procedure for purification. The reaction mixture was dialyzed for 45 min on an MCE membrane (0.1 µm, Merk Millipore) floating on 1X TAE buffer, followed by transferring to a new membrane to dialyze another 45 min. Subsequently, the purified methylated DNA was labeled by proximity-labeling analogue **3b**. To the methylated DNA (20 µL, 1 µg) were added 13.25 µL of Milli-Q, 4 µL of rCutsmart buffer (10X, NEB), 0.15 µg/µL of M.*Taq*I enzyme (2.67 µg/µL, 2.25 µL) and 5 µM of **3b** (0.4 mM, 0.5 µL) to achieve a total reaction volume of 40 µL. The sample was incubated at 37 °C, 350 rpm for 12 h. After completion of the labeling, 2 µL of proteinase K (800 units/mL, P8107S, NEB) was added, and the sample was allowed to incubate at 50 °C, 350 rpm for 1 h to digest the enzyme. The sample was then purified by agarose plug washing and membrane dialysis protocol as specified above. After YOYO-1 staining, the labeled DNA was imaged by a dual-color setup with 488 nm and 640 nm lasers.

2 Supporting Tables and Figures

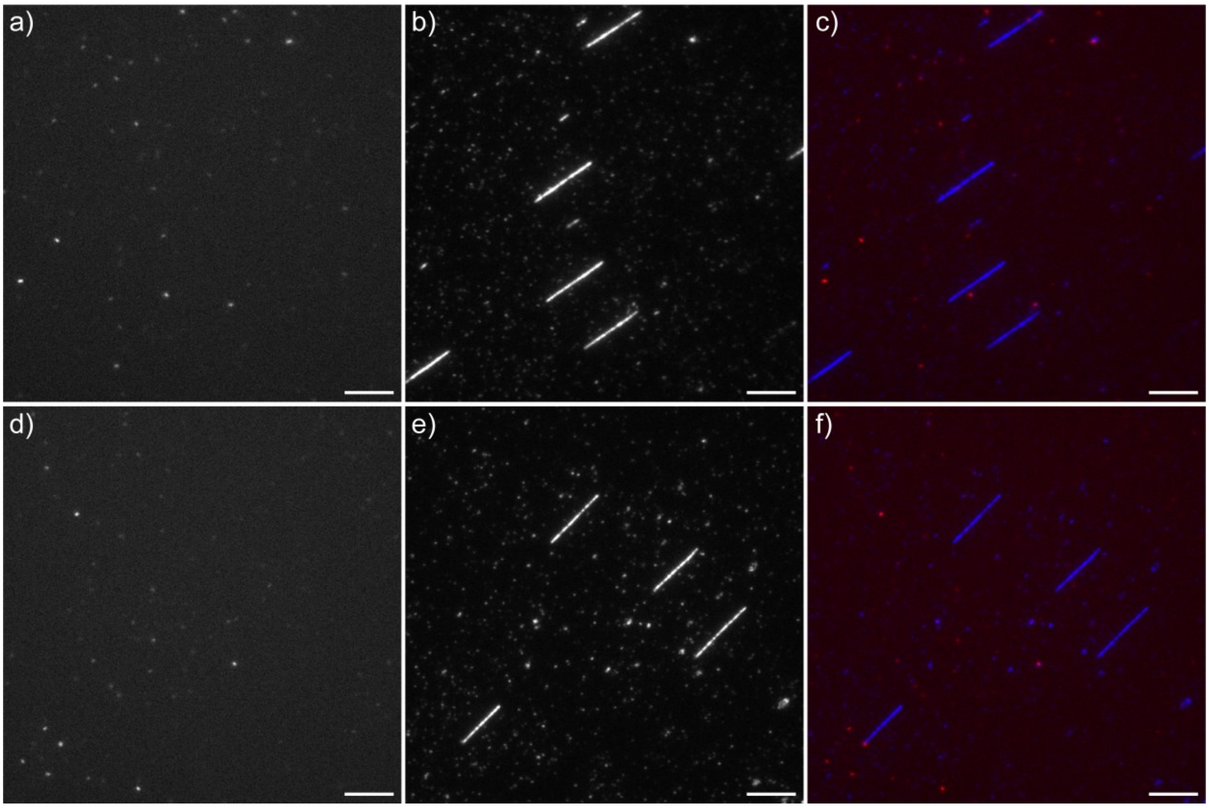

Figure S1. DNA imaging results of C-6 azido proximity-labeling SAM analogues. (a) Imaging result of analogue **1a** with plasmid DNA. (b) YOYO-1 staining of the DNA sample from **1a**. (c) Merged figure of (a) and (b). (d) Imaging result of analogue **1b** with plasmid DNA. (e) YOYO-1 staining of the DNA sample from **1b**. (f) Merged figure of (d) and (e). For DNA labeling, 5 µM of **1a** and **1b** were used, and DBCO-Atto647N was coupled by SPAAC reaction after activating reactive groups. For all samples, the labeled DNA was further stained with YOYO-1 to visualize the backbone. For all images, a widefield microscope was used. Laser channels: 488 nm (b, e), or 638 nm (a, d). Scale bars: 5 µm.

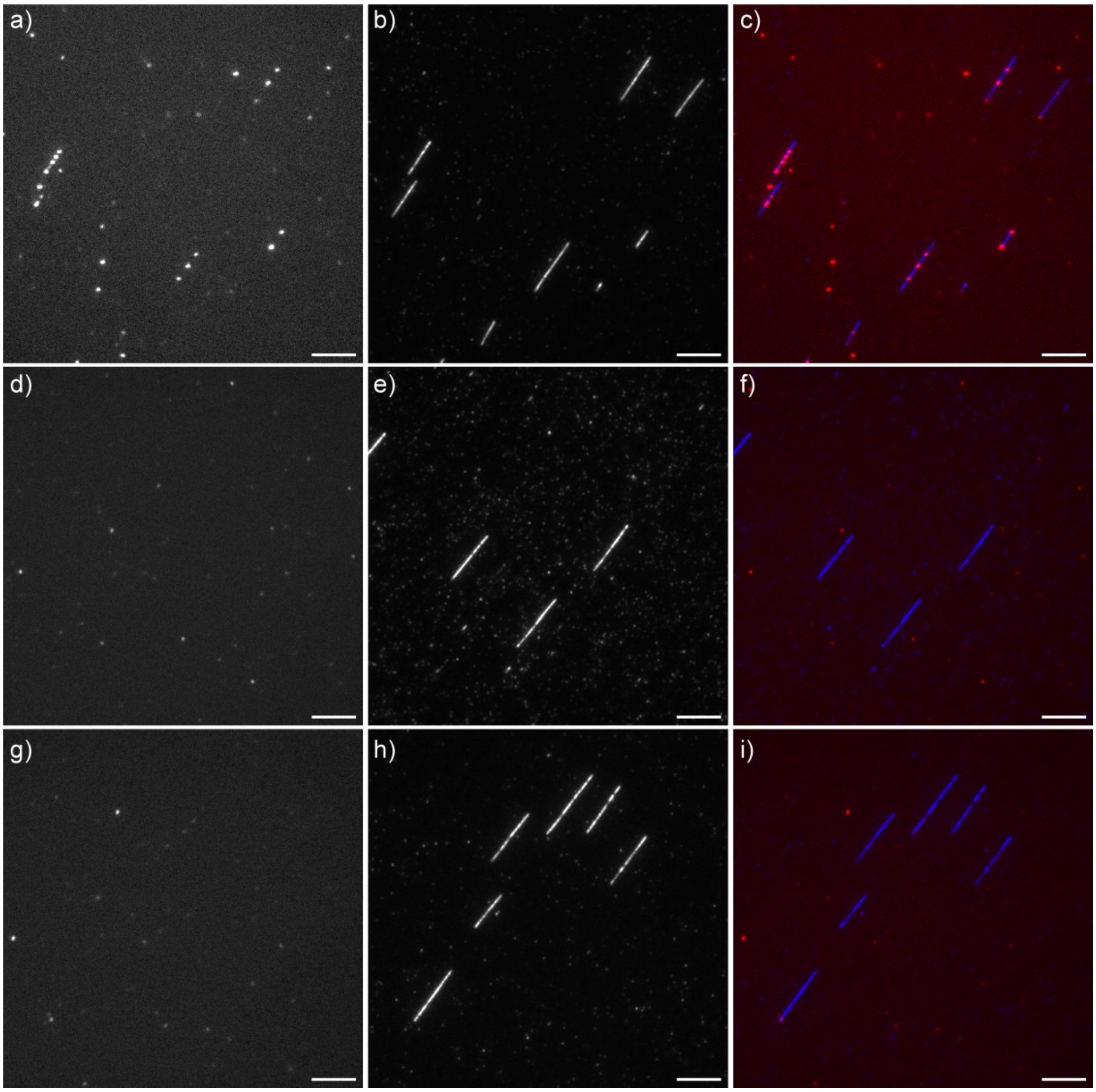

Figure S2. DNA imaging results of light-activated C-5′ azido proximity-labeling SAM analogues. (a) Imaging result of analogue **2b** with plasmid DNA. (b) YOYO-1 staining of the DNA sample from **2b**. (c) Merged figure of (a) and (b). (d) Imaging result of analogue **2b** without using M.*Taq*I (**negative control**). (e) YOYO-1 staining of the DNA sample from the negative control. (f) Merged figure of (d) and (e). (g) Imaging result of analogue **2a** with plasmid DNA. (h) YOYO-1 staining of the DNA sample from **2a**. (i) Merged figure of (g) and (h). For DNA labeling, 5 µM of **2a** and **2b** were used, and DBCO-Atto647N was coupled by SPAAC reaction after activating reactive groups. For all samples, the labeled DNA was further stained with YOYO-1 to visualize the backbone. For all images, a widefield microscope was used. Laser channels: 488 nm (b, e, h), or 638 nm (a, d, g). Scale bars: 5 µm.

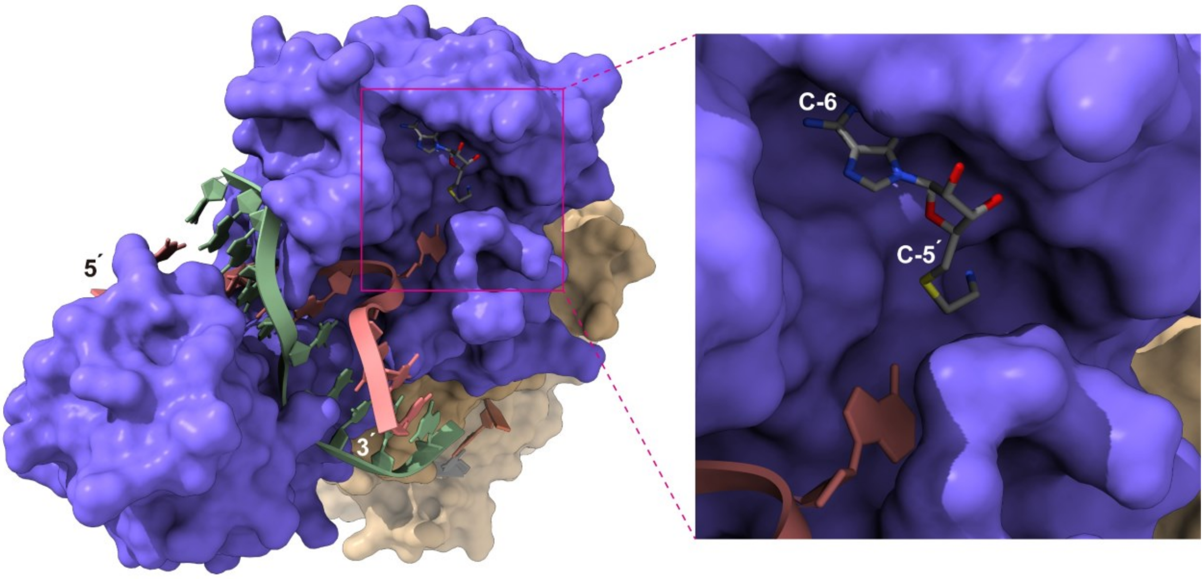

Figure S3. The ternary complex structure of M.*Taq*I, a SAM analogue 5′-[2-(amino)ethylthio]-5′-deoxyadenosine (AETA) and the double-stranded DNA with a target site 5′-GT<u>**TCGA**</u>TGTC-3′. In the left figure, the 5′ and 3′ directions of the labeled DNA strand are shown. The figure on the right represents the zoomed region of the complex. The C-6 and C-5′ positions are indicated in white text. PDB: 1G38.

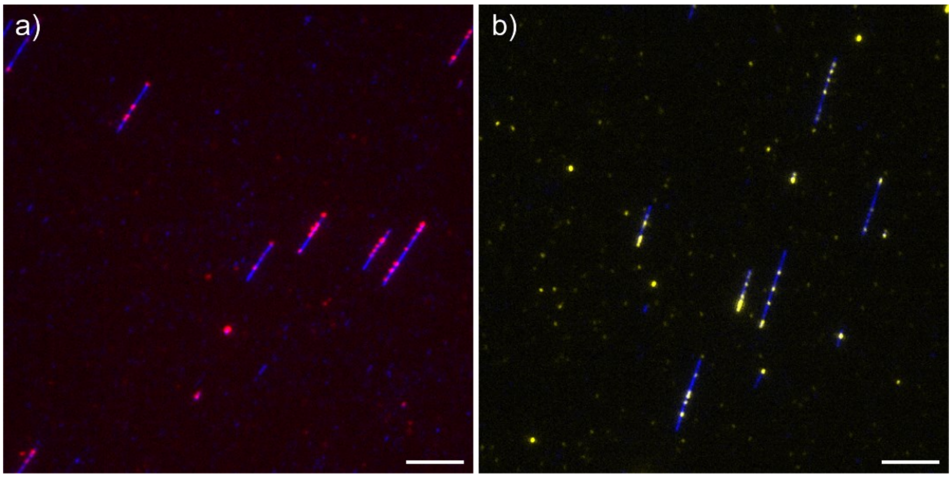

Figure S4. Imaging results of designed plasmid DNA using proximity-labeling SAM analogues **2c** and **3a**. (a) Imaging result of M.*Taq*I labeled plasmid DNA using **2c**. (b) Imaging result of M.*Taq*I labeled plasmid DNA using **3a**. For **2c** labeling, DBCO-Atto647N was used to introduce the fluorophore via SPAAC reaction. For all samples, the labeled DNA was further stained with YOYO-1 to visualize the backbone. Red color: Atto647N. Yellow color: rhodamine B. Blue color: YOYO-1. Scale bars: 5 μm.

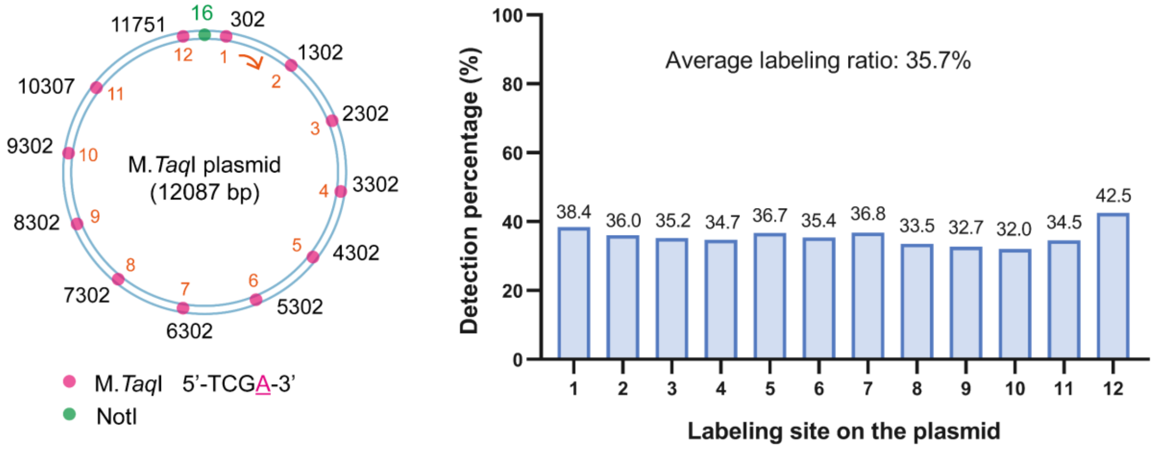

Figure S5. Fluorescence counting assay of **2d** labeled M.*Taq*I plasmid DNA. Labeling sites distribution on M.*Taq*I plasmid DNA (left) and fluorescence counting results of **2d** (right). For the fluorophore, DBCO-Atto647N was used to introduce the fluorescent signals via SPAAC reaction. 802 traces with full plasmid DNA length were analyzed and the quantified ratio for each recognition site was described by percentage on top of the column. The average labeling ratio is 35.7%. Taking the complete labeling of AdoYnAtto647N as a reference (average labeling ratio 50.8% in the counting assay, **Figure S7**), the apparent labeling efficiency of **2d** is 70.3%.

a)

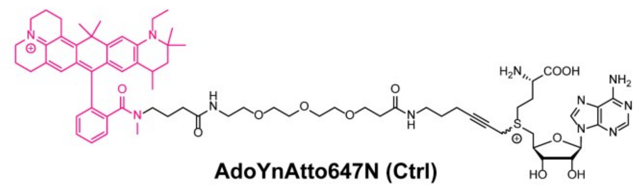

b)

Lane	1	2	3	4	5	6	7	8	9	10
pUC19	+	+	+	+	+	+	+	+	+	+
M.*Taq*I	-	-	+	+	+	+	+	+	+	+
*Taq*I-v2	-	+	+	+	+	+	+	+	+	+
SAM	-	-	-	50	-	-	-	-	-	-
Ctrl	-	30	-	-	2.5	5	10	15	20	30

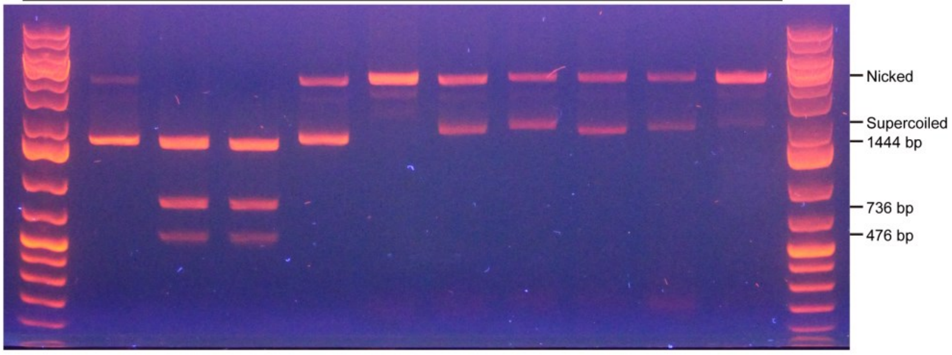

c)

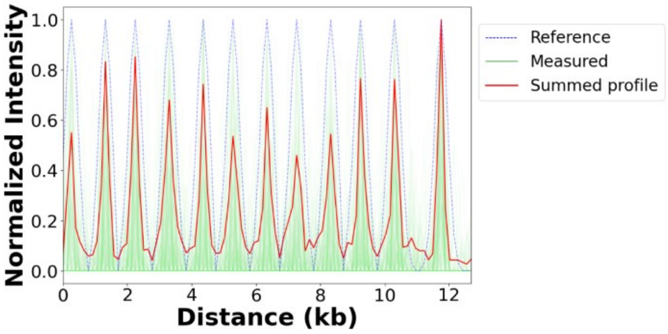

Figure S6. Validation of **AdoYnAtto647N** using gel-based restriction enzyme assay. (a) The chemical structure of a fluorescent double-activated SAM analogue **AdoYnAtto647N** (control). (b) Agarose gel restriction assay. The pUC19 DNA was visualized with GelRed staining in the agarose gel. For each reaction sample, the components are specified in the table above. Lane 2 and lane 3 were designed as controls, either without M.*Taq*I (lane 2) or without cofactor (lane 3). For SAM, 50 µM was used for MTase-directed labeling (lane 4). For **AdoYnAtto647N** (**Ctrl**), 2.5-30 µM were used for MTase-directed labeling (lane 5-10). The different forms of pUC19 (nicked and supercoiled) and the length of restricted fragments (1444, 736 and 476 bp) are specified. (c) Consensus map generated by imaging data of **AdoYnAtto647N** labeling. 183 traces with full plasmid DNA length were analyzed.

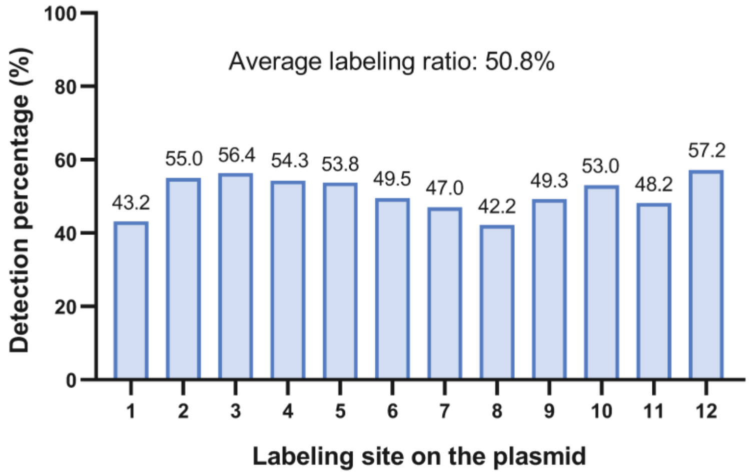

Figure S7. Fluorescence counting assay of **AdoYnAtto647N** labeled M.*Taq*I plasmid DNA. 183 traces with full plasmid DNA length were analyzed and the quantified ratio for each recognition site was described by percentage on top of the column. The average labeling ratio is 50.8% under conditions of complete labeling in the restriction enzyme assay.

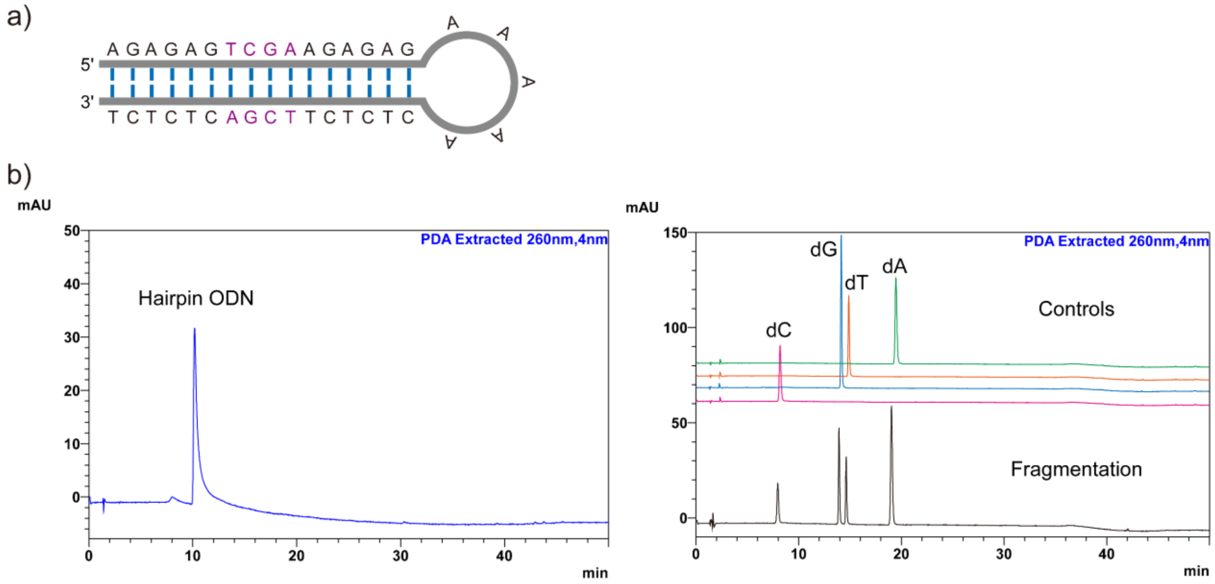

Figure S8. The hairpin ODN design and enzymatic fragmentation assay. (a) The designed hairpin ODN contains a 5′-TCGA-3′ labeling site and a 5-A loop, with 6 base pairs on both sides of the M.*Taq*I recognition site. (b) HPLC analysis of pure hairpin ODN (left) and enzymatic fragmentation analysis of the designed hairpin ODN (right). Deoxycytidine (dC), deoxyguanosine (dG), deoxythymidine (dT) and deoxyadenosine (dA) were used as controls. UV absorption at 260 nm was detected for the hairpin ODN and nucleosides.

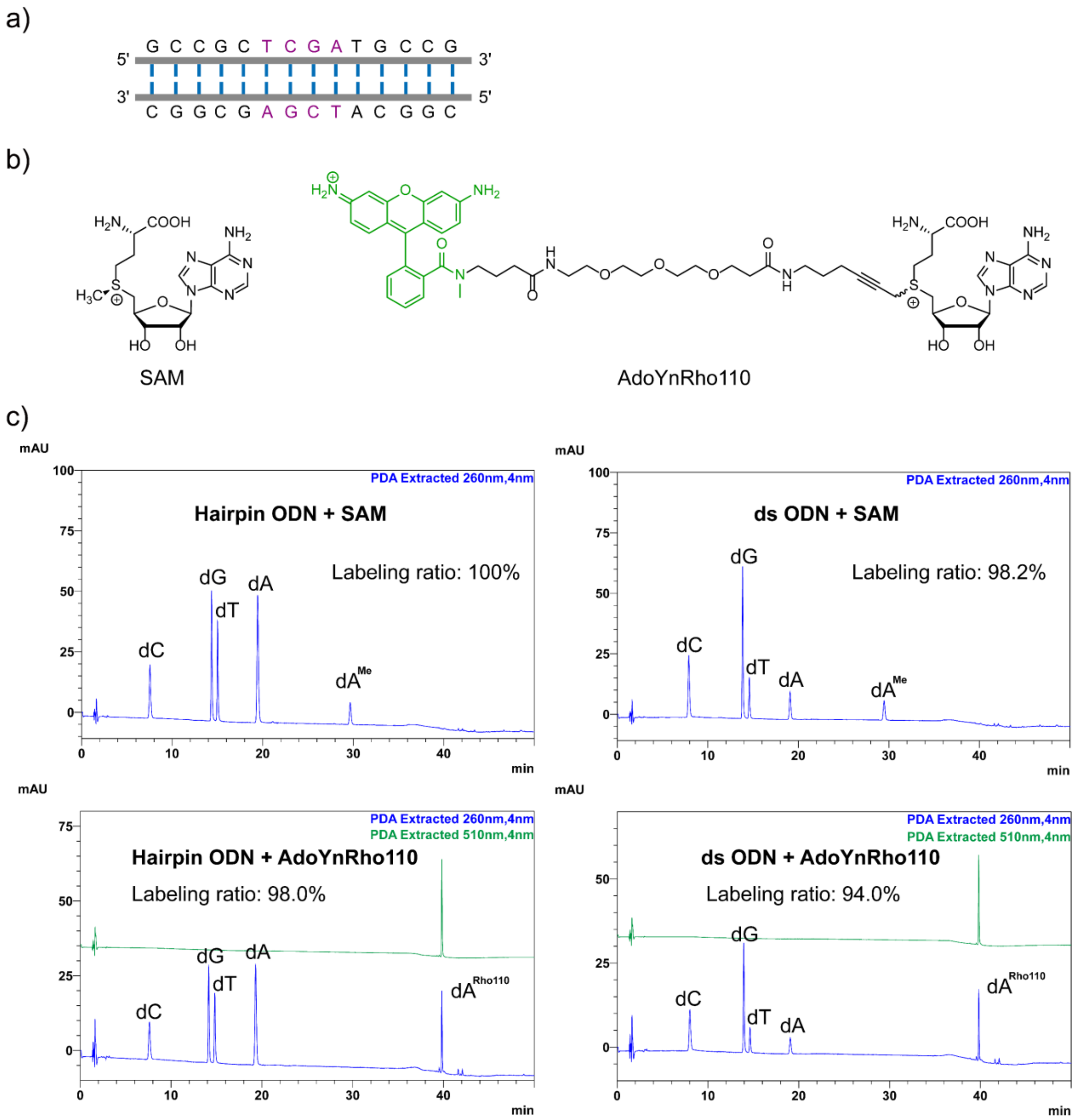

Figure S9. Validation of the hairpin ODN design through MTase-directed DNA labeling and fragmentation analysis. (a) The structure of the double-stranded (ds) ODN used in earlier studies.[4] (b) The chemical structures of cofactors SAM and AdoYnRho110. For AdoYnRho110, a rhodamine 110 (Rho110) dye is coupled and is indicated in green. (c) Enzymatic fragmentation analysis using both hairpin ODN and ds ODN. M.*Taq*I and cofactors SAM/AdoYnRho110 (20 µM) were used for DNA labeling. The HPLC spectrum at 510 nm is indicated in green.

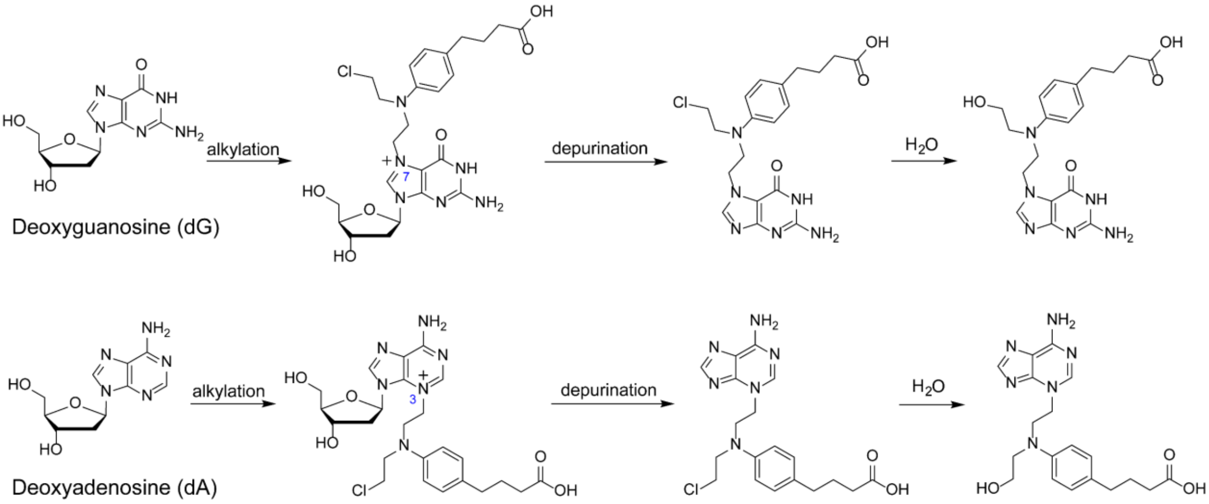

Figure S10. Reported decomposition pathways of chlorambucil-modified deoxyguanosine (dG) and deoxyadenosine (dA).[6]

a)

Lane	1	2	3	4	5	6
pUC19	+	+	+	+	+	+
M.*Taq*I	-	-	+	+	+	+
*Taq*I-v2	-	+	+	+	+	+
SAM	-	-	-	5	-	-
**2d**	-	10	-	-	5	10

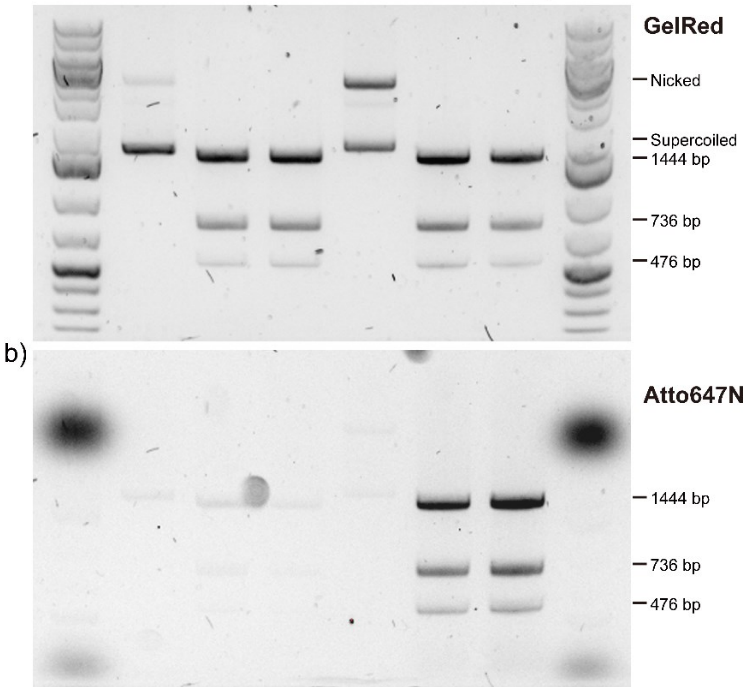

Figure S11. Gel-based restriction enzyme assay with in-gel fluorescence scanning. (a) Agarose gel with GelRed staining. For each reaction sample, the components are specified in the table above. Lane 2 and lane 3 were designed as controls, either without M.*Taq*I (lane 2) or without cofactor (lane 3). For SAM, 5 µM was used for MTase-directed labeling (lane 4). For **2d**, 5 or 10 µM were used for MTase-directed proximity labeling (lane 5 and lane 6). (b) In-gel fluorescence scanning. The fluorescent dye was introduced via SPAAC reaction with DBCO-Atto647N, and DBCO-Atto647N was added to all the samples to maintain consistency.

For the gel, different forms of pUC19 (nicked and supercoiled) and the length of restricted fragments (1444, 736 and 476 bp) are specified.

a)

Lane	1	2	3	4	5	6	7
pUC19	+	+	-	-	-	-	-
^{Me}pUC19	-	-	+	+	+	+	+
M.*Taq*I	-	-	-	-	+	+	+
*Taq*I-v2	-	+	+	+	+	+	+
**2d**	-	-	-	10	-	5	10
**Ctrl**	-	-	-	-	5	-	-

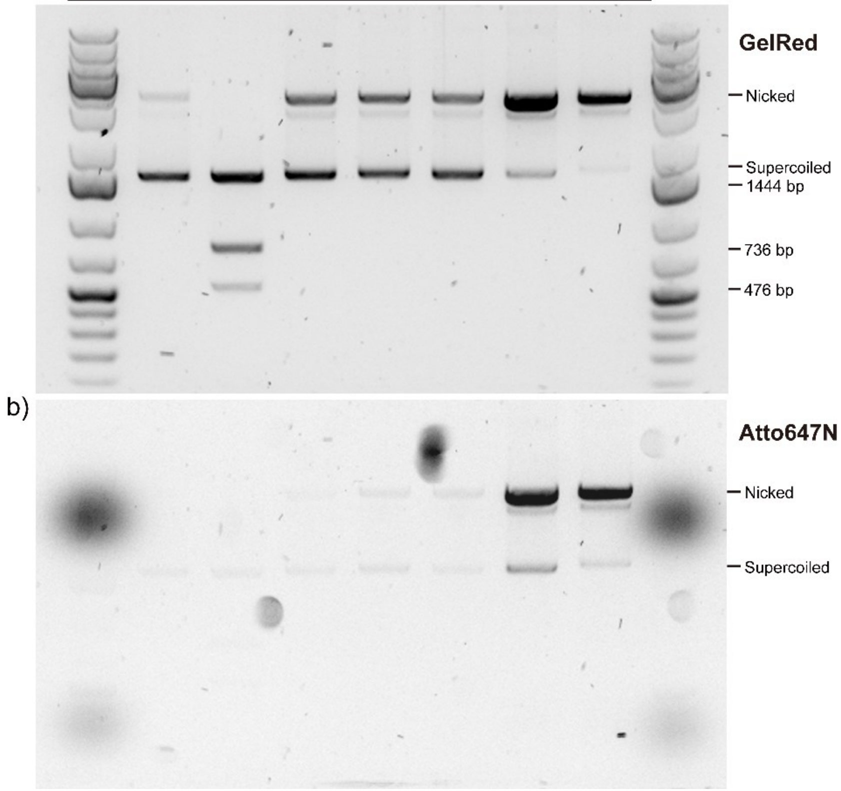

Figure S12. Validation of methylation-independent labeling of DNA using the proximity-labeling SAM analogue. (a) Agarose gel with GelRed staining. For each reaction sample, the components are specified in the table above. Lane 2 was designed as a control to show the restriction patterns of pUC19 DNA. Lane 3 was designed to confirm the methylation of M.*Taq*I sites in pUC19 DNA. Lane 4 was designed as a control to check nonspecific labeling. (b) In-gel fluorescence scanning. **AdoYnAtto647N** (**Ctrl**) was used as a control (lane 5), and 5 or 10 µM of **2d** were used to label the methylated pUC19 DNA (^{Me}pUC19, lane 6 and lane 7). The fluorescent dye was introduced via SPAAC reaction with DBCO-Atto647N, and DBCO-Atto647N was added to all the samples to maintain consistency. For the gel, the different forms of pUC19 (nicked and supercoiled) and the length of restricted fragments (1444, 736 and 476 bp) are specified.

3 Synthesis and Characterization

3.1 General Materials

Commercial chemicals were purchased from ACROS, BLD Pharm or Sigma-Aldrich, and were used without further purification unless otherwise indicated. All reactions were monitored by analytical thin layer chromatography (TLC). TLC was performed on pre-coated silica gel plates (60 F-254, 0.25 mm, Merck KGaA) with detection by UV (λ = 254 or 365 nm). NMR spectra (^{1}H-NMR, ^{13}C-NMR) were recorded at room temperature on a Bruker Avance III 400 MHz or a Bruker Avance II+ 600 MHz instrument. Chemical shifts were recorded in ppm relative to tetramethylsilane (TMS, internal standard) or residual solvent peaks ($CDCl_3$ = 7.26 ppm, MeOD-d_4 = 3.33 ppm, DMSO-d_6 = 2.50 ppm for ^{1}H NMR; $CDCl_3$ =77.06 ppm, MeOD-d_4 = 49.0 ppm, DMSO-d_6 = 39.53 ppm for ^{13}C NMR) with peaks being reported as follows: chemical shift, multiplicity (s = singlet, d = doublet, t = triplet, q = quartet, m = multiplet), coupling constant (Hz). Reversed-phase HPLC preparation was performed by Shimadzu HPLC with a Shim-pack GIST C18 column (5 μm, 100 x 6.0 mm). Mass spectra were obtained on a Shimadzu LCMS-2020 by electrospray ionization (ESI).

3.2 Synthetic Procedures

3.2.1 Synthesis of C-6 azido proximity-labeling SAM analogues

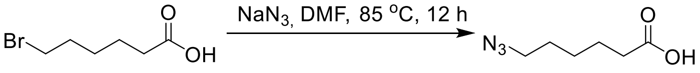

S1

Synthesis of **S1**

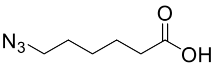

(**S1**)

To a solution of 6-bromo hexanoic acid (6.12 g, 31.4 mmol) in DMF (40 mL) was added sodium azide (6.12 g, 94.1 mmol). Then the mixture was stirred at 85 °C for 12 h. After completion of the reaction, the suspension was diluted with CH_2Cl_2 (400 mL) and washed with 0.1N HCl (400 mL). The organic layer was dried over anhydrous Na_2SO_4, filtered and concentrated to give product **S1** as a light yellow oil (4.44 g, 90% yield), without any further purification. ^{1}H NMR (400 MHz, $CDCl_3$) δ 3.26 (t, J = 6.9 Hz, 2H), 2.35 (t, J = 7.4 Hz, 2H), 1.71 – 1.55 (m, 4H), 1.47 – 1.36 (m, 2H). ^{13}C NMR (101 MHz, $CDCl_3$) δ 178.31, 51.22, 31.59, 28.55, 26.18, 24.25. ESI-MS (m/z): [M+H$^+$] calcd. for $C_6H_{12}N_3O_2^+$ 158.09; found, 158.00. [M-H$^+$] calcd. for $C_6H_{10}N_3O_2^-$ 156.08; found, 156.00.

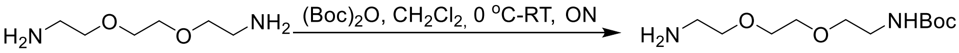

S2

Synthesis of **S2**

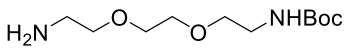
(**S2**)

To a solution of 2,2′-(ethylenedioxy)bis(ethylamine) (13.34 g, 90 mmol) in CH_2Cl_2 (80 mL) at 0 °C was added dropwise di-*tert*-butyl dicarbonate (3.27 g, 15 mmol) in CH_2Cl_2 (25 mL) over 20 min. The resulting mixture was stirred at 0 °C for 30 min and then warmed to room temperature to stir overnight. After completion of the reaction, the solvent was removed by evaporation. The residue was dissolved in H_2O (30 mL) and extracted with CH_2Cl_2 (3 x 40 mL). The combined organic layer was washed with brine (50 mL), dried over anhydrous Na_2SO_4, filtered and concentrated to give product **S2** as a light yellow oil (3.27 g, 88% yield), without any further purification. ^{1}H NMR (400 MHz, $CDCl_3$) δ 3.62 – 3.57 (m, 4H), 3.55 – 3.45 (m, 4H), 3.29 (d, J = 5.0 Hz, 2H), 2.84 (td, J = 5.2, 2.7 Hz, 2H), 1.41 (s, 9H). ^{13}C NMR (101 MHz, $CDCl_3$) δ 156.00, 79.13, 73.47, 70.28, 70.20, 41.78, 41.75, 40.33, 28.41. ESI-MS (m/z): [M+H$^+$] calcd. for $C_{11}H_{25}N_2O_4^+$ 249.18; found, 249.05.

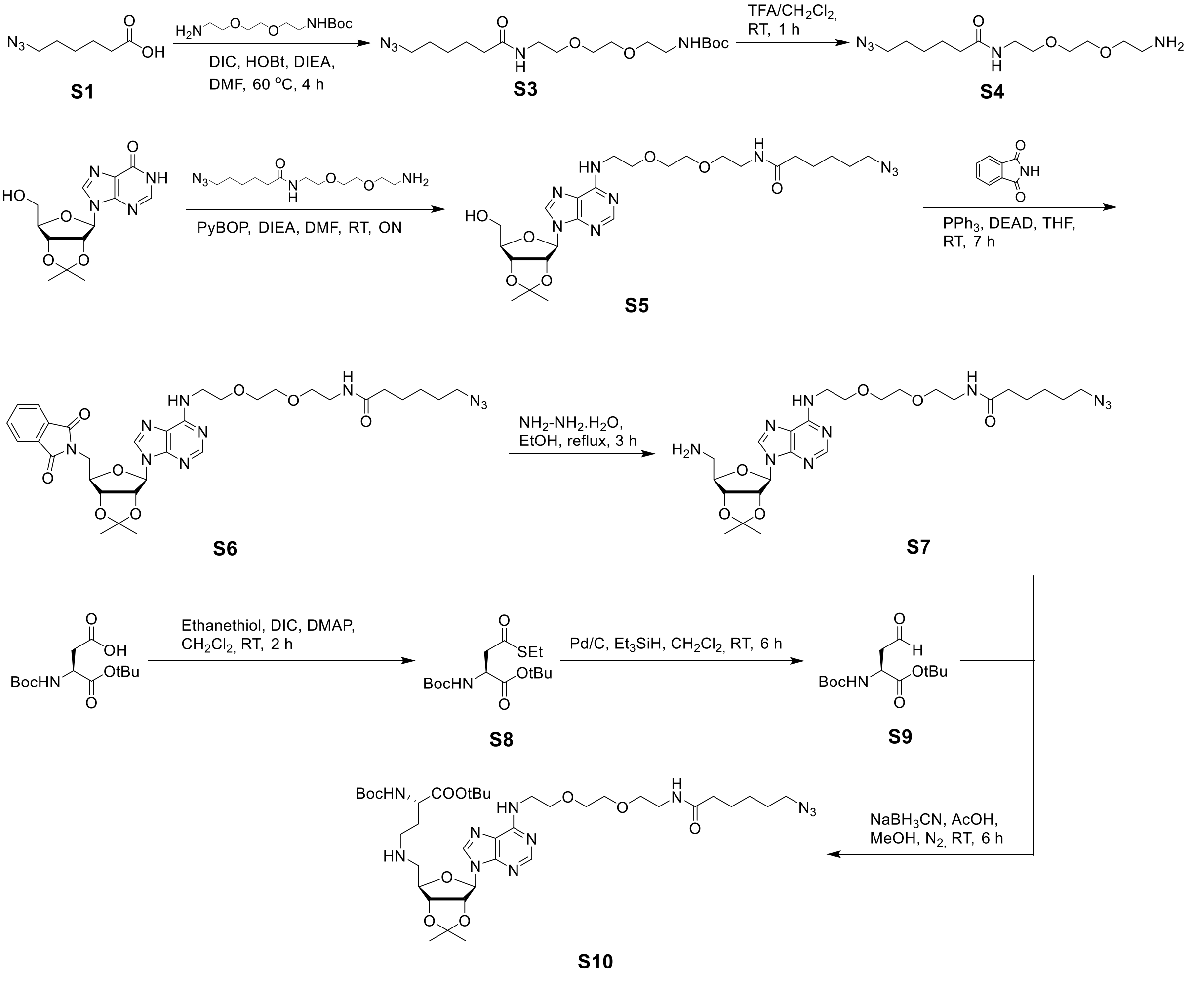

Synthesis of **S3**

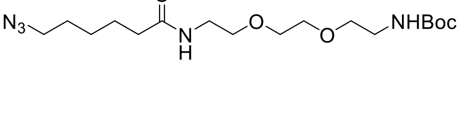
(**S3**)

To a solution of **S1** (1.57 g, 10 mmol) and **S2** (2.73 g, 11 mmol) in DMF (15 mL) were added HOBt (1.48 g, 11 mmol) and DIEA (3.48 mL, 20 mmol). Then the mixture was warmed to 60 °C. Under that temperature, DIC (3.08 mL, 20 mmol) was added and the resulting solution was stirred for 4 h. After completion of the reaction, the suspension was allowed to cool to room temperature and then filtered. The filtrate was slowly added dropwise to a solution of saturated $NaHCO_{3(aq)}/H_2O$ (1:4, 200 mL). The resulting yellow solution was extracted with CH_2Cl_2 (4 x 50 mL). The combined organic layer was washed with water (100 mL) and brine (100 mL), dried over anhydrous Na_2SO_4, filtered and concentrated. The residue was purified by column chromatography on silica-gel (CH_2Cl_2: MeOH = 40:1) to give product **S3** as a yellow oil (2.79 g, 72% yield). ^{1}H NMR (400 MHz, $CDCl_3$) δ 6.08 (s, 1H), 5.00 (s, 1H), 3.57 (s, 4H), 3.52 (t, *J* = 5.1 Hz, 4H), 3.42 (dd, *J* = 10.3, 5.3 Hz, 2H), 3.35 – 3.18 (m, 4H), 2.17 (t, *J* = 7.5 Hz, 2H), 1.61 (ddt, *J* = 21.7, 14.7, 7.3 Hz, 4H), 1.47 – 1.29 (m, 11H). ^{13}C NMR (101 MHz, $CDCl_3$) δ 172.73, 155.96, 79.33, 70.25, 70.17, 69.92, 51.24, 40.31, 39.14, 36.32, 28.60, 28.39, 26.35, 25.12, 23.51. ESI-MS (m/z): [M+H$^+$] calcd. for $C_{17}H_{34}N_5O_5^+$ 388.26; found, 388.15.

Synthesis of **S4**

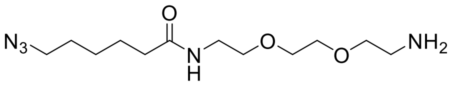

(**S4**)

S3 (2.7 g, 6.97 mmol) was dissolved in TFA/CH_2Cl_2 (3 mL/3 mL) and the mixture was stirred at room temperature for 1 h. Upon completion, the solvent was removed by evaporation and the product was further dried by vacuum. The resulting amine **S4** (2.0 g, 100% yield) can be directly used for next step.

Synthesis of **S5**

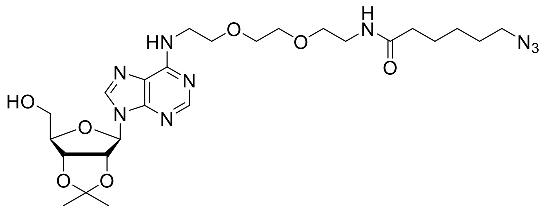

(**S5**)

To a solution of 2',3'-isopropylideneinosine (1.9 g, 6.18 mmol) in DMF (20 mL) were added PyBOP (3.54 g, 6.8 mmol) and DIEA (3.22 mL, 18.5 mmol). After 15 min, **S4** (1.78 g, 6.18 mmol) in DMF (4 mL) was added dropwise to the reaction mixture, followed by stirring at room temperature overnight. After completion of the reaction, H_2O (200 mL) was added and the mixture was extracted with EtOAc (3 x 80 mL). The combined organic layer was washed with 0.1N HCl (100 mL) and brine (100 mL), dried over anhydrous Na_2SO_4, filtered and concentrated. The residue was purified by column chromatography on silica-gel (CH_2Cl_2: MeOH = 30: 1) to give product **S5** as a light yellow solid (2.82 g, 79% yield). ^{1}H NMR (400 MHz, MeOD-*d₄*) δ 8.31 (s, 1H), 8.26 (s, 1H), 6.16 (d, *J* = 3.6 Hz, 1H), 5.28 (dd, *J* = 6.1, 3.7 Hz, 1H), 5.06 (dd, *J* = 6.1, 2.2 Hz, 1H), 4.40 (dd, *J* = 5.8, 3.5 Hz, 1H), 3.89 – 3.61 (m, 10H), 3.55 (t, *J* = 5.4 Hz, 2H), 3.39 – 3.34 (m, 2H), 3.27 (t, *J* = 6.8 Hz, 2H), 2.19 (t, *J* = 7.5 Hz, 2H), 1.64 (s, 3H), 1.58 (ddd, *J* = 17.2, 11.3, 5.2 Hz, 4H), 1.40 (s, 3H), 1.39 – 1.32 (m, 2H). ^{13}C NMR (101

MHz, MeOD-*d₄*) δ 174.60, 154.86, 152.38, 139.92, 119.74, 113.84, 91.51, 86.59, 83.89, 81.61, 70.00, 69.92, 69.22, 62.23, 50.89, 46.01, 45.96, 38.97, 35.41, 28.20, 26.27, 26.02, 25.94, 25.08, 24.19. ESI-MS (m/z): [M+H$^+$] calcd. for $C_{25}H_{40}N_9O_7^+$ 578.30; found, 578.45.

Synthesis of **S6**

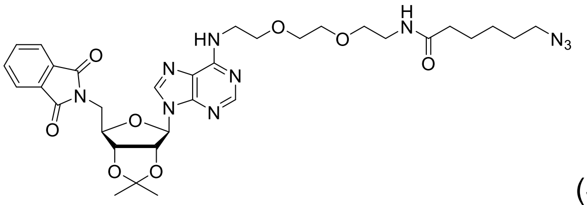

(**S6**)

To a solution of **S5** (2.5 g, 4.32 mmol) in anhydrous THF (20 mL) were added phthalimide (0.64 g, 4.32 mmol) and triphenylphosphine (1.17 g, 4.45 mmol). Then diethyl azodicarboxylate (1.96 mL, 2.2 mol/L, 4.32 mmol) was added dropwise to the mixture with the generation of heat. The reaction mixture was stirred at room temperature for 7 h. After completion of the reaction, the mixture was filtered and the filtrate was concentrated and dried by evaporation. The residue was purified by column chromatography on silica-gel (CH_2Cl_2: MeOH = 40: 1) to give product **S6** as a light yellow solid (2.29 g, 75% yield). ^{1}H NMR (400 MHz, MeOD-d_4) δ 8.17 (s, 1H), 8.01 (s, 1H), 7.78 – 7.69 (m, 4H), 6.17 (d, *J* = 1.7 Hz, 1H), 5.54 (dd, *J* = 6.2, 1.7 Hz, 1H), 5.27 (dd, *J* = 6.2, 3.6 Hz, 1H), 4.49 (td, *J* = 6.0, 3.7 Hz, 1H), 3.96 (d, *J* = 6.3 Hz, 2H), 3.75 (s, 4H), 3.72 – 3.68 (m, 2H), 3.67 – 3.62 (m, 2H), 3.56 (t, *J* = 5.4 Hz, 2H), 3.37 (t, *J* = 5.4 Hz, 2H), 3.25 (t, *J* = 6.8 Hz, 2H), 2.19 (t, *J* = 7.5 Hz, 2H), 1.65 – 1.51 (m, 7H), 1.39 – 1.30 (m, 5H). ^{13}C NMR (101 MHz, MeOD-d_4) δ 174.61, 168.12, 154.58, 152.47, 140.48, 133.98, 131.69, 122.72, 119.63, 114.07, 90.02, 84.92, 83.96, 82.33, 70.01, 69.93, 69.24, 50.88, 39.21, 38.99, 35.43, 28.20, 26.07, 25.94, 25.08, 24.21. ESI-MS (m/z): [M+H$^+$] calcd. for $C_{33}H_{43}N_{10}O_8^+$ 707.33; found, 707.20.

Synthesis of **S7**

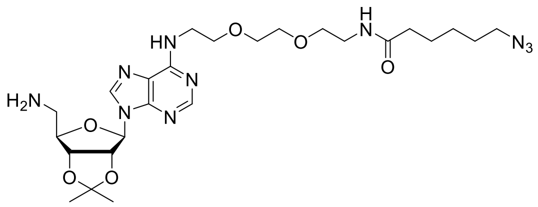

(**S7**)

To a solution of **S6** (2.2 g, 3.11 mmol) in EtOH (25 mL) was added hydrazine monohydrate (3.17 mL, 49.8 mmol). Then the reaction mixture was refluxed for 3 h. After completion of the reaction, the suspension was cooled down to room temperature and filtered. The filtrate was concentrated and dried by evaporation. The residue was dissolved in H_2O (40 mL) and extracted with $CHCl_3$ (3 x 40 mL). The combined organic layer was washed with brine (50 mL), dried over anhydrous Na_2SO_4, filtered and concentrated to give product **S7** as a light yellow solid (1.62 g, 90% yield), without any further purification. ^{1}H NMR (600 MHz, MeOD-d_4) δ 8.30 (s, 1H), 8.25 (s, 1H), 6.23 (d, *J* = 2.7 Hz, 1H), 5.46 (dd, *J* = 6.3, 2.7 Hz, 1H), 5.12 (dd, *J* = 6.3, 3.5 Hz, 1H), 4.39 (dt, *J* = 7.6, 3.7 Hz, 1H), 3.81 (s, 2H), 3.76 (t, *J* = 5.0 Hz, 2H), 3.69 (dd, *J* = 5.8, 3.1 Hz, 2H), 3.64 (dd, *J* = 5.8, 3.0 Hz, 2H), 3.55 (t, *J* = 5.5 Hz, 2H), 3.35 (t, *J* = 5.5 Hz,

2H), 3.26 (dt, J = 13.5, 7.3 Hz, 3H), 3.17 (dd, J = 13.4, 3.9 Hz, 1H), 2.20 (t, J = 7.5 Hz, 2H), 1.67 – 1.63 (m, 3H), 1.63 – 1.54 (m, 4H), 1.41 (s, 3H), 1.39 – 1.33 (m, 2H). ^{13}C NMR (151 MHz, MeOD-d_4) δ 174.64, 168.87, 154.87, 152.62, 140.29, 119.89, 114.49, 90.59, 84.86, 83.76, 81.83, 70.01, 69.91, 69.20, 50.88, 42.10, 38.94, 35.40, 28.19, 26.06, 25.93, 25.07, 24.13. ESI-MS (m/z): [M+H$^+$] calcd. for $C_{25}H_{41}N_{10}O_6^+$ 577.32; found, 577.20.

Synthesis of **S8**

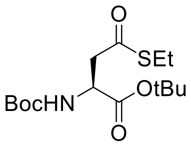

(**S8**)

To a solution of Boc-Asp-OtBu (3.0 g, 10.4 mmol) in anhydrous CH_2Cl_2 (20 mL) were added DIC (4.81 mL, 31.1 mmol), DMAP (1.39 g, 11.4 mmol) and ethanethiol (2.3 mL, 31.1 mmol). Then the reaction mixture was stirred at room temperature for 2 h. After completion of the reaction, the mixture was filtered over celite and the solvent was removed by evaporation. The residue was dissolved in a small amount of CH_2Cl_2 and stored in the freezer. The resulting suspension was filtered and the filtrate was purified by column chromatography on silica-gel (Heptane: EtOAc = 6:1) to give product **S8** as a light yellow oil (3.17 g, 92% yield). ^{1}H NMR (400 MHz, $CDCl_3$) δ 5.40 (d, J = 7.8 Hz, 1H), 4.50 – 4.34 (m, 1H), 3.08 (ddd, J = 47.5, 16.3, 4.6 Hz, 2H), 2.95 – 2.81 (m, 2H), 1.44 (s, 9H), 1.43 (s, 9H), 1.24 (t, J = 7.4 Hz, 3H). ^{13}C NMR (101 MHz, $CDCl_3$) δ 197.02, 169.74, 155.32, 82.39, 79.84, 50.94, 45.60, 28.31, 27.88, 23.47, 14.67. ESI-MS (m/z): [M+H$^+$] calcd. for $C_{15}H_{28}NO_5S^+$ 334.17; found, 334.00.

Synthesis of **S9**

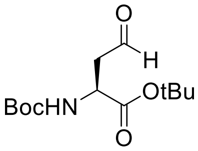

(**S9**)

To a solution of **S9** (1.5 g, 4.5 mmol) in anhydrous CH_2Cl_2 (5 mL) were added Pd/C (0.064 g) and triethylsilane (2 mL, 12.5 mmol). Then the reaction mixture was stirred at room temperature for 6 h. After completion of the reaction, the mixture was filtered over celite, followed by washing with some CH_2Cl_2. The filtrate was concentrated and dried by evaporation. The obtained crude was purified by column chromatography on silica-gel (Heptane: EtOAc = 4:1) to give product **S9** as a light yellow solid (1.02 g, 83% yield). ^{1}H NMR (400 MHz, $CDCl_3$) δ 9.72 (s, 1H), 5.35 (d, J = 6.9 Hz, 1H), 4.47 (dd, J = 12.6, 5.0 Hz, 1H), 2.96 (qd, J = 17.9, 4.9 Hz, 2H), 1.44 (s, 9H), 1.43 (s, 9H). ^{13}C NMR (101 MHz, $CDCl_3$) δ 199.41, 169.92, 155.36, 82.69, 80.02, 49.30, 46.38, 28.29, 27.86. ESI-MS (m/z): [M+Na$^+$] calcd. for $C_{13}H_{23}NNaO_5^+$ 296.15; found, 296.00.

Synthesis of **S10**

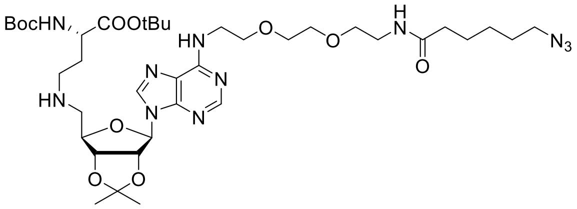

(**S10**)

To a solution of **S7** (1.12 g, 1.94 mmol) in anhydrous MeOH (15 mL) were added **S9** (0.53 g, 1.94 mmol), sodium cyanoborohydride (0.24 g, 3.88 mmol) and acetic acid (108 μL, 1.94 mmol). Then the reaction mixture is stirred at room temperature under N_2 atmosphere for 6 h. After completion of the reaction, the solvent was removed by evaporation and the residue was purified by column chromatography on silica-gel (CH_2Cl_2: MeOH = 20:1) to give product **S10** as a white foam (0.91 g, 56% yield). ^{1}H NMR (400 MHz, MeOD-*d₄*) δ 8.30 (s, 1H), 8.26 (s, 1H), 6.14 (d, *J* = 3.2 Hz, 1H), 5.49 (dd, *J* = 6.4, 3.3 Hz, 1H), 5.06 (dd, *J* = 6.4, 3.3 Hz, 1H), 4.39 – 4.31 (m, 1H), 4.08 (dd, *J* = 8.4, 4.8 Hz, 1H), 3.90 – 3.74 (m, 4H), 3.72 – 3.62 (m, 5H), 3.56 (t, *J* = 5.4 Hz, 2H), 3.36 (t, *J* = 5.4 Hz, 2H), 3.27 (t, *J* = 6.8 Hz, 2H), 2.89 (ddd, *J* = 19.3, 12.6, 5.5 Hz, 2H), 2.74 – 2.58 (m, 2H), 2.20 (t, *J* = 7.4 Hz, 2H), 1.93 (dt, *J* = 7.5, 5.4 Hz, 1H), 1.78 (td, *J* = 13.5, 7.7 Hz, 1H), 1.68 – 1.52 (m, 8H), 1.46 (s, 9H), 1.42 – 1.38 (m, 13H). ^{13}C NMR (101 MHz, MeOD-*d₄*) δ 174.63, 172.00, 156.58, 154.82, 152.64, 140.15, 119.77, 114.31, 90.39, 85.06, 83.29, 82.37, 81.25, 79.03, 70.00, 69.91, 69.21, 52.88, 51.00, 50.88, 45.81, 38.96, 35.41, 30.96, 28.19, 27.31, 26.87, 26.16, 25.93, 25.07, 24.27. ESI-MS (m/z): [M+H$^+$] calcd. for $C_{38}H_{64}N_{11}O_{10}^+$ 834.48; found, 834.35.

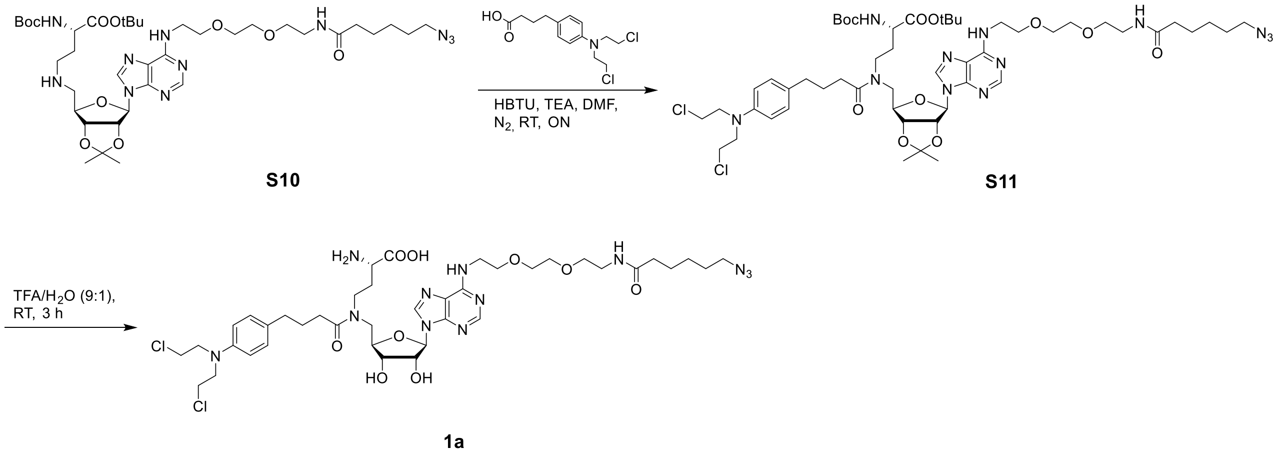

Synthesis of **S11**

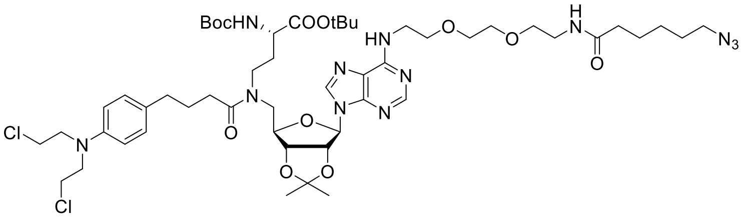

(**S11**)

To a solution of chlorambucil (22 mg, 0.072 mmol) in DMF (3 mL) were added HBTU (32.6 mg, 0.086 mmol) and TEA (30.6 μL, 0.216 mmol). After stirring for 15 min, **S10** (60 mg, 0.072 mmol) was added and the reaction mixture was stirred at room temperature under N_2

atmosphere overnight. After completion of the reaction, the solvent was removed by evaporation and the residue was purified by column chromatography on silica-gel (CH_2Cl_2: MeOH = 20:1) to give product **S11** as a white solid (40.3 mg, 50% yield). ^{1}H NMR (600 MHz, MeOD-*d₄*) δ 8.32 (s, 1H), 8.24 (d, *J* = 21.0 Hz, 1H), 7.06 (d, *J* = 8.6 Hz, 1H), 6.91 (d, *J* = 8.5 Hz, 1H), 6.67 (t, *J* = 8.7 Hz, 2H), 6.22 (dd, *J* = 36.0, 1.8 Hz, 1H), 5.49 (dd, *J* = 23.1, 4.6 Hz, 1H), 5.15 – 5.06 (m, 1H), 4.53 – 4.34 (m, 1H), 3.99 – 3.93 (m, 1H), 3.78 – 3.64 (m, 14H), 3.62 – 3.52 (m, 6H), 3.39 – 3.34 (m, 3H), 3.27 (t, *J* = 6.8 Hz, 2H), 3.23 – 3.08 (m, 1H), 2.55 (t, *J* = 7.5 Hz, 1H), 2.40 – 2.27 (m, 1H), 2.22 – 2.17 (m, 2H), 2.12 – 2.00 (m, 2H), 1.89 (tdd, *J* = 31.6, 15.8, 8.0 Hz, 2H), 1.63 – 1.56 (m, 8H), 1.48 – 1.38 (m, 26H). ESI-MS (m/z): [M+H$^+$] calcd. for $C_{52}H_{81}Cl_2N_{12}O_{11}^+$ 1119.55; found, 1119.40.

Synthesis of **1a**

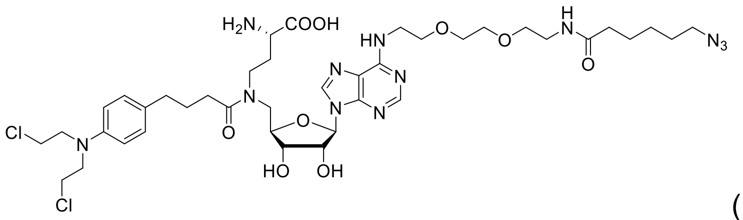

(**1a**)

S11 (20 mg, 0.0178 mmol) was dissolved in a mixture of TFA/H_2O (0.9 mL/0.1 mL). The reaction mixture was stirred at room temperature for 3 h. After completion of the reaction, the solvent was removed by evaporation. EtOH was added to co-evaporate for 3 times, followed by vacuum drying to give product **1a** as a white solid (16.5 mg, 100% yield), without further purification. ESI-MS (m/z): [M+H$^+$] calcd. for $C_{40}H_{61}Cl_2N_{12}O_9^+$ 923.41; found, 923.25.

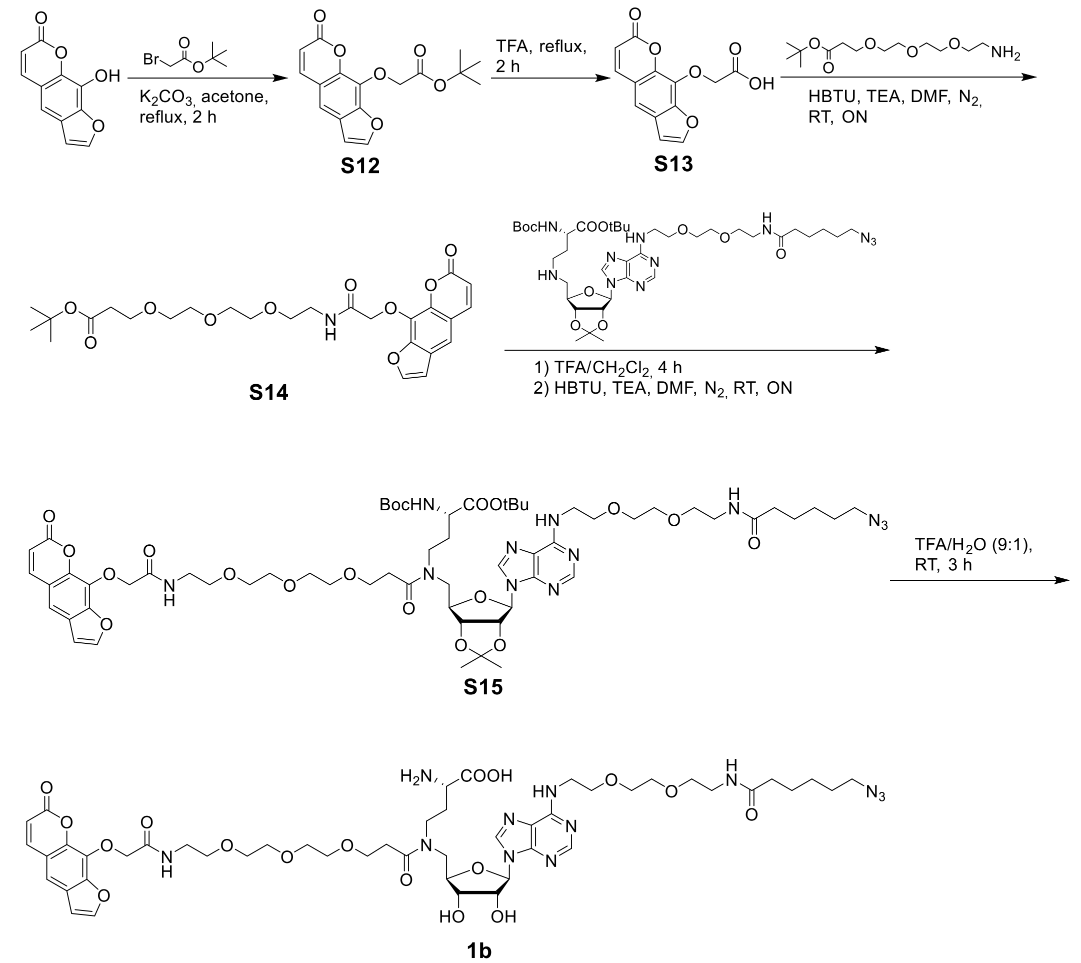

1b

Synthesis of **S12**

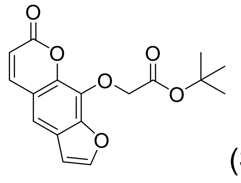

(**S12**)

To a solution of 8-hydroxypsoralen (1.0 g, 4.92 mmol) in anhydrous acetone (100 mL) were added *tert*-butyl bromoacetate (3.84 g, 19.8 mmol) and K_2CO_3 (5.01 g, 36.2 mmol). Then the reaction mixture was refluxed for 2 h. After completion of the reaction, the suspension was filtered and the filtrate was concentrated and dried by evaporation. The obtained crude was purified by column chromatography on silica-gel (Heptane: EtOAc = 3:1) to give product **S12** as a white solid (1.15 g, 74% yield). ^{1}H NMR (400 MHz, $CDCl_3$) δ 7.73 (d, *J* = 9.6 Hz, 1H), 7.63 (d, *J* = 2.2 Hz, 1H), 7.31 (s, 1H), 6.78 (d, *J* = 2.2 Hz, 1H), 6.31 (d, *J* = 9.6 Hz, 1H), 5.03 (s, 2H), 1.40 (s, 9H). ^{13}C NMR (101 MHz, $CDCl_3$) δ 167.85, 160.17, 146.74, 146.54, 144.35, 142.25, 131.01, 126.03, 116.46, 114.68, 112.97, 106.78, 82.26, 69.15, 27.98. ESI-MS (m/z): [M+Na$^+$] calcd. for $C_{17}H_{16}NaO_6^+$ 339.08; found, 338.95.

Synthesis of **S13**

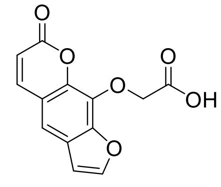

(**S13**)

S12 (1.01 g, 3.21 mmol) was dissolved in TFA (6 mL) and the mixture was refluxed for 2 h. After completion of the reaction, the solvent was removed by evaporation. The residue was co-evaporated with acetonitrile for twice to give product **S13** as a white solid (0.83 g, 100% yield). ^{1}H NMR (400 MHz, DMSO-d_6) δ 13.10 (s, 1H), 8.12 (dd, J = 9.7, 2.1 Hz, 1H), 8.10 – 8.08 (m, 1H), 7.61 (d, J = 2.3 Hz, 1H), 7.07 (t, J = 1.9 Hz, 1H), 6.43 (dd, J = 9.6, 1.1 Hz, 1H), 5.14 (s, 2H). ^{13}C NMR (101 MHz, DMSO-d_6) δ 170.41, 160.13, 148.20, 146.17, 145.64, 141.89, 130.81, 126.36, 116.79, 114.67, 113.71, 107.40, 68.60. ESI-MS (m/z): [M-H$^+$] calcd. for $C_{13}H_7O_6^-$ 259.02; found, 258.90.

Synthesis of **S14**

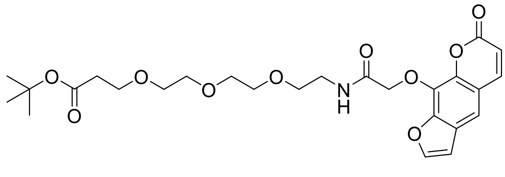

(**S14**)

To a solution of **S13** (222 mg, 0.85 mmol) in DMF (3 mL) were added HBTU (388 mg, 1.02 mmol) and TEA (357 µL, 2.56 mmol). After stirring for 15 min, amino-PEG3-t-butyl ester (260 mg, 0.94 mmol) was added and the reaction mixture was stirred at room temperature under N_2 atmosphere overnight. After completion of the reaction, the solvent was removed by evaporation and the residue was purified by column chromatography on silica-gel (CH_2Cl_2: MeOH = 40:1) to give product **S14** as a light yellow oil (385 mg, 87% yield). ^{1}H NMR (400 MHz, $CDCl_3$) δ 7.77 (d, J = 9.6 Hz, 1H), 7.71 (d, J = 2.2 Hz, 1H), 7.51 (s, 1H), 7.42 (s, 1H), 6.83 (d, J = 2.2 Hz, 1H), 6.36 (d, J = 9.6 Hz, 1H), 4.92 (s, 2H), 3.66 – 3.54 (m, 14H), 2.46 (t, J = 6.6 Hz, 2H), 1.41 (s, 9H). ^{13}C NMR (101 MHz, $CDCl_3$) δ 170.88, 168.20, 159.77, 147.35, 147.06, 144.17, 142.93, 130.75, 126.17, 116.47, 114.96, 114.34, 106.94, 80.48, 72.28, 70.54, 70.47, 70.37, 70.32, 69.56, 66.85, 38.96, 36.23, 28.08. ESI-MS (m/z): [M+H$^+$] calcd. for $C_{26}H_{34}NO_{10}^+$ 520.22; found, 520.10.

Synthesis of **S15**

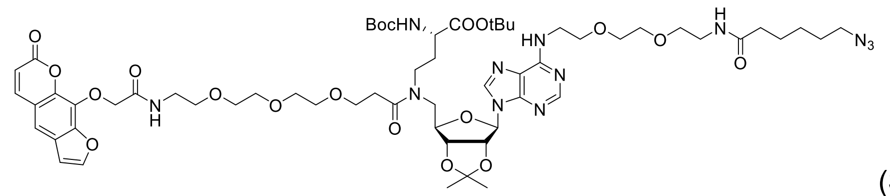

(**S15**)

S14 (37.5 mg, 0.072 mmol) was dissolved in TFA/CH_2Cl_2 (0.8 mL/0.2 mL) and the mixture was stirred at room temperature for 4 h. Upon completion, the solvent was removed by evaporation and the product was further dried by vacuum. The resulting acid can be directly used for next step. Next, to a solution of the acid obtained above (33.4 mg, 0.072 mmol) in

DMF (2 mL) were added HBTU (32.6 mg, 0.086 mmol) and TEA (30.6 μL, 0.216 mmol). After stirring for 15 min, **S10** (60 mg, 0.072 mmol) was added and the reaction mixture was stirred at room temperature under N_2 atmosphere overnight. After completion of the reaction, the solvent was removed by evaporation and the residue was purified by column chromatography on silica-gel (CH_2Cl_2: MeOH = 15:1) to give product **S15** as a light yellow solid (53.4 mg, 58% yield). ^{1}H NMR (600 MHz, MeOD-*d₄*) δ 8.30 (s, 1H), 8.22 (d, *J* = 22.7 Hz, 1H), 8.03 (dd, *J* = 9.6, 3.0 Hz, 1H), 7.92 (t, *J* = 2.1 Hz, 1H), 7.62 (d, *J* = 2.9 Hz, 1H), 6.99 (dd, *J* = 2.2, 1.3 Hz, 1H), 6.41 (dd, *J* = 9.6, 1.3 Hz, 1H), 6.18 (dd, *J* = 22.3, 2.2 Hz, 1H), 5.48 (dd, *J* = 6.3, 2.0 Hz, 1H), 5.13 – 5.03 (m, 1H), 4.96 – 4.92 (m, 2H), 4.48 – 4.36 (m, 1H), 3.93 – 3.87 (m, 1H), 3.82 – 3.73 (m, 4H), 3.72 – 3.63 (m, 10H), 3.62 – 3.52 (m, 10H), 3.38 – 3.35 (m, 3H), 3.27 (t, *J* = 6.8 Hz, 2H), 3.22 (q, *J* = 7.3 Hz, 1H), 2.67 – 2.53 (m, 1H), 2.20 (t, *J* = 7.5 Hz, 2H), 2.05 – 1.82 (m, 1H), 1.69 – 1.52 (m, 8H), 1.47 – 1.32 (m, 26H). ^{13}C NMR (151 MHz, MeOD-*d₄*) δ 174.62, 172.73, 172.69, 169.49, 160.63, 160.60, 147.44, 147.20, 145.07, 145.05, 142.66, 130.41, 130.38, 126.52, 126.50, 116.63, 114.58, 114.49, 114.21, 113.93, 106.74, 84.00, 82.45, 82.12, 81.45, 81.29, 71.59, 70.15, 70.12, 70.05, 70.03, 69.99, 69.95, 69.92, 69.22, 69.13, 69.10, 67.06, 66.85, 50.88, 38.96, 38.78, 35.41, 32.89, 28.71, 28.20, 27.35, 26.86, 26.10, 26.07, 25.94, 25.07, 24.18. ESI-MS (m/z): [M+H$^+$] calcd. for $C_{60}H_{87}N_{12}O_{19}^+$ 1279.62; found, 1279.50.

Synthesis of **1b**

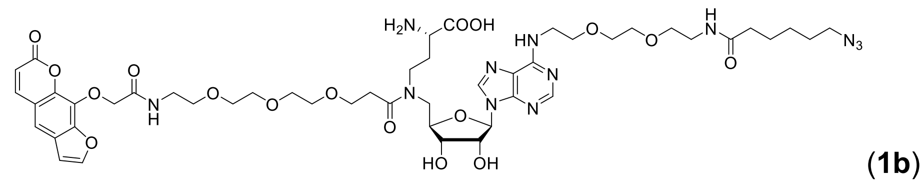

(**1b**)

S15 (20 mg, 0.0156 mmol) was dissolved in a mixture of TFA/H_2O (0.9 mL/0.1 mL). The reaction mixture was stirred at room temperature for 3 h. After completion of the reaction, the solvent was removed by evaporation. EtOH was added to co-evaporate for 3 times, followed by vacuum drying to give product **1b** as a light yellow solid (16.9 mg, 100% yield), without further purification. ESI-MS (m/z): [M+H$^+$] calcd. for $C_{48}H_{67}N_{12}O_{17}^+$ 1083.47; found, 1083.35.

3.2.2 Synthesis of C-5′ azido proximity-labeling SAM analogues

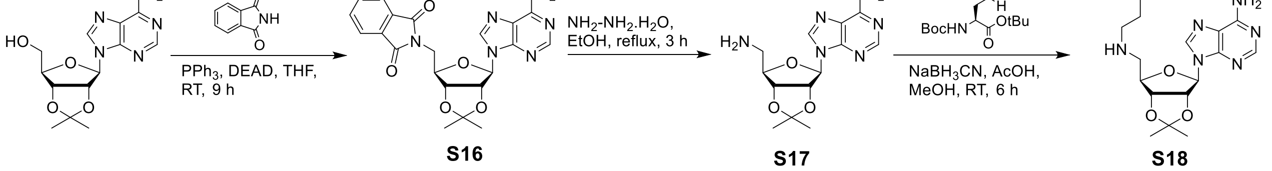

Synthesis of **S16**

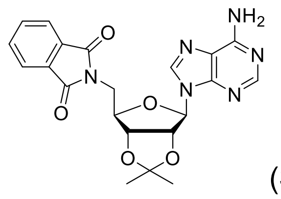

(**S16**)

To a solution of 2′,3′-*O*-isopropylideneadenosine (12.25 g, 40 mmol) in anhydrous THF (50 mL) were added phthalimide (5.9 g, 40 mmol) and triphenylphosphine (10.8 g, 41.2 mmol). Then diethyl azodicarboxylate (18.2 mL, 2.2 mol/L, 40 mmol) was added dropwise to the mixture with the generation of heat. The reaction mixture was stirred at room temperature for 9 h. After completion of the reaction, the mixture was filtered and the solid was washed with cold EtOAc (200 mL) and Et_2O (100 mL). After drying by vacuum, the product **S16** was obtained as a white solid (13.6 g, 78% yield). ^{1}H NMR (400 MHz, DMSO-d_6) δ 8.29 (s, 1H), 7.87 (s, 1H), 7.81 (s, 4H), 7.31 (s, 2H), 6.19 (d, *J* = 2.0 Hz, 1H), 5.45 (dd, *J* = 6.3, 2.0 Hz, 1H), 5.19 (dd, *J* = 6.3, 3.7 Hz, 1H), 4.38 (td, *J* = 6.0, 3.9 Hz, 1H), 3.96 – 3.85 (m, 2H), 1.51 (s, 3H), 1.31 (s, 3H). ^{13}C NMR (101 MHz, DMSO-d_6) δ 168.19, 156.51, 152.92, 149.09, 140.78, 134.90, 131.88, 123.52, 119.70, 113.94, 89.17, 84.22, 83.89, 82.22, 27.46, 25.71. ESI-MS (m/z): [M+H$^+$] calcd. for $C_{21}H_{21}N_6O_5^+$ 437.16; found, 437.25.

Synthesis of **S17**

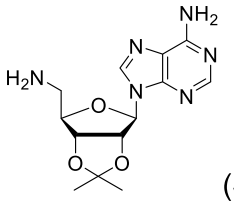

(**S17**)

To a solution of **S16** (2.18 g, 5 mmol) in EtOH (25 mL) was added hydrazine monohydrate (5.09 mL, 80 mmol). Then the reaction mixture was refluxed for 3 h. After completion of the reaction, the suspension was cooled down to room temperature and filtered. The filtrate was concentrated and dried by evaporation. The residue was resuspended in EtOAc and sonicated. The suspension was filtered, followed by washing with EtOAc. After drying by vacuum, the product **S17** was obtained as a white solid (1.32 g, 86% yield). ^{1}H NMR (400 MHz, MeOD-d_4) δ 8.29 (s, 1H), 8.22 (s, 1H), 6.15 (d, *J* = 3.1 Hz, 1H), 5.49 (dd, *J* = 6.4, 3.1 Hz, 1H), 5.02 (dd, *J* = 6.4, 3.4 Hz, 1H), 4.24 (td, *J* = 5.7, 3.5 Hz, 1H), 2.90 (dd, *J* = 5.7, 1.3 Hz, 2H), 1.61 (s, 3H), 1.39 (s, 3H). ^{13}C NMR (101 MHz, MeOD-d_4) δ 156.02, 152.57, 148.92, 140.53, 119.30, 114.26, 90.18, 87.13, 83.47, 81.86, 43.23, 26.11, 24.18. ESI-MS (m/z): [M+H$^+$] calcd. for $C_{13}H_{19}N_6O_3^+$ 307.15; found, 307.05.

Synthesis of **S18**

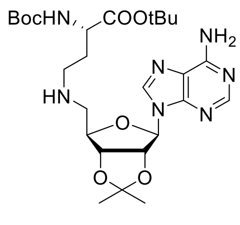

(**S18**)

To a solution of **S17** (0.92 g, 3 mmol) in anhydrous MeOH (20 mL) were added **S9** (0.82 g, 3 mmol), sodium cyanoborohydride (0.37 g, 6 mmol) and acetic acid (167 μL, 3 mmol). Then the reaction mixture is stirred at room temperature under N_2 atmosphere for 6 h. After completion of the reaction, the solvent was removed by evaporation and the residue was purified by column chromatography on silica-gel (CH_2Cl_2: MeOH = 25:1) to give product **S18** as a white foam (1.27 g, 75% yield). ^1H NMR (600 MHz, $CDCl_3$) δ 8.32 (s, 1H), 7.91 (s, 1H), 5.96 (d, *J* = 3.6 Hz, 2H), 5.91 (s, 2H), 5.52 – 5.45 (m, 1H), 5.07 (dd, *J* = 6.3, 3.0 Hz, 1H), 4.36 (d, *J* = 3.2 Hz, 1H), 4.28 (dd, *J* = 11.8, 7.4 Hz, 1H), 2.94 (dd, *J* = 12.5, 3.6 Hz, 1H), 2.81 – 2.71 (m, 2H), 2.62 (dd, *J* = 16.0, 10.2 Hz, 1H), 1.93 (d, *J* = 5.8 Hz, 1H), 1.82 (dd, *J* = 13.2, 6.7 Hz, 1H), 1.60 (s, 3H), 1.44 (s, 9H), 1.38 (d, *J* = 8.5 Hz, 12H). ^{13}C NMR (151 MHz, $CDCl_3$) δ 171.85, 155.78, 155.59, 153.08, 149.43, 139.92, 120.41, 114.58, 91.05, 85.05, 83.04, 82.17, 81.62, 79.37, 52.96, 51.38, 46.26, 32.32, 28.32, 28.02, 27.35, 25.45. ESI-MS (m/z): [M+H$^+$] calcd. for $C_{26}H_{42}N_7O_7^+$ 564.31; found, 564.20.

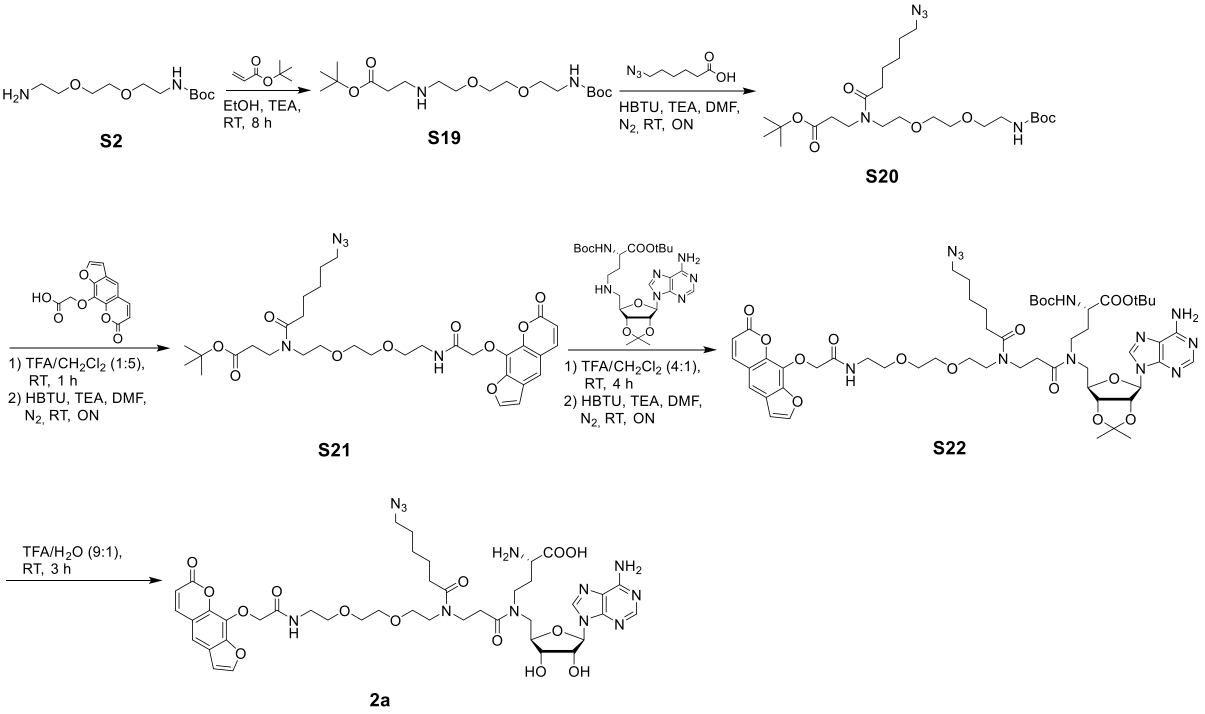

Synthesis of **S19**

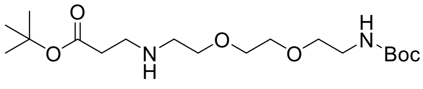
(**S19**)

To a solution of **S2** (4.0 g, 16.1 mmol) in EtOH (50 mL) was added *tert*-butyl acrylate (2.82 mL,19.3 mmol) and TEA (2.68 mL,19.3 mmol). Then the reaction mixture was stirred at room temperature for 8 h. After completion of the reaction, the solvent was removed by evaporation and the residue was purified by column chromatography on silica-gel (CH_2Cl_2: MeOH = 20:1) to give product **S19** as a light yellow oil (4.36 g, 72% yield). ^1H NMR (400 MHz, $CDCl_3$) δ 5.19 (s, 1H), 3.60 – 3.55 (m, 6H), 3.51 (t, *J* = 5.1 Hz, 2H), 3.29 (dd, *J* = 10.1, 5.0 Hz, 2H), 2.84 (t, *J* = 6.7 Hz, 2H), 2.78 (t, *J* = 5.3 Hz, 2H), 2.41 (t, *J* = 6.7 Hz, 2H), 1.83 (s, 1H), 1.42 (s, 18H). ^{13}C

NMR (101 MHz, $CDCl_3$) δ 172.02, 156.02, 80.43, 79.09, 70.62, 70.22, 49.08, 45.26, 40.36, 35.96, 28.42, 28.11. ESI-MS (m/z): [M+H$^+$] calcd. for $C_{18}H_{37}N_2O_6^+$ 377.26; found, 377.40.

Synthesis of **S20**

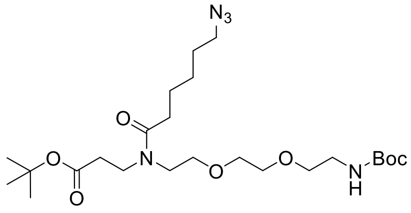

(**S20**)

To a solution of **S1** (1.0 g, 6.36 mmol) in DMF (10 mL) were added HBTU (2.88 g, 7.63 mmol) and TEA (2.71 mL, 19.1 mmol). After stirring for 15 min, **S19** (2.39 g, 6.36 mmol) was added and the reaction mixture was stirred at room temperature under N_2 atmosphere overnight. After completion of the reaction, H_2O (100 mL) was added and the mixture was extracted with EtOAc (3 x 50 mL). The combined organic layer was washed with H_2O (2 x 80 mL) and brine (80 mL), dried over anhydrous Na_2SO_4, filtered and concentrated. The residue was purified by column chromatography on silica-gel (Heptane: EtOAc = 1:1) to give product **S20** as a light yellow oil (2.95 g, 90% yield). ^{1}H NMR (400 MHz, $CDCl_3$) δ 5.02 (s, 1H), 3.60 – 3.48 (m, 10H), 3.26 (t, *J* = 6.9 Hz, 5H), 2.51 (t, *J* = 7.3 Hz, 2H), 2.33 (dt, *J* = 22.1, 7.3 Hz, 4H), 1.70 – 1.56 (m, 8H), 1.44 – 1.40 (m, 18H). ^{13}C NMR (101 MHz, $CDCl_3$) δ 173.04, 171.52, 155.96, 81.25, 80.59, 70.75, 70.34, 70.25, 69.42, 51.29, 45.96, 42.81, 38.60, 32.87, 32.76, 28.77, 28.42, 28.08, 26.50, 24.67. ESI-MS (m/z): [M+H$^+$] calcd. for $C_{24}H_{46}N_5O_7^+$ 516.34; found, 516.20.

Synthesis of **S21**

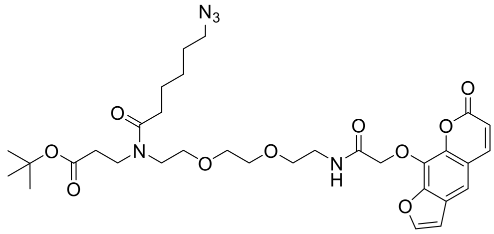

(**S21**)

S20 (200 mg, 0.388 mmol) was dissolved in TFA/CH_2Cl_2 (0.3 mL/1.5 mL) and the mixture was stirred at room temperature for 1 h. Upon completion, the solvent was removed by evaporation and the product was further dried by vacuum. The resulting amine can be directly used for next step. Next, to a solution of **S13** (101 mg, 0.388 mmol) in DMF (2 mL) were added HBTU (176 mg, 0.466 mmol) and TEA (161 μL, 1.16 mmol). After stirring for 15 min, the amine obtained from last step in DMF (1 mL) was added and the reaction mixture was stirred at room temperature under N_2 atmosphere overnight. After completion of the reaction, the solvent was removed by evaporation and the residue was purified by column chromatography on silica-gel (CH_2Cl_2: MeOH = 50:1) to give product **S21** as a light yellow oil (214 mg, 84% yield). ^{1}H NMR (400 MHz, MeOD-*d_4*) δ 8.07 (d, *J* = 9.7 Hz, 1H), 7.94 (d, *J* = 2.2 Hz, 1H), 7.67 (s, 1H), 7.02 (d, *J* = 2.2 Hz, 1H), 6.43 (d, *J* = 9.6 Hz, 1H), 4.97 (s, 2H), 3.69 – 3.53 (m, 14H), 3.28 (td, *J* = 6.8, 4.2 Hz, 2H), 2.58 (t, *J* = 7.1 Hz, 1H), 2.50 (t, *J* = 7.2 Hz, 1H), 2.41 (td, *J* = 7.5, 4.3 Hz, 2H), 1.65 – 1.54 (m, 4H), 1.43 (d, *J* = 0.7 Hz, 9H), 1.41 – 1.35 (m, 2H). ^{13}C NMR (101 MHz, MeOD-

d$_4$) δ 174.51, 171.59, 169.42, 160.65, 147.44, 147.26, 145.10, 142.72, 130.45, 126.58, 116.69, 114.64, 113.92, 106.76, 80.40, 71.59, 70.35, 70.08, 69.00, 68.71, 50.92, 42.55, 38.70, 34.31, 33.27, 32.46, 28.34, 26.92, 26.09, 24.52. ESI-MS (m/z): [M+H$^+$] calcd. for $C_{32}H_{44}N_5O_{10}^+$ 658.31; found, 658.20.

Synthesis of **S22**

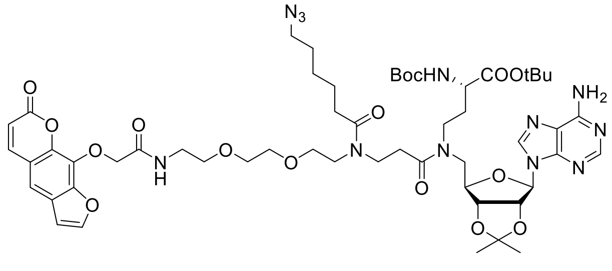

(**S22**)

S21 (56 mg, 0.085 mmol) was dissolved in TFA/CH_2Cl_2 (0.8 mL/0.2 mL) and the mixture was stirred at room temperature for 4 h. Upon completion, the solvent was removed by evaporation and the product was further dried by vacuum. The resulting acid can be directly used for next step. Next, to a solution of the acid obtained above (51 mg, 0.085 mmol) in DMF (2 mL) were added HBTU (37.8 mg, 0.1 mmol) and TEA (35.4 μL, 0.255 mmol). After stirring for 15 min, **S18** (48 mg, 0.085 mmol) was added and the reaction mixture was stirred at room temperature under N_2 atmosphere overnight. After completion of the reaction, the solvent was removed by evaporation and the residue was purified by column chromatography on silica-gel (CH_2Cl_2: MeOH = 30:1) to give product **S22** as a light yellow solid (58 mg, 59% yield). ^{1}H NMR (600 MHz, MeOD-*d*$_4$) δ 8.25 (ddd, *J* = 13.1, 11.5, 6.3 Hz, 2H), 8.07 – 8.00 (m, 1H), 7.92 (dd, *J* = 3.3, 2.4 Hz, 1H), 7.62 (dd, *J* = 4.4, 2.2 Hz, 1H), 6.99 (t, *J* = 2.7 Hz, 1H), 6.41 (dt, *J* = 9.6, 2.2 Hz, 1H), 6.18 (ddd, *J* = 31.6, 7.0, 2.1 Hz, 1H), 5.50 – 5.36 (m, 1H), 5.12 – 5.01 (m, 1H), 4.95 (d, *J* = 7.4 Hz, 2H), 4.47 – 4.31 (m, 1H), 3.99 – 3.88 (m, 1H), 3.69 – 3.38 (m, 16H), 3.29 – 3.25 (m, 2H), 2.79 – 2.16 (m, 4H), 2.06 – 1.84 (m, 1H), 1.76 (ddd, *J* = 17.8, 8.9, 4.5 Hz, 1H), 1.67 – 1.49 (m, 8H), 1.41 (ddd, *J* = 27.6, 13.6, 4.4 Hz, 24H). ^{13}C NMR (151 MHz, MeOD-*d*$_4$) δ 174.22, 173.78, 172.57, 169.43, 160.61, 156.00, 152.80, 147.41, 147.19, 145.05, 142.66, 140.73, 130.42, 126.53, 116.65, 114.66, 114.61, 114.59, 114.55, 114.32, 113.97, 113.95, 106.76, 89.77, 84.16, 83.63, 82.42, 81.99, 71.61, 70.28, 70.06, 69.98, 69.05, 50.97, 50.93, 50.26, 38.73, 32.47, 32.36, 32.23, 28.38, 28.31, 27.36, 26.87, 26.84, 26.16, 26.10, 24.44, 24.19. ESI-MS (m/z): [M+H$^+$] calcd. for $C_{54}H_{75}N_{12}O_{16}^+$ 1147.54; found, 1147.40.

Synthesis of **2a**

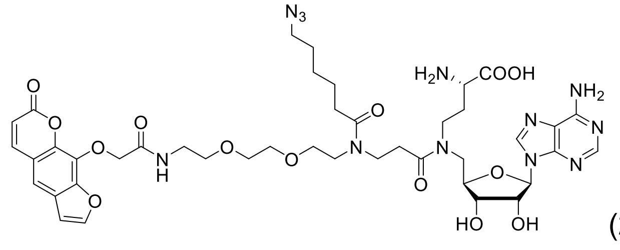

(**2a**)

S22 (20 mg, 0.0174 mmol) was dissolved in a mixture of TFA/H_2O (0.9 mL/0.1 mL). The reaction mixture was stirred at room temperature for 3 h. After completion of the reaction, the

solvent was removed by evaporation. EtOH was added to co-evaporate for 3 times, followed by vacuum drying to give product **2a** as a light yellow solid (16.6 mg, 100% yield). ^{1}H NMR (400 MHz, MeOD-d_4) δ 8.45 (t, *J* = 8.3 Hz, 1H), 8.40 (d, *J* = 1.3 Hz, 1H), 8.06 (d, *J* = 9.6 Hz, 1H), 7.93 (d, *J* = 2.2 Hz, 1H), 7.65 (d, *J* = 2.8 Hz, 1H), 7.01 (d, *J* = 2.2 Hz, 1H), 6.42 (d, *J* = 9.6 Hz, 1H), 6.06 (d, *J* = 3.2 Hz, 1H), 4.97 (s, 2H), 4.63 (dt, *J* = 7.9, 3.9 Hz, 1H), 4.44 (dd, *J* = 9.6, 4.6 Hz, 1H), 4.24 (dd, *J* = 11.3, 5.8 Hz, 1H), 4.02 – 3.92 (m, 1H), 3.85 (d, *J* = 5.9 Hz, 1H), 3.69 – 3.52 (m, 14H), 3.48 (t, *J* = 4.8 Hz, 2H), 3.27 (q, *J* = 6.9 Hz, 2H), 2.69 (dt, *J* = 15.7, 7.1 Hz, 2H), 2.42 – 2.10 (m, 4H), 1.64 – 1.48 (m, 4H), 1.42 – 1.31 (m, 4H). ^{13}C NMR (101 MHz, MeOD-d_4) δ 174.46, 174.25, 170.05, 169.50, 160.73, 151.20, 148.35, 147.44, 147.18, 145.15, 144.94, 142.87, 142.72, 142.63, 130.39, 126.60, 116.66, 114.66, 113.91, 106.78, 90.26, 82.58, 73.79, 71.56, 71.31, 70.32, 70.08, 69.01, 64.28, 50.95, 50.22, 49.82, 43.23, 42.05, 38.80, 38.72, 32.43, 31.16, 28.34, 28.29, 26.15, 24.47. ESI-MS (m/z): [M+H$^+$] calcd. for $C_{42}H_{55}N_{12}O_{14}^+$ 951.40; found, 951.25.

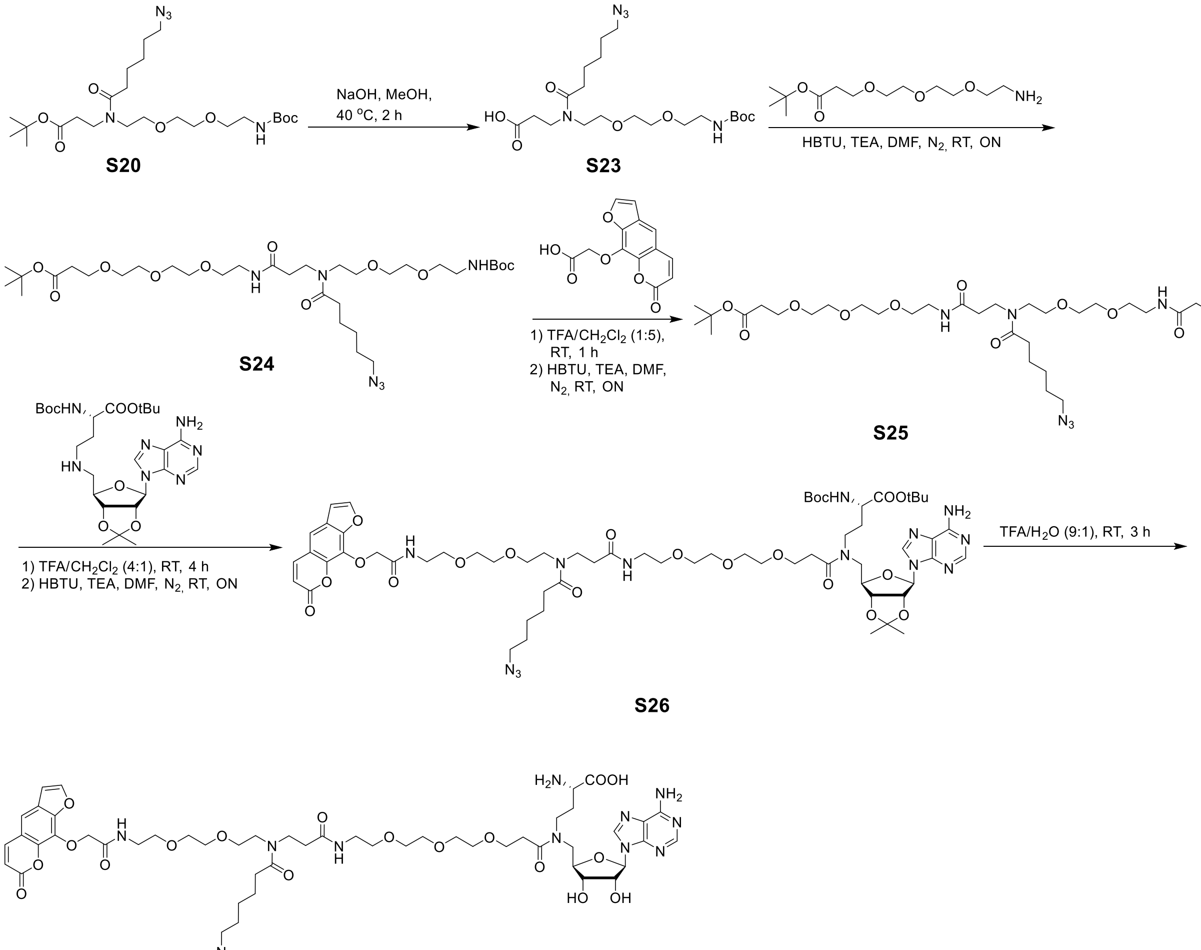

Synthesis of **S23**

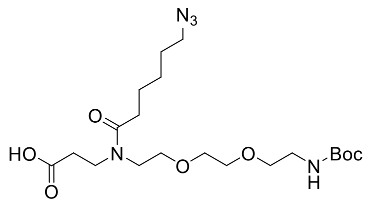

(**S23**)

To a solution of **S20** (1.03 g, 2 mmol) in MeOH (8 mL) was added 2N NaOH (8 mL). Then the reaction mixture was stirred at 40 °C for 2 h. After completion of the reaction, the mixture was acidified with 2N HCl to adjust pH to 3, followed by extraction with EtOAc (3 x 40 mL). The combined organic layer was washed with brine (60 mL), dried over anhydrous Na_2SO_4, filtered and concentrated to give product **S23** as a light yellow oil (0.87 g, 95% yield). ^{1}H NMR (400 MHz, $CDCl_3$) δ 5.05 (s, 1H), 3.77 – 3.56 (m, 8H), 3.55 – 3.46 (m, 4H), 3.27 (t, *J* = 6.9 Hz, 4H), 2.66 (s, 2H), 2.35 (td, *J* = 14.7, 7.4 Hz, 2H), 1.68 – 1.59 (m, 4H), 1.44 (s, 9H), 1.42 – 1.35 (m, 2H). ^{13}C NMR (101 MHz, $CDCl_3$) δ 173.88, 173.08, 156.16, 79.41, 70.69, 70.26, 69.44, 60.42, 51.28, 40.31, 38.63, 33.06, 32.93, 32.78, 28.73, 28.40, 26.46, 24.73. ESI-MS (m/z): [M-H$^+$] calcd. for $C_{20}H_{36}N_5O_7^-$ 458.26; found, 458.15.

Synthesis of **S24**

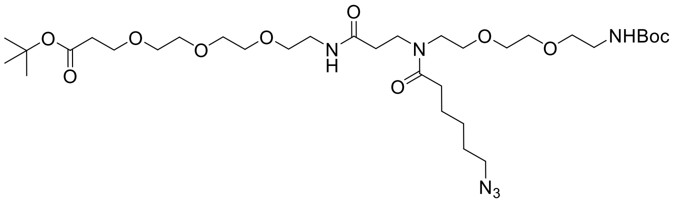

(**S24**)

To a solution of **S23** (891 mg, 1.93 mmol) in DMF (4 mL) were added HBTU (875 mg, 2.31 mmol) and TEA (805 µL, 5.79 mmol). After stirring for 15 min, amino-PEG3-t-butyl ester (538 mg, 1.93 mmol) was added and the reaction mixture was stirred at room temperature under N_2 atmosphere overnight. After completion of the reaction, H_2O (80 mL) was added and the mixture was extracted with EtOAc (3 x 40 mL). The combined organic layer was washed with H_2O (2 x 60 mL) and brine (60 mL), dried over anhydrous Na_2SO_4, filtered and concentrated. The residue was purified by column chromatography on silica-gel (CH_2Cl_2: MeOH = 40:1) to give product **S24** as a light yellow oil (1.11 g, 80% yield). ^{1}H NMR (400 MHz, $CDCl_3$) δ 6.92 (s, 1H), 5.02 (s, 1H), 3.73 – 3.48 (m, 24H), 3.44 – 3.37 (m, 2H), 3.26 (td, *J* = 6.9, 3.5 Hz, 4H), 2.56 – 2.50 (m, 2H), 2.49 – 2.39 (m, 2H), 2.36 (d, *J* = 9.6 Hz, 2H), 1.66 – 1.52 (m, 4H), 1.47 – 1.41 (m, 18H), 1.40 – 1.33 (m, 2H). ^{13}C NMR (101 MHz, $CDCl_3$) δ 174.14, 173.56, 172.09, 156.32, 81.46, 81.05, 70.56, 70.39, 70.16, 70.01, 69.88, 69.78, 69.66, 69.23, 66.76, 66.69, 51.30, 51.24, 43.10, 39.37, 39.25, 35.59, 32.89, 32.67, 28.68, 28.40, 28.01, 26.44, 24.72. ESI-MS (m/z): [M+H$^+$] calcd. for $C_{33}H_{63}N_6O_{11}^+$ 719.45; found, 719.35.

Synthesis of **S25**

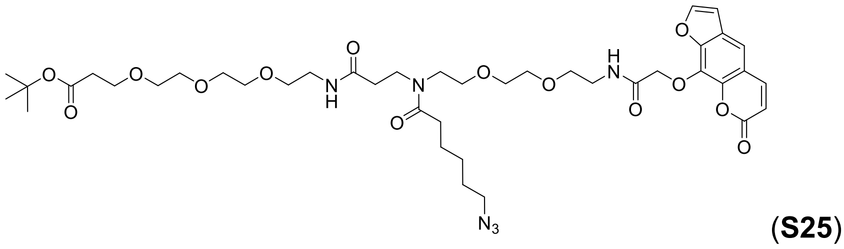

(**S25**)

S24 (173 mg, 0.24 mmol) was dissolved in TFA/CH_2Cl_2 (0.3 mL/1.5 mL) and the mixture was stirred at room temperature for 1 h. Upon completion, the solvent was removed by evaporation and the product was further dried by vacuum. The resulting amine can be directly used for next step. Next, to a solution of **S13** (62.5 mg, 0.24 mmol) in DMF (2 mL) were added HBTU (109 mg, 0.288 mmol) and TEA (100 μL, 0.72 mmol). After stirring for 15 min, the amine obtained from last step in DMF (1 mL) was added and the reaction mixture was stirred at room temperature under N_2 atmosphere overnight. After completion of the reaction, the solvent was removed by evaporation and the residue was purified by column chromatography on silica-gel (CH_2Cl_2: MeOH = 40:1) to give product **S25** as a light yellow oil (178 mg, 86% yield). ^{1}H NMR (600 MHz, $CDCl_3$) δ 7.79 (dd, *J* = 9.6, 2.5 Hz, 1H), 7.72 (dd, *J* = 3.5, 2.2 Hz, 1H), 7.44 (d, *J* = 3.3 Hz, 1H), 6.87 (d, *J* = 5.3 Hz, 1H), 6.85 (d, *J* = 2.2 Hz, 1H), 6.52 (t, *J* = 4.8 Hz, 1H), 6.38 (d, *J* = 9.6 Hz, 1H), 4.94 (d, *J* = 10.3 Hz, 2H), 3.70 – 3.57 (m, 22H), 3.49 (ddd, *J* = 12.6, 10.3, 5.0 Hz, 4H), 3.42 – 3.37 (m, 2H), 3.25 (dd, *J* = 6.9, 5.9 Hz, 2H), 2.53 – 2.43 (m, 4H), 2.36 (dt, *J* = 19.8, 7.5 Hz, 2H), 1.63 – 1.56 (m, 4H), 1.43 (d, *J* = 2.0 Hz, 9H), 1.37 (dd, *J* = 15.5, 7.7 Hz, 2H). ^{13}C NMR (151 MHz, $CDCl_3$) δ 173.44, 171.21, 170.91, 168.27, 159.85, 147.32, 147.08, 144.28, 142.87, 130.76, 126.24, 116.48, 114.95, 114.32, 106.99, 80.61, 80.56, 72.25, 70.74, 70.48, 70.35, 70.26, 70.17, 69.78, 69.64, 69.21, 66.89, 66.87, 51.32, 51.29, 48.37, 39.22, 38.93, 36.24, 35.06, 32.85, 28.77, 28.10, 26.50, 24.77. ESI-MS (m/z): [M+H$^+$] calcd. for $C_{41}H_{61}N_6O_{14}^+$ 861.42; found, 861.25.

Synthesis of **S26**

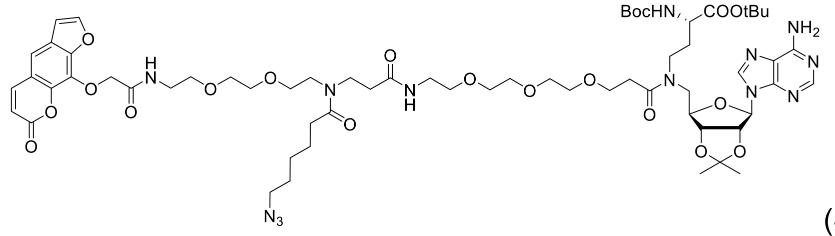

(**S26**)

S25 (86 mg, 0.1 mmol) was dissolved in TFA/CH_2Cl_2 (0.8 mL/0.2 mL) and the mixture was stirred at room temperature for 4 h. Upon completion, the solvent was removed by evaporation and the product was further dried by vacuum. The resulting acid can be directly used for next step. Next, to a solution of the acid obtained above (80.5 mg, 0.1 mmol) in DMF (2 mL) were added HBTU (45 mg, 0.12 mmol) and TEA (41.6 μL, 0.3 mmol). After stirring for 15 min, **S18** (56.4 mg, 0.1 mmol) was added and the reaction mixture was stirred at room temperature under N_2 atmosphere overnight. After completion of the reaction, the solvent was removed by evaporation and the residue was purified by column chromatography on silica-gel (CH_2Cl_2: MeOH = 12:1) to give product **S26** as a light yellow solid (76 mg, 56% yield). ^{1}H NMR (600 MHz, MeOD-d_4) δ 8.30 – 8.25 (m, 2H), 8.05 (d, *J* = 9.6 Hz, 1H), 7.94 (d, *J* = 2.1 Hz, 1H), 7.65

(s, 1H), 7.00 (d, *J* = 2.2 Hz, 1H), 6.42 (d, *J* = 9.6 Hz, 1H), 6.25 – 6.14 (m, 1H), 5.49 (d, *J* = 6.3 Hz, 1H), 5.07 (dd, *J* = 22.7, 19.2 Hz, 1H), 4.96 (t, *J* = 2.3 Hz, 2H), 4.41 (dd, *J* = 26.4, 21.7 Hz, 1H), 3.94 – 3.89 (m, 1H), 3.75 (t, *J* = 6.0 Hz, 1H), 3.71 (t, *J* = 6.9 Hz, 1H), 3.68 – 3.46 (m, 26H), 3.42 – 3.38 (m, 1H), 3.35 (dd, *J* = 5.5, 2.7 Hz, 1H), 3.28 (dt, *J* = 6.8, 5.4 Hz, 2H), 2.70 – 2.57 (m, 1H), 2.52 (td, *J* = 6.9, 2.1 Hz, 1H), 2.46 (t, *J* = 7.1 Hz, 1H), 2.41 (ddd, *J* = 10.5, 6.5, 2.6 Hz, 2H), 2.37 – 2.19 (m, 1H), 2.06 – 1.86 (m, 1H), 1.81 – 1.64 (m, 1H), 1.63 – 1.54 (m, 7H), 1.47 – 1.29 (m, 25H). ^{13}C NMR (151 MHz, MeOD-*d₄*) δ 174.47, 174.14, 172.54, 171.89, 169.42, 160.63, 156.06, 152.87, 152.75, 148.66, 147.45, 147.22, 145.09, 142.69, 140.90, 130.43, 126.55, 119.44, 116.67, 114.62, 114.53, 114.25, 113.94, 106.77, 90.01, 84.05, 83.57, 82.11, 81.31, 71.60, 70.36, 70.11, 70.05, 69.85, 69.17, 69.02, 68.85, 68.74, 67.08, 66.86, 50.94, 45.52, 43.10, 39.01, 38.72, 34.94, 32.90, 32.46, 32.25, 28.36, 27.36, 26.86, 26.84, 26.14, 26.11, 24.62, 24.54, 24.20. ESI-MS (m/z): [M+H$^+$] calcd. for $C_{63}H_{92}N_{13}O_{20}^+$ 1350.66; found, 1350.55.

Synthesis of **2b**

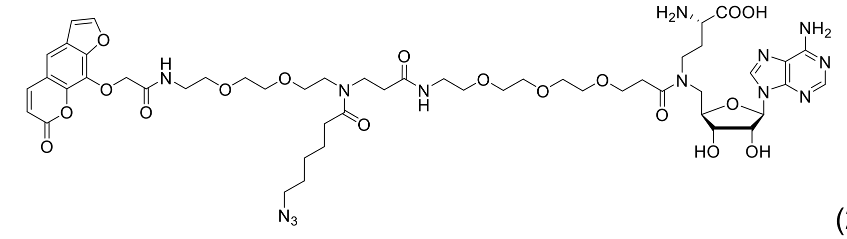

(**2b**)

S26 (20 mg, 0.0148 mmol) was dissolved in a mixture of TFA/H_2O (0.9 mL/0.1 mL). The reaction mixture was stirred at room temperature for 3 h. After completion of the reaction, the solvent was removed by evaporation. EtOH was added to co-evaporate for 3 times, followed by vacuum drying to give product **2b** as a light yellow solid (17.1 mg, 100% yield). ^{1}H NMR (600 MHz, MeOD-d_4) δ 8.46 (d, *J* = 3.0 Hz, 1H), 8.43 (d, *J* = 4.5 Hz, 1H), 8.06 (d, *J* = 9.6 Hz, 1H), 7.95 – 7.92 (m, 1H), 7.66 (s, 1H), 7.01 (d, *J* = 2.2 Hz, 1H), 6.43 (d, *J* = 9.6 Hz, 1H), 6.08 – 6.04 (m, 1H), 4.97 (d, *J* = 1.3 Hz, 2H), 4.72 (dt, *J* = 8.7, 4.2 Hz, 1H), 4.50 – 4.45 (m, 1H), 4.32 – 4.25 (m, 1H), 3.92 – 3.86 (m, 1H), 3.82 – 3.77 (m, 1H), 3.72 (t, *J* = 6.8 Hz, 1H), 3.66 – 3.51 (m, 26H), 3.36 (dt, *J* = 8.5, 4.2 Hz, 2H), 3.28 (q, *J* = 6.7 Hz, 2H), 2.78 (dd, *J* = 14.1, 6.7 Hz, 1H), 2.55 (ddd, *J* = 18.9, 14.6, 8.4 Hz, 2H), 2.47 (t, *J* = 7.1 Hz, 1H), 2.42 (t, *J* = 7.5 Hz, 2H), 2.37 – 2.27 (m, 1H), 2.13 – 2.04 (m, 1H), 1.64 – 1.53 (m, 4H), 1.39 (dd, *J* = 9.1, 5.2 Hz, 4H). ^{13}C NMR (151 MHz, MeOD-d_4) δ 174.57, 174.38, 174.23, 170.10, 160.72, 148.34, 147.47, 147.46, 147.22, 145.17, 145.08, 143.02, 142.98, 142.67, 130.43, 126.60, 119.59, 116.68, 114.67, 113.92, 106.79, 90.42, 73.54, 71.58, 71.29, 70.36, 70.04, 69.84, 69.81, 69.19, 69.01, 68.88, 68.74, 66.92, 64.29, 56.94, 50.94, 42.13, 38.72, 34.96, 33.92, 33.00, 32.48, 32.27, 28.35, 28.00, 26.14, 26.11, 24.64, 24.56. ESI-MS (m/z): [M+H$^+$] calcd. for $C_{51}H_{72}N_{13}O_{18}^+$ 1154.51; found, 1154.40.

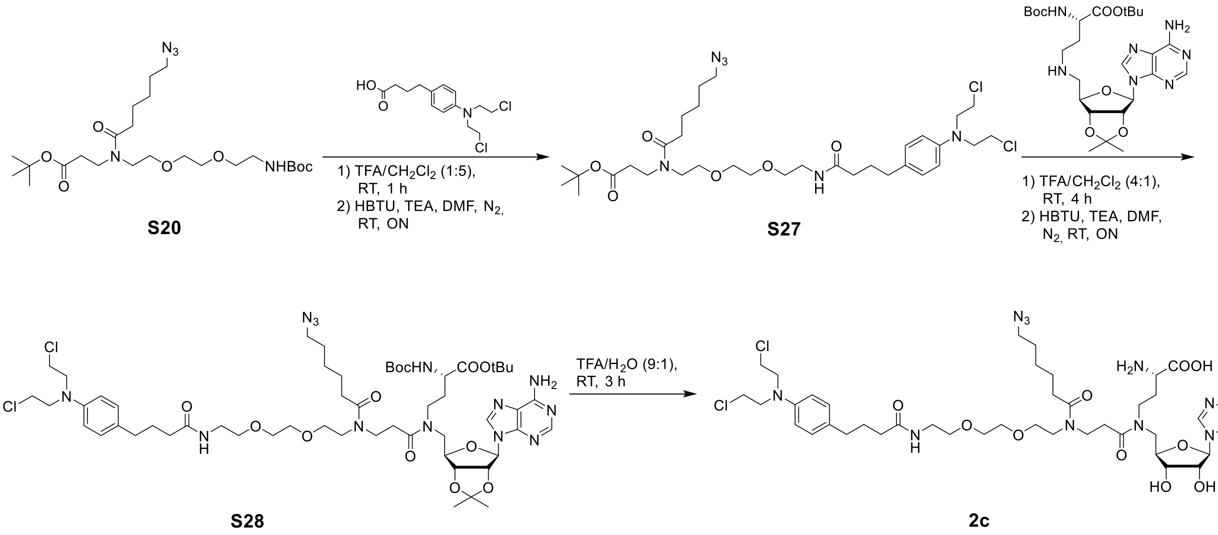

Synthesis of **S27**

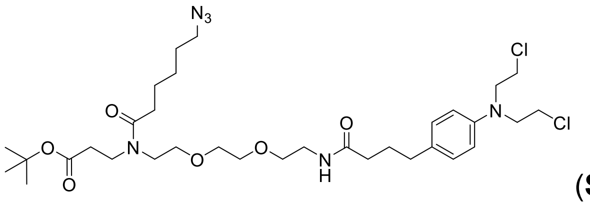

(**S27**)

S20 (200 mg, 0.388 mmol) was dissolved in TFA/CH_2Cl_2 (0.3 mL/1.5 mL) and the mixture was stirred at room temperature for 1 h. Upon completion, the solvent was removed by evaporation and the product was further dried by vacuum. The resulting amine can be directly used for next step. Next, to a solution of chlorambucil (118 mg, 0.388 mmol) in DMF (2 mL) were added HBTU (176 mg, 0.466 mmol) and TEA (161 μL, 1.16 mmol). After stirring for 15 min, the amine obtained from last step in DMF (1 mL) was added and the reaction mixture was stirred at room temperature under N_2 atmosphere overnight. After completion of the reaction, the solvent was removed by evaporation and the residue was purified by column chromatography on silica-gel (CH_2Cl_2: MeOH = 40:1) to give product **S27** as a light yellow oil (234 mg, 86% yield). ^{1}H NMR (400 MHz, MeOD-*d₄*) δ 7.09 (d, *J* = 8.6 Hz, 2H), 6.70 (d, *J* = 8.8 Hz, 2H), 3.75 – 3.53 (m, 20H), 3.36 (t, *J* = 5.5 Hz, 2H), 3.30 (dd, *J* = 6.8, 3.1 Hz, 2H), 2.55 (ddd, *J* = 14.4, 11.9, 7.2 Hz, 4H), 2.44 (dd, *J* = 15.6, 8.1 Hz, 2H), 2.22 (t, *J* = 7.5 Hz, 2H), 1.93 – 1.84 (m, 2H), 1.69 – 1.57 (m, 4H), 1.47 (d, *J* = 2.3 Hz, 9H), 1.45 – 1.37 (m, 2H). ^{13}C NMR (101 MHz, MeOD-*d₄*) δ 174.76, 174.57, 171.62, 144.60, 130.40, 129.23, 112.15, 80.46, 70.38, 69.96, 69.28, 68.94, 53.17, 50.94, 45.50, 42.59, 40.32, 38.93, 35.09, 33.78, 33.30, 32.50, 28.37, 27.63, 26.96, 26.13, 24.56. ESI-MS (m/z): [M+H$^+$] calcd. for $C_{33}H_{55}Cl_2N_6O_6^+$ 701.36; found, 701.25.

Synthesis of **S28**

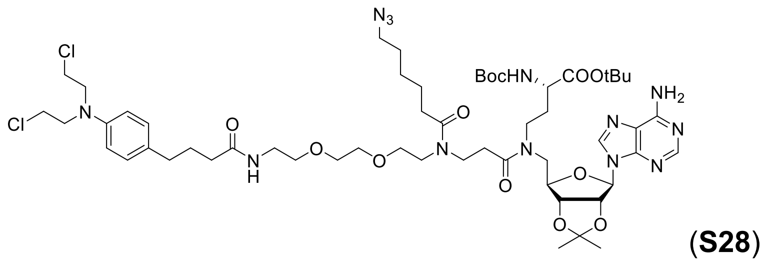

(**S28**)

S27 (70.2 mg, 0.1 mmol) was dissolved in TFA/CH_2Cl_2 (0.8 mL/0.2 mL) and the mixture was stirred at room temperature for 4 h. Upon completion, the solvent was removed by evaporation and the product was further dried by vacuum. The resulting acid can be directly used for next step. Next, to a solution of the acid obtained above (64.6 mg, 0.1 mmol) in DMF (2 mL) were added HBTU (45 mg, 0.12 mmol) and TEA (41.6 μL, 0.3 mmol). After stirring for 15 min, **S18** (56.4 mg, 0.1 mmol) was added and the reaction mixture was stirred at room temperature under N_2 atmosphere overnight. After completion of the reaction, the solvent was removed by evaporation and the residue was purified by column chromatography on silica-gel (CH_2Cl_2: MeOH = 25:1) to give product **S28** as a light yellow solid (60.8 mg, 51% yield). ^{1}H NMR (400 MHz, MeOD-*d₄*) δ 8.32 – 8.23 (m, 2H), 7.06 (dd, *J* = 8.6, 3.5 Hz, 2H), 6.68 (dd, *J* = 8.7, 2.0 Hz, 2H), 6.26 – 6.14 (m, 1H), 5.50 – 5.36 (m, 1H), 5.08 (ddd, *J* = 10.2, 8.5, 4.3 Hz, 1H), 4.51 – 4.35 (m, 1H), 3.96 (dd, *J* = 14.9, 9.0 Hz, 1H), 3.85 – 3.42 (m, 22H), 3.36 (dd, *J* = 6.3, 3.5 Hz, 2H), 3.31 – 3.26 (m, 2H), 2.54 (t, *J* = 7.4 Hz, 2H), 2.42 (dt, *J* = 13.0, 7.4 Hz, 2H), 2.22 (td, *J* = 7.6, 2.2 Hz, 2H), 2.03 (dd, *J* = 27.0, 22.0 Hz, 1H), 1.92 – 1.84 (m, 2H), 1.83 – 1.70 (m, 1H), 1.69 – 1.51 (m, 8H), 1.50 – 1.30 (m, 26H). ^{13}C NMR (101 MHz, MeOD-*d₄*) δ 174.75, 174.28, 172.60, 171.70, 156.61, 156.05, 152.86, 152.79, 148.69, 144.57, 140.76, 130.40, 129.24, 114.65, 114.34, 112.13, 89.78, 84.20, 83.65, 81.99, 79.21, 70.31, 69.95, 69.28, 68.83, 53.16, 50.99, 50.94, 40.35, 38.95, 35.13, 33.80, 32.40, 29.29, 28.40, 28.34, 27.62, 27.37, 26.89, 26.86, 26.19, 26.13, 26.10, 24.61, 24.47, 24.22. ESI-MS (m/z): [M+H$^+$] calcd. for $C_{55}H_{86}Cl_2N_{13}O_{12}^+$ 1190.59; found, 1190.45.

Synthesis of **2c**

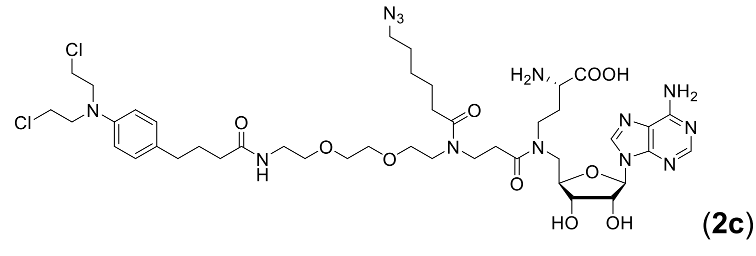

(**2c**)

S28 (20 mg, 0.0168 mmol) was dissolved in a mixture of TFA/H_2O (0.9 mL/0.1 mL). The reaction mixture was stirred at room temperature for 3 h. After completion of the reaction, the solvent was removed by evaporation. EtOH was added to co-evaporate for 3 times, followed by vacuum drying to give product **2c** as a light yellow solid (16.7 mg, 100% yield). ^{1}H NMR (600 MHz, MeOD-*d₄*) δ 8.47 (d, *J* = 8.7 Hz, 1H), 8.43 (d, *J* = 10.6 Hz, 1H), 7.07 (d, *J* = 8.5 Hz, 2H), 6.69 (d, *J* = 8.7 Hz, 2H), 6.09 – 6.04 (m, 1H), 4.66 – 4.59 (m, 1H), 4.48 – 4.43 (m, 1H), 4.25 (dt, *J* = 17.5, 5.8 Hz, 1H), 3.98 – 3.92 (m, 1H), 3.75 – 3.52 (m, 22H), 3.36 (dd, *J* = 11.3, 5.6 Hz, 2H), 3.31 – 3.26 (m, 2H), 2.77 – 2.63 (m, 2H), 2.54 (t, *J* = 7.5 Hz, 2H), 2.40 (dd, *J* =

20.9, 13.5 Hz, 2H), 2.31 – 2.26 (m, 1H), 2.22 (t, *J* = 7.5 Hz, 2H), 2.16 – 2.08 (m, 1H), 1.91 – 1.84 (m, 2H), 1.59 (ddd, *J* = 11.2, 9.4, 5.9 Hz, 4H), 1.44 – 1.28 (m, 4H). ^{13}C NMR (151 MHz, MeOD-*d₄*) δ 174.83, 174.20, 170.01, 151.09, 148.35, 144.68, 144.52, 142.95, 142.82, 130.47, 129.22, 112.19, 90.32, 82.62, 73.81, 71.29, 70.35, 69.96, 69.24, 68.95, 64.28, 56.94, 53.19, 50.97, 50.21, 40.32, 38.92, 35.10, 33.78, 32.44, 31.17, 28.38, 27.87, 27.61, 26.18, 26.09, 24.60, 24.50. ESI-MS (m/z): [M+H$^+$] calcd. for $C_{43}H_{66}Cl_2N_{13}O_{10}^+$ 994.44; found, 994.40.

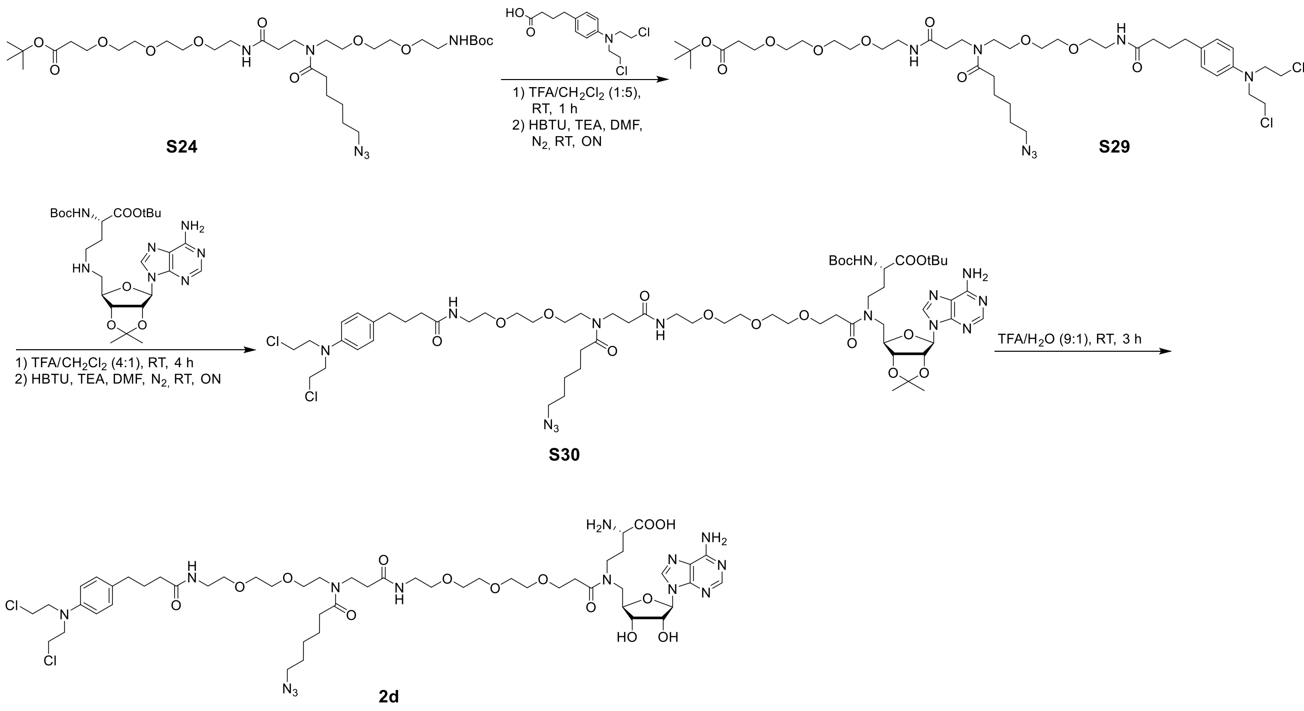

Synthesis of **S29**

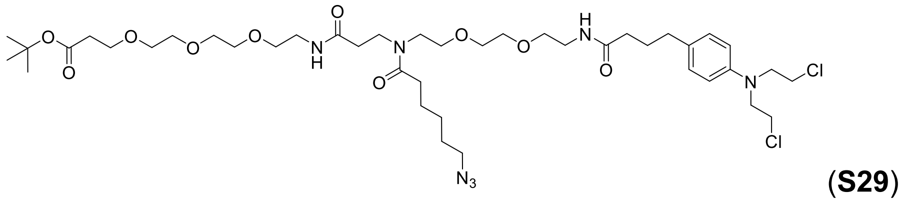

(**S29**)

S24 (173 mg, 0.24 mmol) was dissolved in TFA/CH_2Cl_2 (0.3 mL/1.5 mL) and the mixture was stirred at room temperature for 1 h. Upon completion, the solvent was removed by evaporation and the product was further dried by vacuum. The resulting amine can be directly used for next step. Next, to a solution of chlorambucil (73 mg, 0.24 mmol) in DMF (2 mL) were added HBTU (109 mg, 0.288 mmol) and TEA (100 μL, 0.72 mmol). After stirring for 15 min, the amine obtained from last step in DMF (1 mL) was added and the reaction mixture was stirred at room temperature under N_2 atmosphere overnight. After completion of the reaction, the solvent was removed by evaporation and the residue was purified by column chromatography on silica-gel (CH_2Cl_2: MeOH = 40:1) to give product **S29** as a light yellow oil (174 mg, 80% yield). ^{1}H NMR (400 MHz, $CDCl_3$) δ 7.06 (dd, *J* = 8.6, 2.0 Hz, 2H), 6.61 (d, *J* = 8.7 Hz, 2H), 3.71 – 3.51 (m, 32H), 3.42 (dt, *J* = 10.5, 5.1 Hz, 4H), 3.27 (td, *J* = 6.8, 2.3 Hz, 2H), 2.58 – 2.52 (m, 2H), 2.49 (td, *J* = 6.5, 3.7 Hz, 4H), 2.38 (dt, *J* = 17.7, 7.4 Hz, 2H), 2.20 (dt, *J* = 10.3, 7.6 Hz, 2H), 1.94 –

1.87 (m, 2H), 1.67 – 1.57 (m, 4H), 1.44 (s, 9H), 1.39 (dd, *J* = 10.8, 5.5 Hz, 2H). ^{13}C NMR (101 MHz, $CDCl_3$) δ 173.49, 173.04, 171.33, 170.89, 144.29, 130.92, 129.68, 112.16, 80.57, 70.73, 70.48, 70.36, 70.33, 70.25, 70.06, 69.89, 69.74, 69.34, 66.88, 53.62, 51.29, 47.16, 43.54, 40.56, 39.40, 39.23, 36.23, 35.84, 34.17, 32.96, 32.70, 28.78, 28.11, 27.48, 26.51, 24.69. ESI-MS (m/z): [M+H$^+$] calcd. for $C_{42}H_{72}Cl_2N_7O_{10}^+$ 904.47; found, 904.35.

Synthesis of **S30**

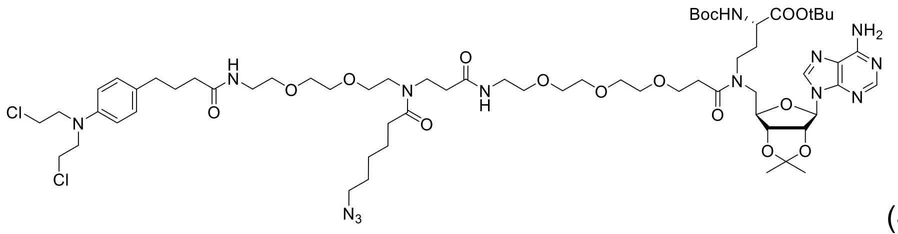

(**S30**)

S29 (90.5 mg, 0.1 mmol) was dissolved in TFA/CH_2Cl_2 (0.8 mL/0.2 mL) and the mixture was stirred at room temperature for 4 h. Upon completion, the solvent was removed by evaporation and the product was further dried by vacuum. The resulting acid can be directly used for next step. Next, to a solution of the acid obtained above (84.9 mg, 0.1 mmol) in DMF (2 mL) were added HBTU (45 mg, 0.12 mmol) and TEA (41.6 µL, 0.3 mmol). After stirring for 15 min, **S18** (56.4 mg, 0.1 mmol) was added and the reaction mixture was stirred at room temperature under N_2 atmosphere overnight. After completion of the reaction, the solvent was removed by evaporation and the residue was purified by column chromatography on silica-gel (CH_2Cl_2: MeOH = 10:1) to give product **S30** as a light yellow solid (74 mg, 53% yield). ^{1}H NMR (600 MHz, MeOD-*d$_4$*) δ 8.31 – 8.26 (m, 2H), 7.07 (d, *J* = 8.5 Hz, 2H), 6.69 (d, *J* = 8.2 Hz, 2H), 6.21 (dd, *J* = 34.9, 1.8 Hz, 1H), 5.51 – 5.46 (m, 1H), 5.15 – 5.05 (m, 1H), 4.42 (dd, *J* = 26.2, 21.6 Hz, 1H), 3.95 – 3.90 (m, 1H), 3.76 – 3.71 (m, 16H), 3.67 (t, *J* = 6.5 Hz, 4H), 3.61 (d, *J* = 1.2 Hz, 6H), 3.57 – 3.51 (m, 8H), 3.38 – 3.35 (m, 4H), 3.31 – 3.28 (m, 2H), 2.70 – 2.57 (m, 1H), 2.56 – 2.42 (m, 6H), 2.35 (dd, *J* = 12.8, 6.0 Hz, 1H), 2.24 – 2.18 (m, 2H), 1.90 – 1.87 (m, 12H), 1.63 – 1.59 (m, 6H), 1.44 (dd, *J* = 12.1, 8.4 Hz, 18H). ^{13}C NMR (151 MHz, MeOD-*d$_4$*) δ 174.71, 174.51, 174.14, 172.55, 171.87, 163.45, 156.10, 152.78, 148.71, 144.59, 140.92, 130.40, 129.24, 119.47, 114.55, 114.26, 112.15, 90.01, 84.07, 83.59, 82.11, 81.32, 70.38, 70.11, 70.05, 69.97, 69.93, 69.29, 69.24, 68.85, 68.67, 67.46, 53.16, 50.96, 43.09, 40.37, 39.12, 38.94, 35.11, 33.81, 32.93, 32.50, 32.29, 28.39, 27.62, 27.38, 26.89, 26.86, 26.18, 26.16, 26.14, 25.10, 24.66, 24.57, 24.23. ESI-MS (m/z): [M+H$^+$] calcd. for $C_{64}H_{103}Cl_2N_{14}O_{16}^+$ 1393.70; found, 1393.60.

Synthesis of **2d**

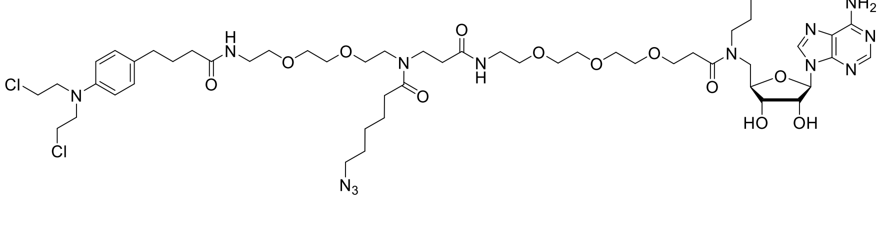

(**2d**)

S30 (20 mg, 0.0143 mmol) was dissolved in a mixture of TFA/H_2O (0.9 mL/0.1 mL). The reaction mixture was stirred at room temperature for 3 h. After completion of the reaction, the solvent was removed by evaporation. EtOH was added to co-evaporate for 3 times, followed by vacuum drying to give product **2d** as a light yellow solid (17.2 mg, 100% yield). ^1H NMR (600 MHz, MeOD-d_4) δ 8.47 (d, J = 2.7 Hz, 1H), 8.46 – 8.43 (m, 1H), 7.08 (d, J = 8.2 Hz, 2H), 6.70 (d, J = 8.7 Hz, 2H), 6.07 (d, J = 3.5 Hz, 1H), 4.71 (dd, J = 9.2, 4.5 Hz, 1H), 4.52 – 4.46 (m, 1H), 4.29 – 4.25 (m, 1H), 3.88 (dt, J = 8.6, 4.3 Hz, 1H), 3.74 (t, J = 7.1 Hz, 6H), 3.67 (dd, J = 7.1, 5.9 Hz, 6H), 3.63 – 3.61 (m, 12H), 3.57 – 3.49 (m, 10H), 3.36 (dt, J = 5.8, 3.8 Hz, 4H), 3.30 (td, J = 6.8, 3.0 Hz, 2H), 2.57 – 2.51 (m, 4H), 2.50 – 2.42 (m, 4H), 2.35 – 2.29 (m, 1H), 2.22 (t, J = 7.5 Hz, 2H), 2.09 (ddd, J = 14.6, 7.3, 4.3 Hz, 1H), 1.87 (dd, J = 15.0, 7.3 Hz, 2H), 1.64 – 1.59 (m, 4H), 1.42 (ddd, J = 20.8, 12.1, 5.4 Hz, 4H). ^{13}C NMR (151 MHz, MeOD-d_4) δ 174.79, 174.38, 172.65, 170.08, 151.12, 148.34, 144.85, 144.54, 143.09, 130.46, 129.23, 119.62, 112.19, 90.45, 82.88, 73.55, 71.28, 70.05, 70.02, 69.96, 69.83, 69.26, 69.20, 68.85, 66.92, 56.93, 53.19, 50.95, 50.17, 49.75, 40.33, 38.93, 35.10, 33.91, 33.79, 33.00, 32.50, 32.30, 28.38, 28.00, 27.62, 26.17, 26.14, 24.67, 24.58. ESI-MS (m/z): [M+H$^+$] calcd. for $C_{52}H_{83}Cl_2N_{14}O_{14}^+$ 1197.56; found, 1197.50.

3.2.3 Synthesis of fluorescent proximity-labeling SAM analogues

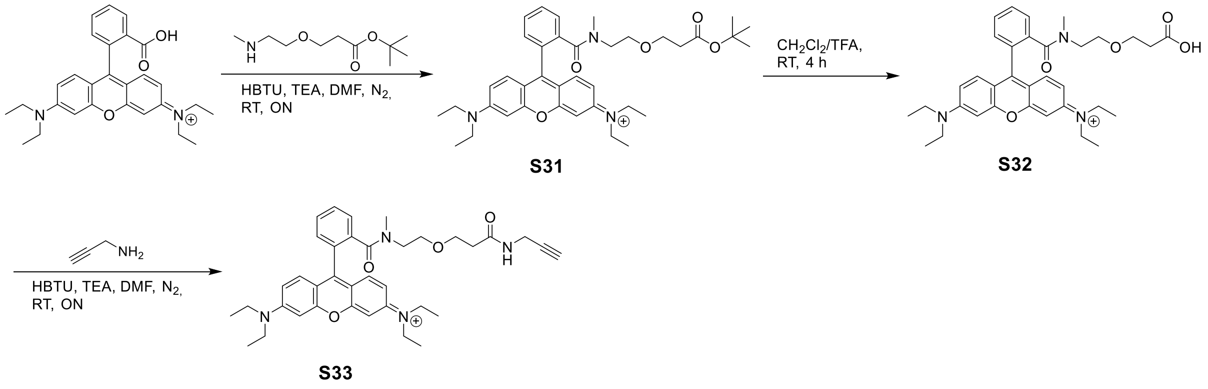

Synthesis of **S31**

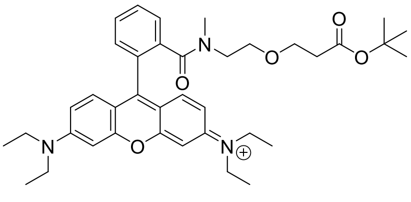

(**S31**)

To a solution of Rhodamine B-COOH (2.39 g, 5 mmol) in DMF (8 mL) were added HBTU (2.08 g, 5.5 mmol) and TEA (2.08 mL, 15 mmol). After stirring for 15 min, the methylamino-PEG1-t-butyl ester linker (1.22 g, 6 mmol) was added and the reaction mixture was stirred at room temperature under N_2 atmosphere overnight. After completion of the reaction, H_2O (100 mL) was added and the mixture was extracted with EtOAc (3 x 50 mL). The combined organic layer was washed with H_2O (2 x 80 mL) and brine (80 mL), dried over anhydrous Na_2SO_4, filtered and concentrated. The residue was purified by column chromatography on silica-gel (CH_2Cl_2: MeOH = 50:1) to give product **S31** as a purple foam (3.15 g, 93% yield). ^1H NMR (400 MHz, $CDCl_3$) δ 8.00 (s, 1H), 7.61 (dddd, J = 9.0, 6.4, 3.0, 1.7 Hz, 3H), 7.26 – 7.20 (m,

2H), 6.96 – 6.87 (m, 2H), 6.75 (dd, J = 15.9, 2.4 Hz, 2H), 3.71 – 3.50 (m, 10H), 3.43 (t, J = 6.3 Hz, 1H), 3.35 (t, J = 5.3 Hz, 2H), 3.22 (t, J = 5.2 Hz, 1H), 2.79 (s, 3H), 2.48 (t, J = 6.3 Hz, 1H), 2.33 (t, J = 6.3 Hz, 1H), 1.43 – 1.41 (m, 9H), 1.31 (t, J = 7.1 Hz, 12H). ESI-MS (m/z): [M$^+$] calcd. for $C_{38}H_{50}N_3O_5^+$ 628.37; found, 628.35.

Synthesis of **S32**

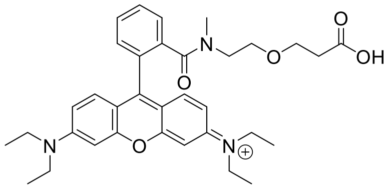

(**S32**)

S31 (0.8 g, 1.27 mmol) was dissolved in TFA/CH_2Cl_2 (2 mL/0.5 mL) and the mixture was stirred at room temperature for 4 h. Upon completion, the solvent was removed by evaporation and the product was further dried by vacuum to give product **S32** as a purple foam (0.73 g, 100% yield), without any further purification. ^{1}H NMR (400 MHz, $CDCl_3$) δ 7.81 – 7.60 (m, 3H), 7.32 (dt, J = 9.5, 6.0 Hz, 3H), 6.96 (t, J = 10.2 Hz, 2H), 6.82 (d, J = 23.2 Hz, 2H), 3.78 – 3.52 (m, 10H), 3.47 (t, J = 6.1 Hz, 1H), 3.40 (dd, J = 12.8, 8.0 Hz, 2H), 3.22 (t, J = 4.9 Hz, 1H), 2.90 (s, 2H), 2.75 (s, 1H), 2.61 (t, J = 5.8 Hz, 1H), 2.46 (t, J = 6.1 Hz, 1H), 1.33 (t, J = 7.1 Hz, 12H). ESI-MS (m/z): [M$^+$] calcd. for $C_{34}H_{42}N_3O_5^+$ 572.31; found, 572.40.

Synthesis of **S33**

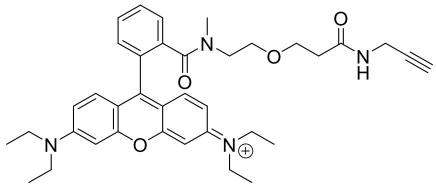

(**S33**)

To a solution of **S32** (309 mg, 0.54 mmol) in DMF (3 mL) were added HBTU (245 mg, 0.648 mmol) and TEA (225 µL, 1.62 mmol). After stirring for 15 min, propargylamine (33 mg, 0.594 mmol) was added and the reaction mixture was stirred at room temperature under N_2 atmosphere overnight. After completion of the reaction, the solvent was removed by evaporation and the residue was purified by column chromatography on silica-gel (CH_2Cl_2: MeOH = 50:1) to give product **S33** as a purple foam (276 mg, 84% yield). ^{1}H NMR (400 MHz, MeOD-d_4) δ 7.82 – 7.66 (m, 3H), 7.50 (ddd, J = 20.5, 5.6, 1.4 Hz, 1H), 7.29 (t, J = 10.0 Hz, 2H), 7.09 (dt, J = 9.6, 3.0 Hz, 2H), 6.97 (dd, J = 9.5, 2.4 Hz, 2H), 3.98 (d, J = 2.5 Hz, 1H), 3.92 (d, J = 2.5 Hz, 1H), 3.71 (d, J = 6.2 Hz, 8H), 3.54 (t, J = 5.1 Hz, 1H), 3.44 (t, J = 6.3 Hz, 1H), 3.38 (t, J = 5.2 Hz, 2H), 3.16 (t, J = 5.3 Hz, 1H), 3.04 (s, 1H), 2.94 (s, 2H), 2.69 (s, 1H), 2.59 (q, J = 2.4 Hz, 1H), 2.48 (t, J = 5.9 Hz, 1H), 2.31 (t, J = 6.3 Hz, 1H), 1.33 (t, J = 7.1 Hz, 12H). ^{13}C NMR (101 MHz, MeOD-d_4) δ 171.73, 169.25, 157.87, 155.79, 155.61, 136.16, 135.98, 131.87, 130.40, 130.22, 129.93, 129.74, 129.51, 129.30, 128.52, 127.42, 113.85, 113.38, 113.34, 95.95, 95.89, 79.32, 70.84, 68.02, 66.49, 46.88, 45.50, 38.38, 35.92, 31.21, 28.11, 27.98, 11.47. ESI-MS (m/z): [M$^+$] calcd. for $C_{37}H_{45}N_4O_4^+$ 609.34; found, 609.25.

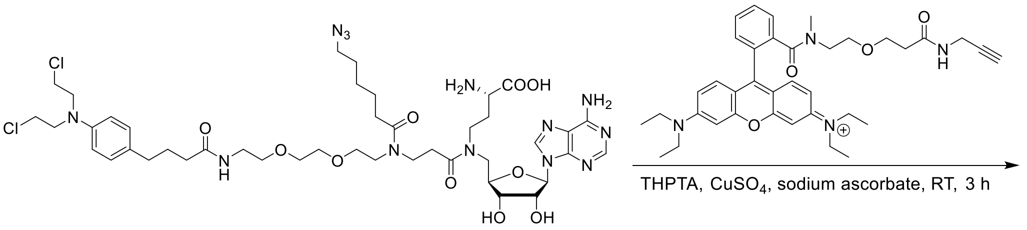

2c

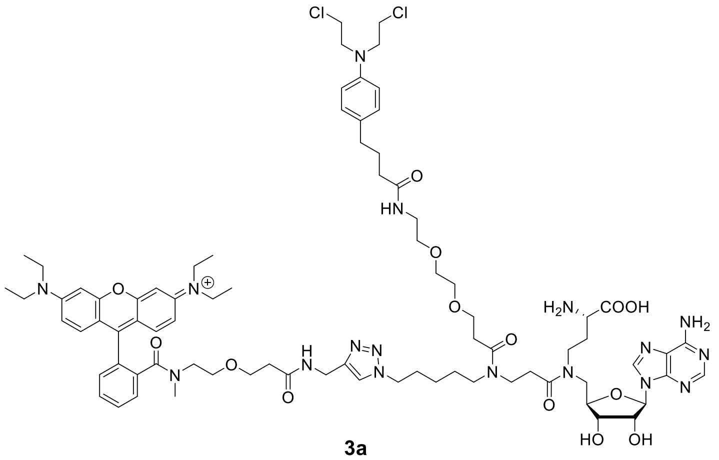

3a

Synthesis of **3a**

To a 1.5 mL of tube was added a premixture of THPTA (100 mM, 30 μL in DMSO, 3.0 eq) and $CuSO_4$ (100 mM, 15 μL in ddH_2O, 1.5 eq), followed by the addition of **2c** (20 mM, 50 μL in DMSO, 1.0 eq) and **S33** (50.7 mM, 19.7 μL in DMSO, 1.0 eq). The click reaction was initiated by adding sodium ascorbate (100 mM, 30 μL in ddH_2O, 3.0 eq). The reaction mixture was incubated at 350 rpm, 25 °C for 3 h. After completion of the reaction, the mixture was purified by reverse HPLC: C18 column (5 μm, 100 x 6.0 mm) with H_2O (0.1% HCOOH)/MeOH as mobile phase. The purification was performed with a gradient of 40-85% MeOH (0-20 min), followed by constant 85% MeOH (20-25 min) at a flow rate of 2.0 mL/min, to give a red stock of **3a** (1.83 mM, 200 μL in DMSO, 36.6% yield). ESI-MS (m/z): [M+H^{2+}] calcd. for $C_{80}H_{111}Cl_2N_{17}O_{14}^{2+}$ 802.39; found, 802.40. [M+2H^{3+}] calcd. for $C_{80}H_{112}Cl_2N_{17}O_{14}^{3+}$ 535.27; found, 535.30.

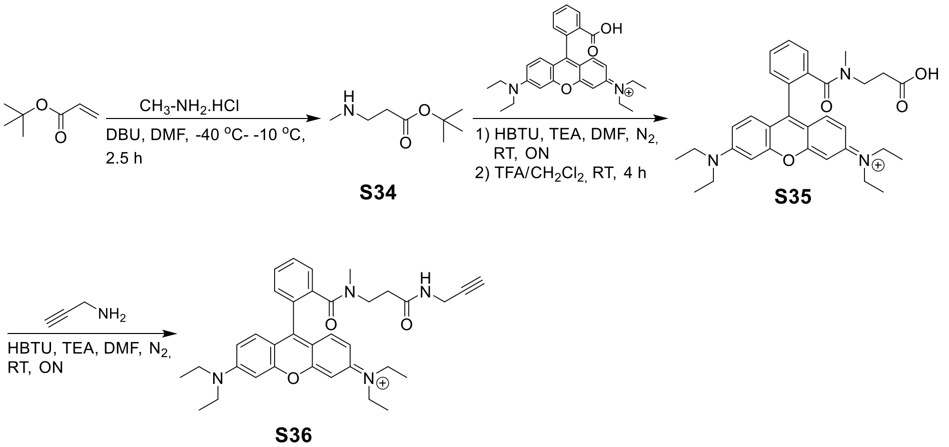

S34 **S35** **S36**

Synthesis of **S34**

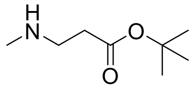

(**S34**)

To a solution of *tert*-butyl acrylate (4.88 mL, 33.3 mmol) in DMF (50 mL) was added methylamine hydrochloride (6.75 g, 100 mmol) and DBU (29.8 mL, 200 mmol) at -40 °C. Then under -10 °C, the reaction mixture was stirred for additional 2.5 h. After completion of the reaction, Et_2O (300 mL) was added and the resulting mixture was washed with brine (4 x 80 mL). The organic layer was dried over anhydrous Na_2SO_4, filtered and concentrated. The residue was purified by column chromatography on silica-gel (CH_2Cl_2: MeOH = 20:1) to give product **S34** as a light yellow oil (3.6 g, 68% yield). ^1H NMR (600 MHz, $CDCl_3$) δ 3.62 (s, 1H), 2.91 (t, *J* = 6.6 Hz, 2H), 2.54 (t, *J* = 6.6 Hz, 2H), 2.51 (s, 3H), 1.45 (s, 9H). ^{13}C NMR (151 MHz, $CDCl_3$) δ 171.61, 81.04, 46.73, 35.52, 34.48, 28.10. ESI-MS (m/z): [M+H$^+$] calcd. for $C_8H_{18}NO_2^+$ 160.13; found, 160.15.

Synthesis of **S35**

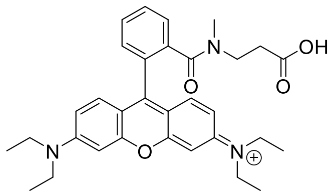

(**S35**)

To a solution of Rhodamine B-COOH (2.39 g, 5 mmol) in DMF (8 mL) were added HBTU (2.08 g, 5.5 mmol) and TEA (2.08 mL, 15 mmol). After stirring for 15 min, **S34** (0.955 g, 6 mmol) was added and the reaction mixture was stirred at room temperature under N_2 atmosphere overnight. After completion of the reaction, H_2O (100 mL) was added and the mixture was extracted with EtOAc (3 x 50 mL). The combined organic layer was washed with H_2O (2 x 80 mL) and brine (80 mL), dried over anhydrous Na_2SO_4, filtered and concentrated. The residue was purified by column chromatography on silica-gel (CH_2Cl_2: MeOH = 40:1) to give product as a purple foam (2.87 g, 91% yield). Subsequently, the purple foam (0.87 g, 1.5 mmol) was dissolved in TFA/CH_2Cl_2 (2 mL/0.5 mL) and the mixture was stirred at room temperature for 4 h. Upon completion, the solvent was removed by evaporation and the product was further dried by vacuum to give product **S35** as a purple foam (0.79 g, 100% yield), without any further purification. ^1H NMR (400 MHz, MeOD-*d$_4$*) δ 7.84 – 7.74 (m, 2H), 7.73 – 7.64 (m, 1H), 7.58 – 7.49 (m, 1H), 7.31 (dd, *J* = 9.4, 4.8 Hz, 2H), 7.09 (dd, *J* = 9.5, 2.3 Hz, 2H), 6.99 (dd, *J* = 6.0, 2.4 Hz, 2H), 3.75 – 3.68 (m, 8H), 3.43 (dt, *J* = 10.7, 6.8 Hz, 2H), 2.90 (t, *J* = 9.9 Hz, 3H), 2.75 – 2.66 (m, 1H), 2.08 (dd, *J* = 12.4, 5.7 Hz, 1H), 1.34 (t, *J* = 7.1 Hz, 12H). ^{13}C NMR (101 MHz, MeOD-*d$_4$*) δ 171.80, 169.35, 157.92, 155.82, 155.40, 136.11, 131.86, 131.73, 130.40, 130.31, 130.26, 130.03, 130.00, 129.56, 127.49, 127.37, 113.82, 113.33, 113.27, 96.07, 95.96, 50.71, 45.49, 43.30, 37.68, 37.50, 30.53, 11.42. ESI-MS (m/z): [M$^+$] calcd. for $C_{32}H_{38}N_3O_4^+$ 528.29; found, 528.20.

Synthesis of **S36**

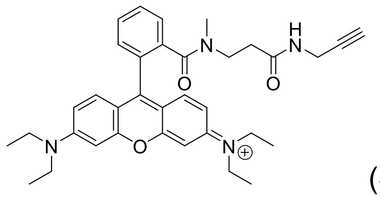

(**S36**)

To a solution of **S35** (528 mg, 1.0 mmol) in DMF (3 mL) were added HBTU (454 mg, 1.2 mmol) and TEA (417 μL, 3 mmol). After stirring for 15 min, propargylamine (61 mg, 1.1 mmol) was added and the reaction mixture was stirred at room temperature under N_2 atmosphere overnight. After completion of the reaction, the solvent was removed by evaporation and the residue was purified by column chromatography on silica-gel (CH_2Cl_2: MeOH = 50:1) to give product **S36** as a purple foam (452 mg, 80% yield). ^1H NMR (400 MHz, MeOD-d_4) δ 7.76 (dd, J = 6.1, 2.7 Hz, 2H), 7.69 (dd, J = 6.5, 2.3 Hz, 1H), 7.54 – 7.48 (m, 1H), 7.29 (d, J = 9.5 Hz, 2H), 7.08 (dd, J = 9.5, 2.3 Hz, 2H), 6.97 (d, J = 2.0 Hz, 2H), 4.07 (d, J = 2.4 Hz, 1H), 3.85 (d, J = 2.4 Hz, 1H), 3.71 (q, J = 7.1 Hz, 8H), 3.43 (t, J = 6.8 Hz, 2H), 3.04 (s, 3H), 2.69 (s, 1H), 2.62 (t, J = 2.4 Hz, 1H), 2.08 (t, J = 6.9 Hz, 1H), 1.33 (s, 12H). ^{13}C NMR (101 MHz, MeOD-d_4) δ 171.21, 169.31, 157.90, 155.79, 155.56, 135.99, 135.76, 131.87, 131.77, 130.48, 130.27, 130.15, 129.94, 129.87, 129.57, 127.47, 113.82, 113.39, 113.33, 95.95, 79.13, 73.24, 71.02, 46.58, 45.48, 38.77, 37.55, 32.48, 28.04, 11.44, 7.82. ESI-MS (m/z): [M^+] calcd. for $C_{35}H_{41}N_4O_3^+$ 565.32; found, 565.20.

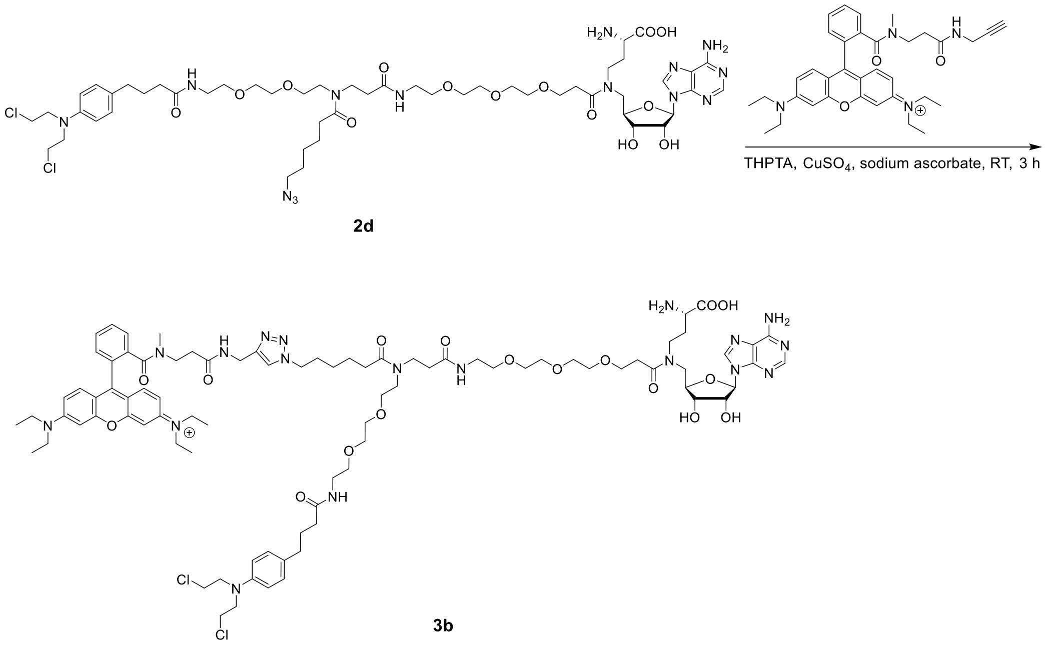

Synthesis of **3b**

To a 1.5 mL of tube was added a premixture of THPTA (100 mM, 30 μL in DMSO, 3.0 eq) and $CuSO_4$ (100 mM, 15 μL in ddH_2O, 1.5 eq), followed by the addition of **2d** (20 mM, 50 μL in DMSO, 1.0 eq) and **S37** (33.2 mM, 30 μL in DMSO, 1.0 eq). The click reaction was initiated by adding sodium ascorbate (100 mM, 30 μL in ddH_2O, 3.0 eq). The reaction mixture was incubated at 350 rpm, 25 °C for 3 h. After completion of the reaction, the mixture was purified by reverse HPLC: C18 column (5 μm, 100 x 6.0 mm) with H_2O (0.1% HCOOH)/MeOH as

mobile phase. The purification was performed with a gradient of 40-85% MeOH (0-20 min), followed by constant 85% MeOH (20-25 min) at a flow rate of 2.0 mL/min, to give a red stock of **3b** (1.24 mM, 200 µL in DMSO, 24.8% yield). ESI-MS (m/z): $[M+H^{2+}]$ calcd. for $C_{87}H_{124}Cl_2N_{18}O_{17}^{2+}$ 881.94; found, 882.00. $[M+2H^{3+}]$ calcd. for $C_{87}H_{125}Cl_2N_{18}O_{17}^{3+}$ 588.30; found, 588.40.

3.2.4 Synthesis of the double-activated SAM analogues

Synthesis of **AdoYnAtto647N**

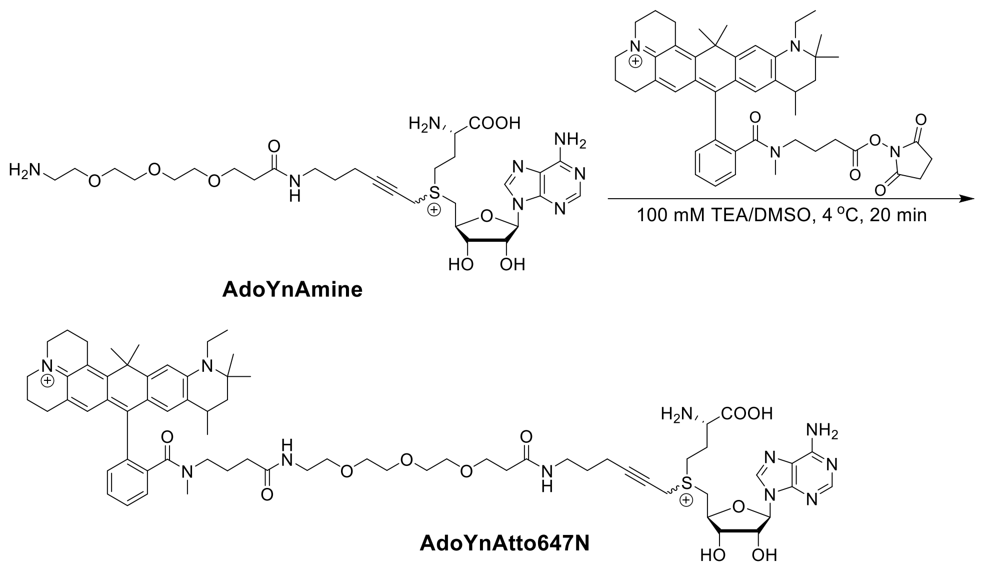

The SAM analogue **AdoYnAmine** was prepared as previously reported.[7] To a 1.5 mL of tube was added **AdoYnAmine** (11 mM, 50 µL in ddH_2O-0.1% HCOOH, 1.0 eq) and Atto647N-NHS (5 mM, 110 µL in DMSO, 1.0 eq). The reaction tube was cooled on ice for 3 min, followed by the addition of TEA (100 mM, 100 µL in DMSO). The reaction mixture was incubated at 350 rpm, 4 °C for 20 min. After completion of the reaction, HCOOH (26 µL) was added to quench the reaction and stabilize the product, and the mixture was purified by reverse HPLC: C18 column (5 µm, 100 x 6.0 mm) with H_2O (0.1% HCOOH)/MeOH as mobile phase. The purification was performed with a gradient of 50-85% MeOH (0-20 min), followed by constant 85% MeOH (20-25 min) at a flow rate of 2.0 mL/min, to give a blue stock of **AdoYnAtto647N** (0.62 mM, 280 µL in ddH_2O-0.1% HCOOH, 31.6% yield). ESI-MS (m/z): $[M^{2+}]$ calcd. for $C_{71}H_{97}N_{11}O_{11}S^{2+}$ 656.36; found, 656.30. $[M+H^{3+}]$ calcd. for $C_{71}H_{98}N_{11}O_{11}S^{3+}$ 437.57; found, 437.55.

Synthesis of **AdoYnRho110**

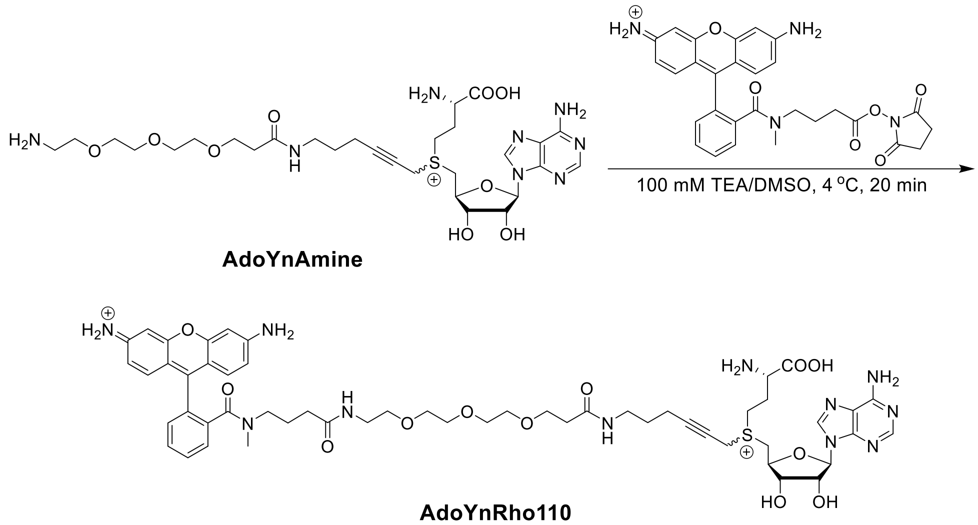

To a 1.5 mL of tube was added **AdoYnAmine** (11 mM, 50 µL in ddH_2O-0.1% HCOOH, 1.0 eq) and Rho110-NHS (5 mM, 110 µL in DMSO, 1.0 eq). The reaction tube was cooled on ice for 3 min, followed by the addition of TEA (100 mM, 100 µL in DMSO). The reaction mixture was incubated at 350 rpm, 4 °C for 20 min. After completion of the reaction, HCOOH (26 µL) was added to quench the reaction and stabilize the product, and the mixture was purified by reverse HPLC: C18 column (5 µm, 100 x 6.0 mm) with H_2O (0.1% HCOOH)/MeOH as mobile phase. The purification was performed with a gradient of 50-85% MeOH (0-20 min), followed by constant 85% MeOH (20-25 min) at a flow rate of 2.0 mL/min, to give a yellow-green stock of **AdoYnRho110** (0.46 mM, 260 µL in ddH_2O-0.1% HCOOH, 21.7% yield). ESI-MS (m/z): [M^{2+}] calcd. for $C_{54}H_{69}N_{11}O_{12}S^{2+}$ 547.74; found, 547.80.

4 Copies of ^1H and ^{13}C NMR Spectra

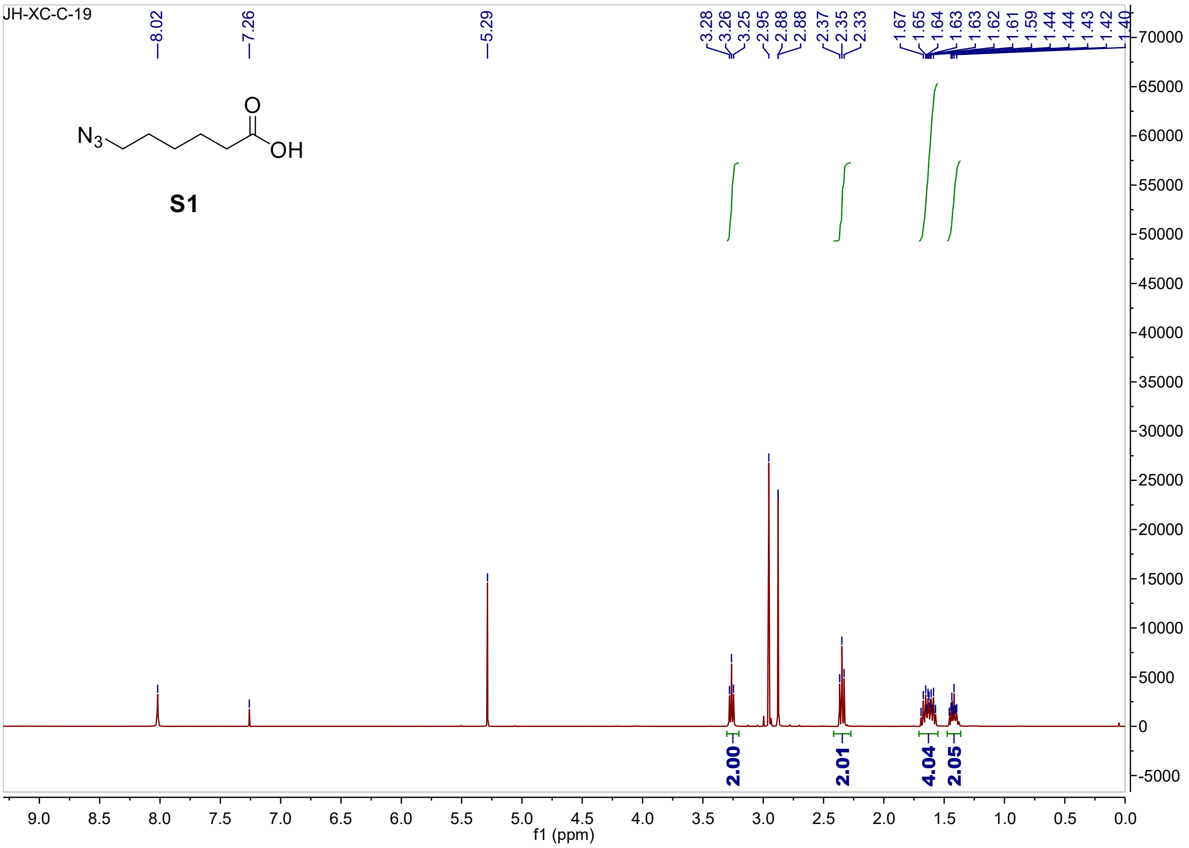

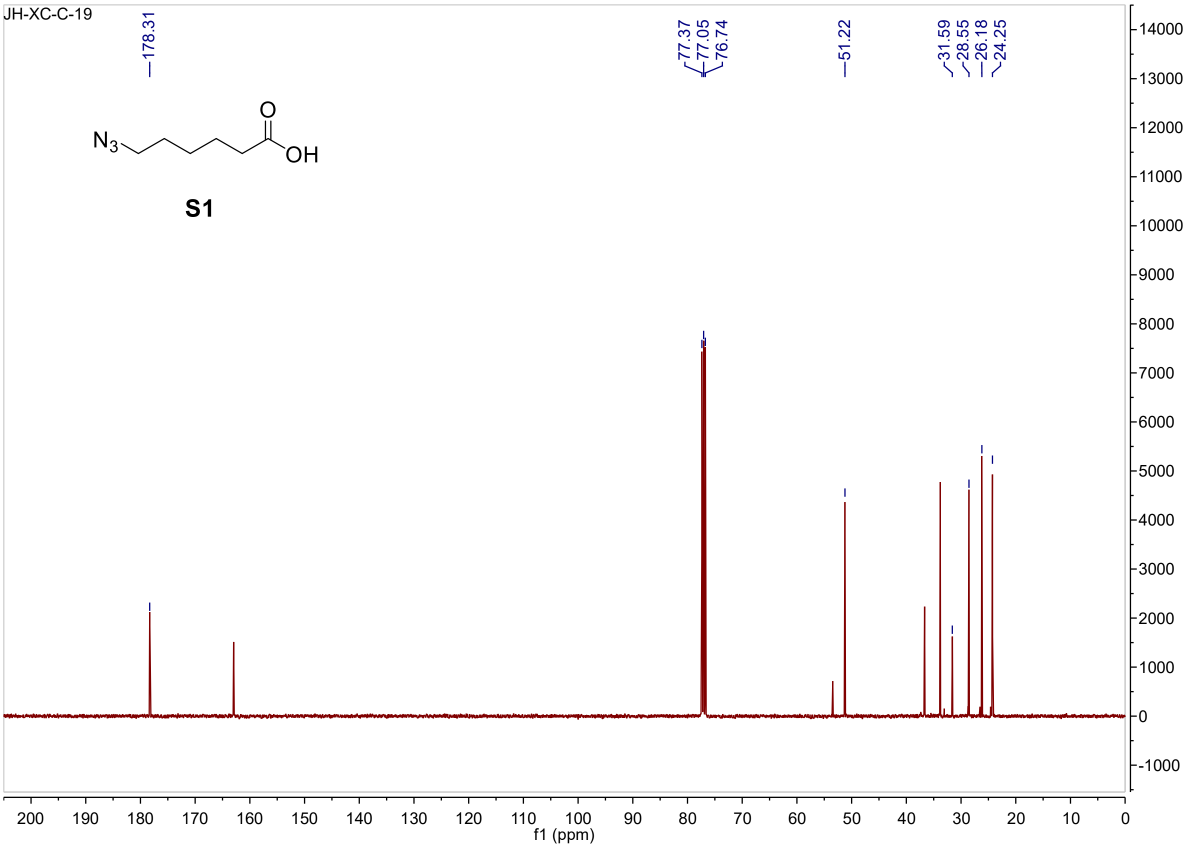

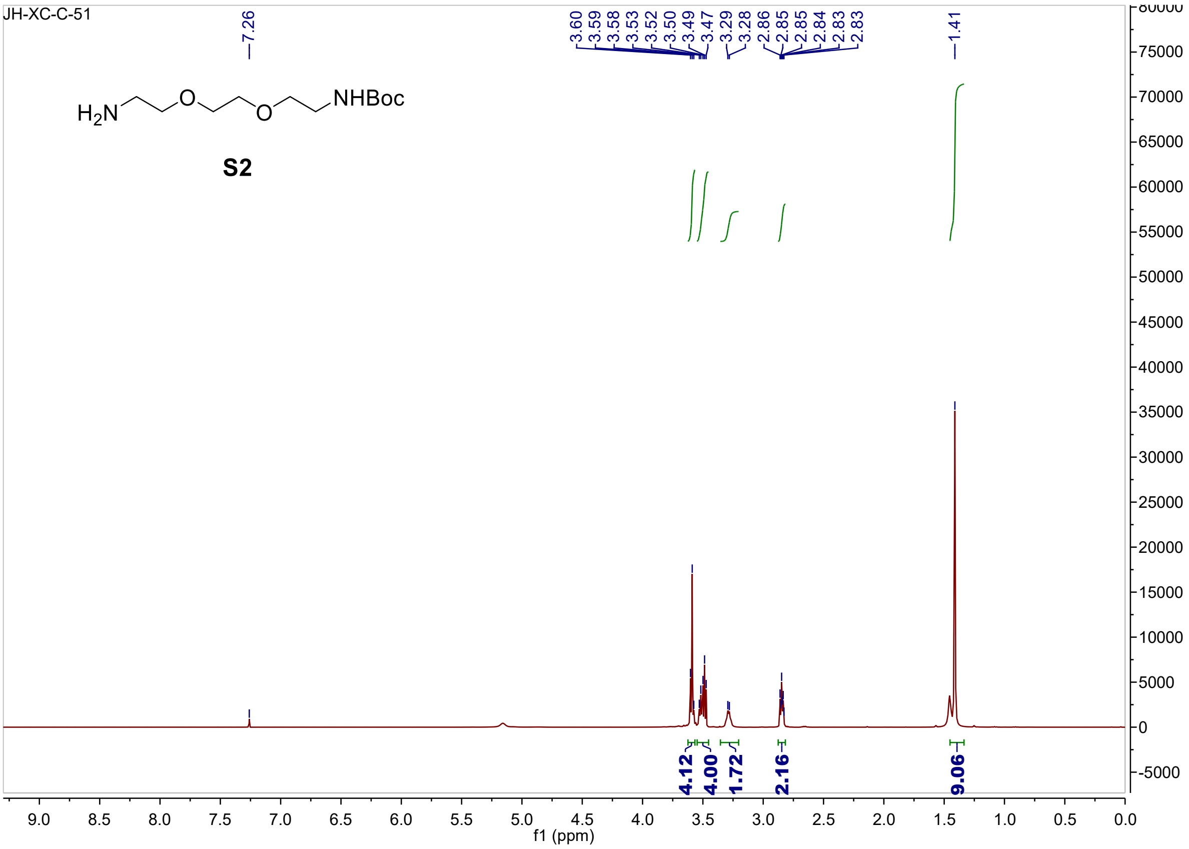
JH-XC-C-51
7.26
3.60
3.59
3.58
3.53
3.52
3.50
3.49
3.47
3.29
3.28
2.86
2.85
2.85
2.84
2.83
2.83
1.41
H2N
O
O
NHBoc
S2
4.12
4.00
1.72
2.16
9.06
f1 (ppm)

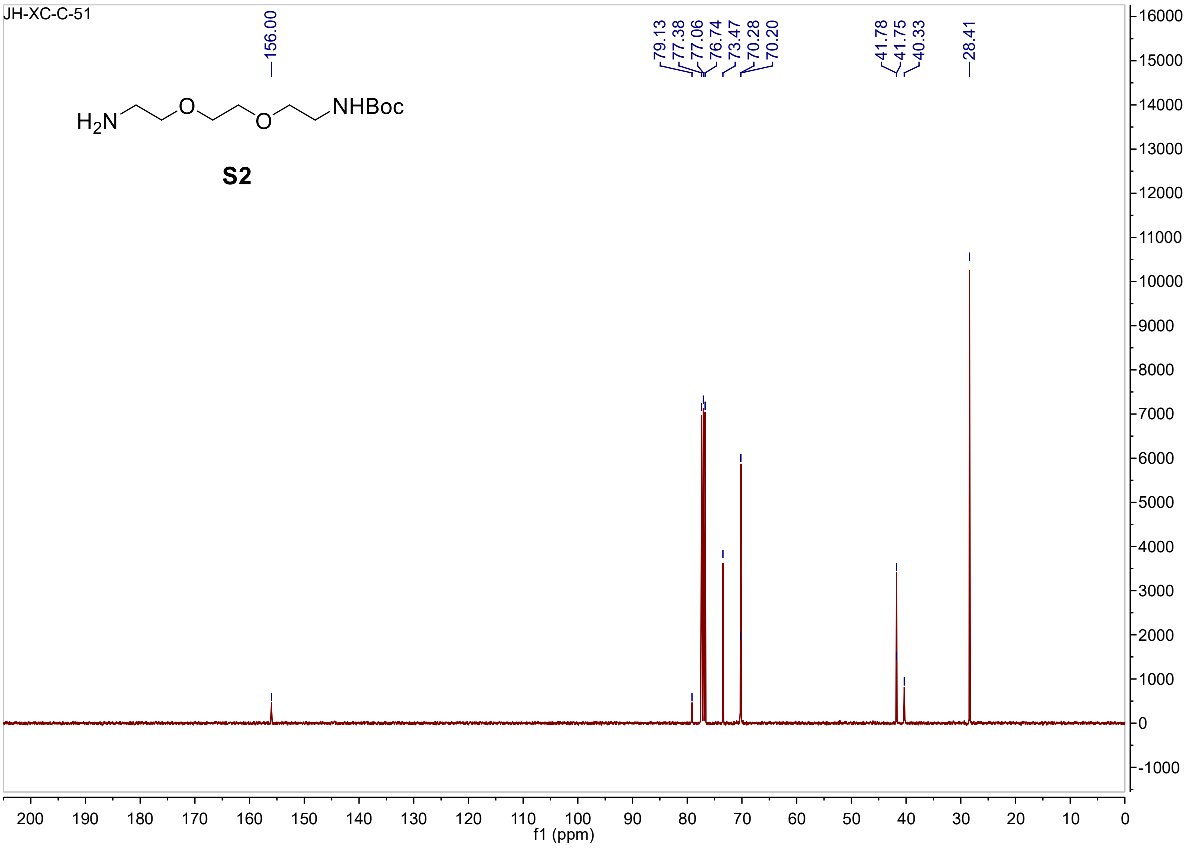
JH-XC-C-51
156.00
79.13
77.38
77.06
76.74
73.47
70.28
70.20
41.78
41.75
40.33
28.41
H2N
O
O
NHBoc
S2
f1 (ppm)

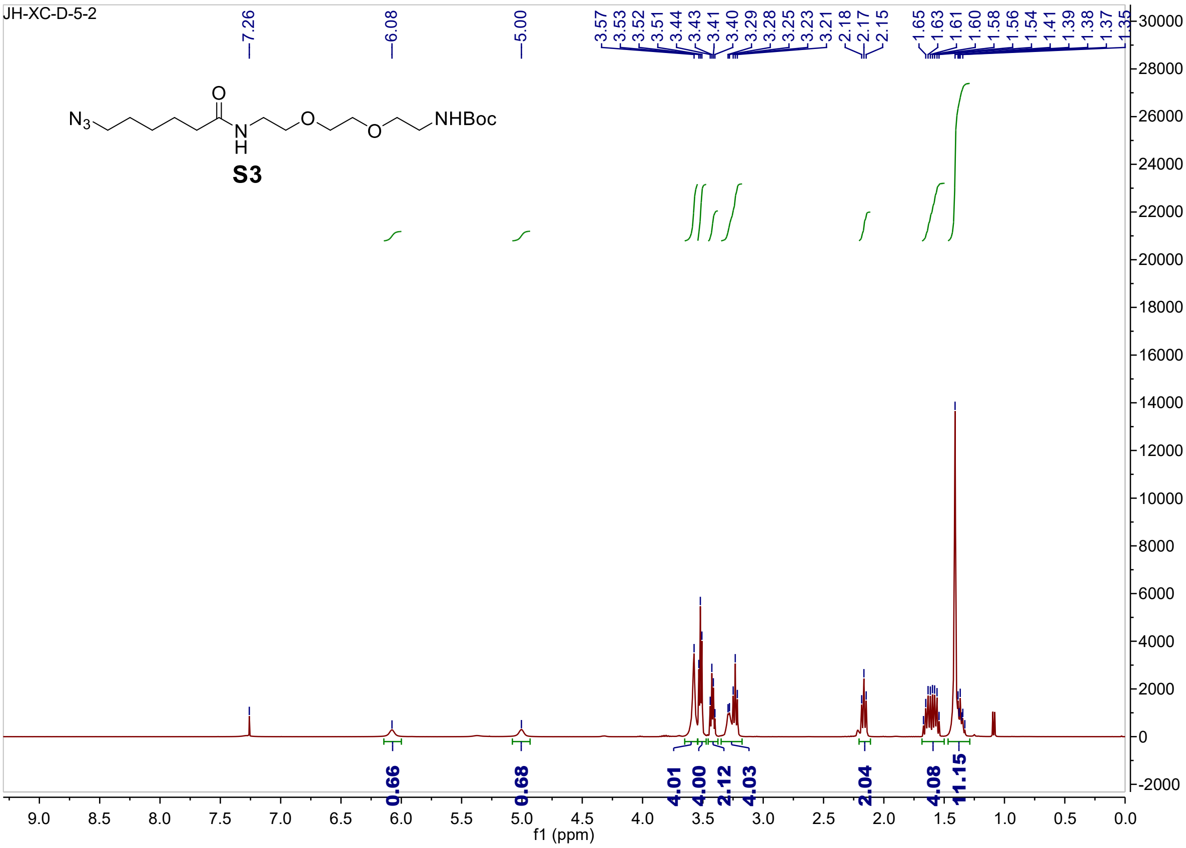

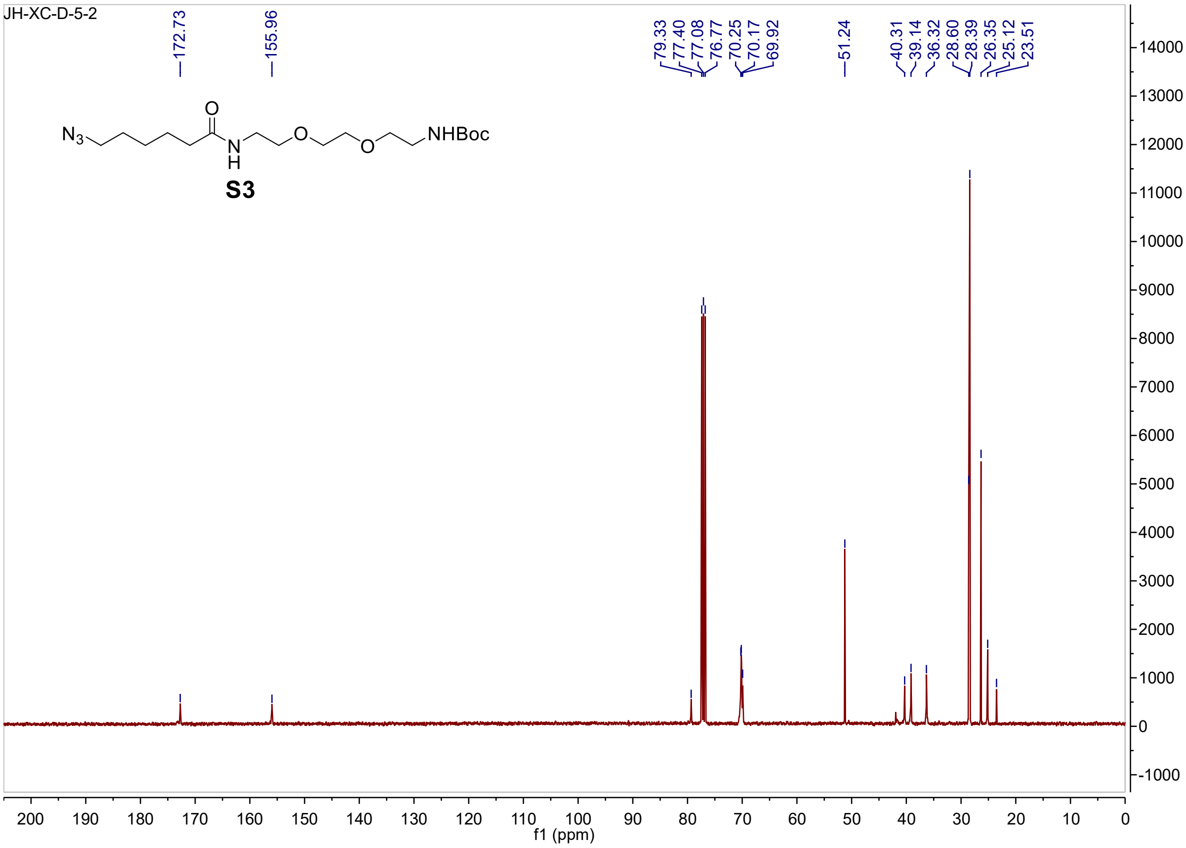

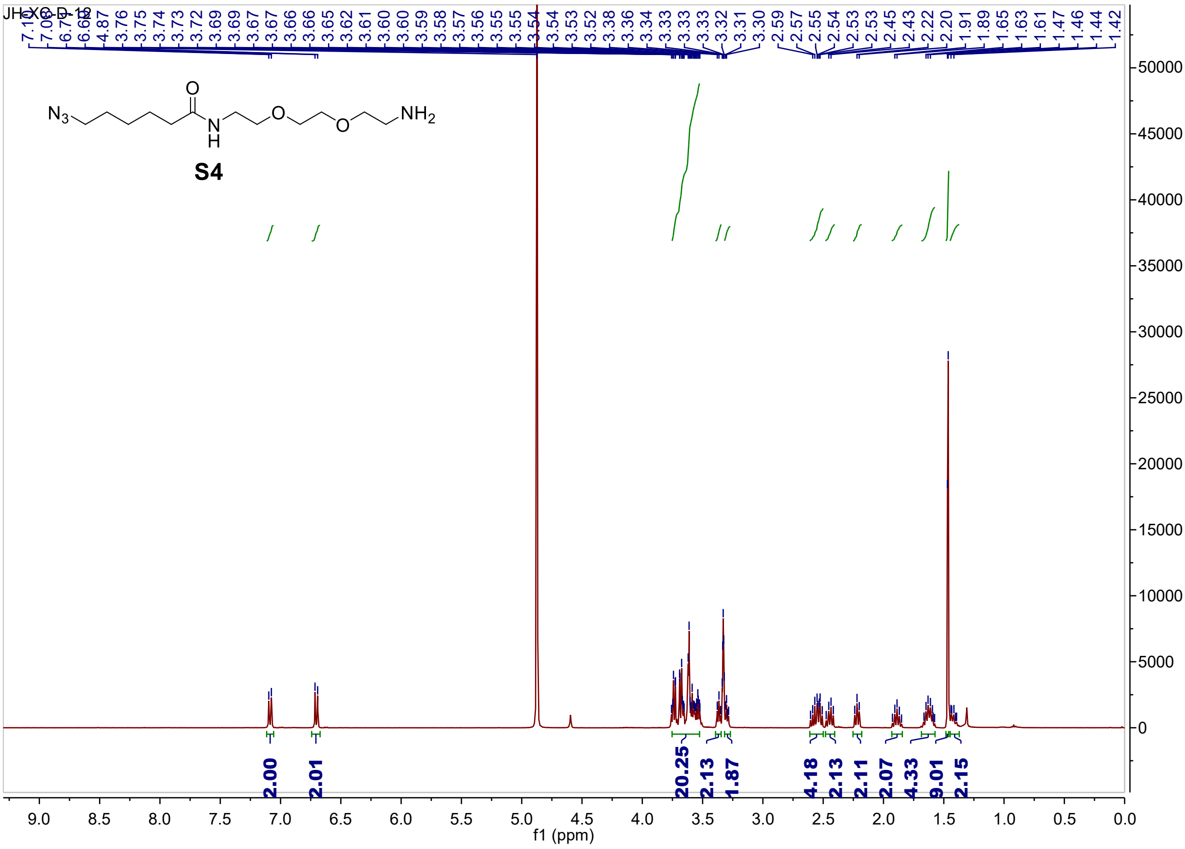
JH-XC-D-12
7.06
6.70
6.68
4.87
3.76
3.75
3.74
3.73
3.72
3.69
3.69
3.67
3.66
3.66
3.65
3.62
3.61
3.60
3.59
3.58
3.57
3.56
3.55
3.55
3.54
3.54
3.53
3.52
3.38
3.36
3.34
3.33
3.33
3.33
3.32
3.31
3.30
2.59
2.57
2.55
2.54
2.53
2.53
2.45
2.43
2.22
2.20
1.91
1.89
1.65
1.63
1.61
1.47
1.46
1.44
1.42
N3
O
N
H
O
O
NH2
S4
2.00
2.01
20.25
2.13
1.87
4.18
2.13
2.11
2.07
4.33
9.01
2.15
f1 (ppm)

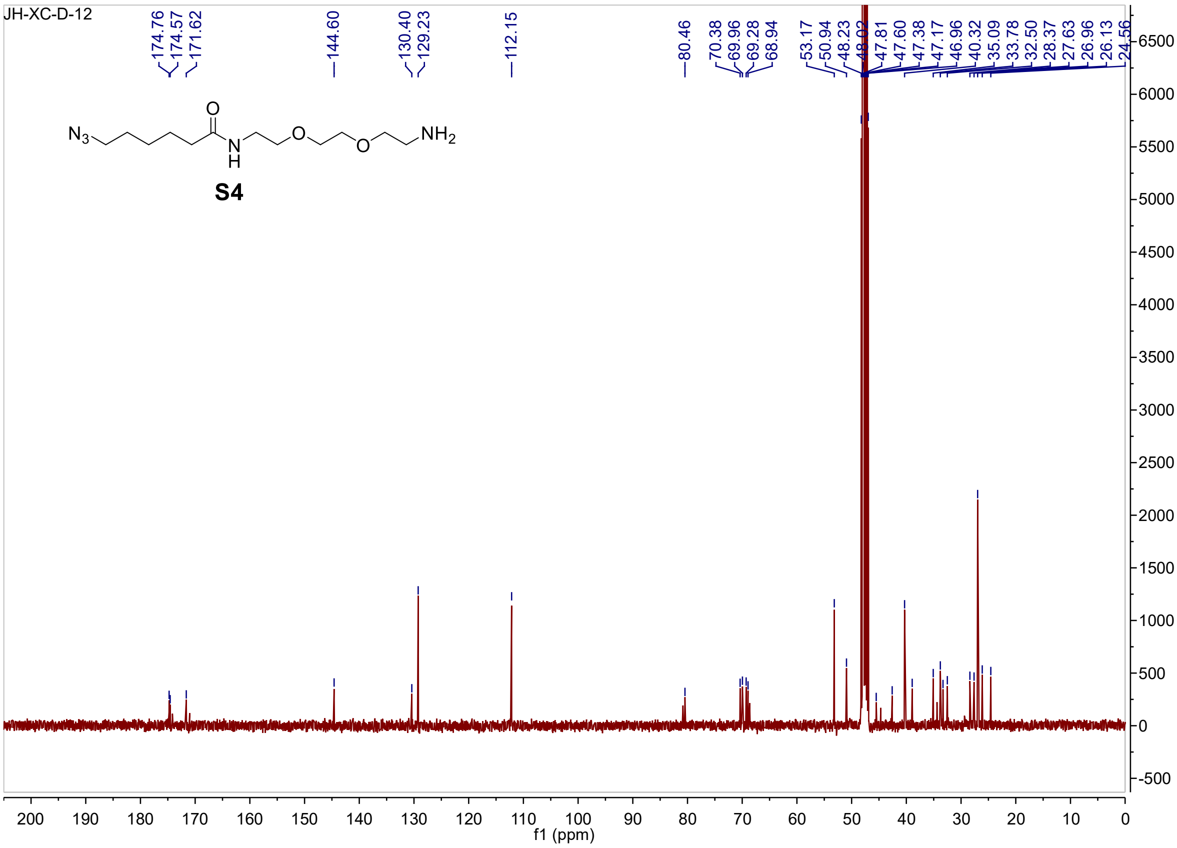
JH-XC-D-12
174.76
174.57
171.62
144.60
130.40
129.23
112.15
80.46
70.38
69.96
69.28
68.94
53.17
50.94
48.23
47.81
47.60
47.38
47.17
46.96
40.32
35.09
33.78
32.50
28.37
27.63
26.96
26.13
24.56
N3
O
N
H
O
O
NH2
S4
f1 (ppm)

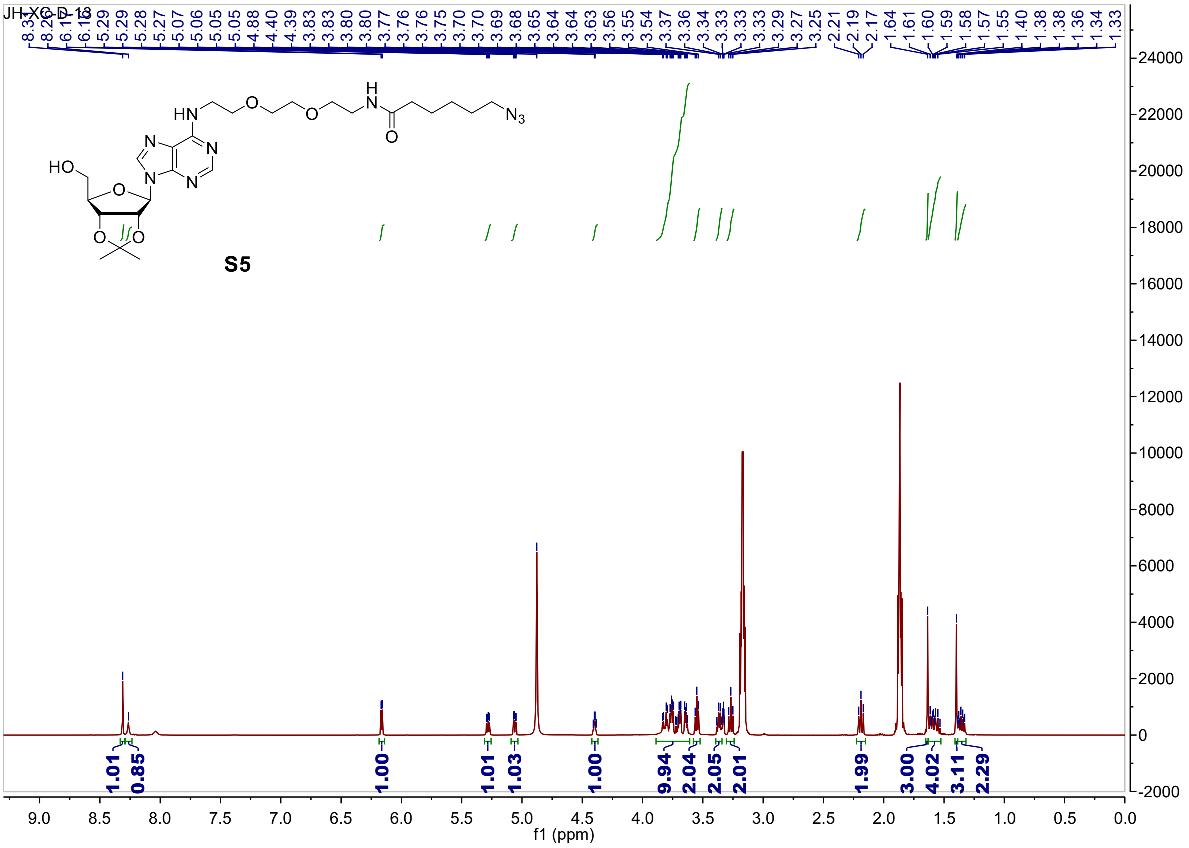

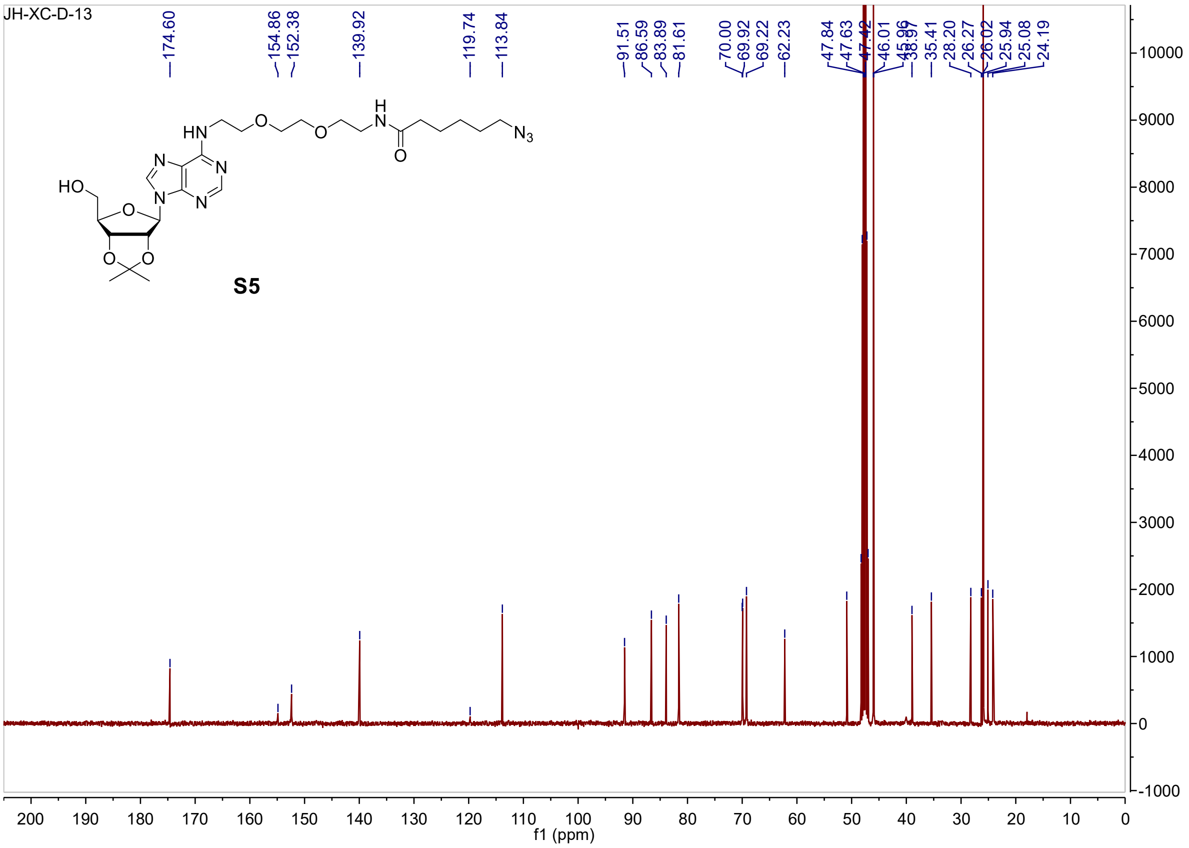

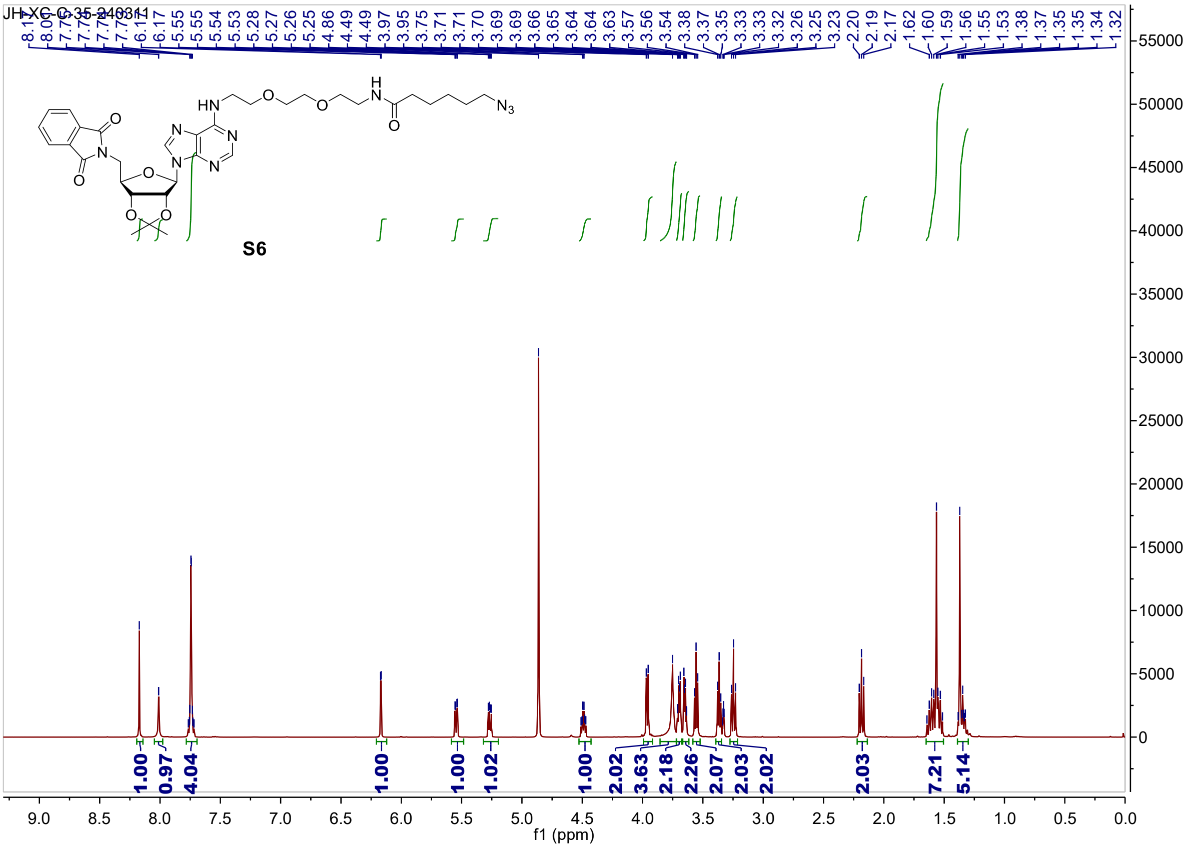

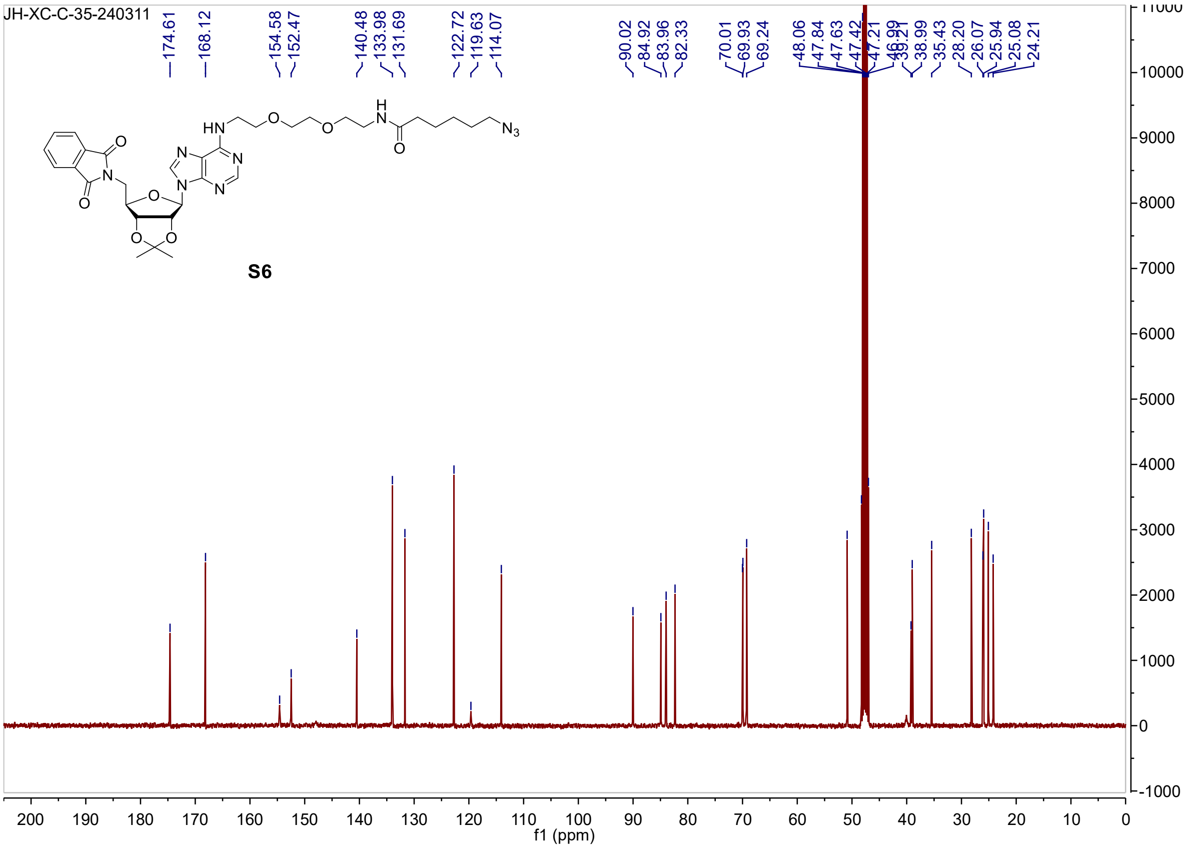

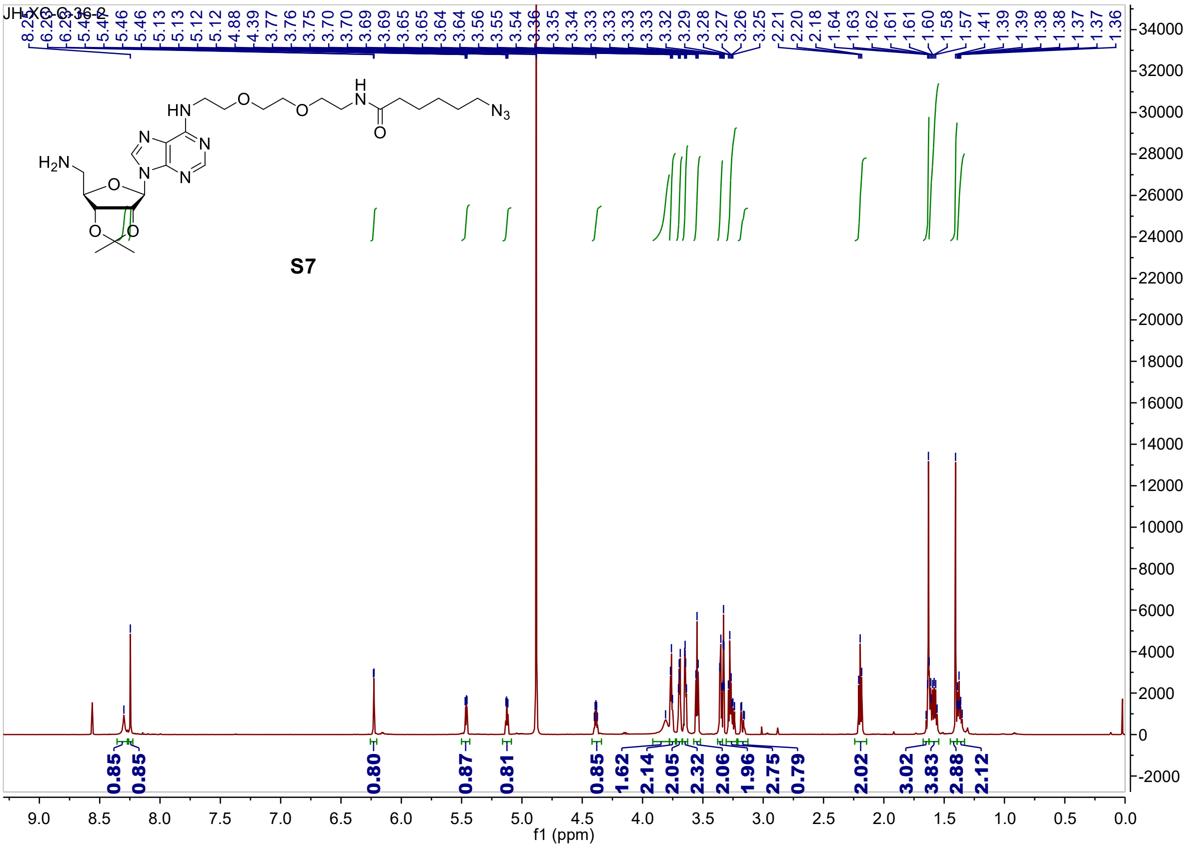

JH-XC-C-36-2
8.25 6.26 6.25 5.47 5.47 5.46 5.46 5.13 5.13 5.12 5.12 4.88 4.39 3.77 3.76 3.75 3.70 3.69 3.69 3.65 3.65 3.64 3.64 3.56 3.55 3.54 3.36 3.35 3.34 3.33 3.33 3.33 3.33 3.33 3.32 3.29 3.28 3.27 3.26 3.25 2.21 2.20 2.18 1.64 1.63 1.62 1.61 1.61 1.60 1.58 1.57 1.41 1.39 1.39 1.38 1.38 1.37 1.37 1.36
S7
0.85 0.85 0.80 0.87 0.81 0.85 1.62 2.14 2.05 2.32 2.06 1.96 2.75 0.79 2.02 3.02 3.83 2.88 2.12
f1 (ppm)

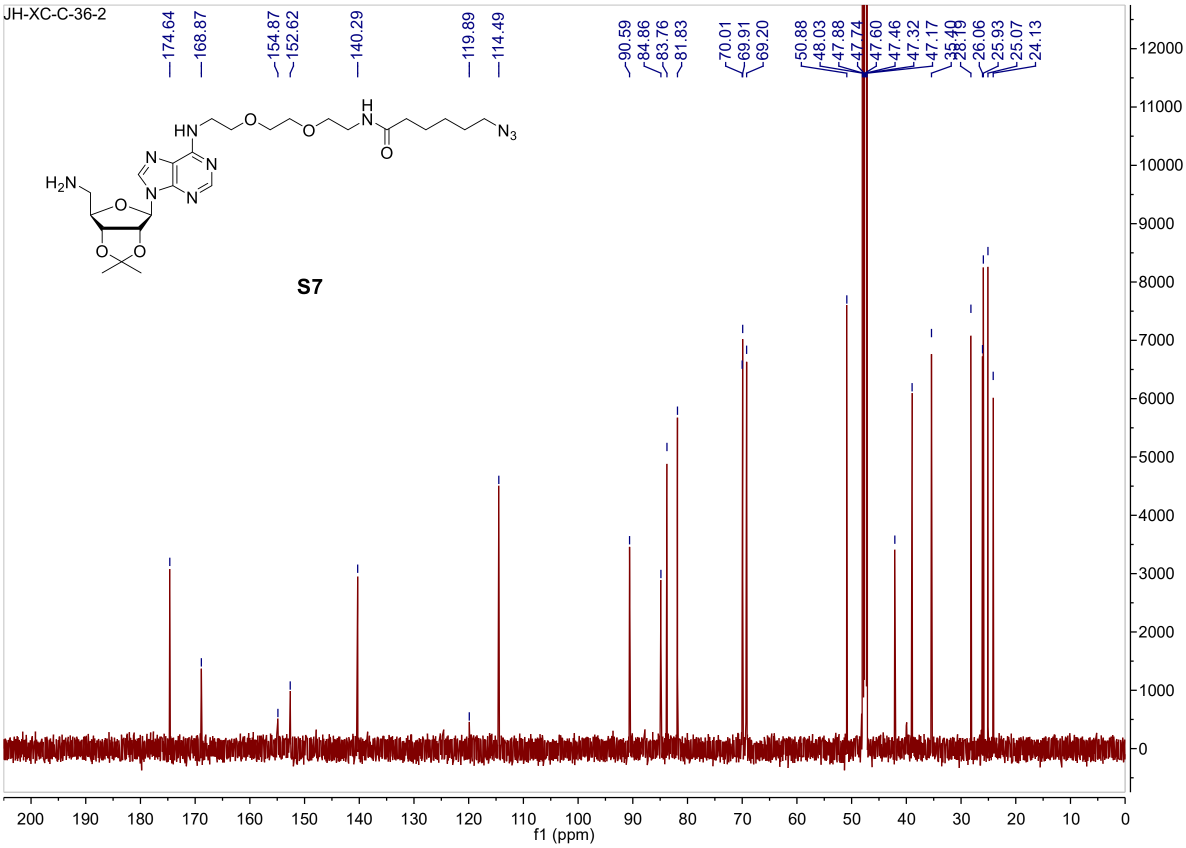

JH-XC-C-36-2
174.64 168.87 154.87 152.62 140.29 119.89 114.49 90.59 84.86 83.76 81.83 70.01 69.91 69.20 50.88 48.03 47.88 47.74 47.60 47.46 47.32 47.17 35.40 28.19 26.06 25.93 25.07 24.13
S7
f1 (ppm)

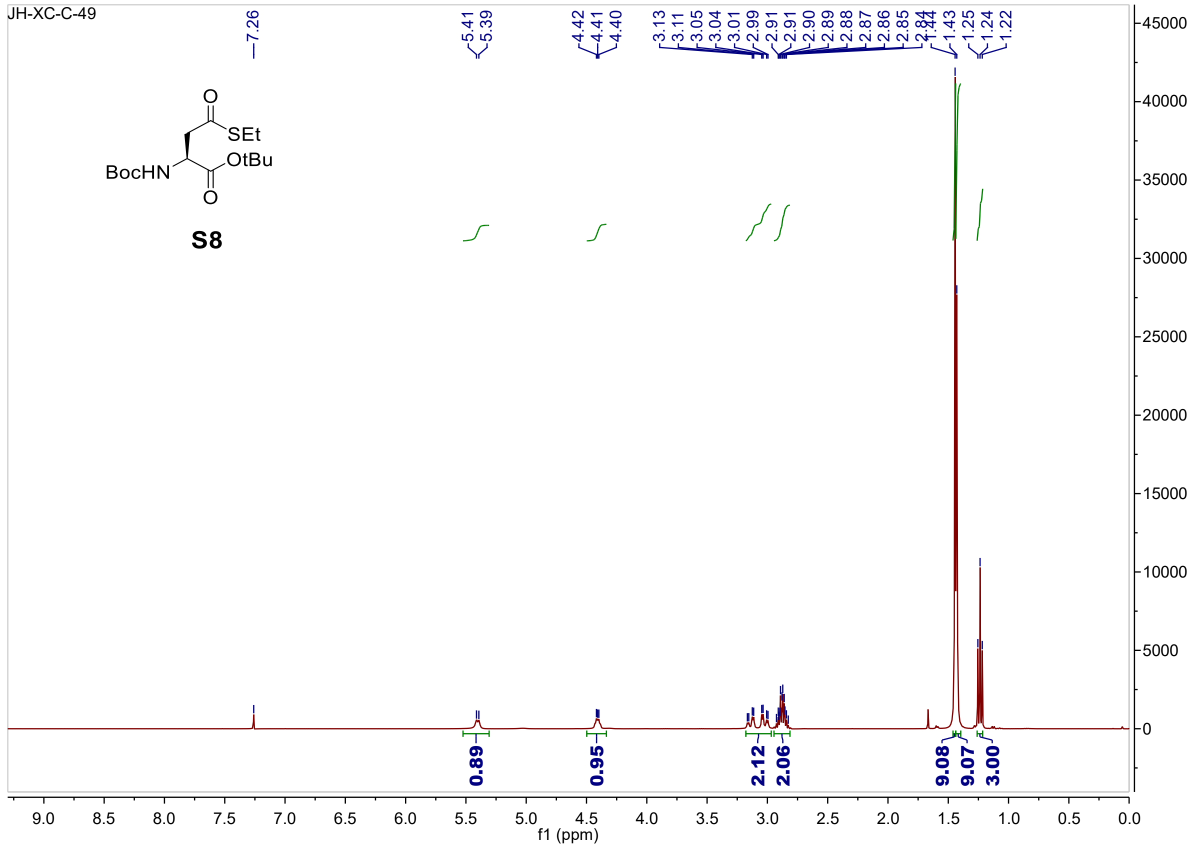

JH-XC-C-49
BocHN
SEt
OtBu
S8
0.89
0.95
2.12
2.06
9.08
9.07
3.00
f1 (ppm)

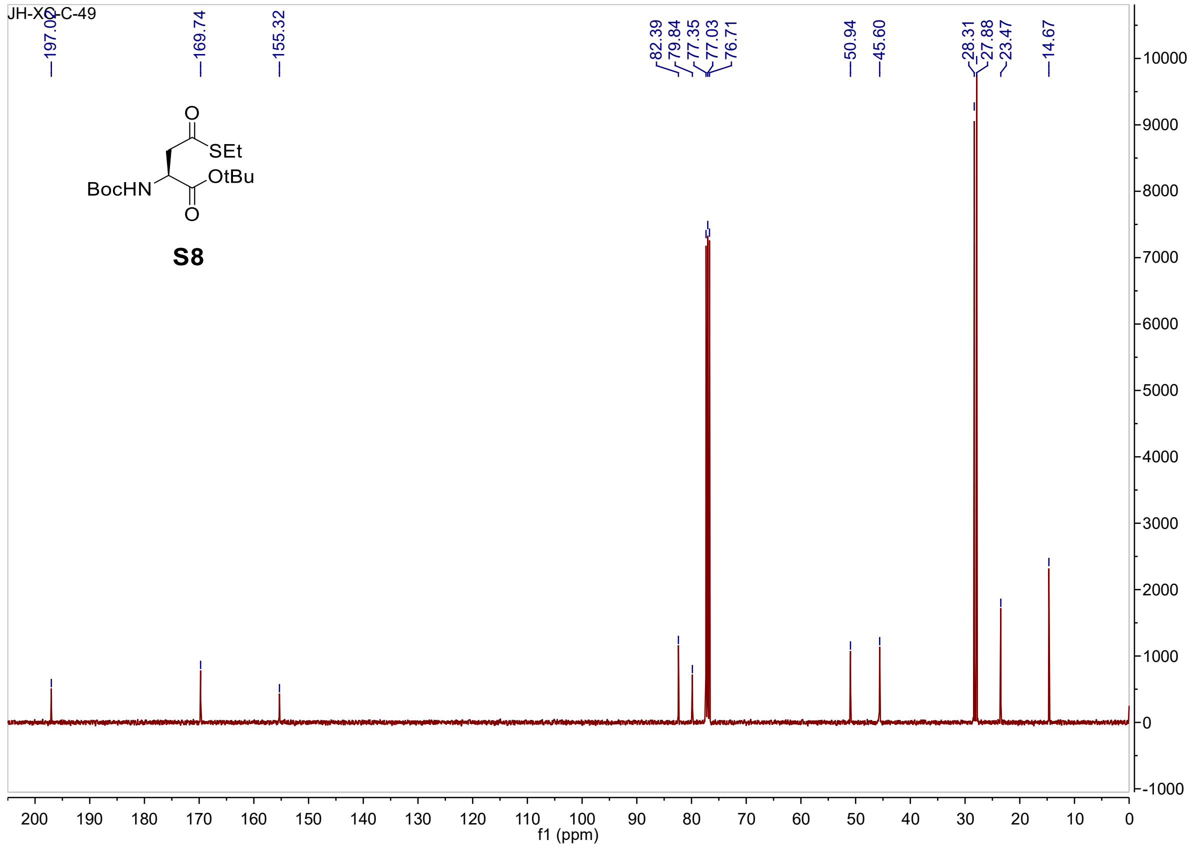

JH-XC-C-49
BocHN
SEt
OtBu
S8
f1 (ppm)

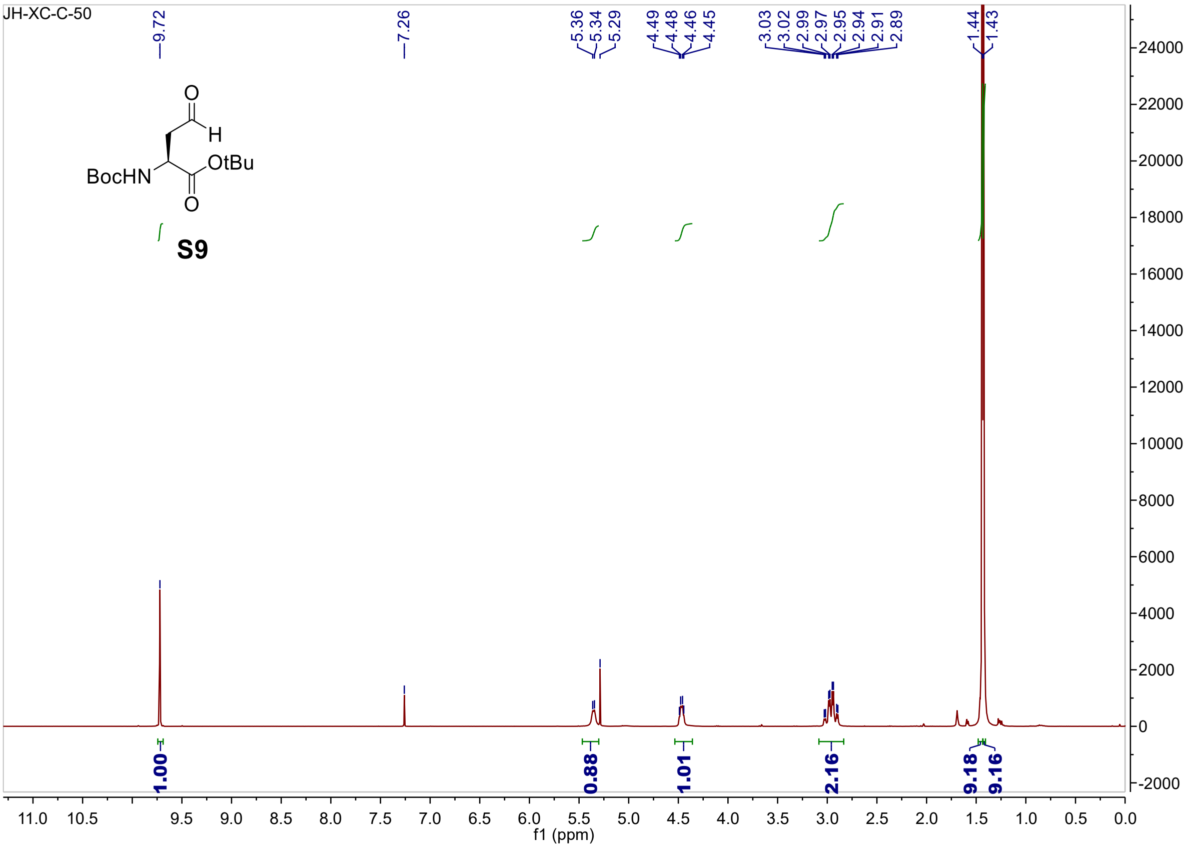
JH-XC-C-50
9.72
7.26
5.36
5.34
5.29
4.49
4.48
4.46
4.45
3.03
3.02
2.99
2.97
2.95
2.94
2.91
2.89
1.44
1.43
O
H
BocHN
OtBu
O
S9
1.00
0.88
1.01
2.16
9.18
9.16
f1 (ppm)

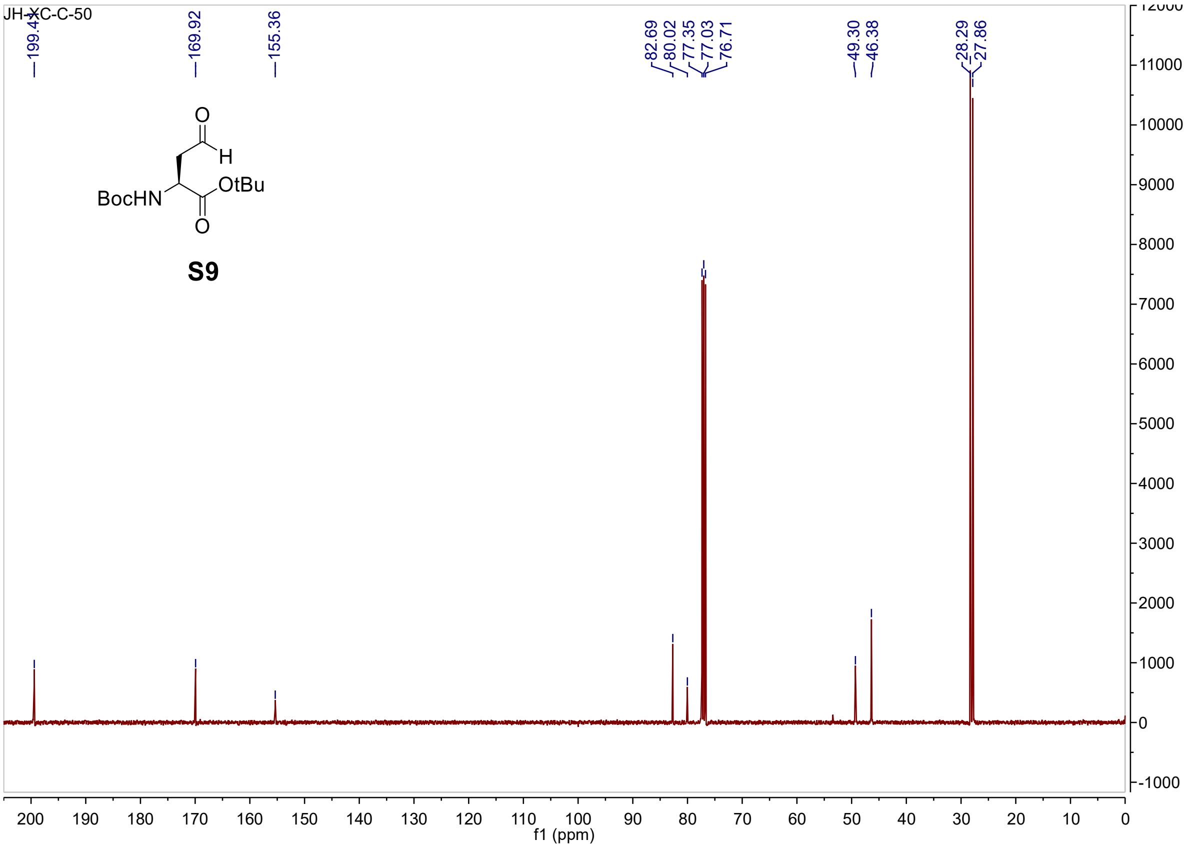
JH-XC-C-50
199.4
169.92
155.36
82.69
80.02
77.35
77.03
76.71
49.30
46.38
28.29
27.86
O
H
BocHN
OtBu
O
S9
f1 (ppm)

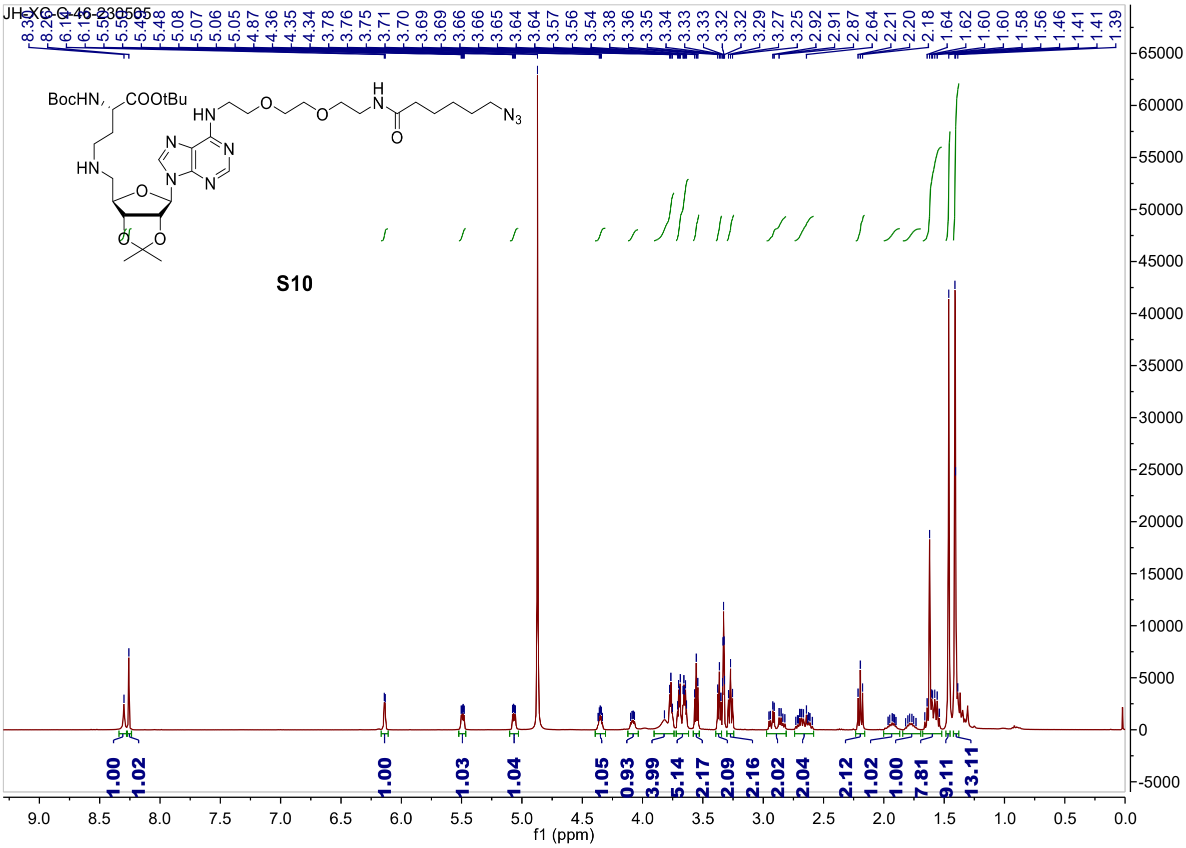

S10

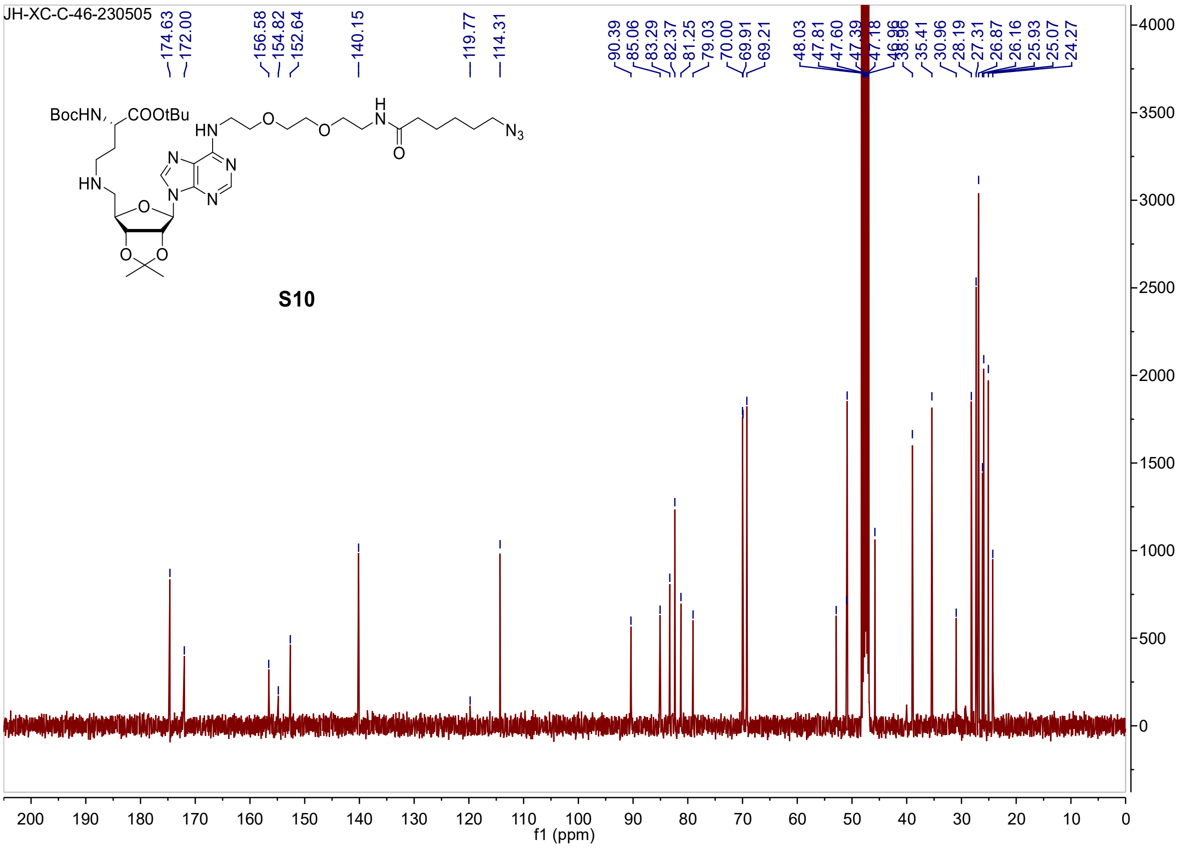

S10

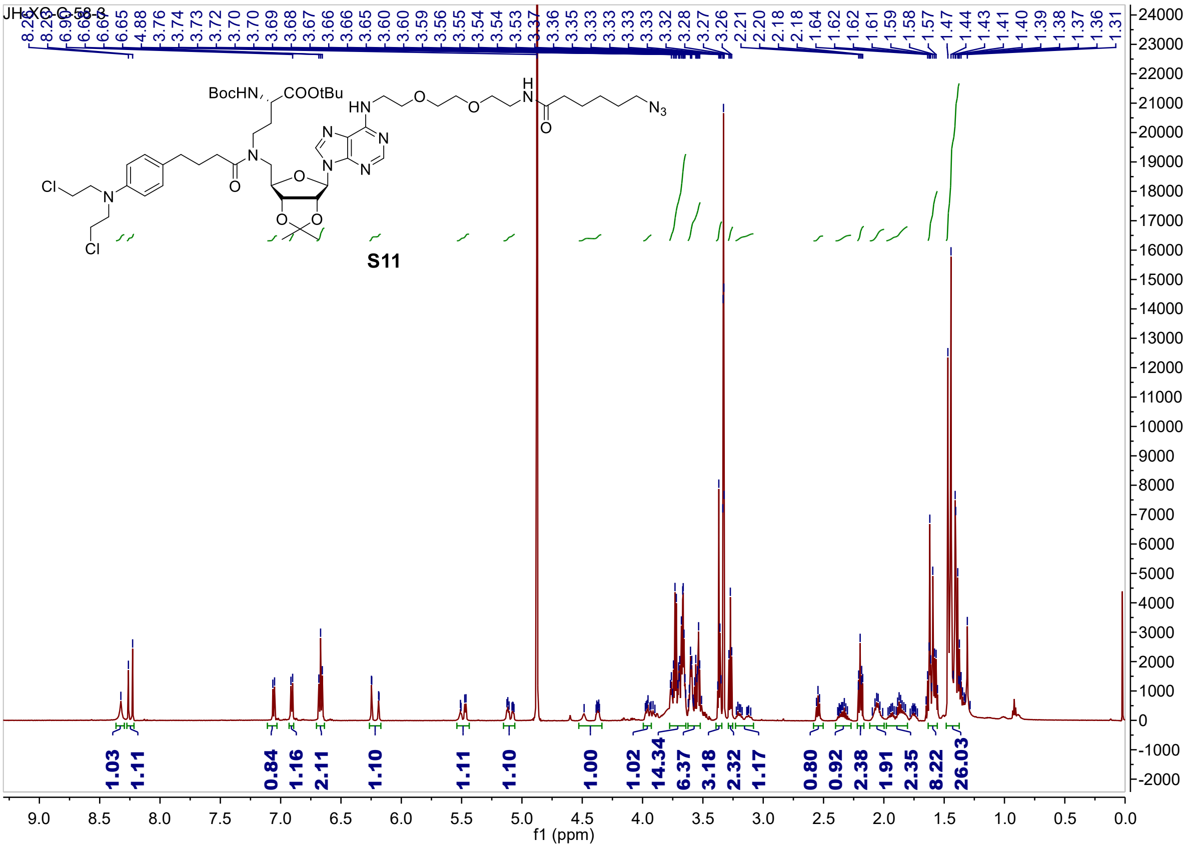
JH-XC-C-58-3
BocHN, COOtBu
HN O O H N O N3
N N N N
O
Cl N O N
O O
Cl
S11
1.03 1.11 0.84 1.16 2.11 1.10 1.11 1.10 1.00 1.02 14.34 6.37 3.18 2.32 1.17 0.80 0.92 2.38 1.91 2.35 8.22 26.03
f1 (ppm)

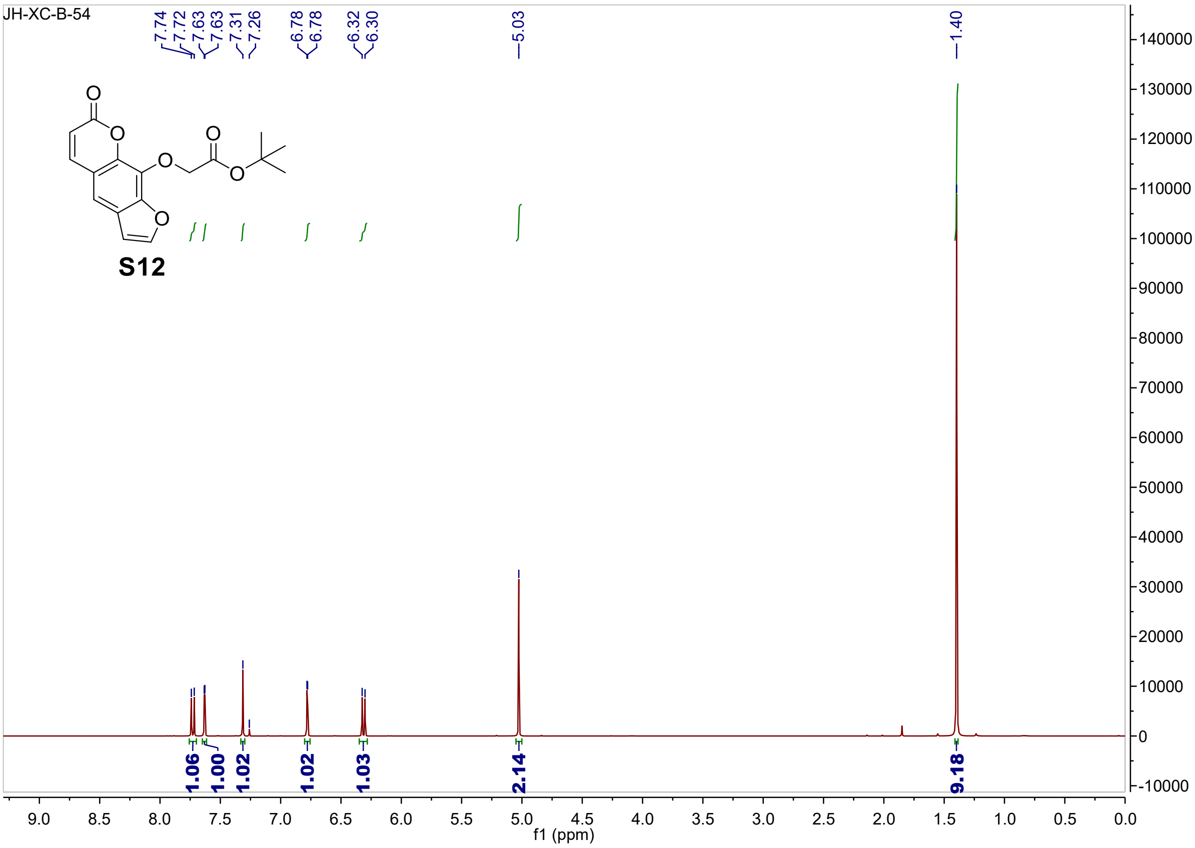
JH-XC-B-54
7.74 7.72 7.63 7.63 7.31 7.26 6.78 6.78 6.32 6.30 5.03 1.40
O O O O O O
S12
1.06 1.00 1.02 1.02 1.03 2.14 9.18
f1 (ppm)

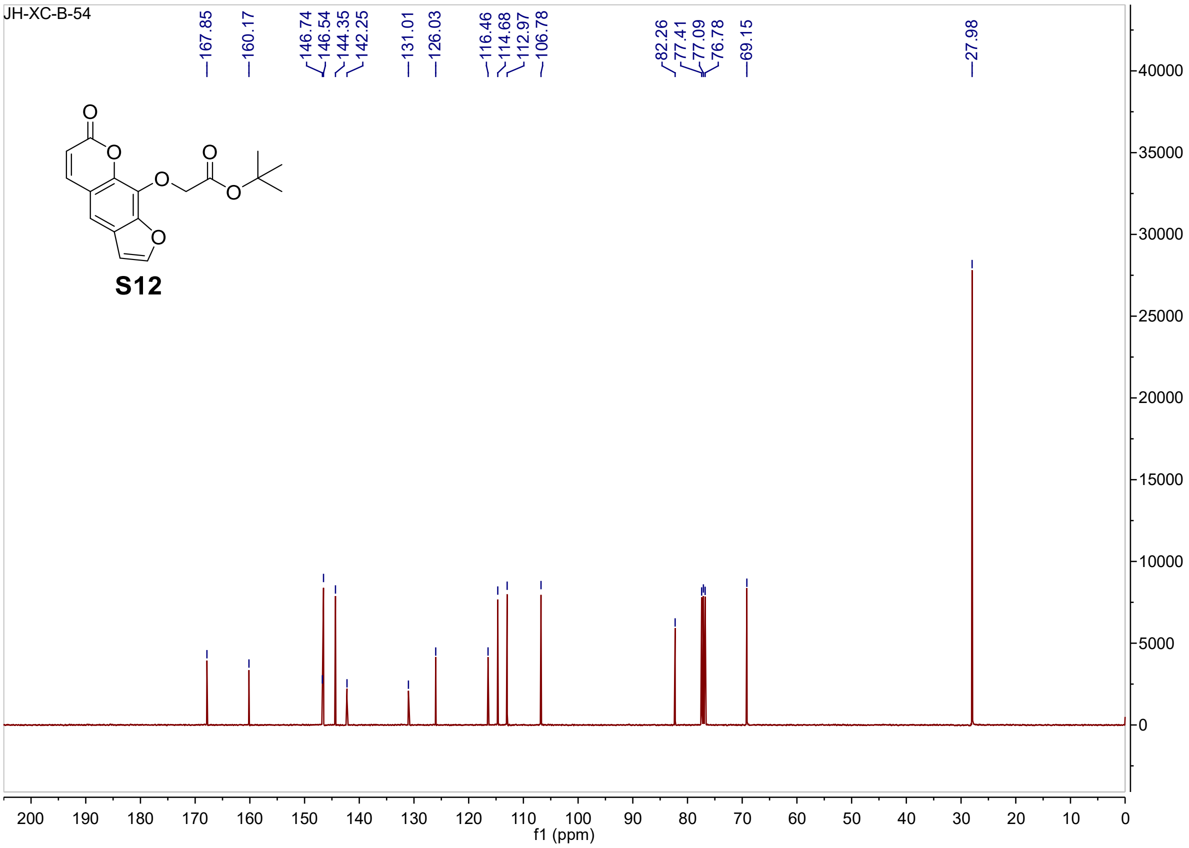
JH-XC-B-54
167.85
160.17
146.74
146.54
144.35
142.25
131.01
126.03
116.46
114.68
112.97
106.78
82.26
77.41
77.09
76.78
69.15
27.98
f1 (ppm)

S12

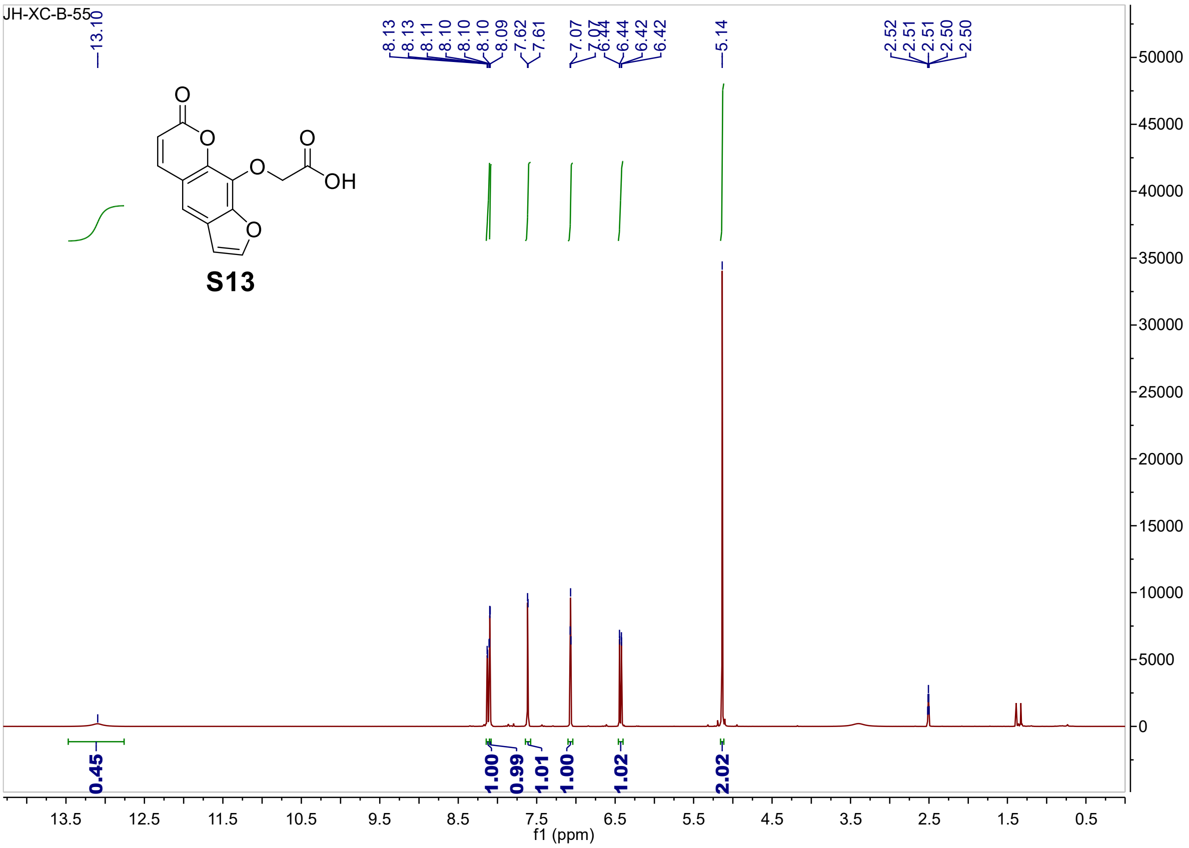
JH-XC-B-55
13.10
8.13
8.13
8.11
8.10
8.10
8.10
8.09
7.62
7.61
7.07
7.07
6.44
6.44
6.42
6.42
5.14
2.52
2.51
2.51
2.50
2.50
0.45
1.00
0.99
1.01
1.00
1.02
2.02
f1 (ppm)

S13

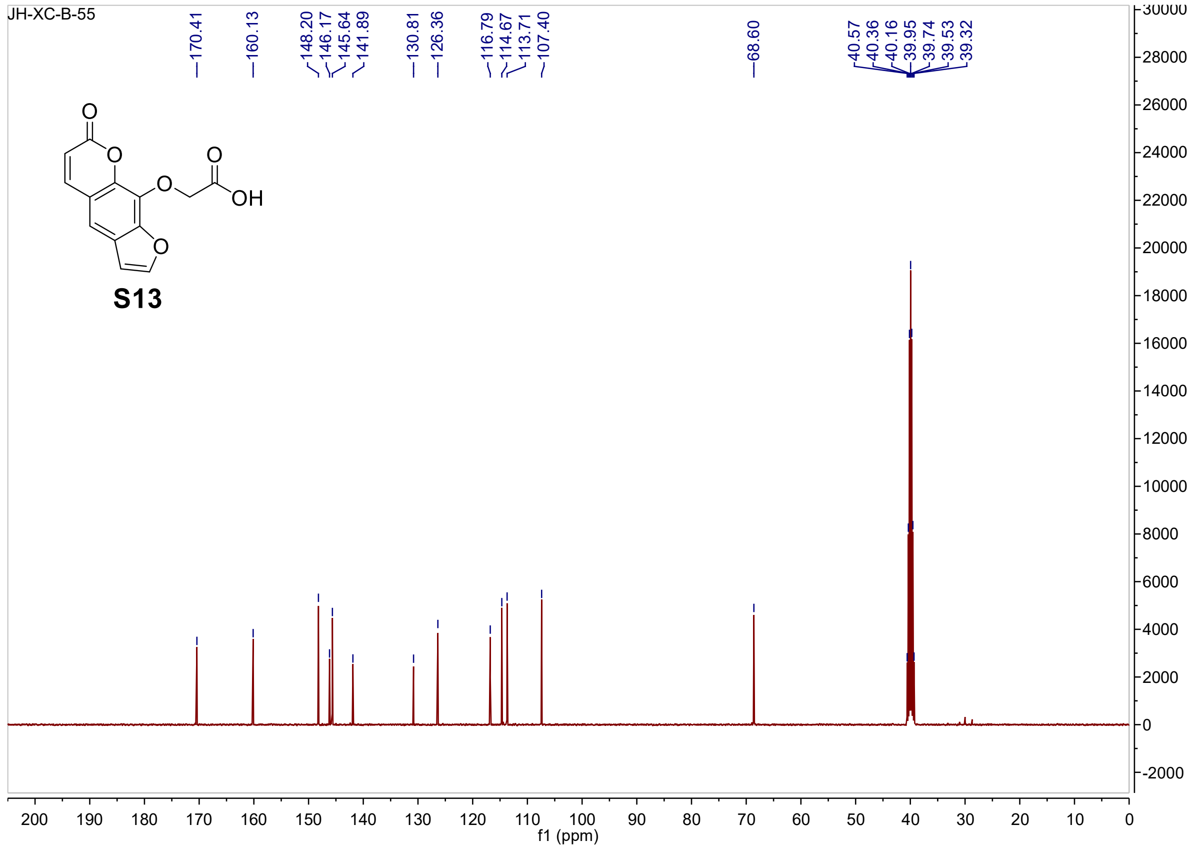
JH-XC-B-55
S13
f1 (ppm)

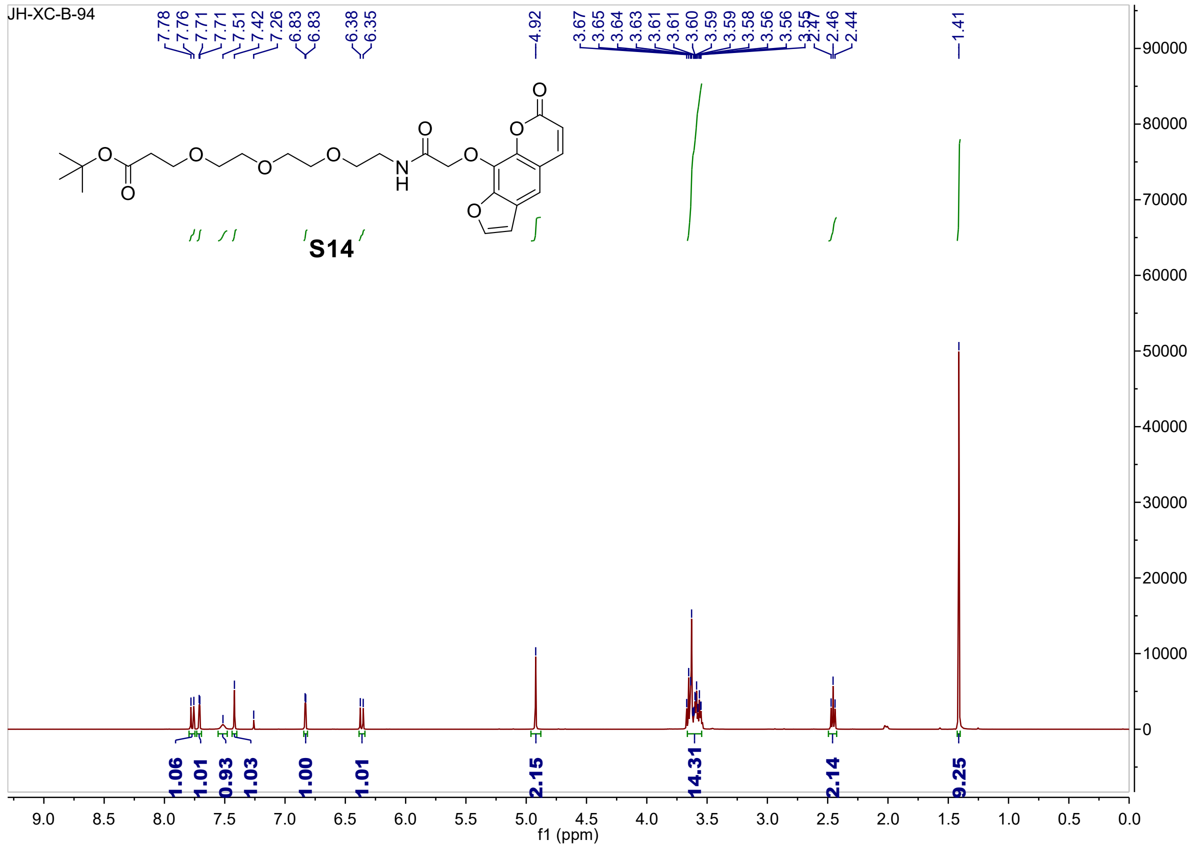
JH-XC-B-94
S14
f1 (ppm)

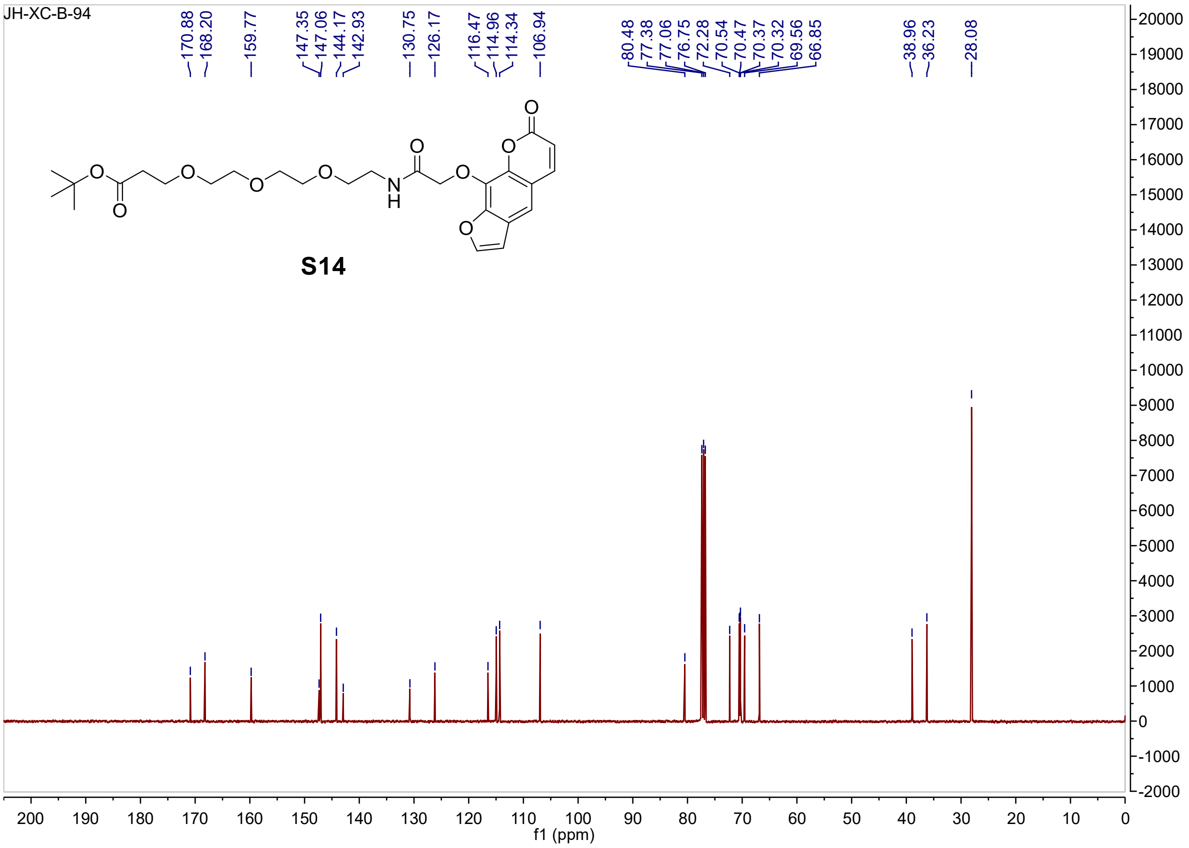
JH-XC-B-94
170.88
168.20
159.77
147.35
147.06
144.17
142.93
130.75
126.17
116.47
114.96
114.34
106.94
80.48
77.38
77.06
76.75
72.28
70.54
70.47
70.37
70.32
69.56
66.85
38.96
36.23
28.08
S14
f1 (ppm)

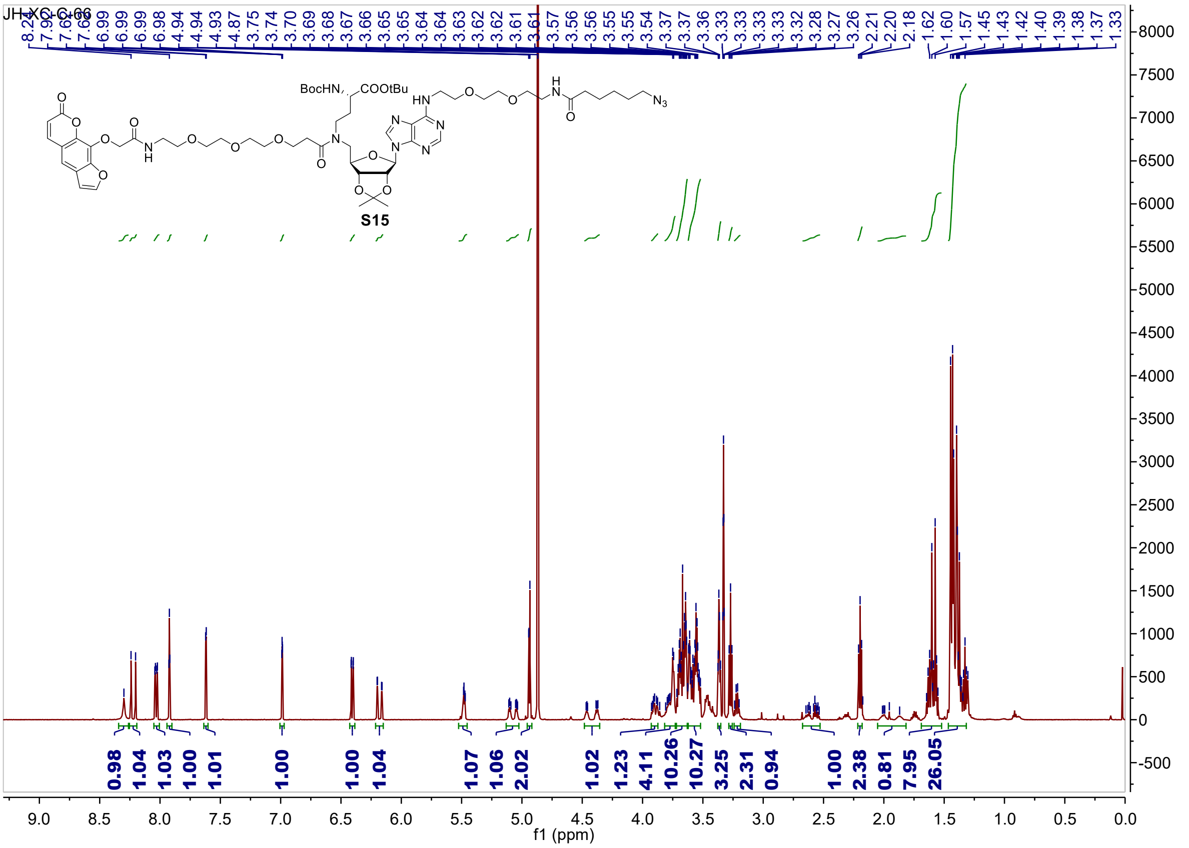
JH-XC-C-66
S15
0.98
1.04
1.03
1.00
1.01
1.00
1.00
1.04
1.07
1.06
2.02
1.02
1.23
4.11
10.26
10.27
3.25
2.31
0.94
1.00
2.38
0.81
7.95
26.05
f1 (ppm)

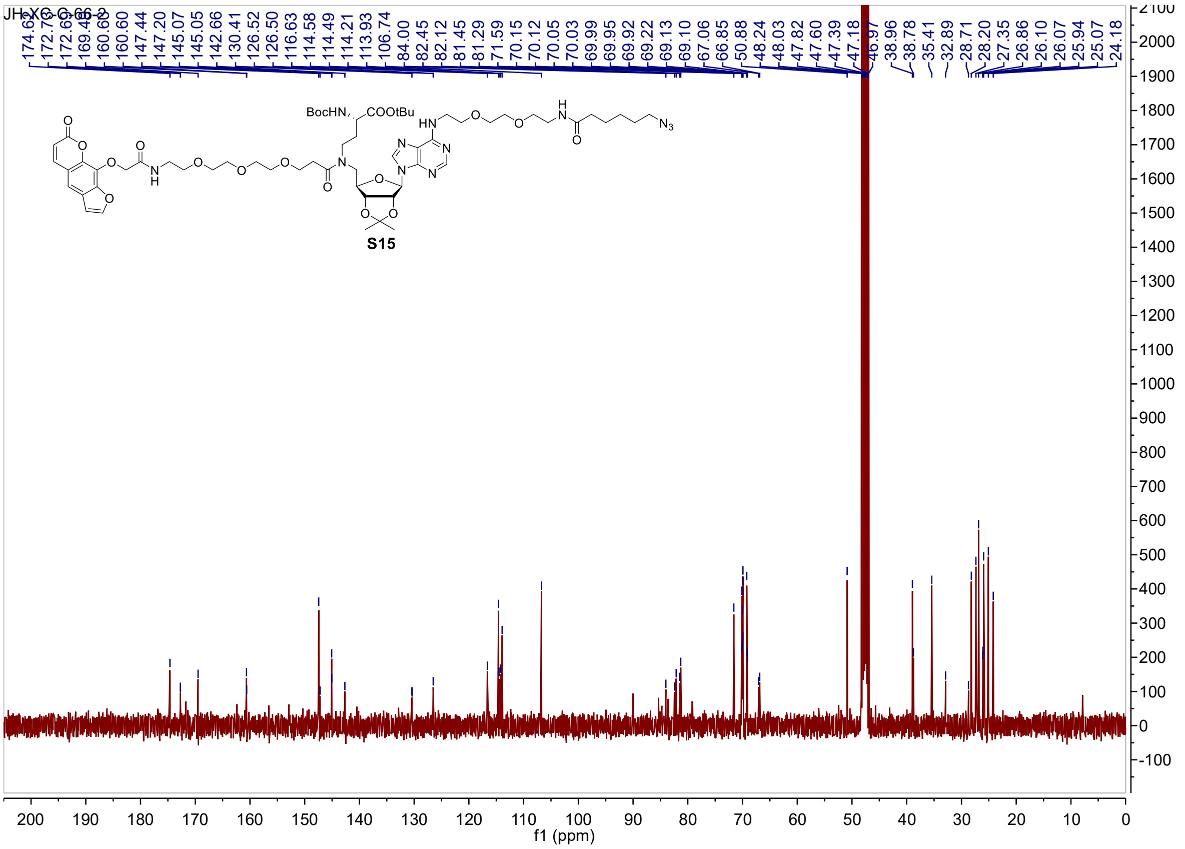
JH-XC-C-66-2
174.63
172.76
172.65
169.48
160.62
160.60
147.44
147.20
145.07
145.05
142.66
130.41
126.52
126.50
116.63
114.58
114.49
114.21
113.93
106.74
84.00
82.45
82.12
81.45
81.29
71.59
70.15
70.12
70.05
70.03
69.99
69.95
69.92
69.22
69.13
69.10
67.06
66.85
50.88
48.24
48.03
47.82
47.60
47.39
47.18
46.97
38.96
38.78
35.41
32.89
28.71
28.20
27.35
26.86
26.10
26.07
25.94
25.07
24.18
BocHN
COOtBu
HN
N3
S15
f1 (ppm)

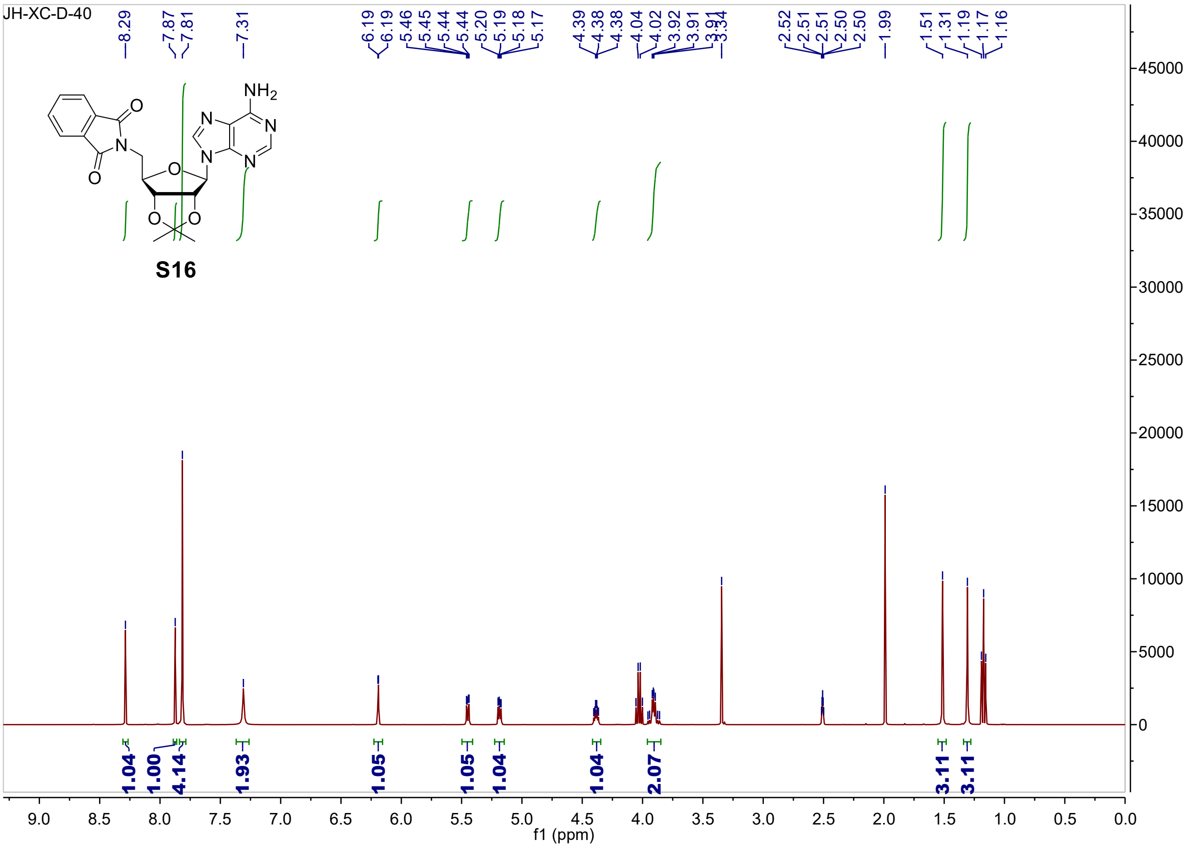
JH-XC-D-40
8.29
7.87
7.81
7.31
6.19
6.19
5.46
5.45
5.44
5.44
5.20
5.19
5.18
5.17
4.39
4.38
4.38
4.04
4.02
3.92
3.91
3.91
3.34
2.52
2.51
2.51
2.50
2.50
1.99
1.51
1.31
1.19
1.17
1.16
NH2
S16
1.04
1.00
4.14
1.93
1.05
1.05
1.04
1.04
2.07
3.11
3.11
f1 (ppm)

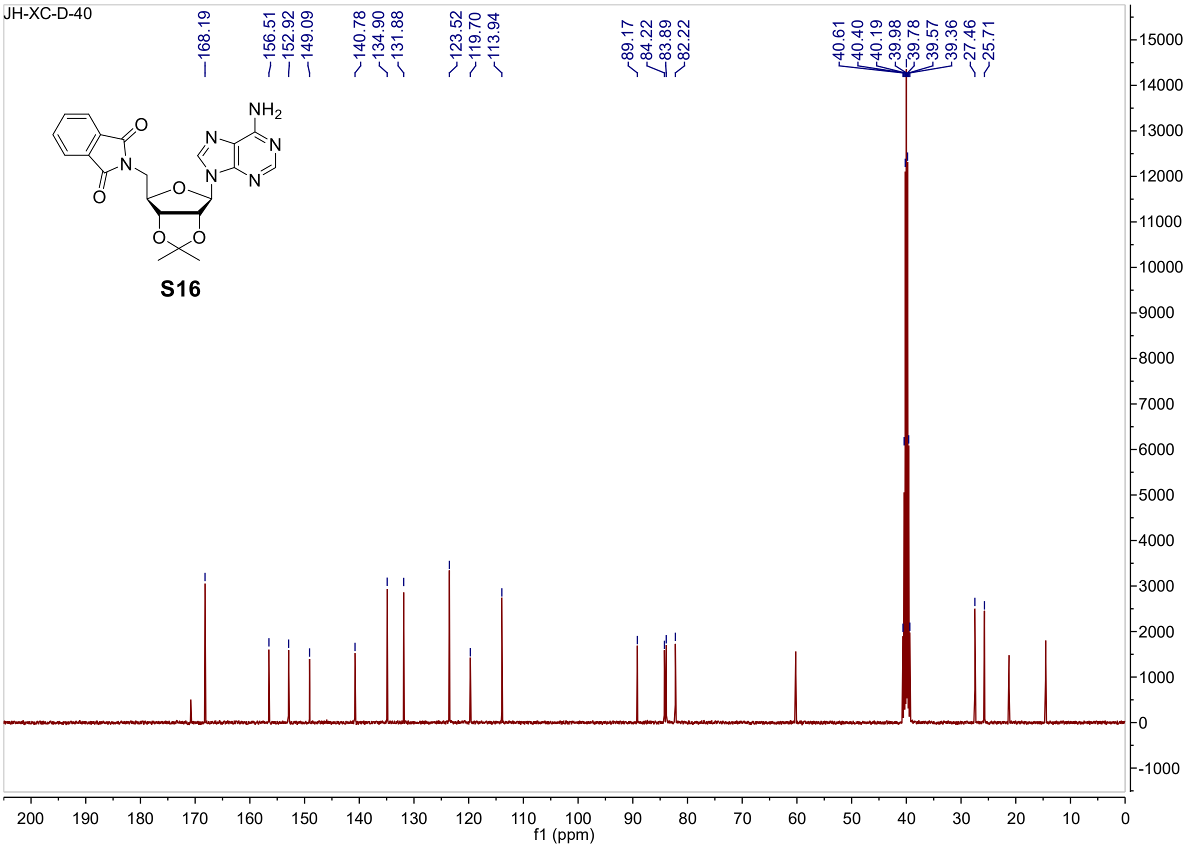
JH-XC-D-40
168.19
156.51
152.92
149.09
140.78
134.90
131.88
123.52
119.70
113.94
89.17
84.22
83.89
82.22
40.61
40.40
40.19
39.98
39.78
39.57
39.36
27.46
25.71
NH_2
S16
f1 (ppm)

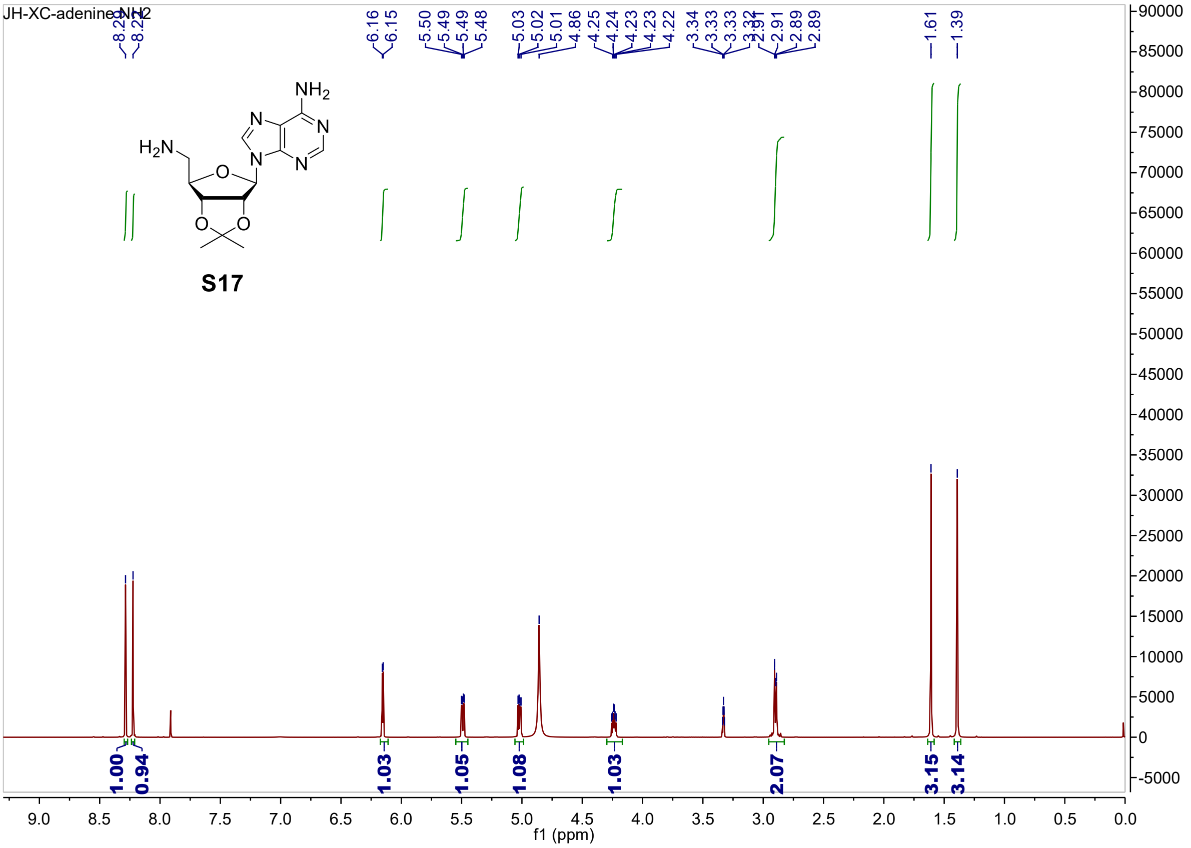
JH-XC-adenine-NH2
6.16
6.15
5.50
5.49
5.49
5.48
5.03
5.02
5.01
4.86
4.25
4.24
4.23
4.23
4.22
3.34
3.33
3.33
2.91
2.89
2.89
1.61
1.39
NH_2
H_2N
S17
1.00
0.94
1.03
1.05
1.08
1.03
2.07
3.15
3.14
f1 (ppm)

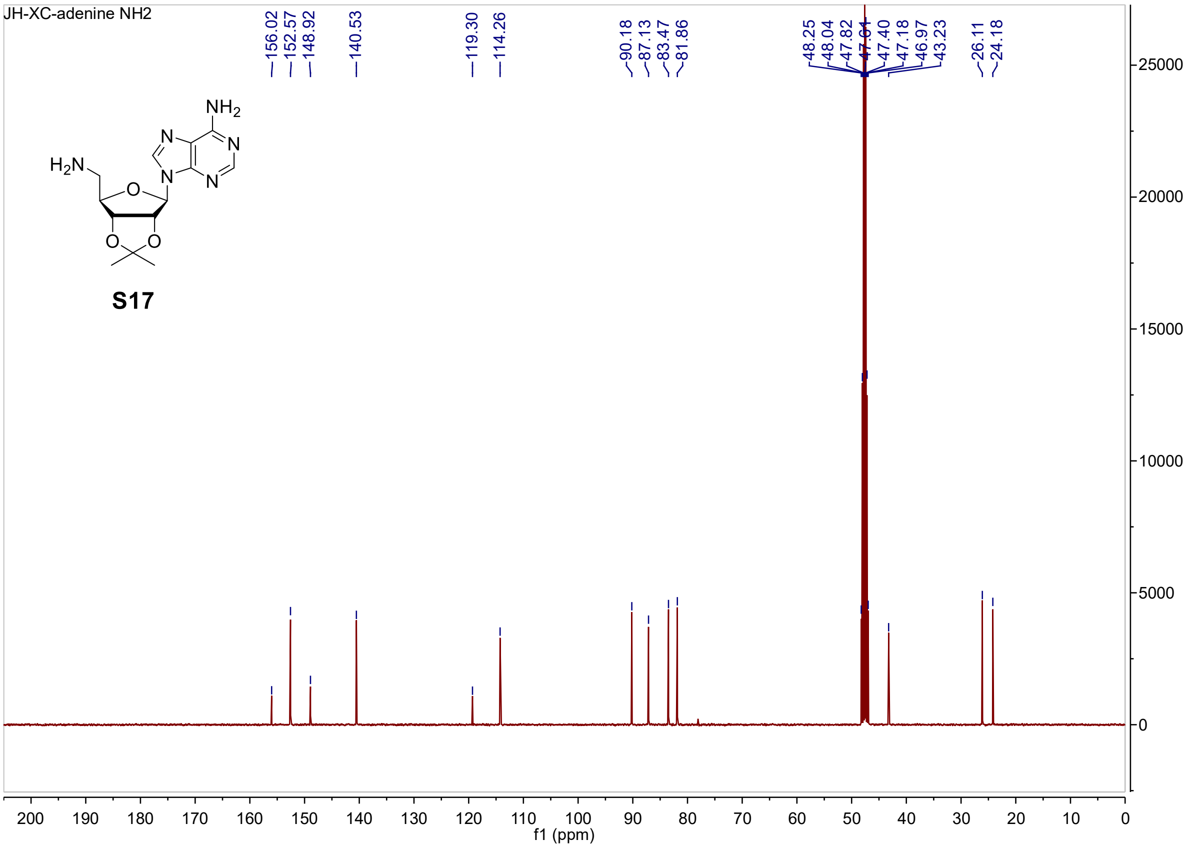
JH-XC-adenine NH2
156.02
152.57
148.92
140.53
119.30
114.26
90.18
87.13
83.47
81.86
48.25
48.04
47.82
47.61
47.40
47.18
46.97
43.23
26.11
24.18
S17
f1 (ppm)

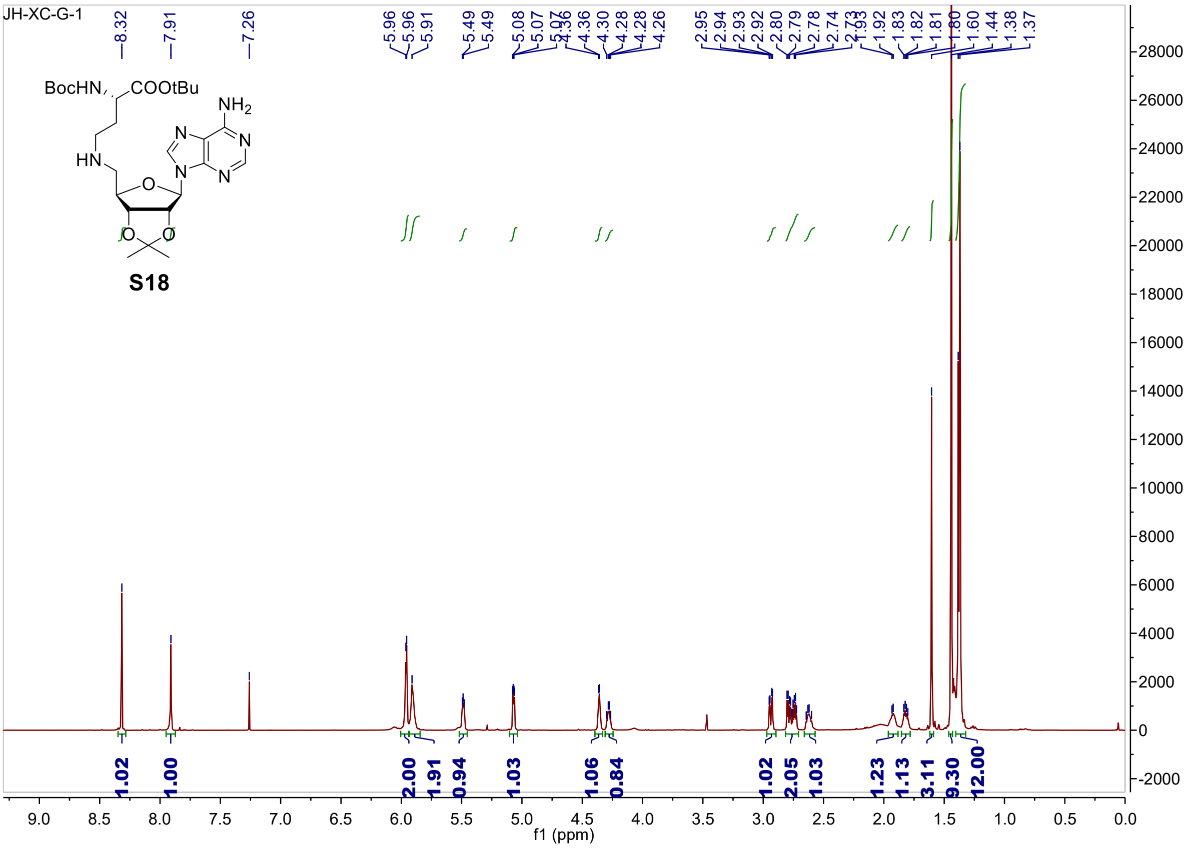
JH-XC-G-1
8.32
7.91
7.26
5.96
5.96
5.91
5.49
5.49
5.08
5.07
5.06
4.36
4.30
4.28
4.26
2.95
2.94
2.93
2.92
2.80
2.79
2.78
2.74
2.73
1.93
1.92
1.83
1.82
1.81
1.80
1.60
1.44
1.38
1.37
BocHN
COOtBu
S18
1.02
1.00
2.00
1.91
0.94
1.03
1.06
0.84
1.02
2.05
1.03
1.23
1.13
3.11
9.30
12.00
f1 (ppm)

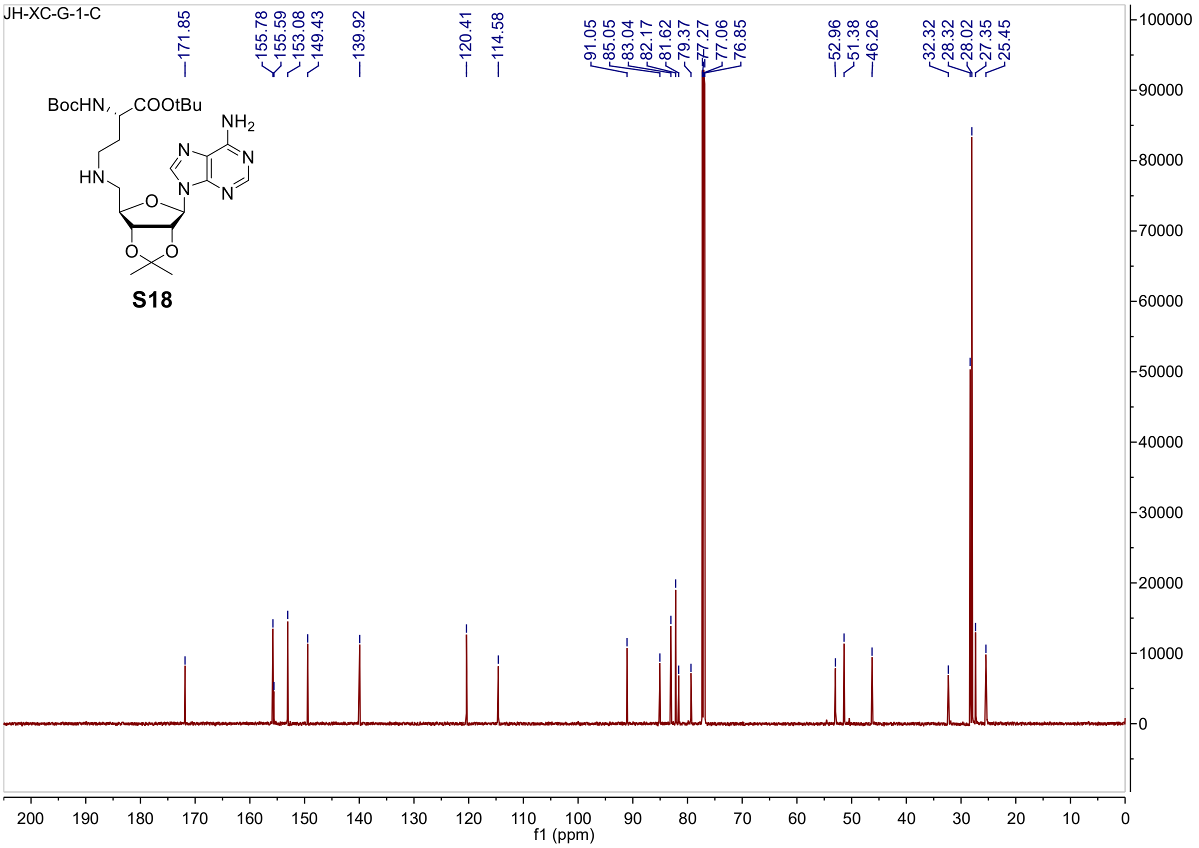
JH-XC-G-1-C
171.85
155.78
155.59
153.08
149.43
139.92
120.41
114.58
91.05
85.05
83.04
82.17
81.62
79.37
77.27
77.06
76.85
52.96
51.38
46.26
32.32
28.32
28.02
27.35
25.45
BocHN
COOtBu
NH_2
HN
S18
f1 (ppm)

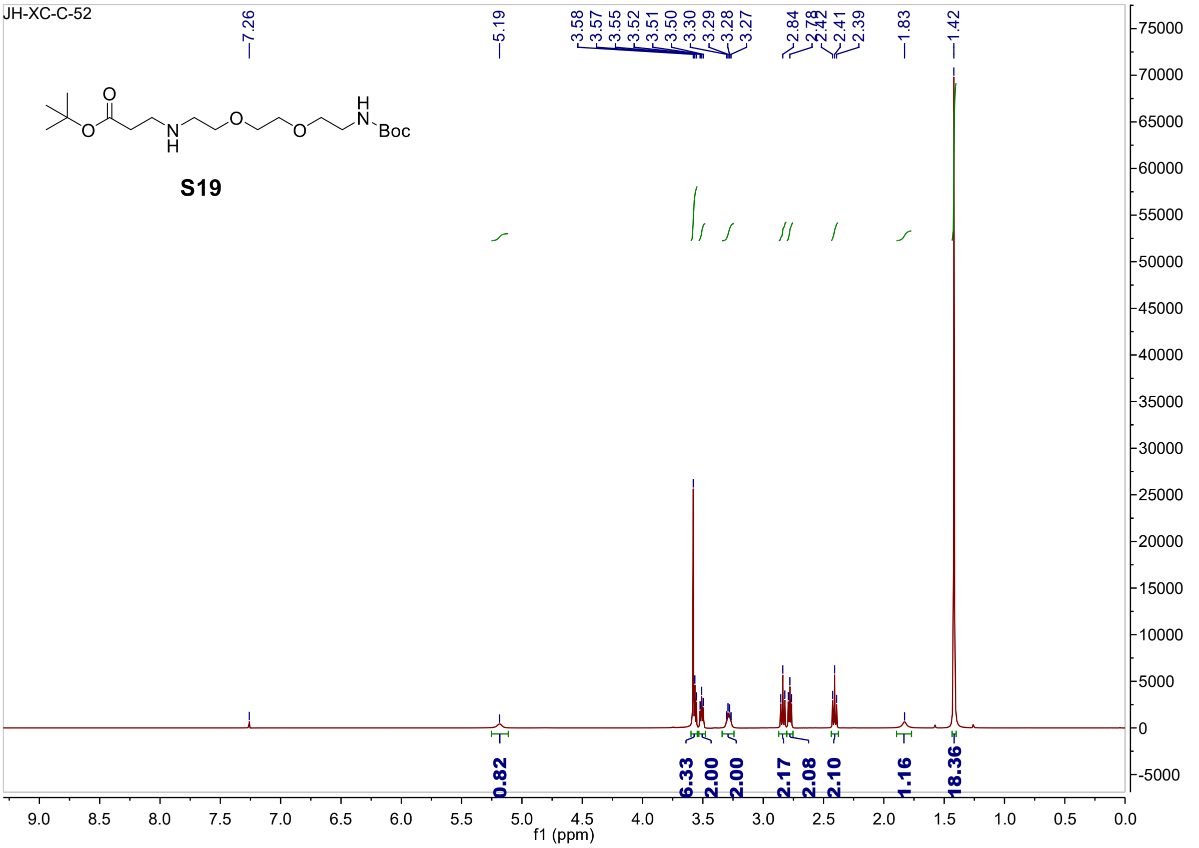
JH-XC-C-52
7.26
5.19
3.58
3.57
3.55
3.52
3.51
3.50
3.30
3.29
3.28
3.27
2.84
2.42
2.41
2.39
1.83
1.42
Boc
S19
0.82
6.33
2.00
2.00
2.17
2.08
2.10
1.16
18.36
f1 (ppm)

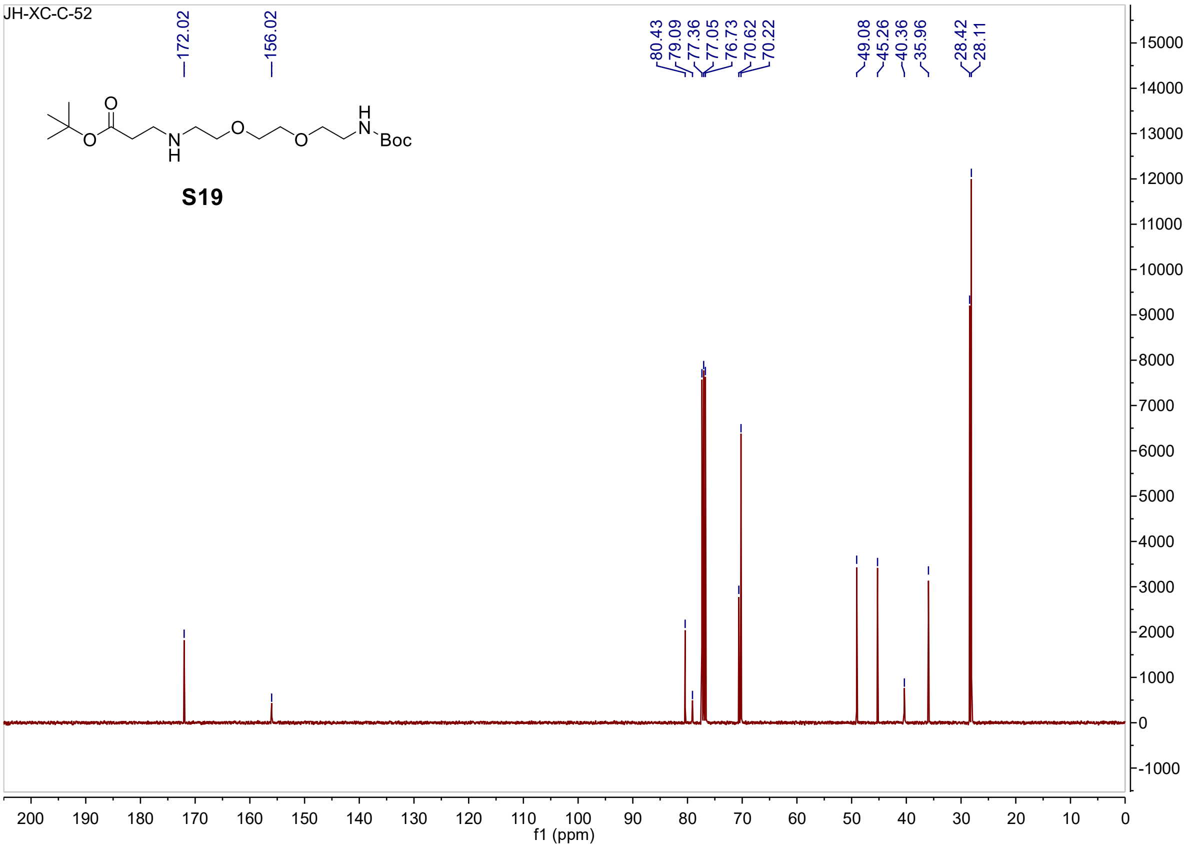
JH-XC-C-52
S19
Boc
172.02
156.02
80.43
79.09
77.36
77.05
76.73
70.62
70.22
49.08
45.26
40.36
35.96
28.42
28.11
f1 (ppm)

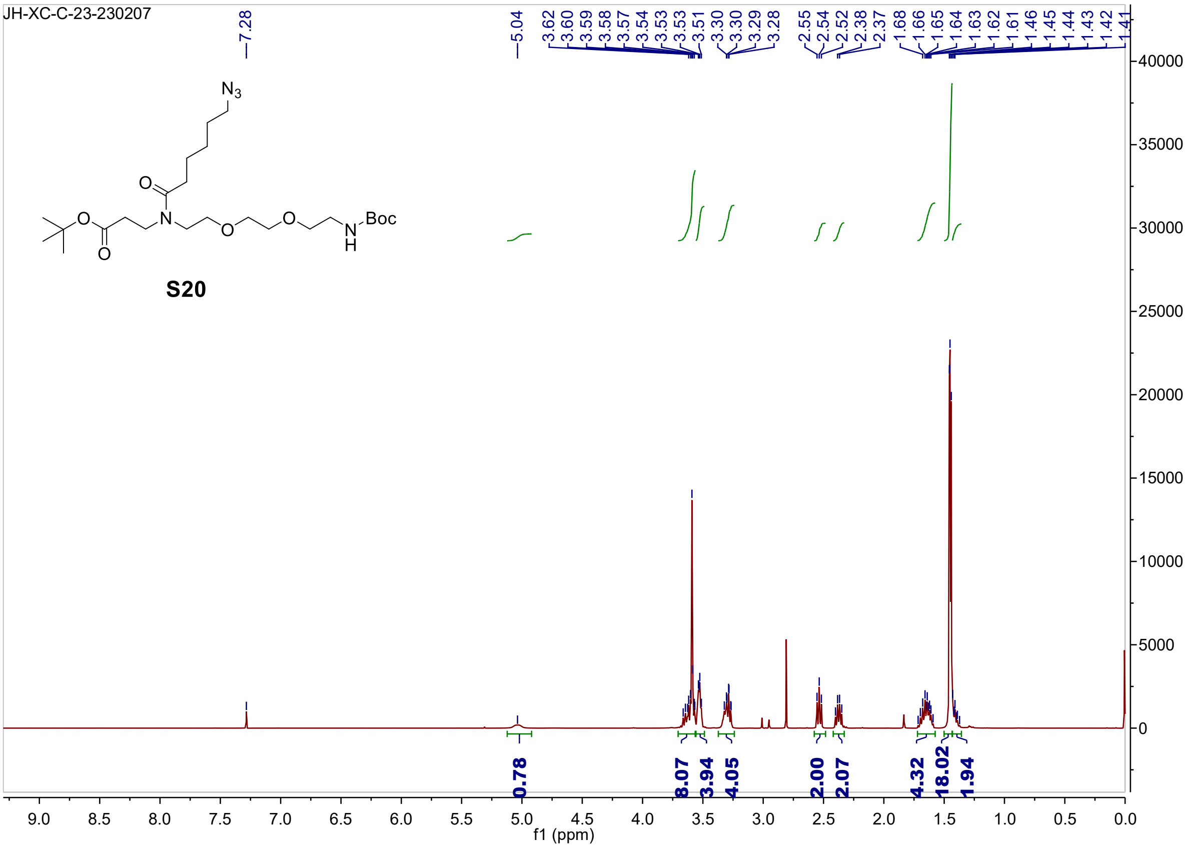
JH-XC-C-23-230207
S20
Boc
7.28
5.04
3.62
3.60
3.59
3.58
3.57
3.54
3.53
3.53
3.51
3.30
3.30
3.29
3.28
2.55
2.54
2.52
2.38
2.37
1.68
1.66
1.65
1.64
1.63
1.62
1.61
1.46
1.45
1.44
1.43
1.42
1.41
0.78
8.07
3.94
4.05
2.00
2.07
4.32
18.02
1.94
f1 (ppm)

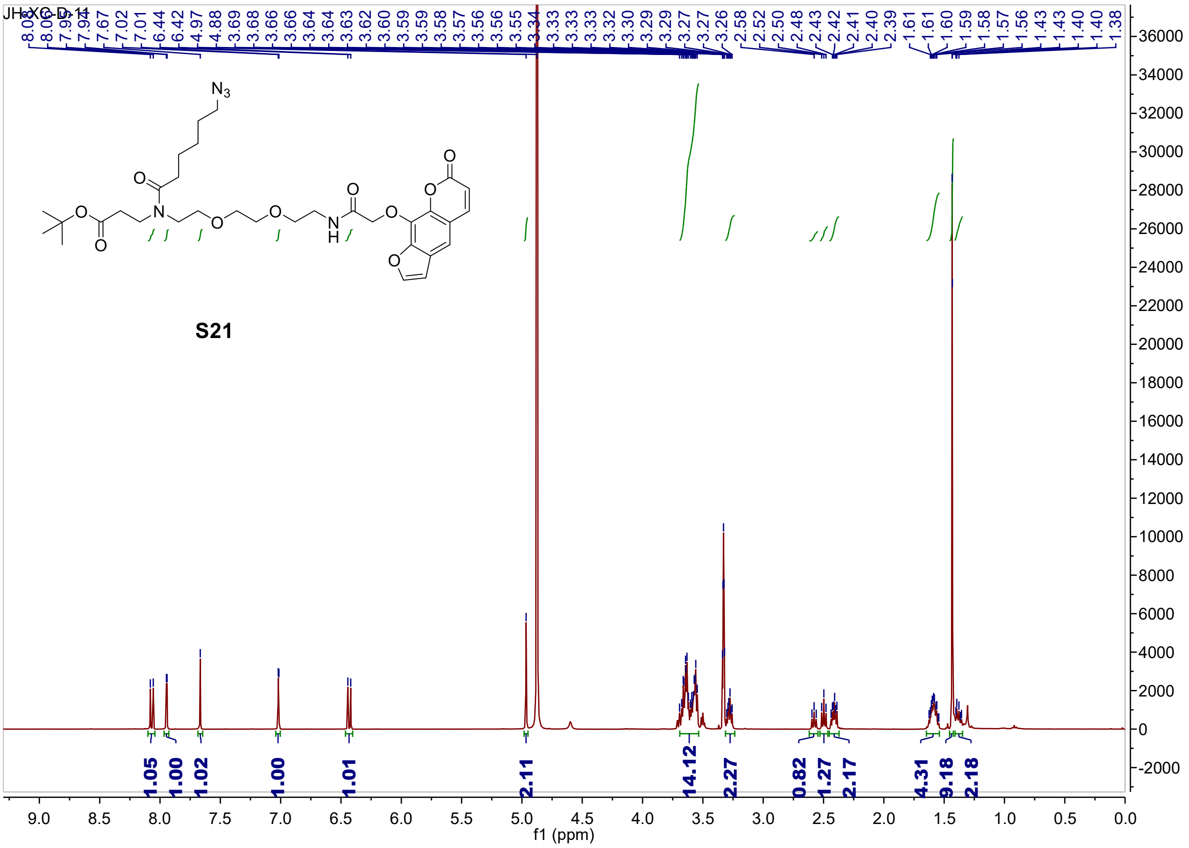

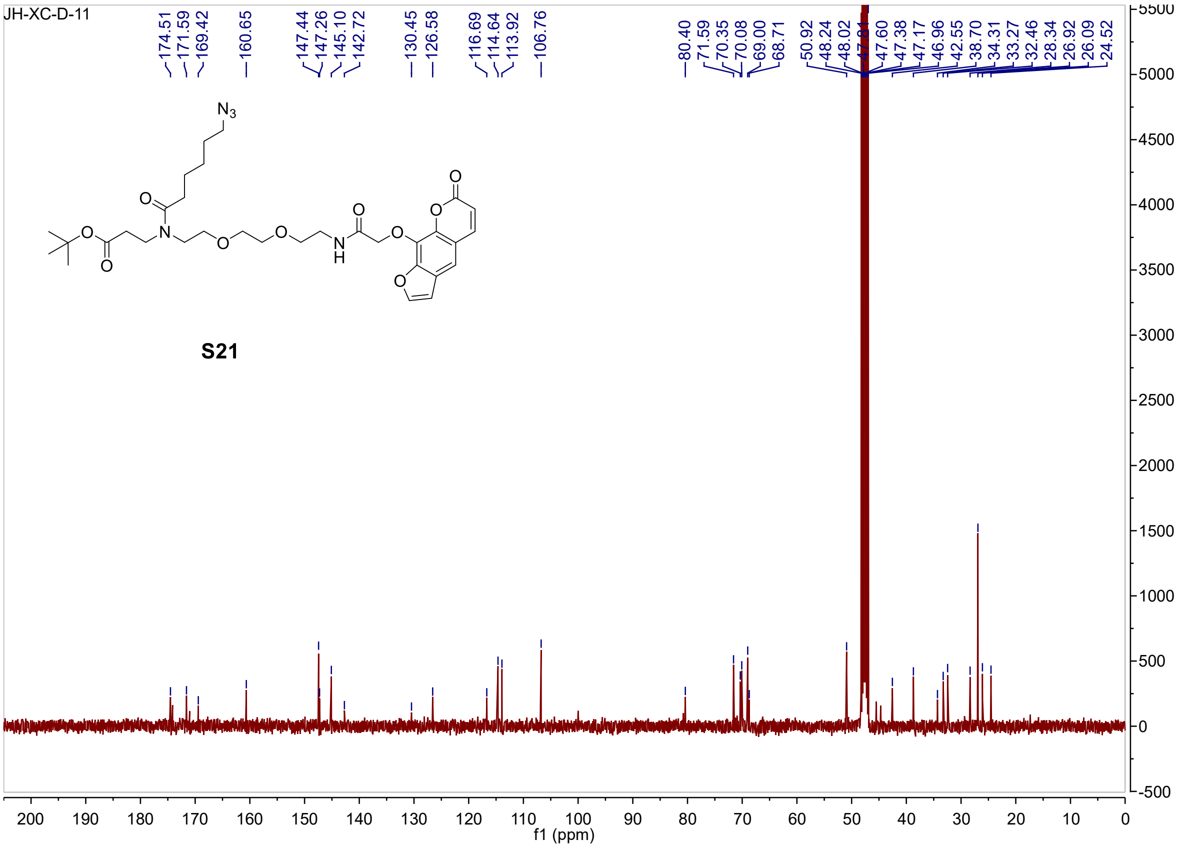

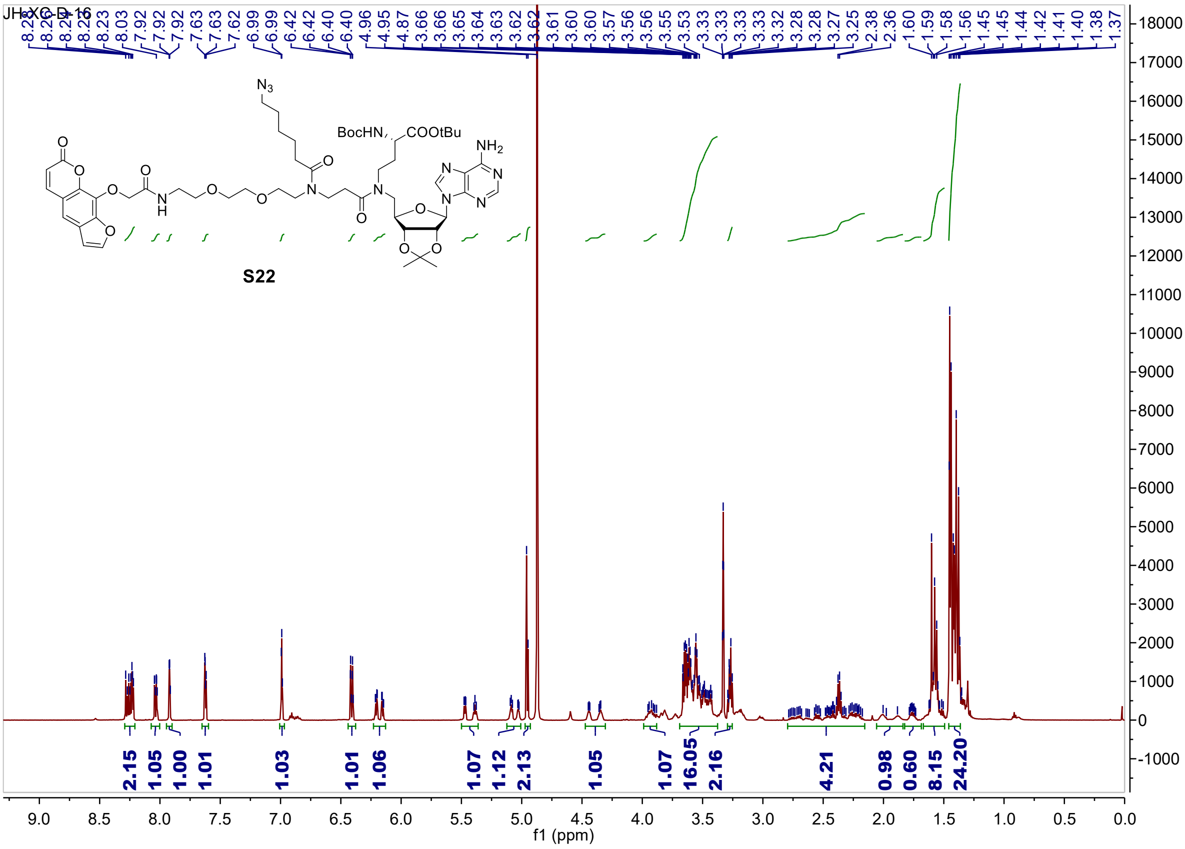
JH-XC-D-16
S22
f1 (ppm)

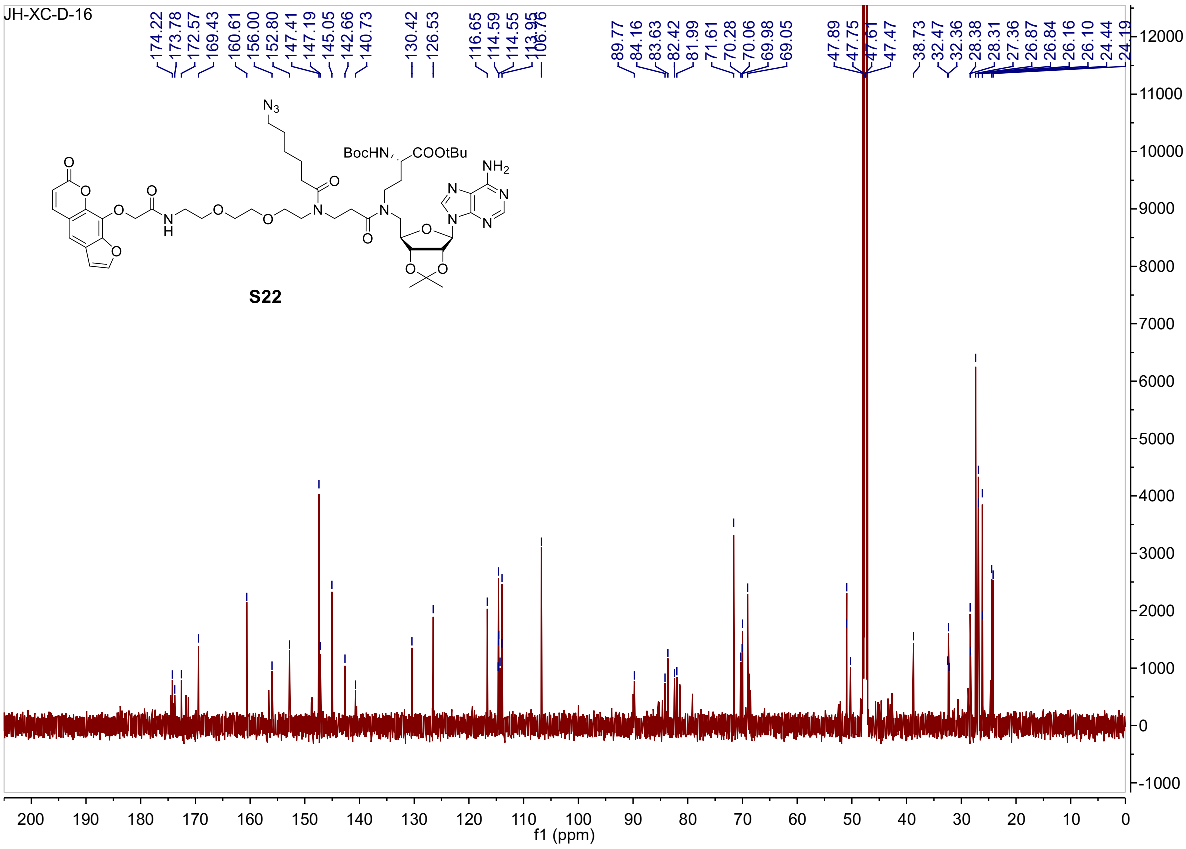
JH-XC-D-16
S22
f1 (ppm)

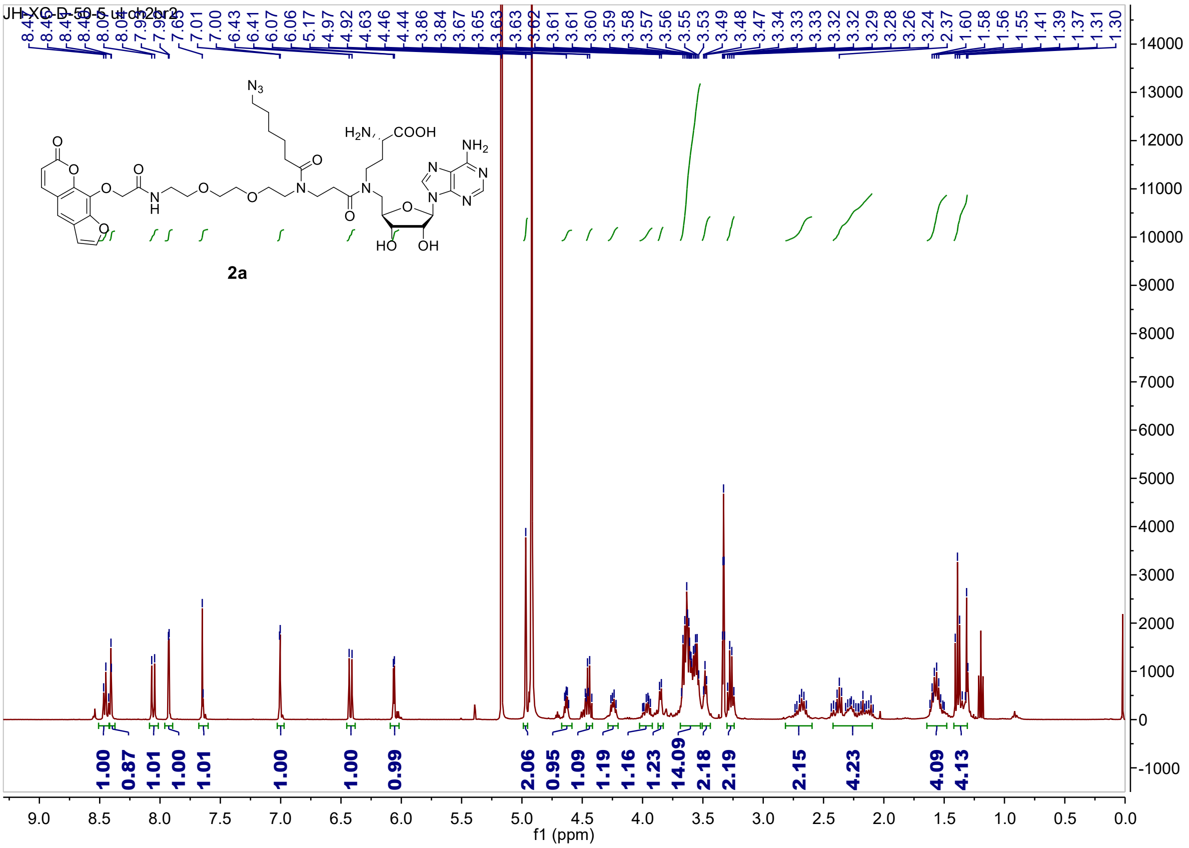

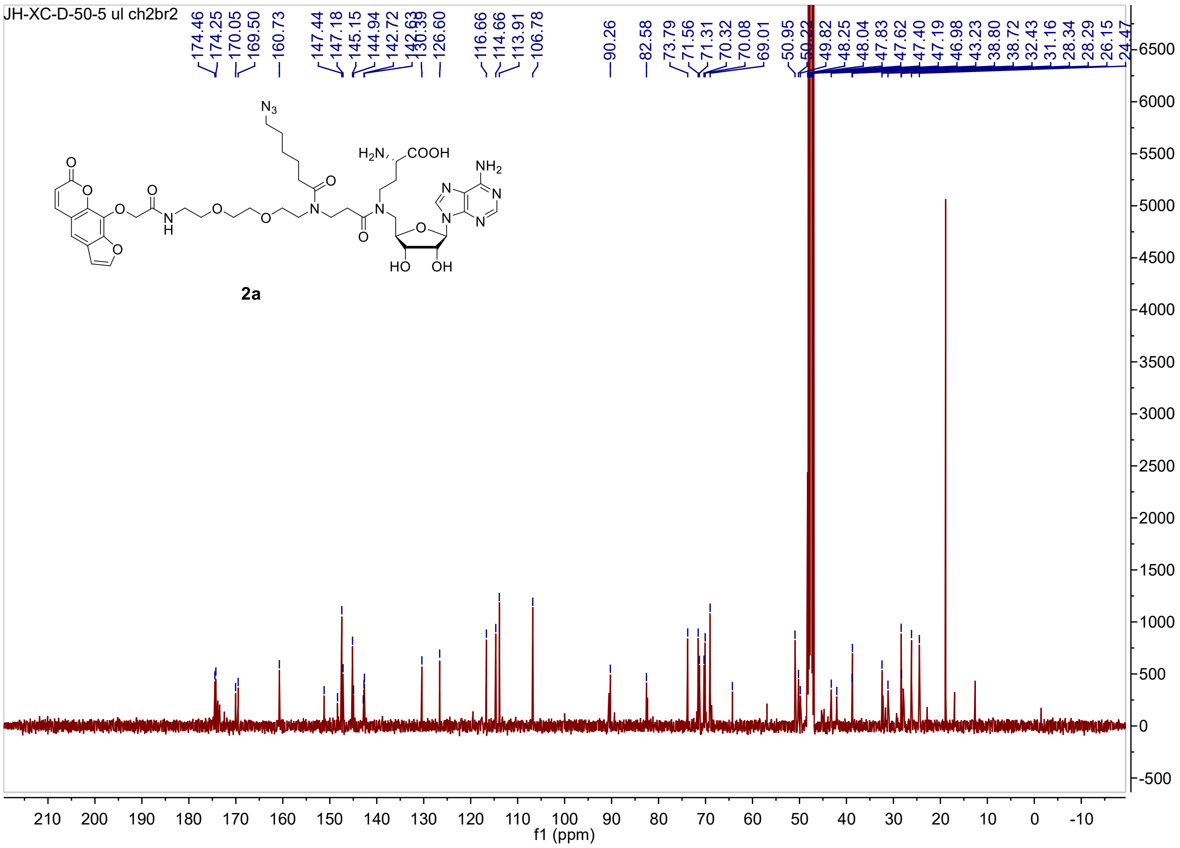

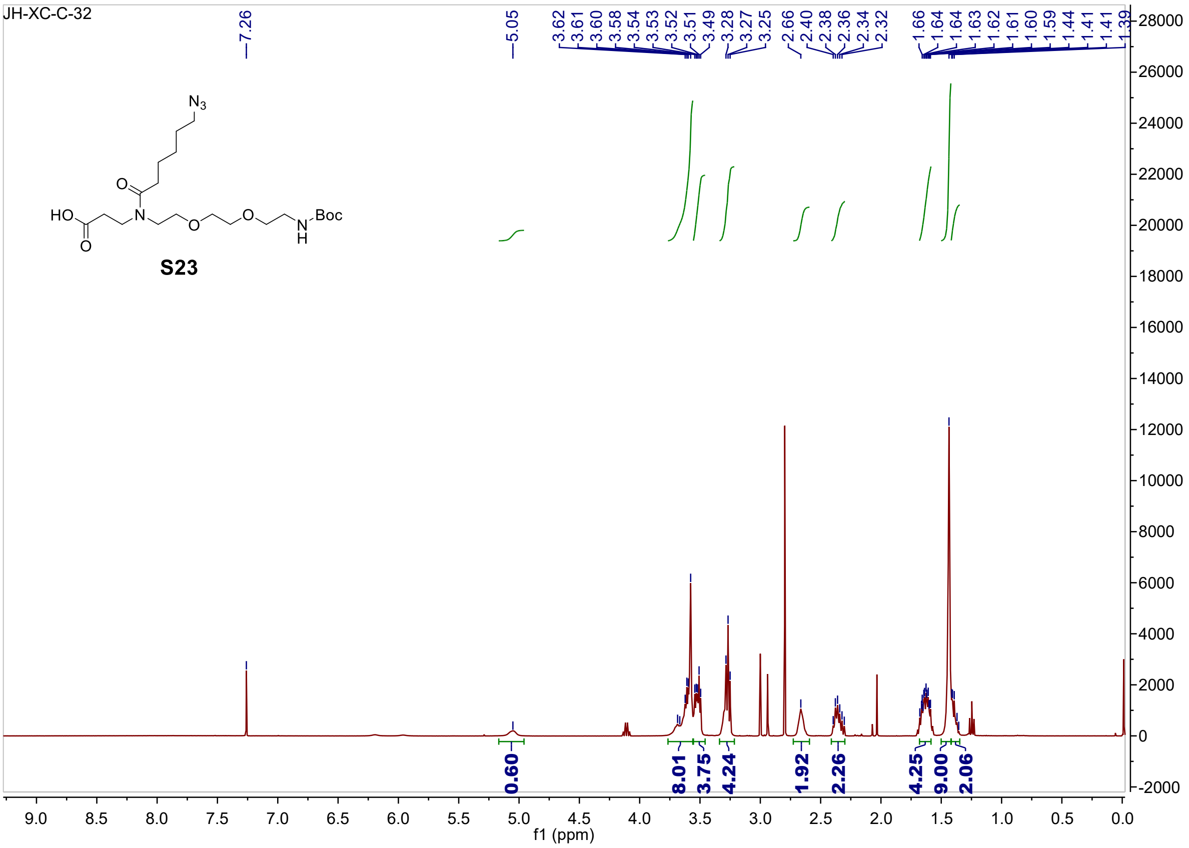
JH-XC-C-32
7.26
5.05
3.62 3.61 3.60 3.58 3.54 3.53 3.52 3.51 3.49 3.28 3.27 3.25
2.66 2.40 2.38 2.36 2.34 2.32
1.66 1.64 1.64 1.63 1.62 1.61 1.60 1.59 1.44 1.41 1.41 1.39
0.60 8.01 3.75 4.24 1.92 2.26 4.25 9.00 2.06
N3
O
HO
N
O
O
N
Boc
H
O
S23
f1 (ppm)

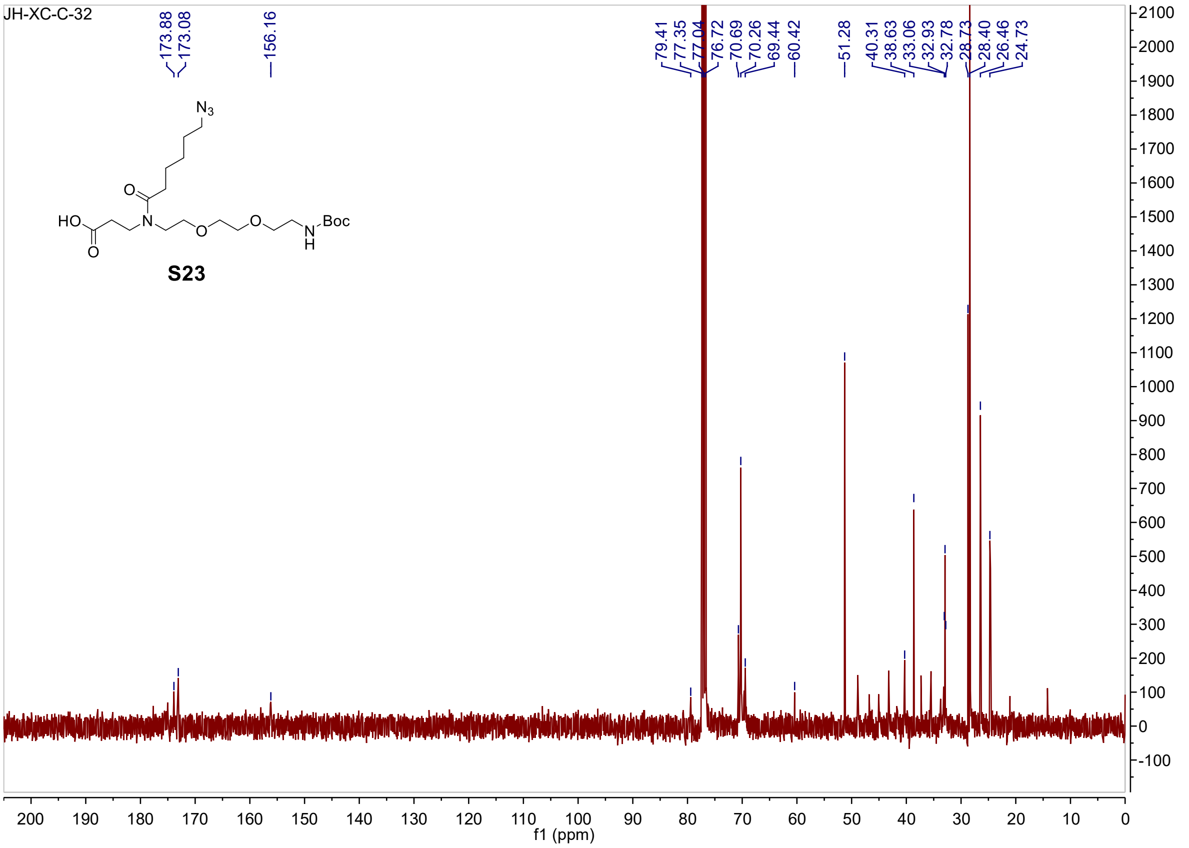
JH-XC-C-32
173.88 173.08
156.16
79.41 77.35 77.04 76.72 70.69 70.26 69.44 60.42
51.28
40.31 38.63 33.06 32.93 32.78 28.73 28.40 26.46 24.73
N3
O
HO
N
O
O
N
Boc
H
O
S23
f1 (ppm)

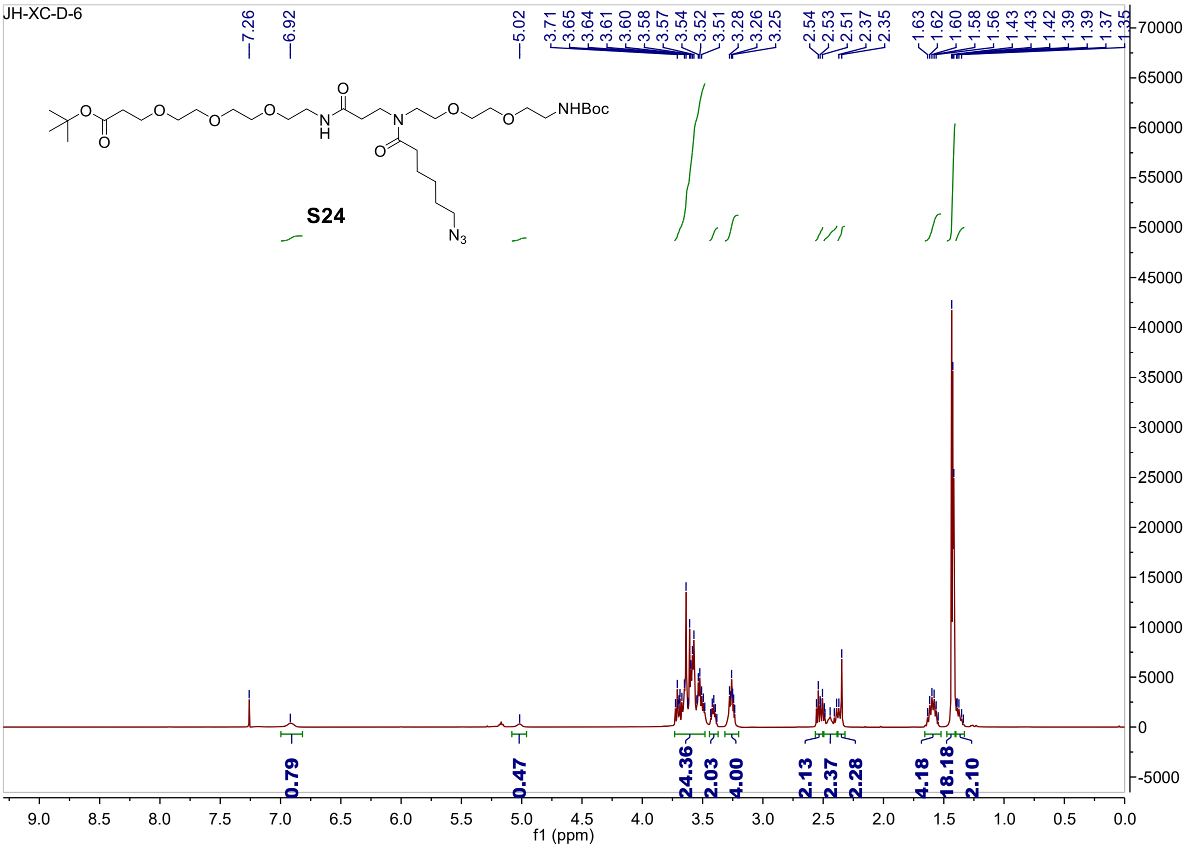
JH-XC-D-6
NHBoc
S24
N3
f1 (ppm)

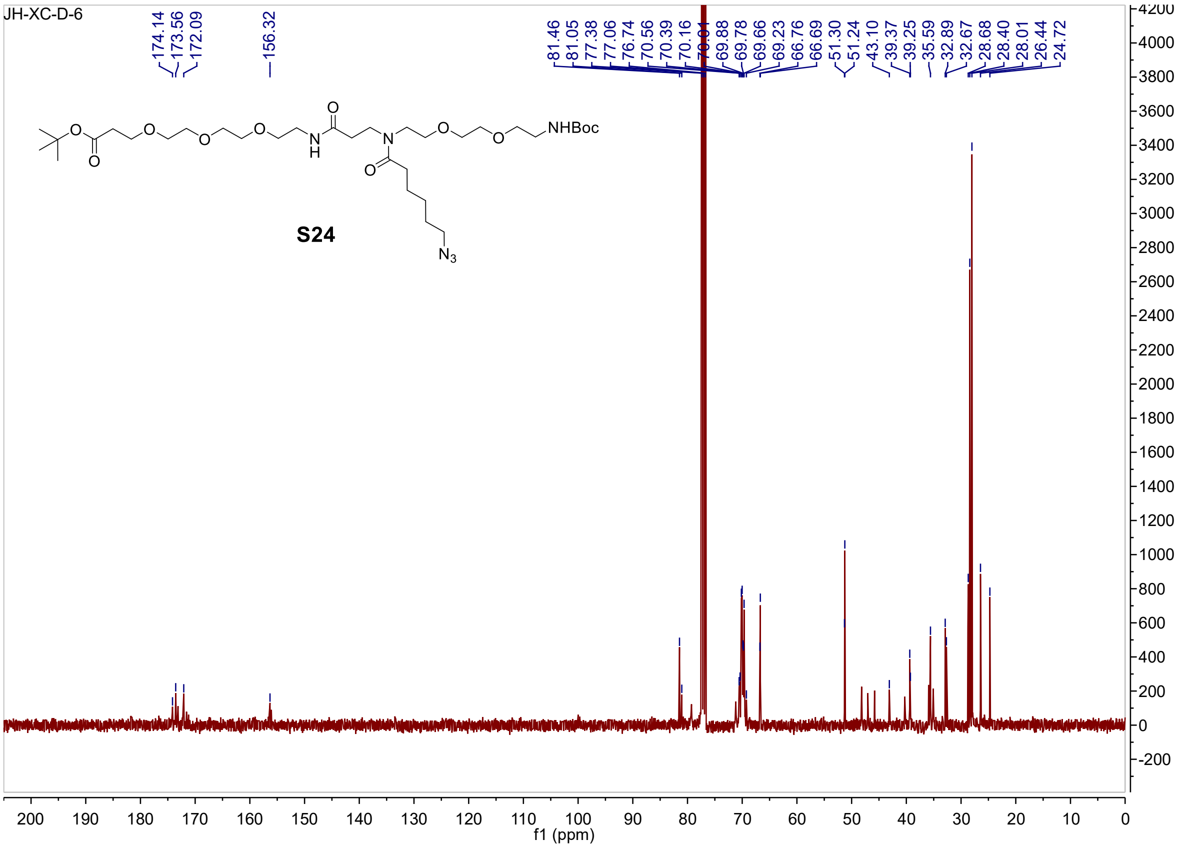
JH-XC-D-6
NHBoc
S24
N3
f1 (ppm)

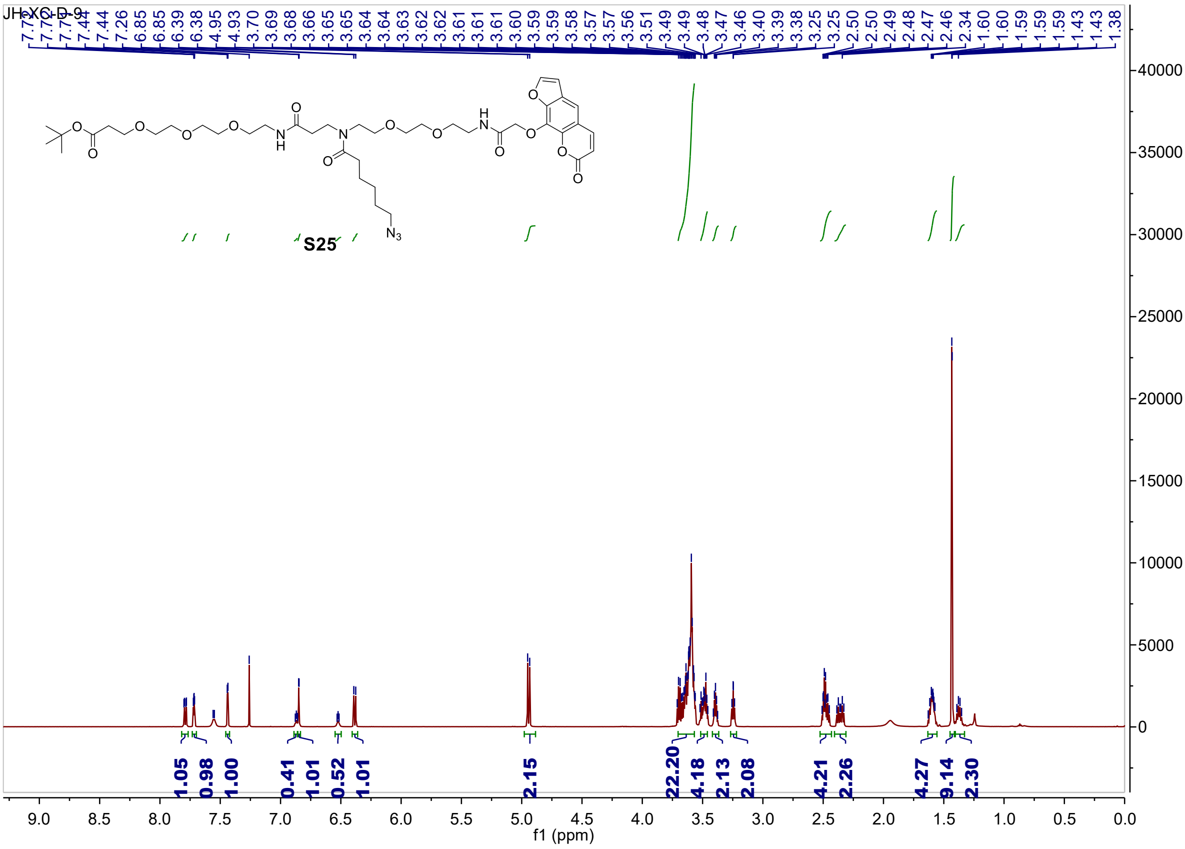
JH-XC-D-9
S25
f1 (ppm)

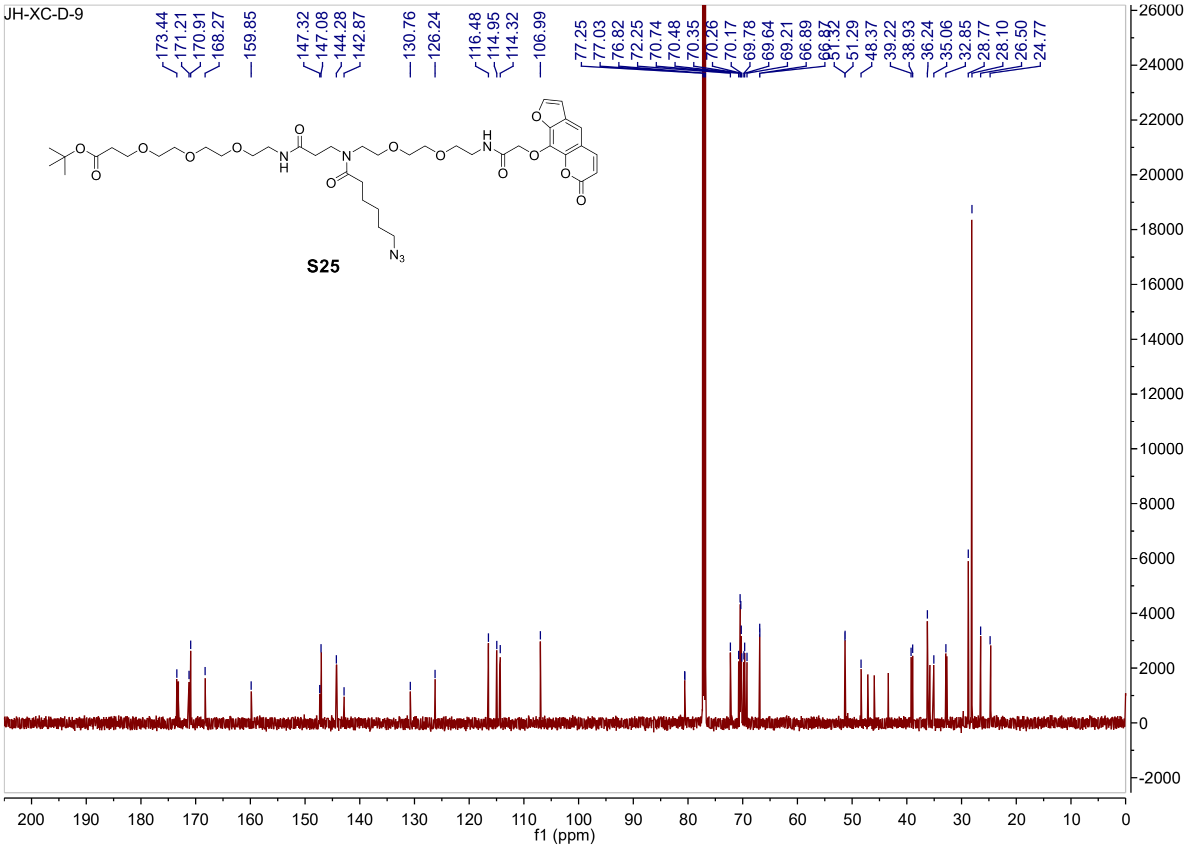
JH-XC-D-9
S25
f1 (ppm)

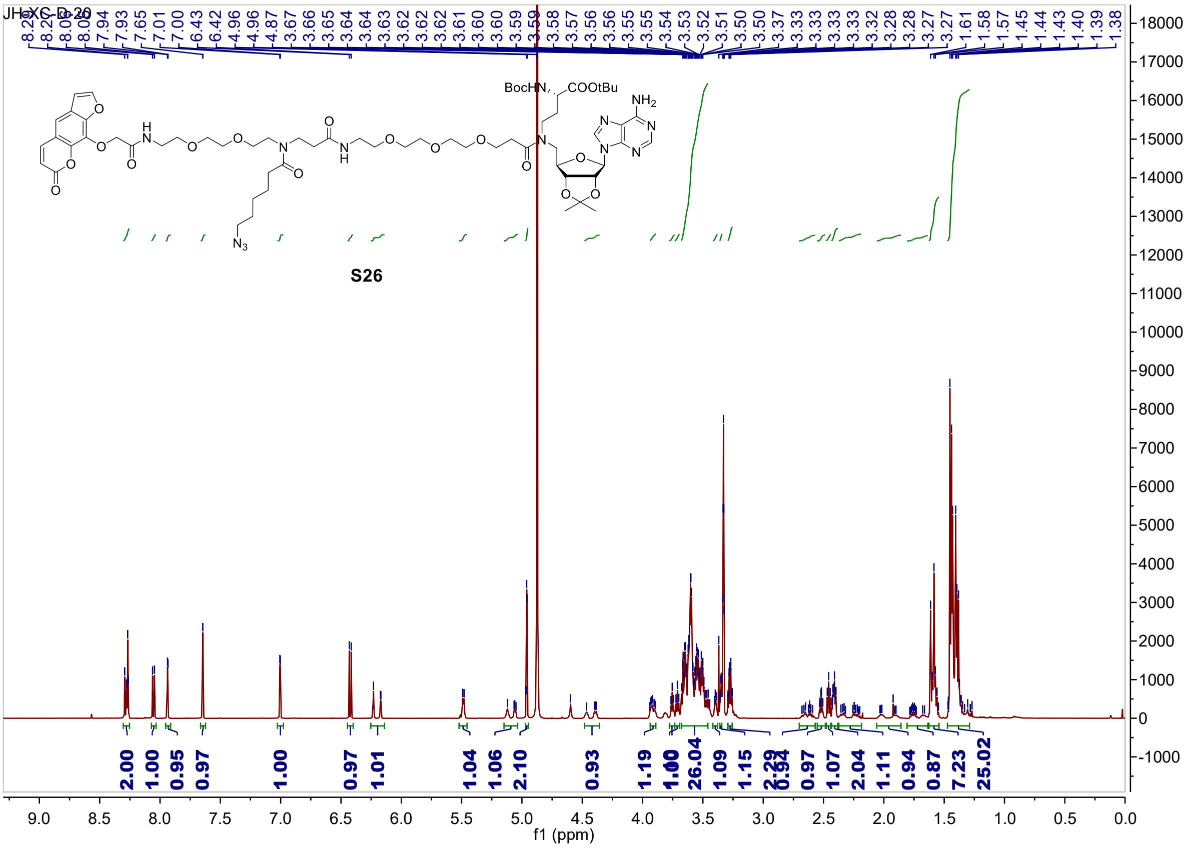
JH-XC-D-20
S26
f1 (ppm)

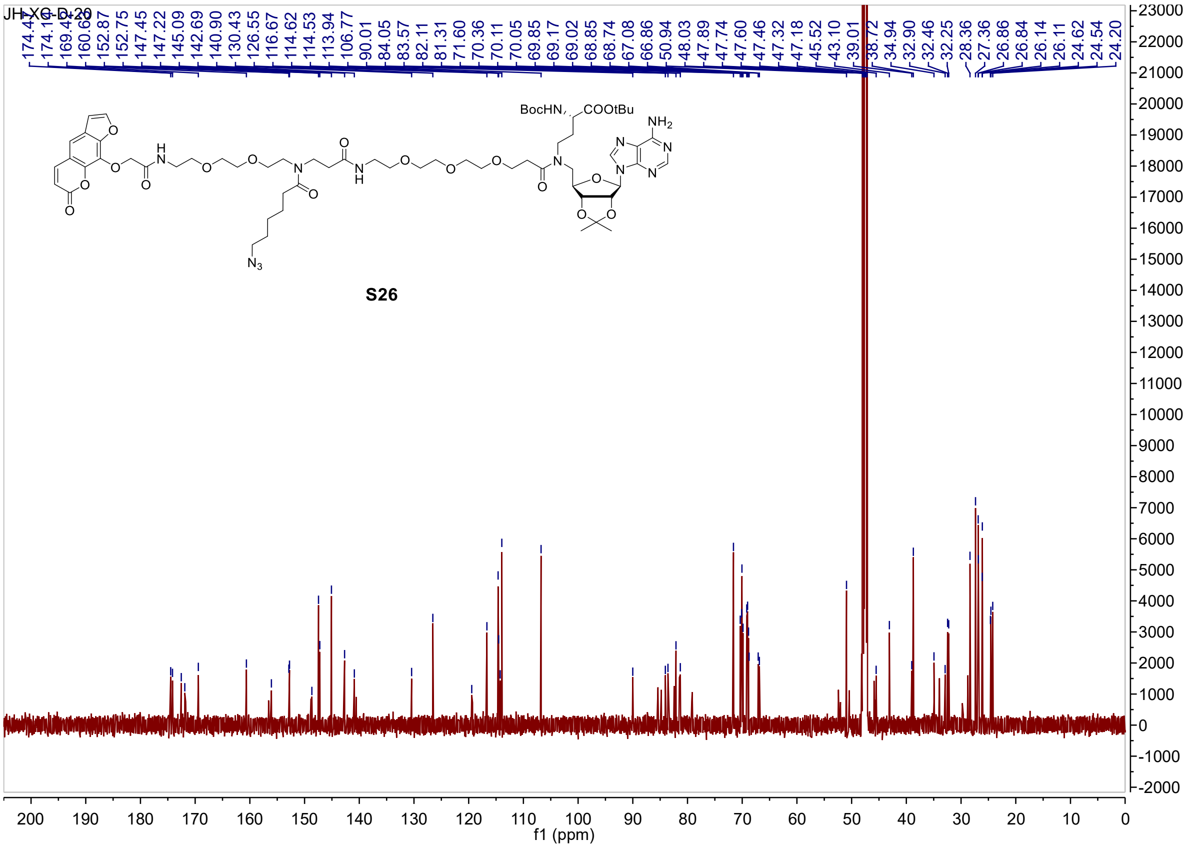
JH-XC-D-20
S26
f1 (ppm)

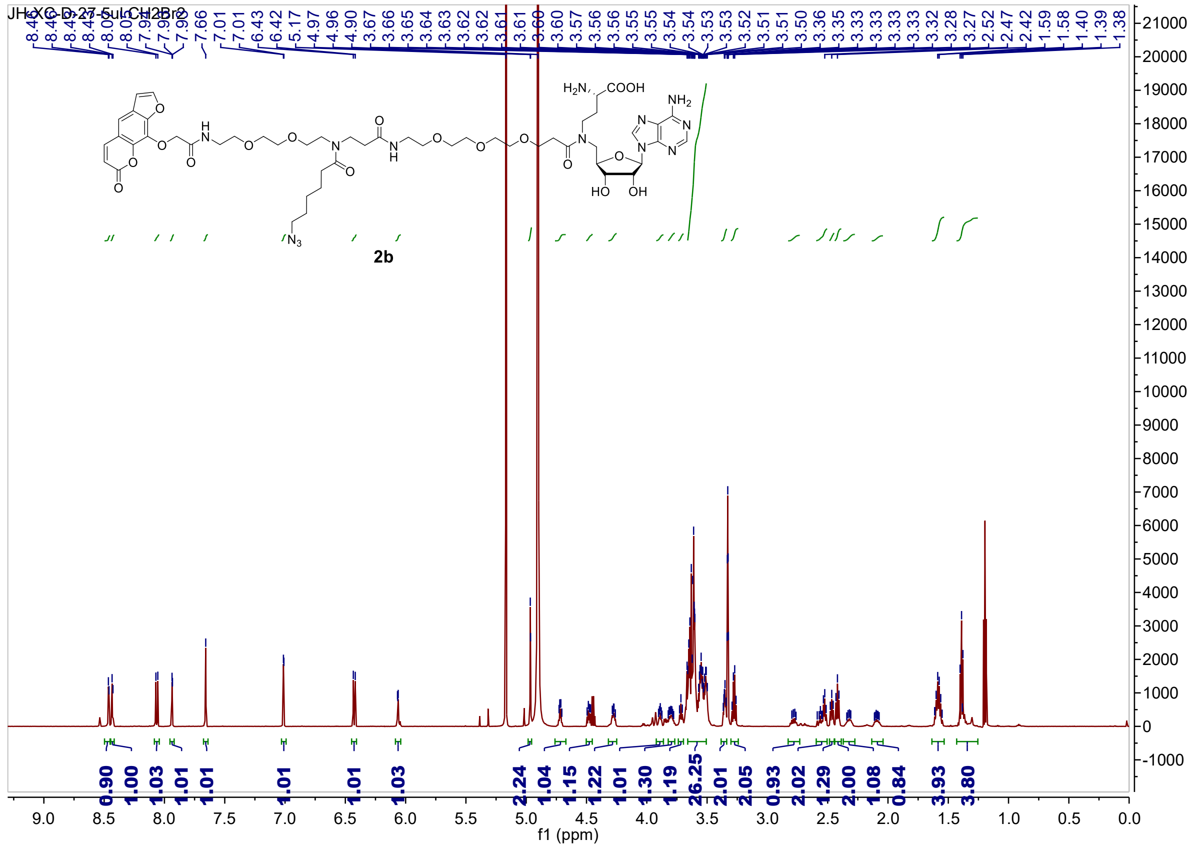

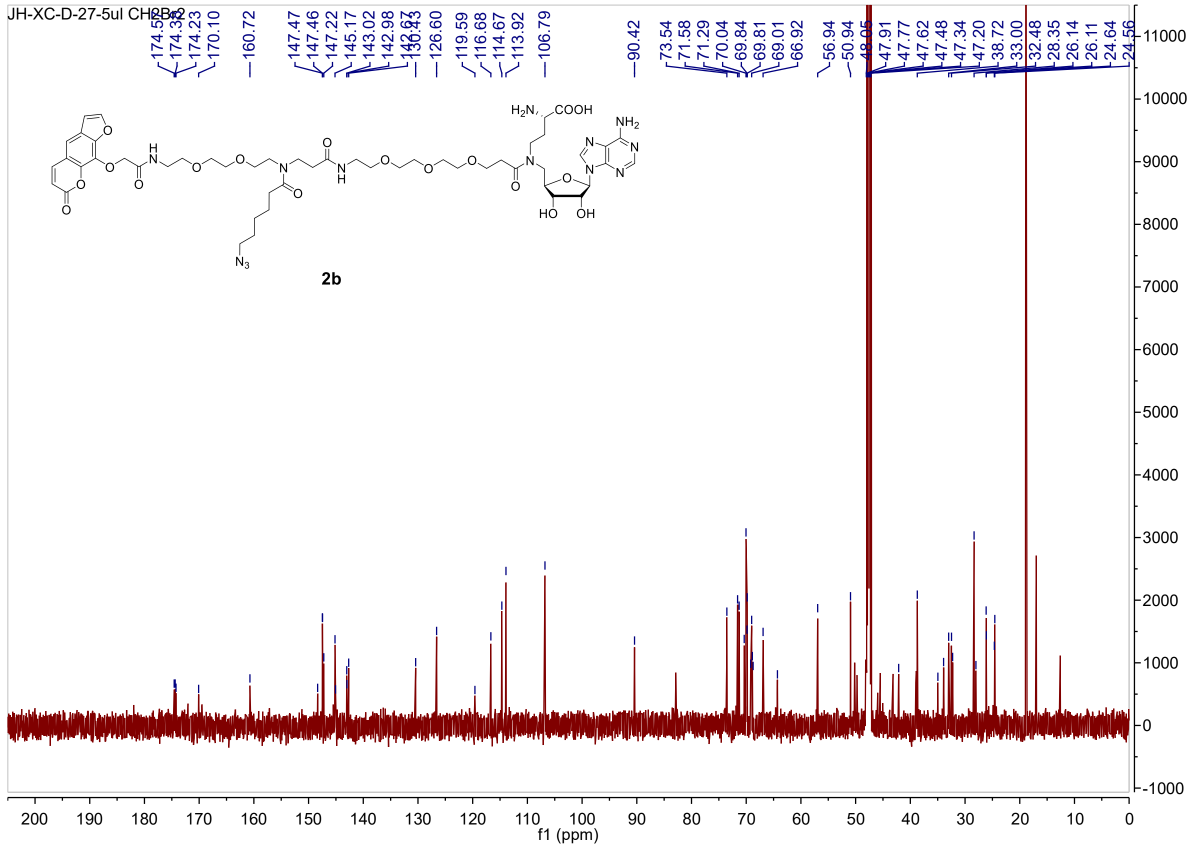

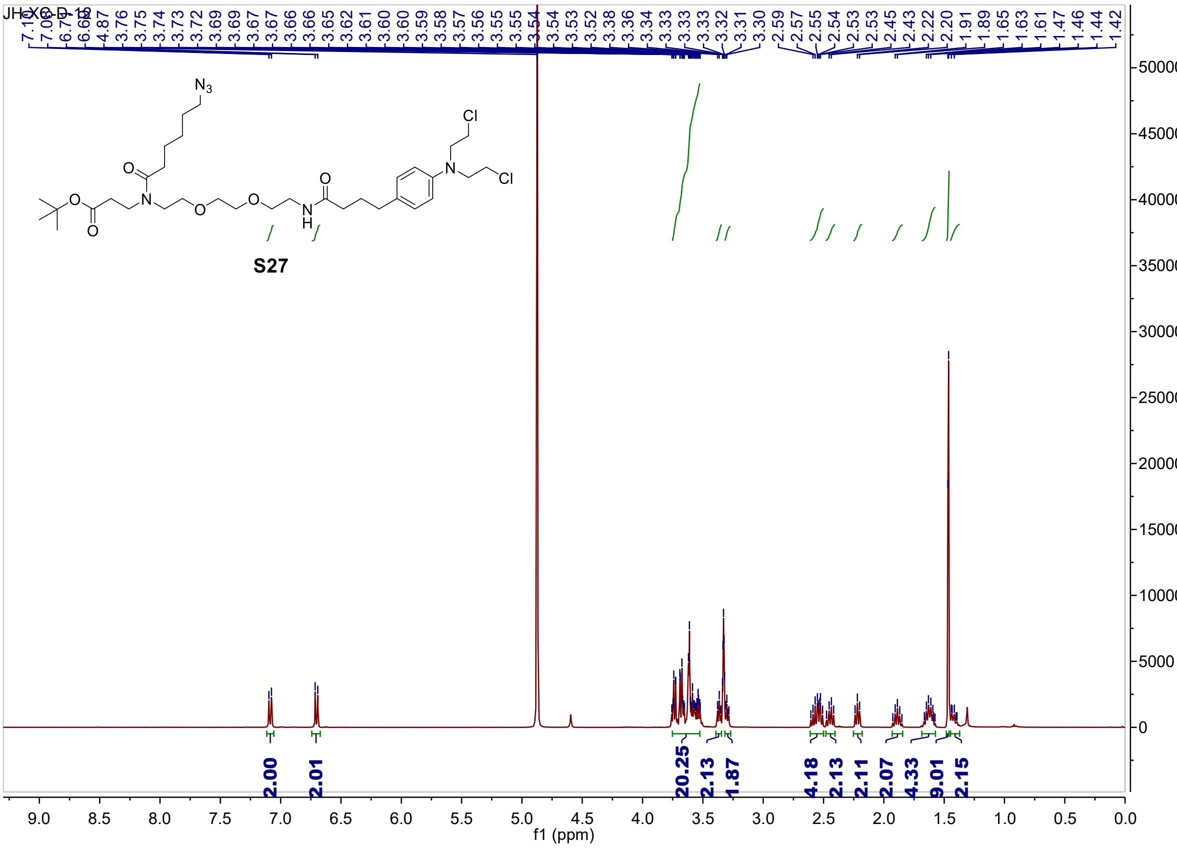

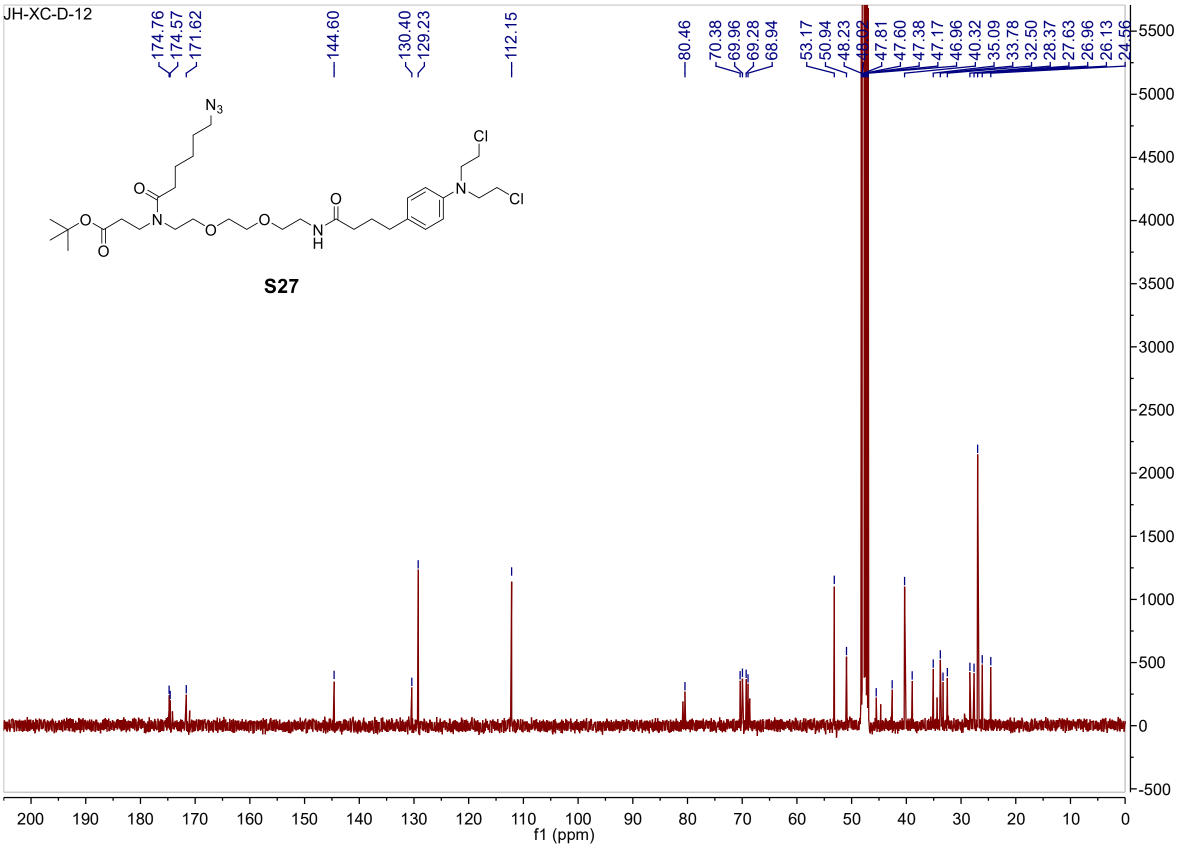

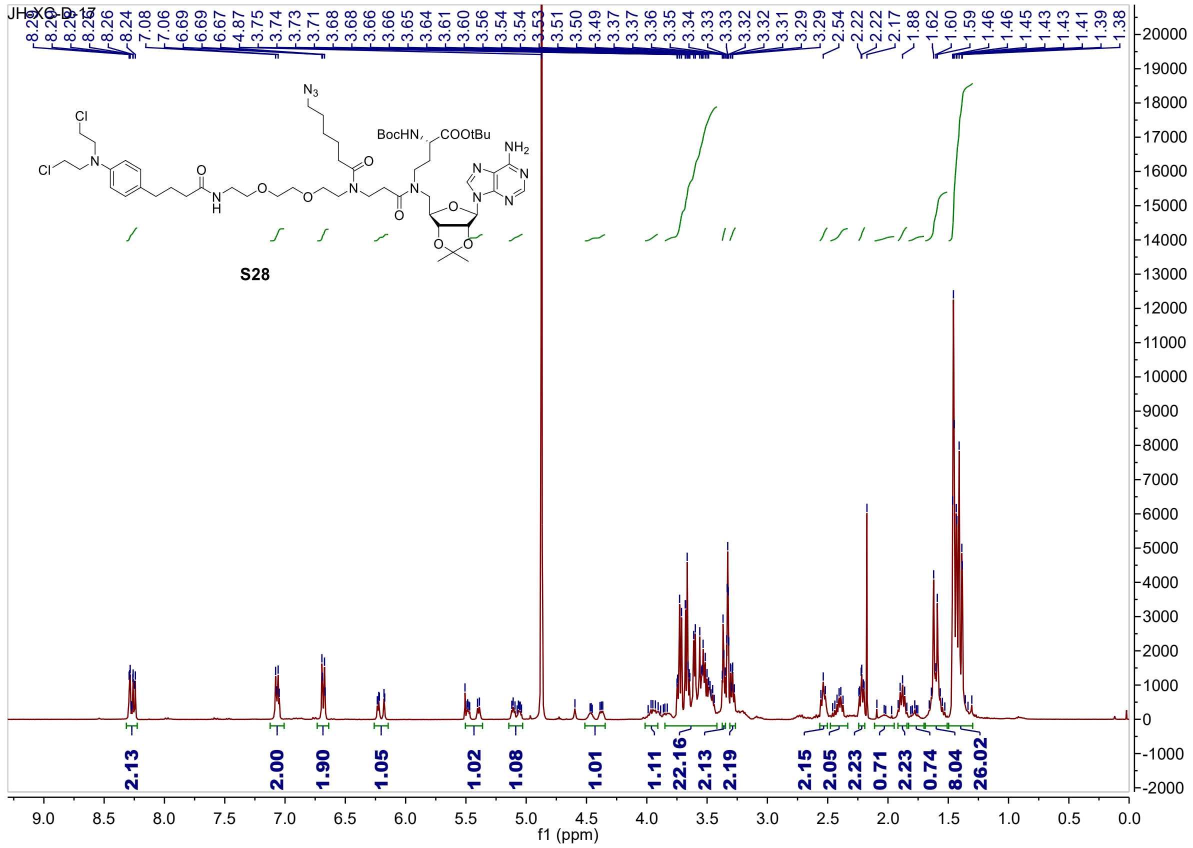

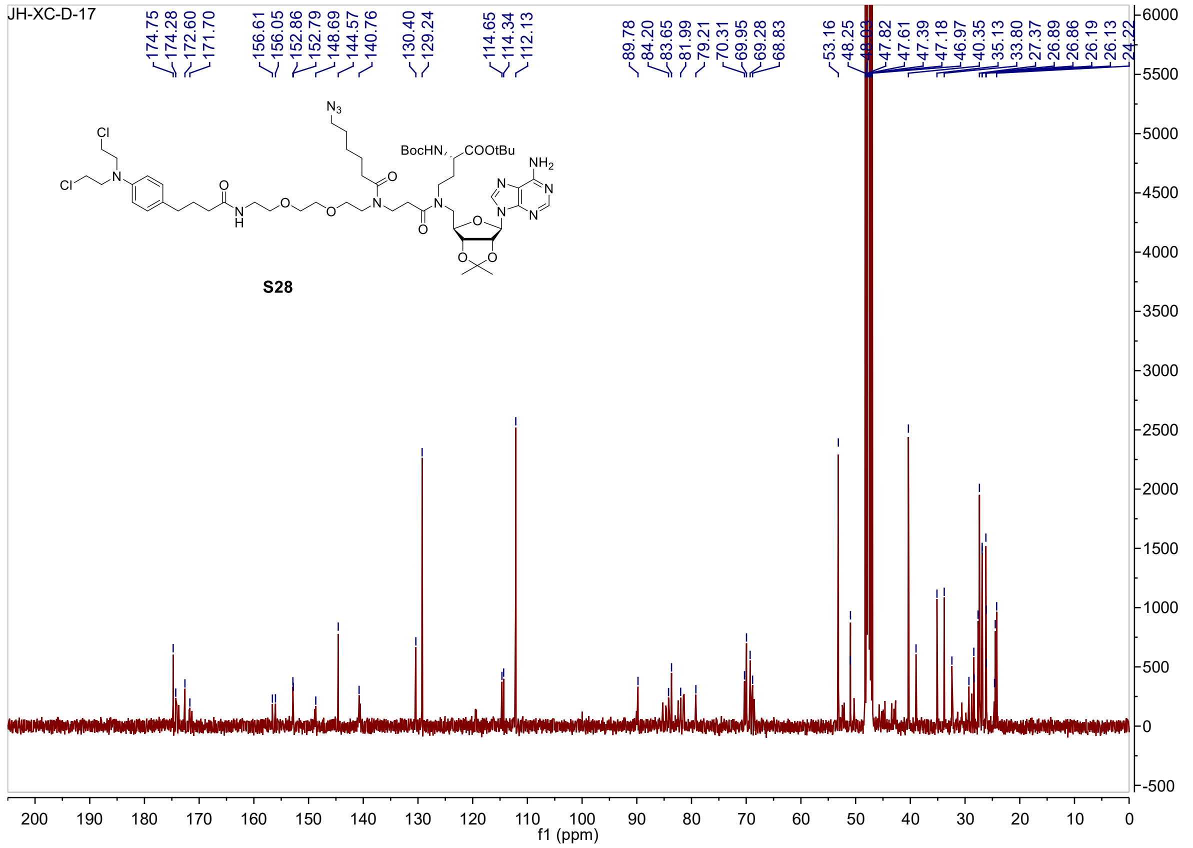

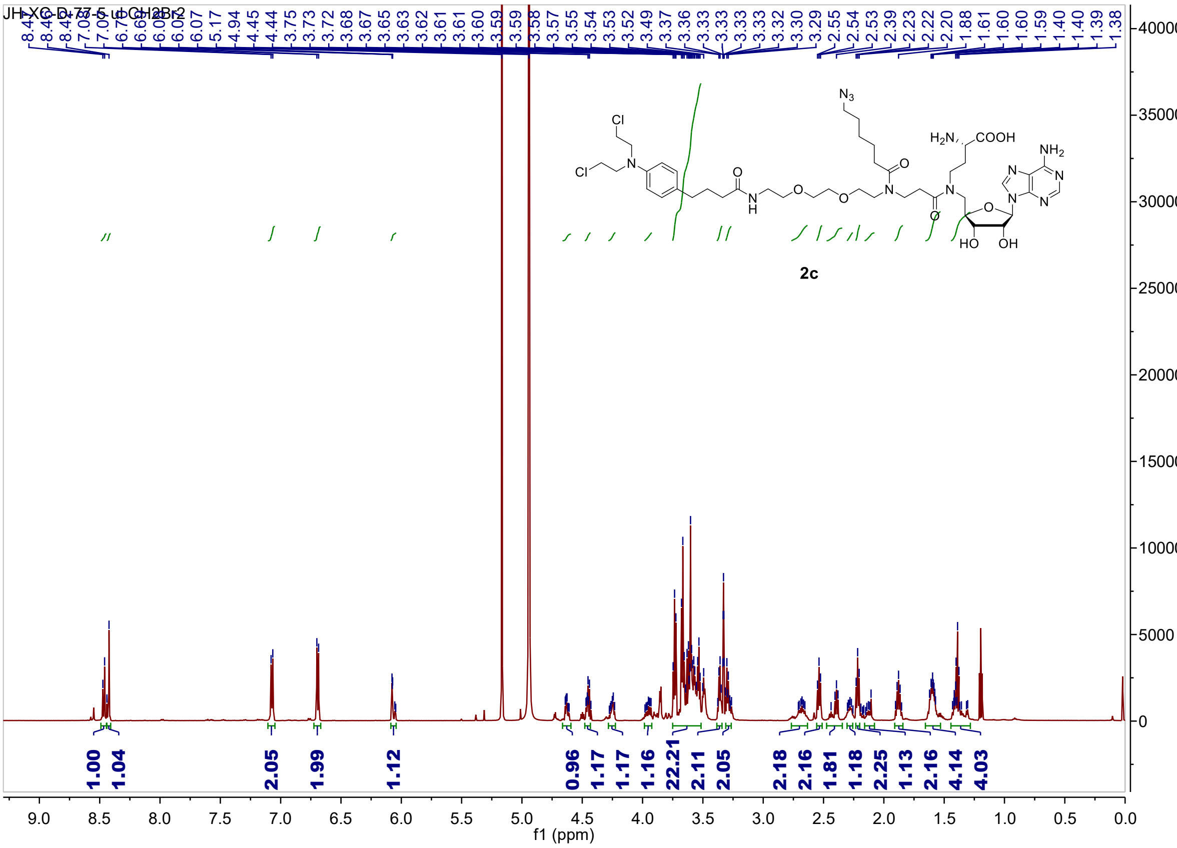
3.75
3.73
3.72
3.68
3.67
3.65
3.63
3.62
3.61
3.61
3.60
3.59
3.59
3.58
3.57
3.55
3.54
3.53
3.52
3.49
3.37
3.36
3.33
3.33
3.33
3.33
3.32
3.30
3.29
2.55
2.54
2.53
2.39
2.23
2.22
2.20
1.88
1.61
1.60
1.60
1.59
1.40
1.39
1.38
2c
1.00
1.04
2.05
1.99
1.12
0.96
1.17
1.17
1.16
22.21
2.11
2.05
2.18
2.16
1.81
1.18
2.25
1.13
2.16
4.14
4.03
f1 (ppm)

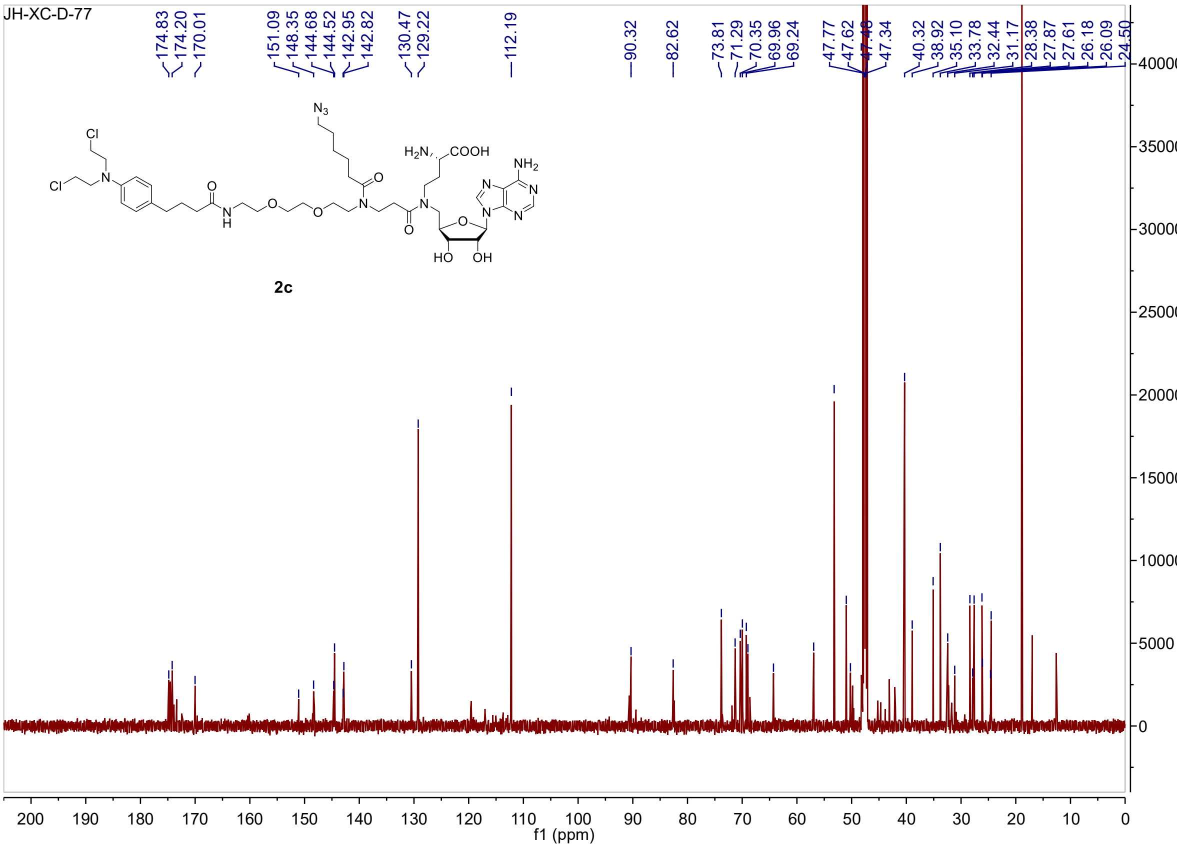
JH-XC-D-77
174.83
174.20
170.01
151.09
148.35
144.68
144.52
142.95
142.82
130.47
129.22
112.19
90.32
82.62
73.81
71.29
70.35
69.96
69.24
47.77
47.62
47.48
47.34
40.32
38.92
35.10
33.78
32.44
31.17
28.38
27.87
27.61
26.18
26.09
24.50
2c
f1 (ppm)

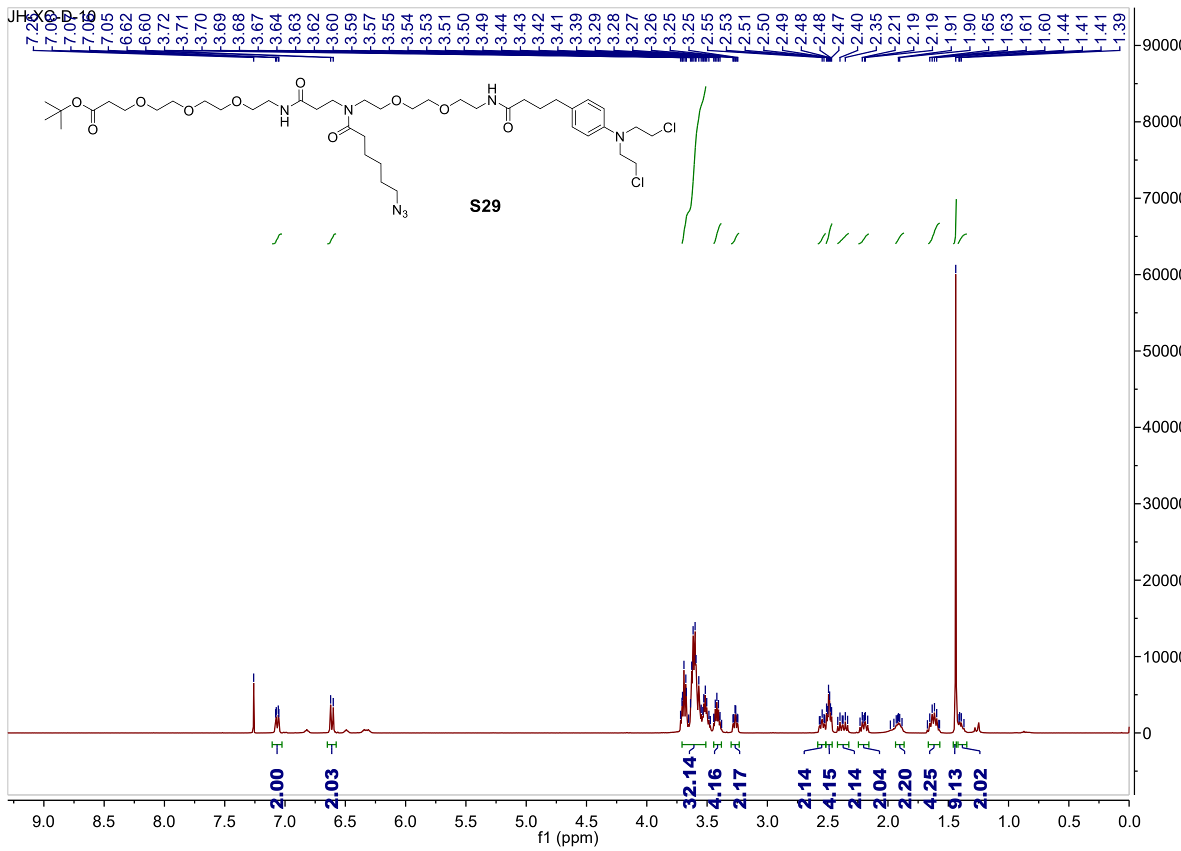

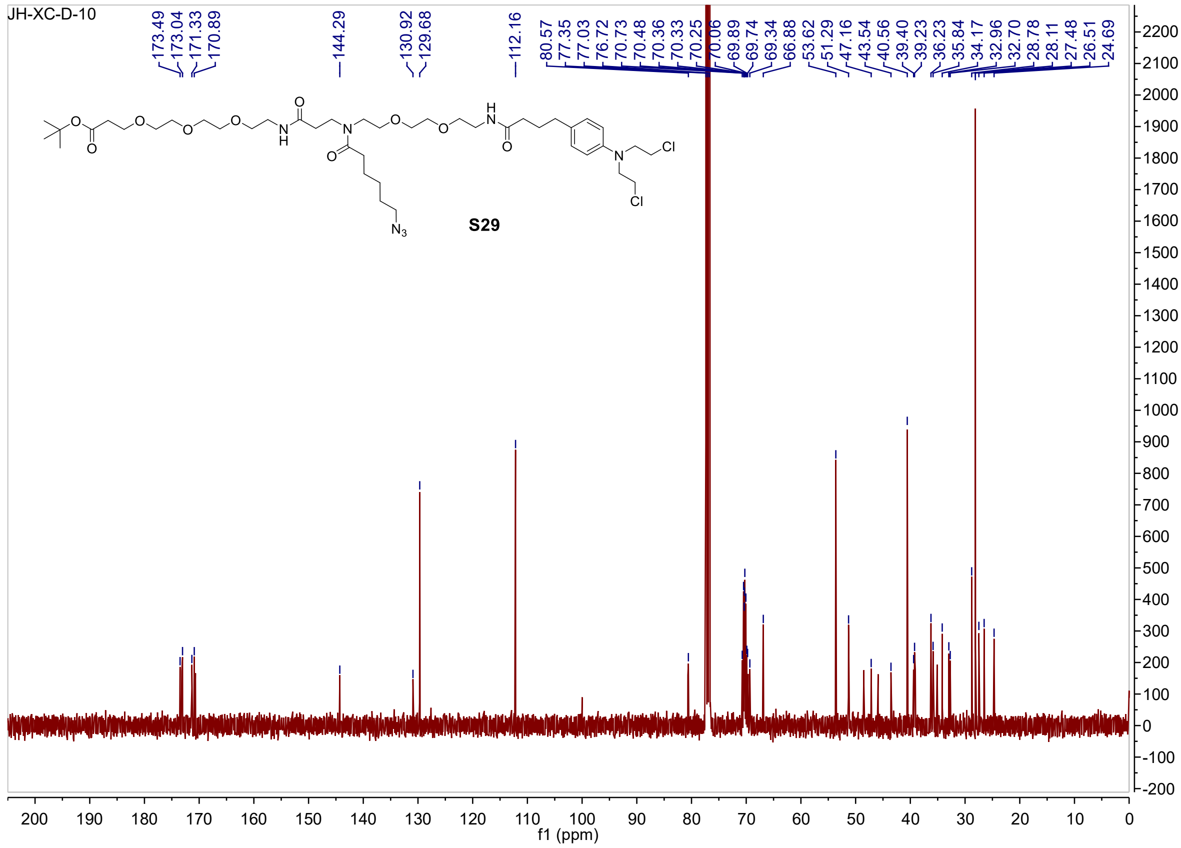

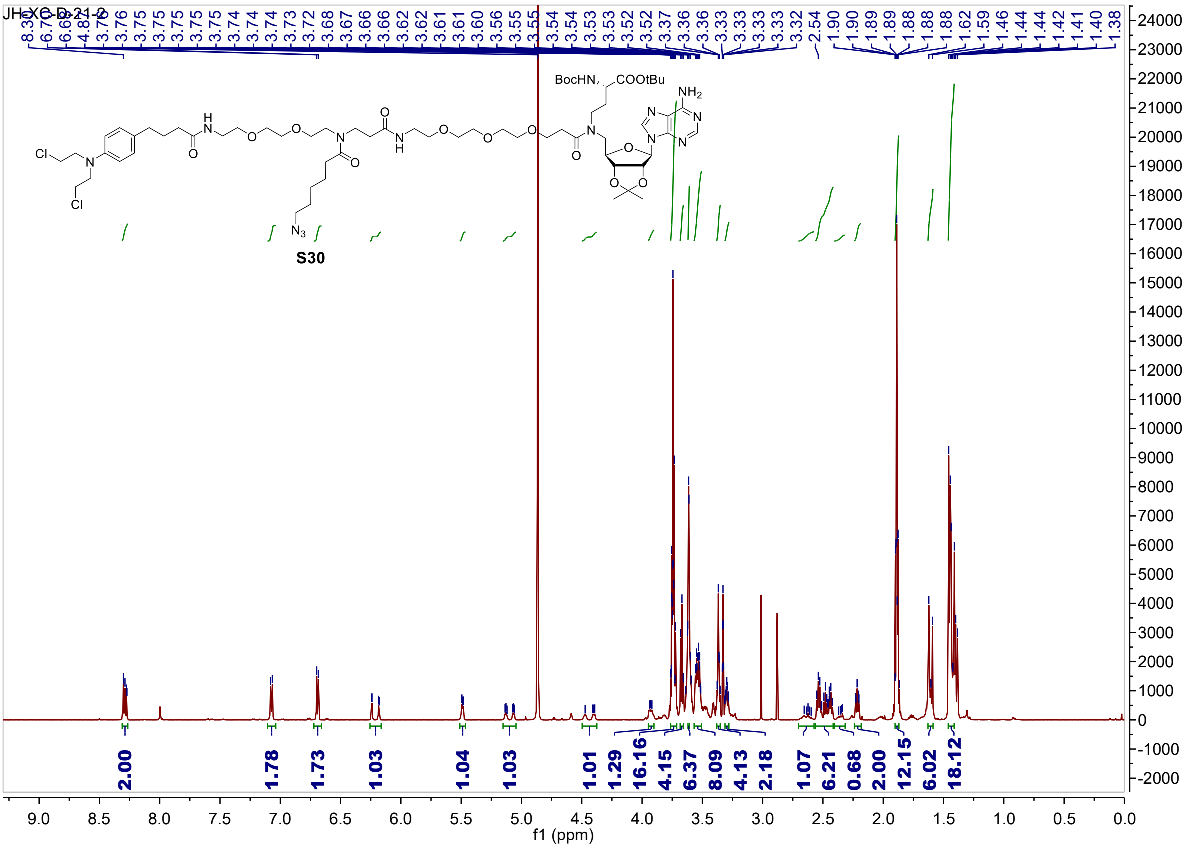

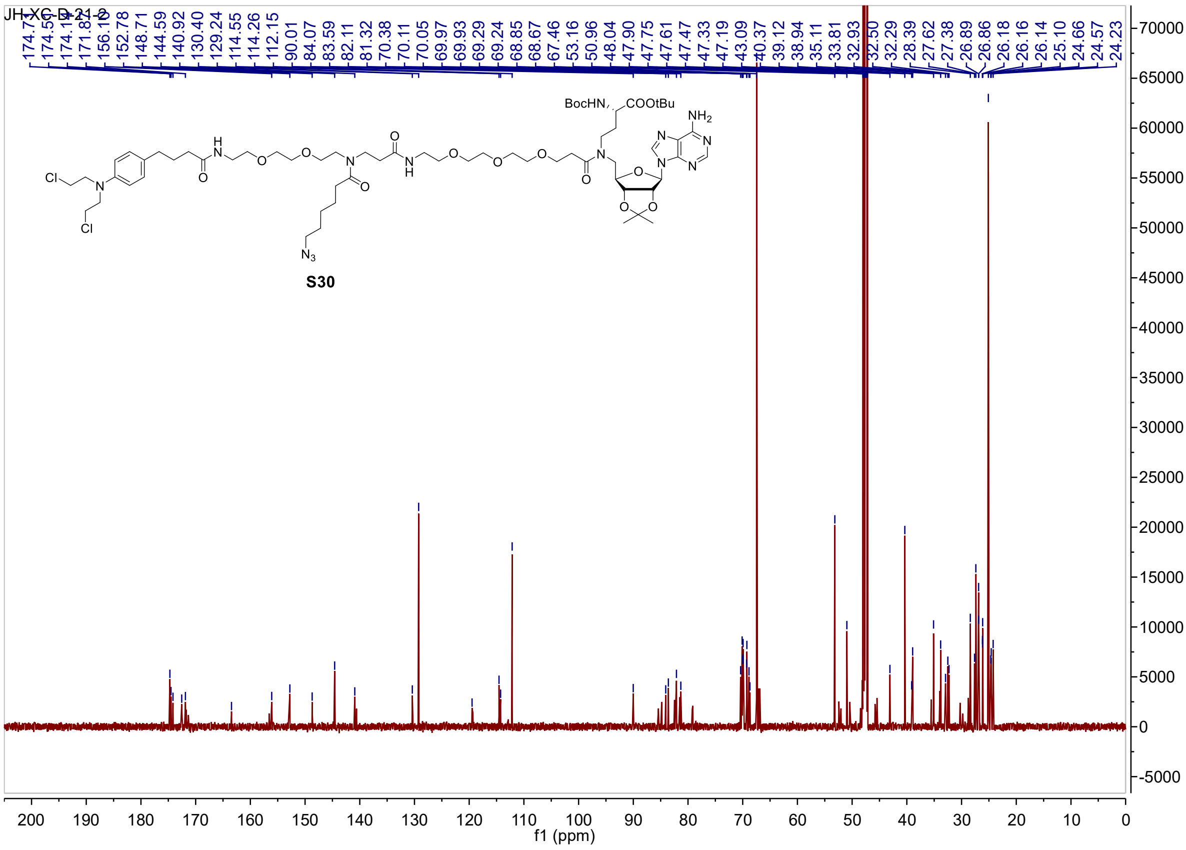

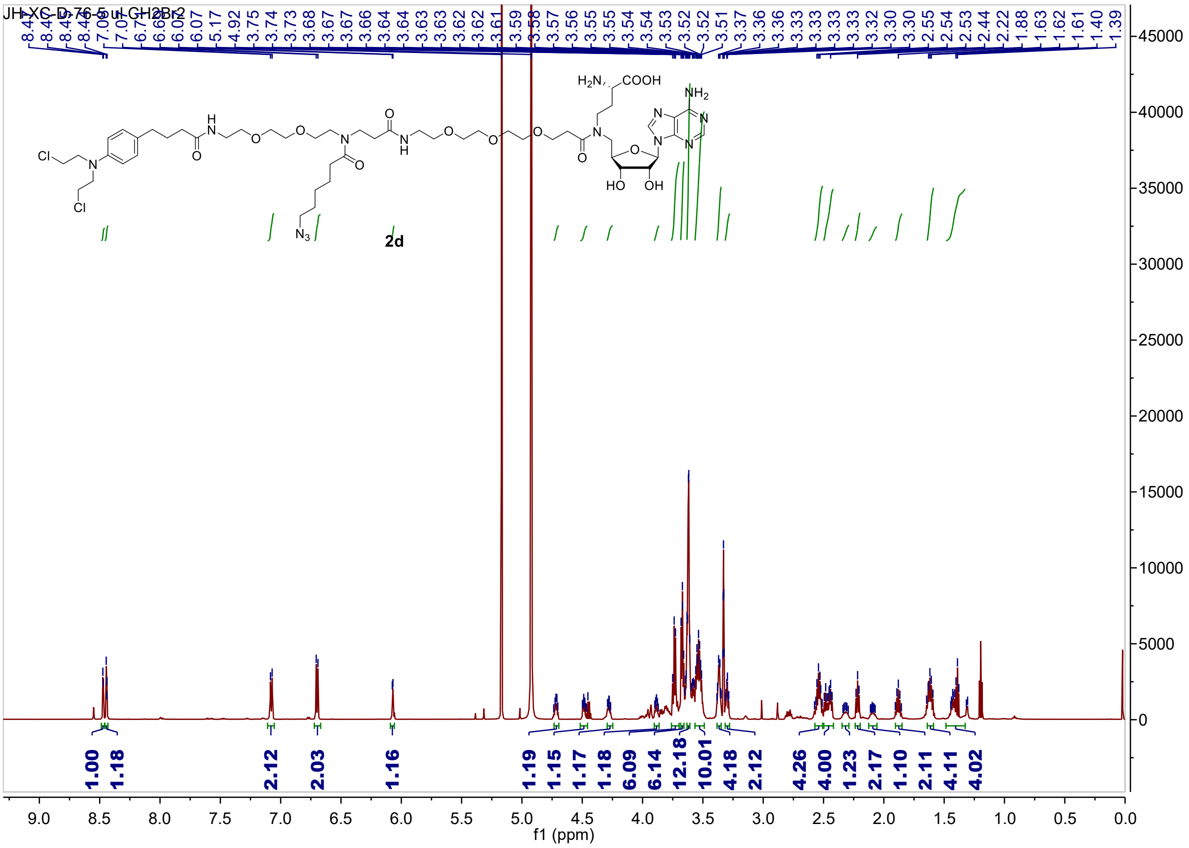

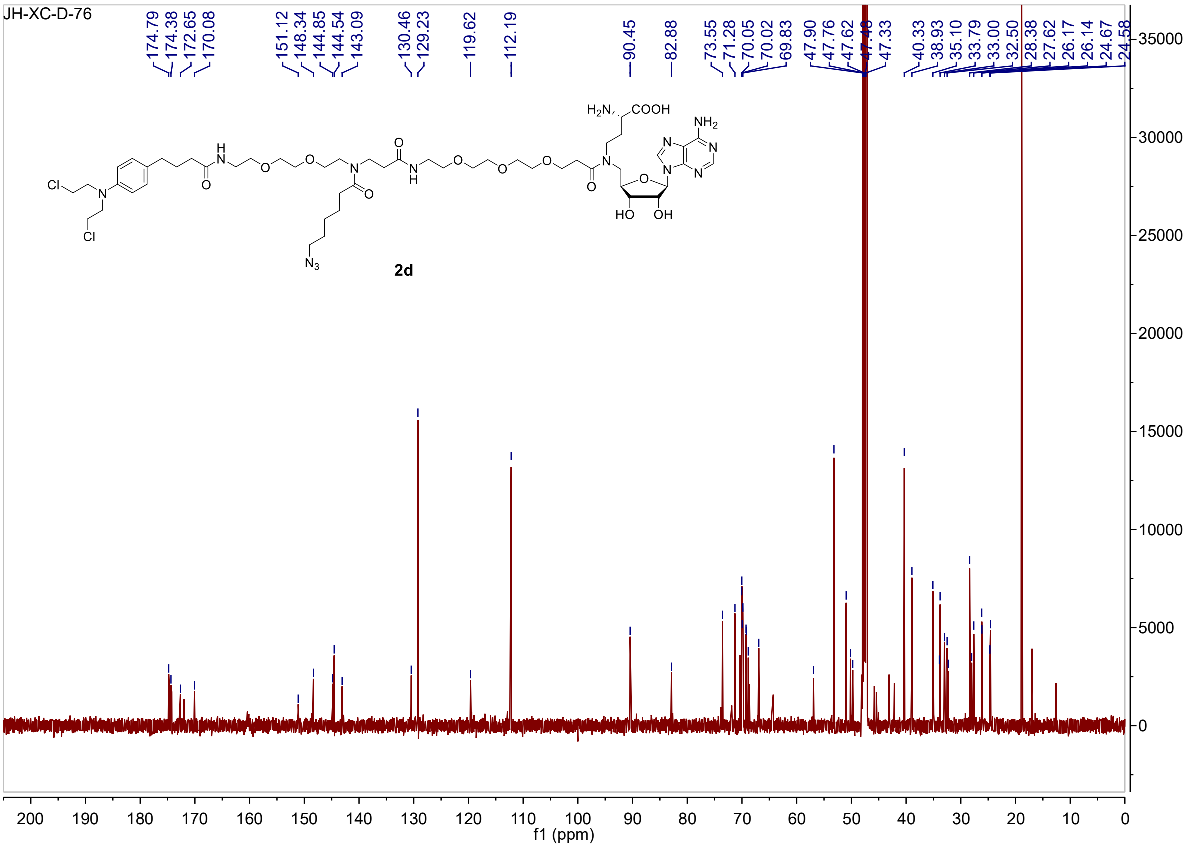

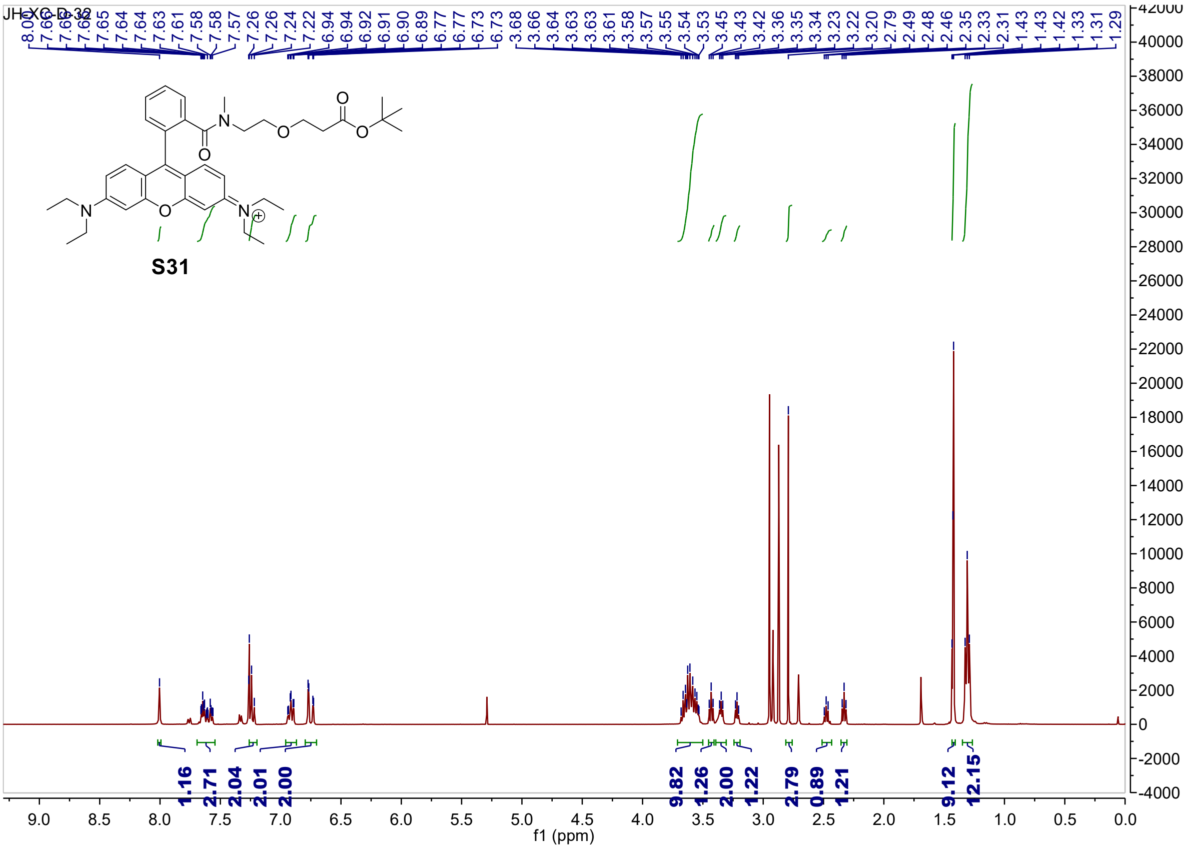

S31

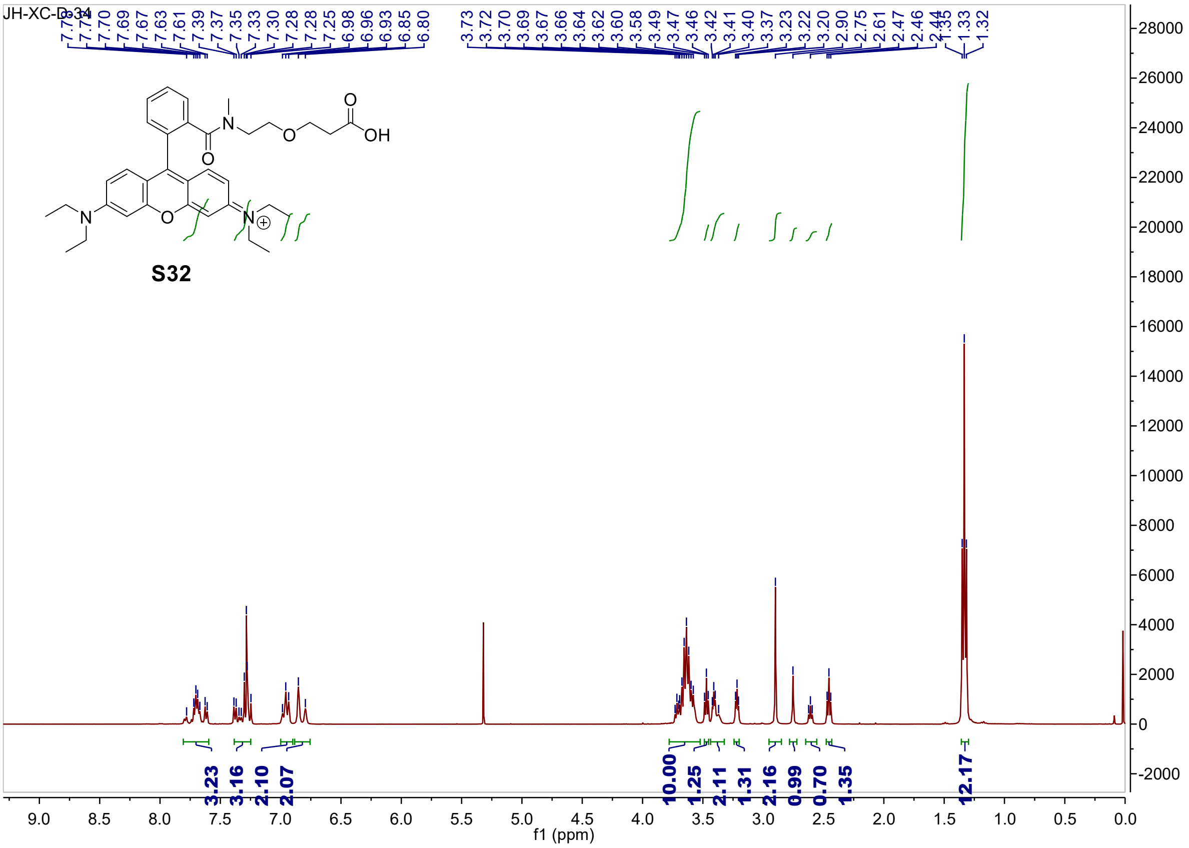

S32

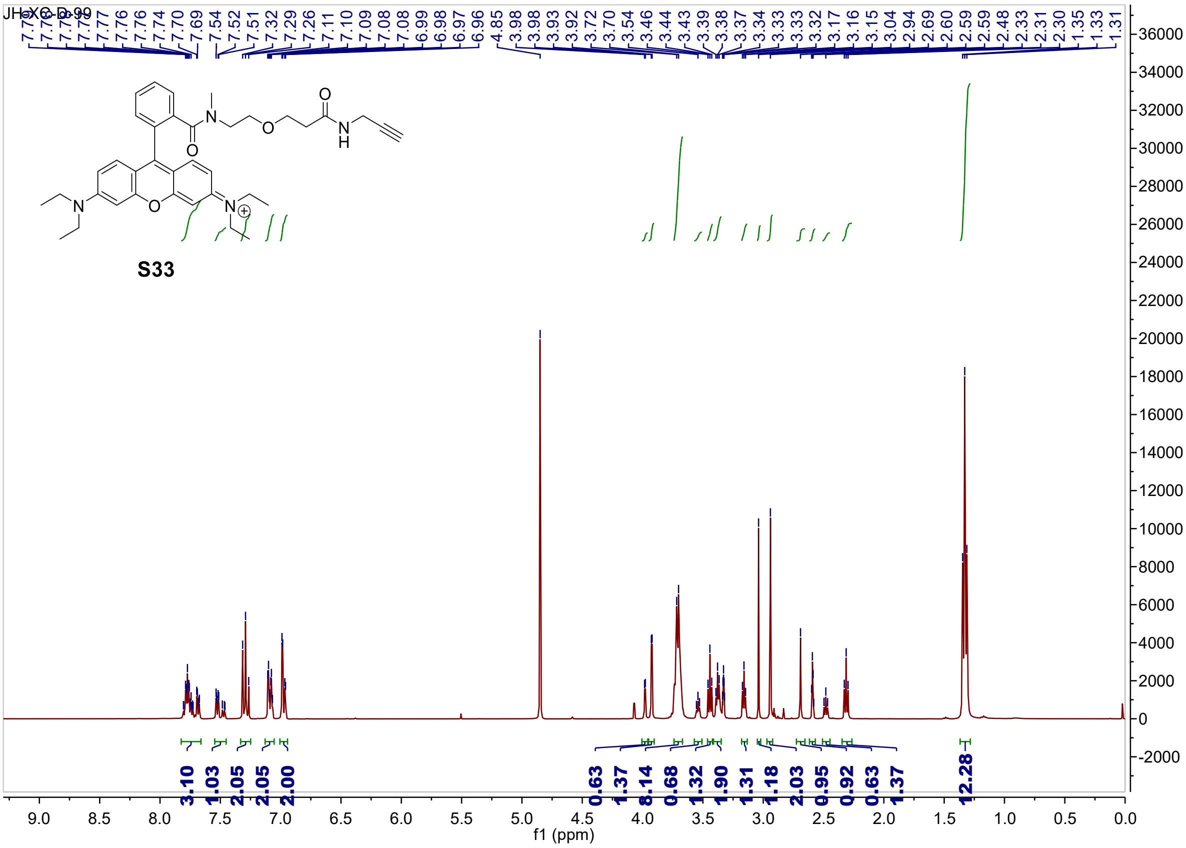

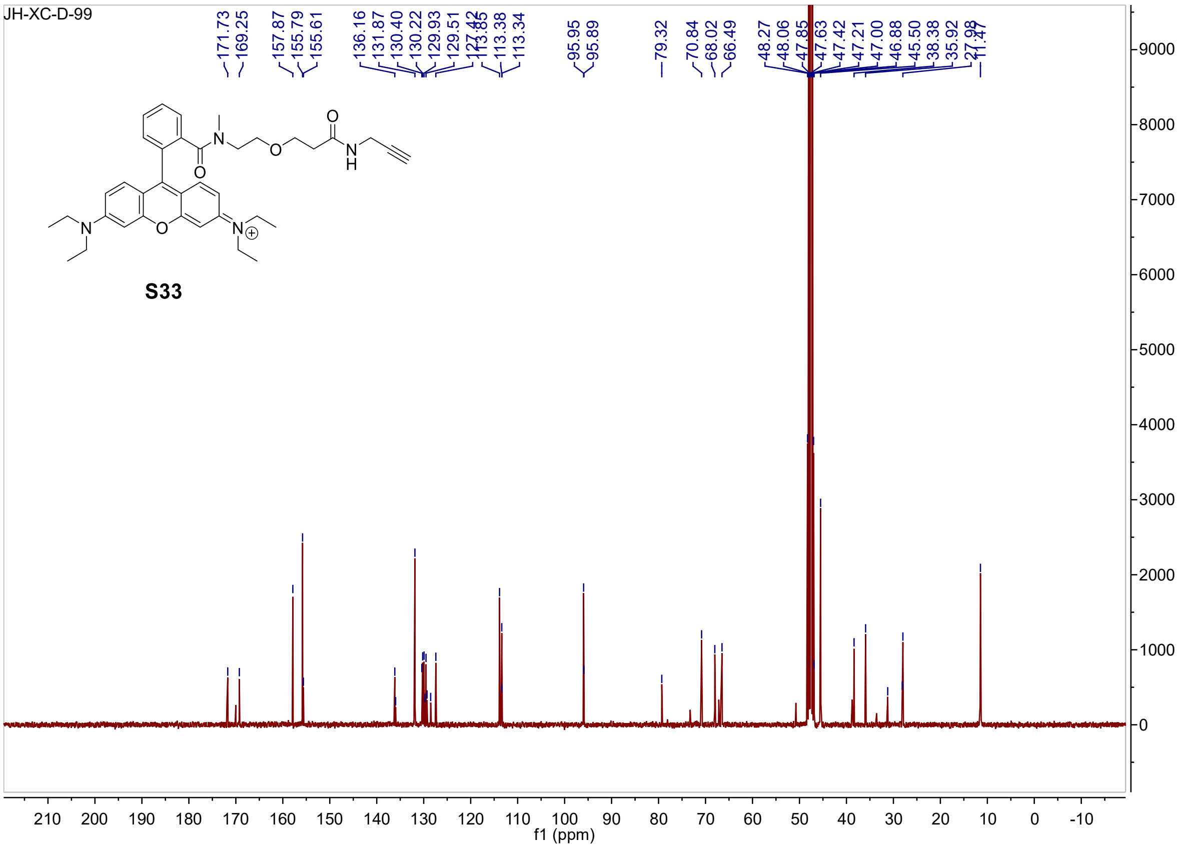

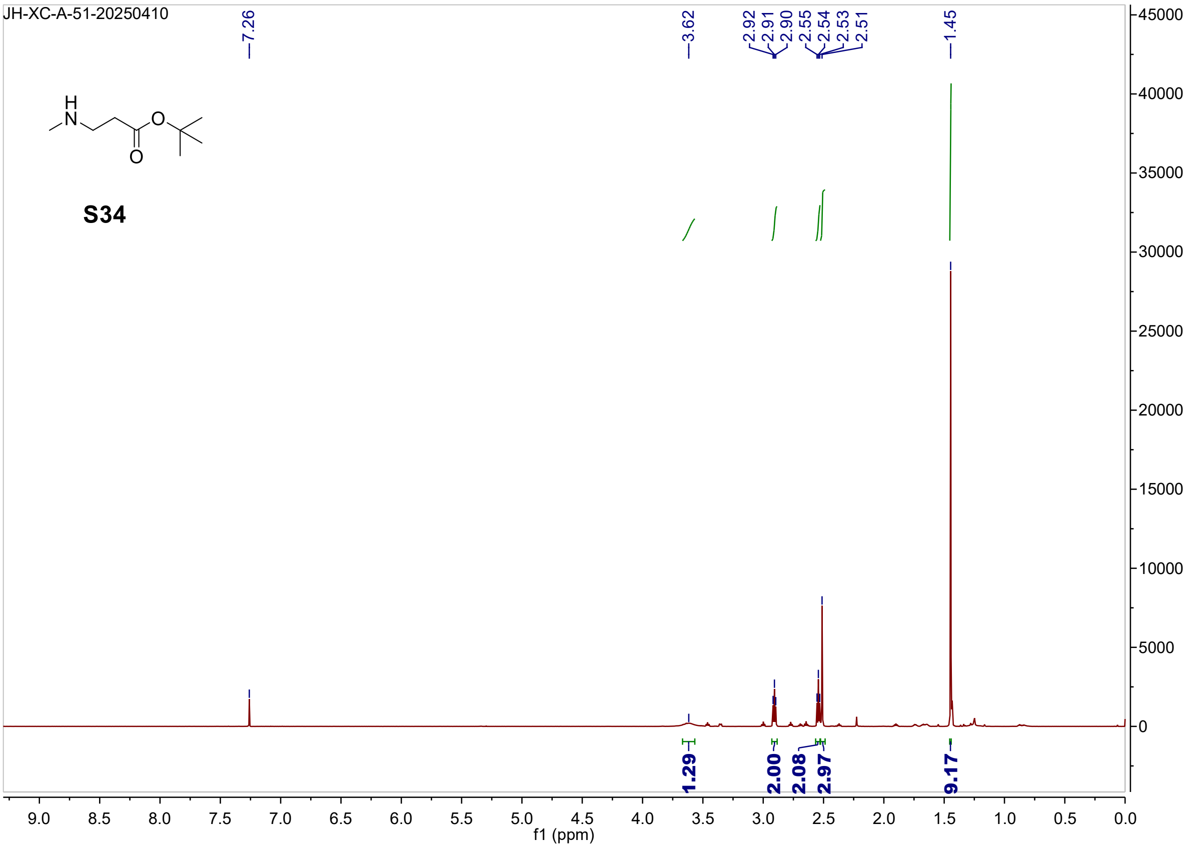
JH-XC-A-51-20250410
7.26
3.62
2.92
2.91
2.90
2.55
2.54
2.53
2.51
1.45
S34
1.29
2.00
2.08
2.97
9.17
f1 (ppm)

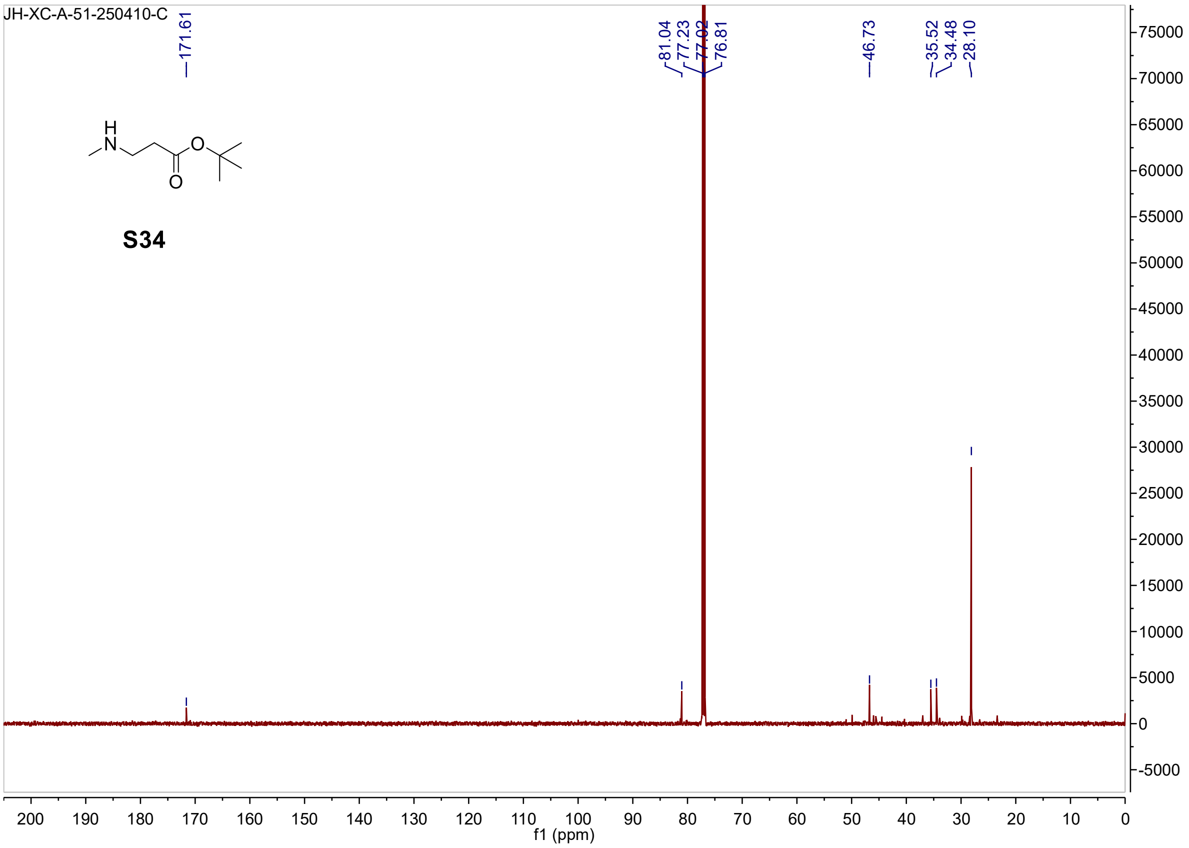
JH-XC-A-51-250410-C
171.61
81.04
77.23
77.02
76.81
46.73
35.52
34.48
28.10
S34
f1 (ppm)

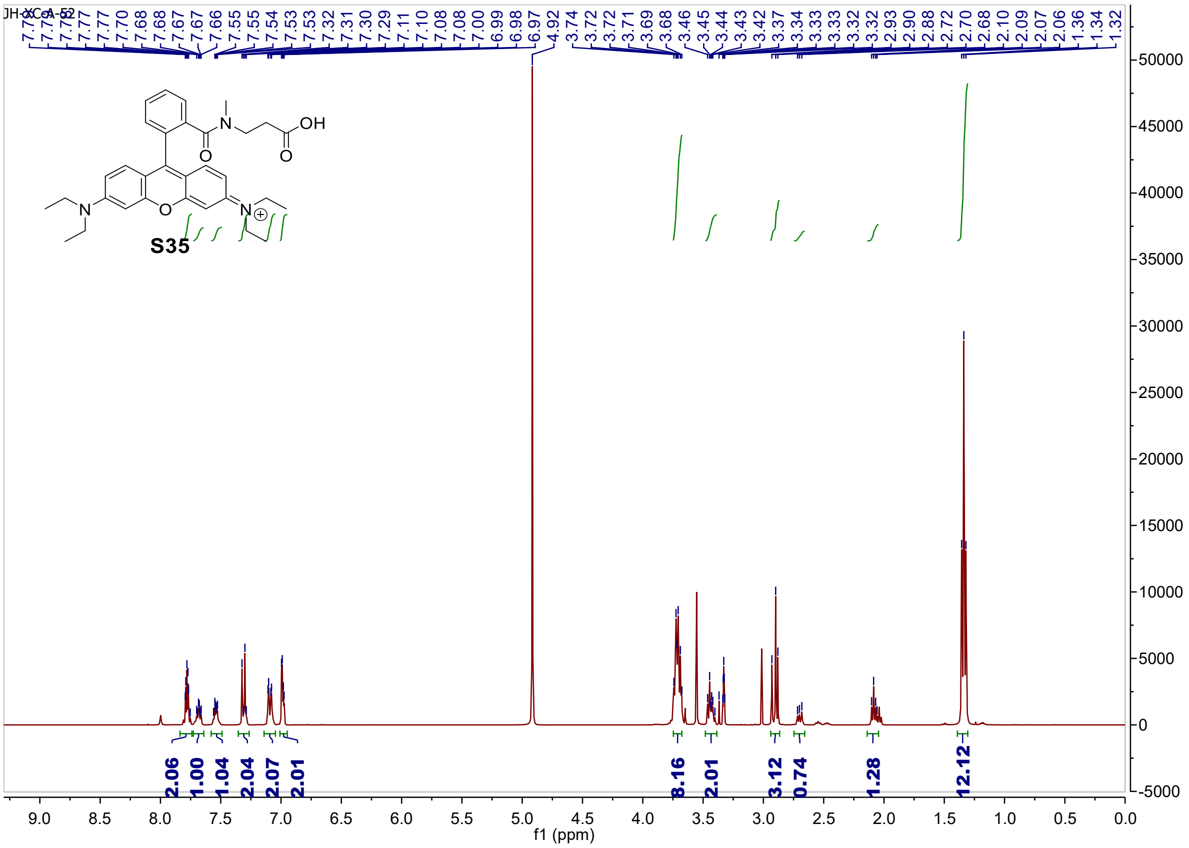
JH-XC-A-52
S35
f1 (ppm)

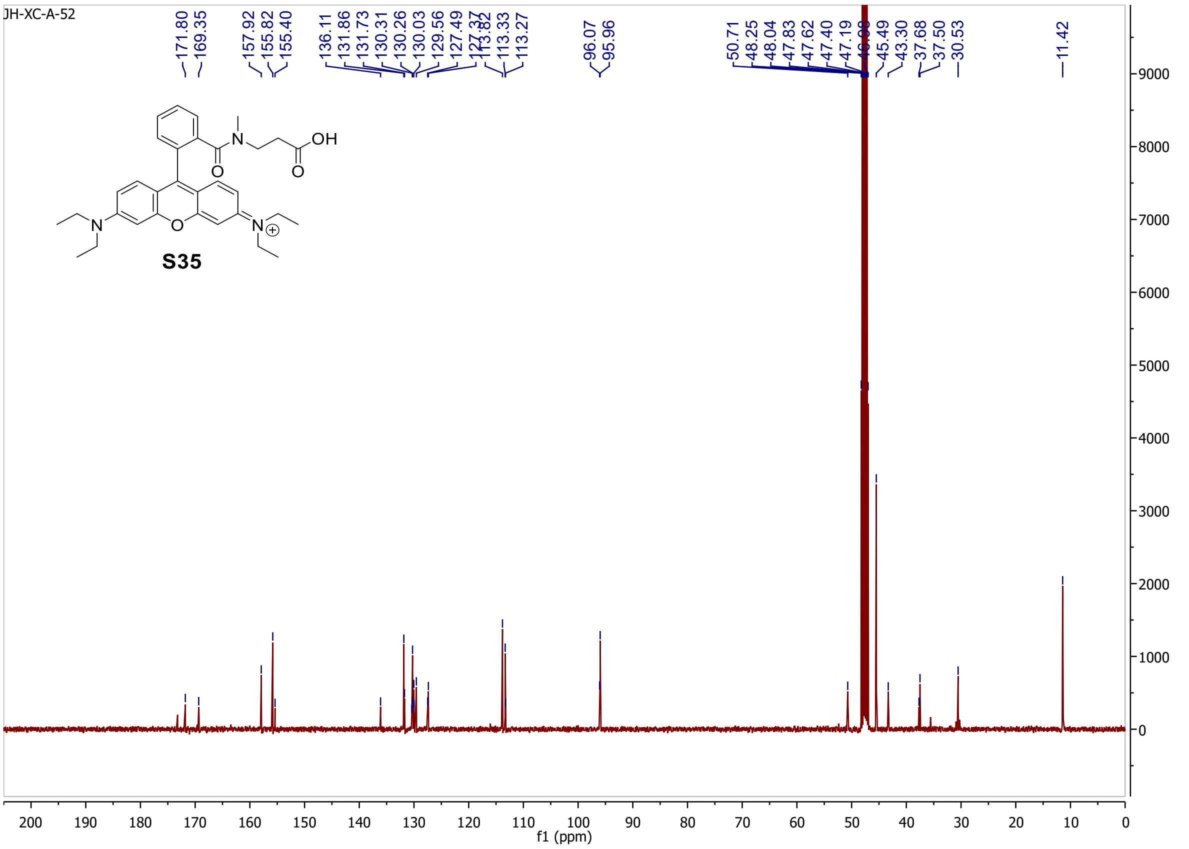
JH-XC-A-52
171.80
169.35
157.92
155.82
155.40
136.11
131.86
131.73
130.31
130.26
130.03
129.56
127.49
113.33
113.27
96.07
95.96
50.71
48.25
48.04
47.83
47.62
47.40
47.19
45.49
43.30
37.68
37.50
30.53
11.42
S35
f1 (ppm)

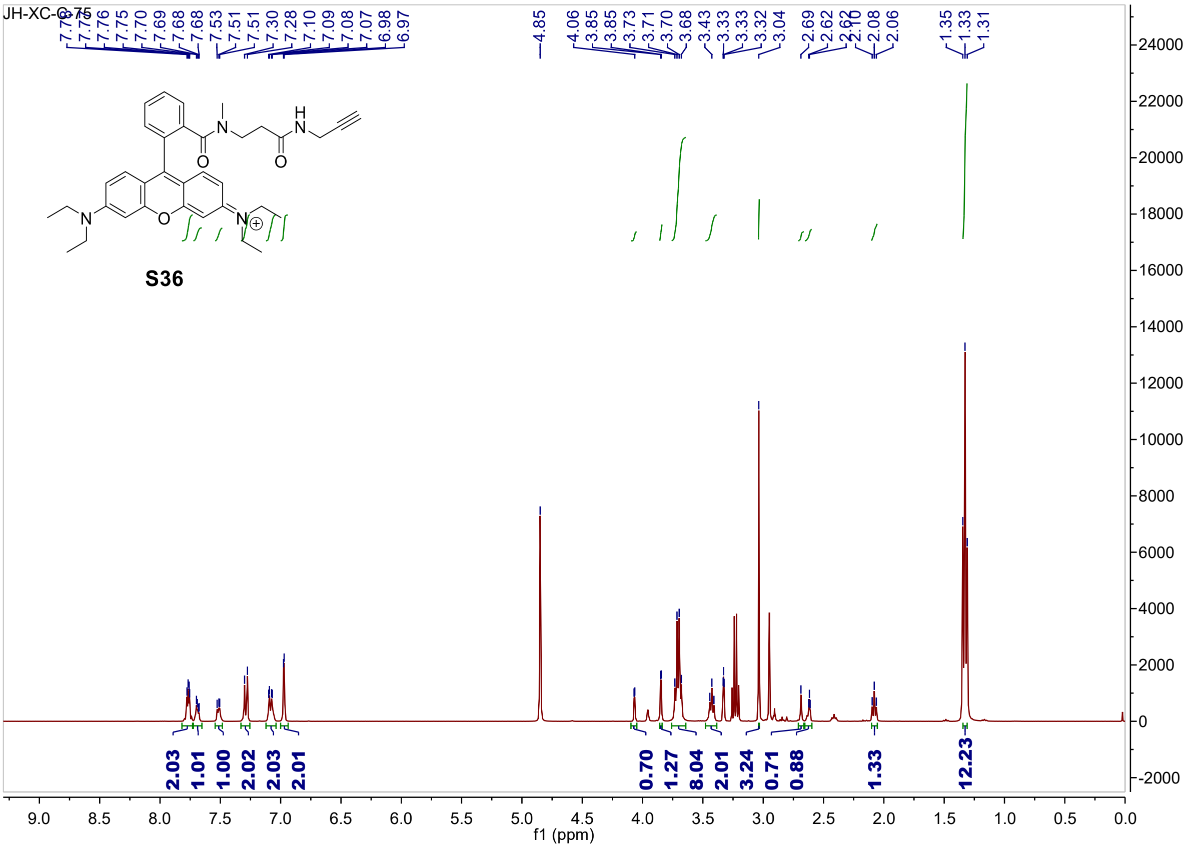
JH-XC-C-75
S36
f1 (ppm)

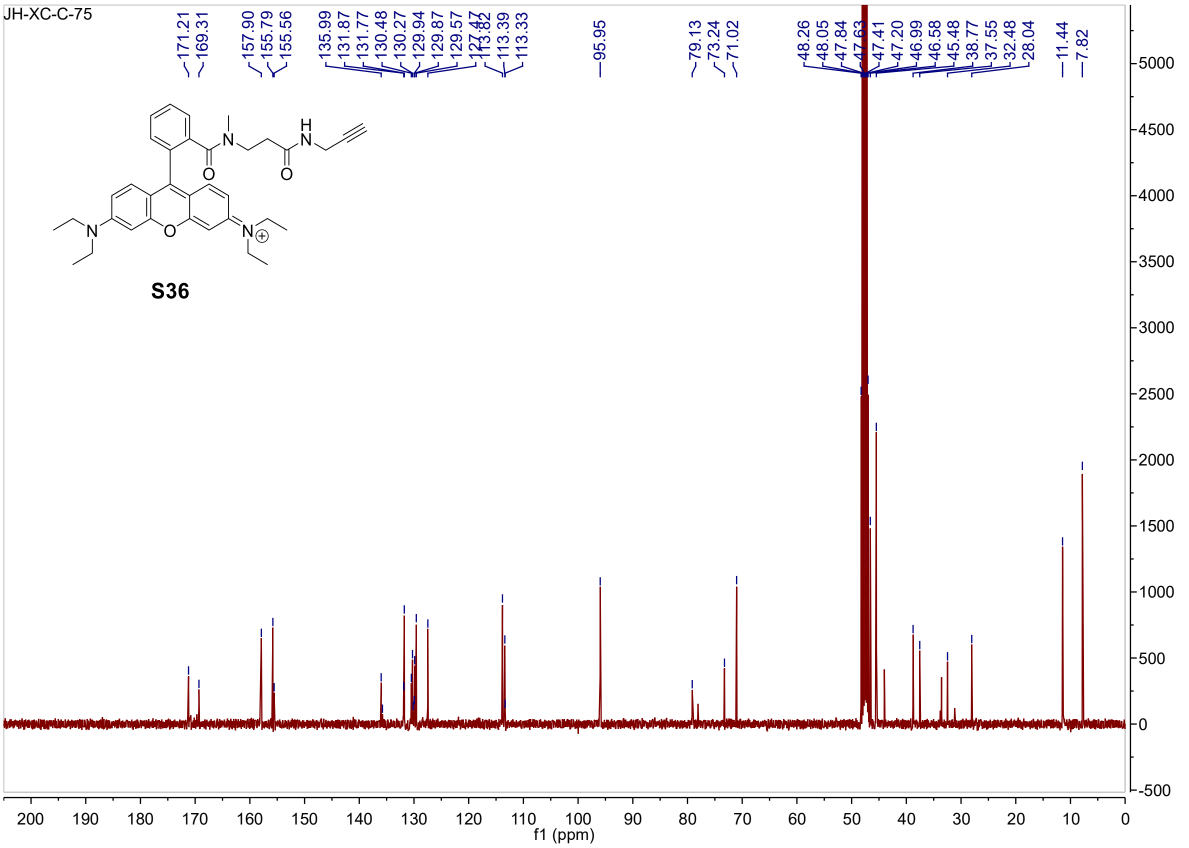
JH-XC-C-75
S36
f1 (ppm)

5 References

[1] X. Chen, S. Abakumov, M. S. Wranne, V. Goyvaerts, M. H. Lauer, J. Rubberecht, T. Rohand, V. Leen, F. Westerlund, J. Hofkens, *J. Am. Chem. Soc.* **2025**, *147*, 384–396.

[2] J. Deen, W. Sempels, R. De Dier, J. Vermant, P. Dedecker, J. Hofkens, R. K. Neely, *ACS Nano* **2015**, *9*, 809–816.

[3] A. Bouwens, J. Deen, R. Vitale, L. D'Huys, V. Goyvaerts, A. Descloux, D. Borrenberghs, K. Grussmayer, T. Lukes, R. Camacho, J. Su, C. Ruckebusch, T. Lasser, D. V. de Ville, J. Hofkens, A. Radenovic, K. P. F. Janssen, *NAR Genomics Bioinforma.* **2020**, *2*, lqz007.

[4] C. Dalhoff, G. Lukinavičius, S. Klimašauskas, E. Weinhold, *Nat. Protoc.* **2006**, *1*, 1879–1886.

[5] S. Avraham, L. Schütz, L. Käver, A. Dankers, S. Margalit, Y. Michaeli, S. Zirkin, D. Torchinsky, N. Gilat, O. Bahr, G. Nifker, M. Koren-Michowitz, E. Weinhold, Y. Ebenstein, *ChemBioChem* **2023**, *24*, e202300400.

[6] E. Haapala, K. Hakala, E. Jokipelto, J. Vilpo, J. Hovinen, *Chem. Res. Toxicol.* **2001**, *14*, 988–995.

[7] G. Lukinavičius, V. Lapienė, Z. Staševskij, C. Dalhoff, E. Weinhold, S. Klimašauskas, *J. Am. Chem. Soc.* **2007**, *129*, 2758–2759.
